# The burden and trend of diseases and their risk factors in Australia, 1990–2019: a systematic analysis for the Global Burden of Disease Study 2019

**DOI:** 10.1016/S2468-2667(23)00123-8

**Published:** 2023-07-27

**Authors:** GBD 2019 Australia Collaborators

## Abstract

**Background:**

A comprehensive understanding of temporal trends in the disease burden in Australia is lacking, and these trends are required to inform health service planning and improve population health. We explored the burden and trends of diseases and their risk factors in Australia from 1990 to 2019 through a comprehensive analysis of the Global Burden of Disease Study (GBD) 2019.

**Methods:**

In this systematic analysis for GBD 2019, we estimated all-cause mortality using the standardised GBD methodology. Data sources included primarily vital registration systems with additional data from sample registrations, censuses, surveys, surveillance, registries, and verbal autopsies. A composite measure of health loss caused by fatal and non-fatal disease burden (disability-adjusted life-years [DALYs]) was calculated as the sum of years of life lost (YLLs) and years of life lived with disability (YLDs). Comparisons between Australia and 14 other high-income countries were made.

**Findings:**

Life expectancy at birth in Australia improved from 77·0 years (95% uncertainty interval [UI] 76·9–77·1) in 1990 to 82·9 years (82·7–83·1) in 2019. Between 1990 and 2019, the age-standardised death rate decreased from 637·7 deaths (95% UI 634·1–641·3) to 389·2 deaths (381·4–397·6) per 100 000 population. In 2019, non-communicable diseases remained the major cause of mortality in Australia, accounting for 90·9% (95% UI 90·4–91·9) of total deaths, followed by injuries (5·7%, 5·3–6·1) and communicable, maternal, neonatal, and nutritional diseases (3·3%, 2·9–3·7). Ischaemic heart disease, self-harm, tracheal, bronchus, and lung cancer, stroke, and colorectal cancer were the leading causes of YLLs. The leading causes of YLDs were low back pain, depressive disorders, other musculoskeletal diseases, falls, and anxiety disorders. The leading risk factors for DALYs were high BMI, smoking, high blood pressure, high fasting plasma glucose, and drug use. Between 1990 and 2019, all-cause DALYs decreased by 24·6% (95% UI 21·5–28·1). Relative to similar countries, Australia's ranking improved for age-standardised death rates and life expectancy at birth but not for YLDs and YLLs between 1990 and 2019.

**Interpretation:**

An important challenge for Australia is to address the health needs of people with non-communicable diseases. The health systems must be prepared to address the increasing demands of non-communicable diseases and ageing.

**Funding:**

Bill & Melinda Gates Foundation.

## Introduction

Australians have one of the highest life expectancies globally (82·5 years), ranking sixth among 35 Organisation for Economic Co-operation and Development (OECD) countries in 2015.^1^ Life expectancy at birth in Australia has been increasing steadily over the past three decades and is one of the highest in the world.^2^ This high life expectancy might be partially attributable to the high quality of health care in Australia. Australia's health-care system is one of the best in the world and is ranked fifth by the 2017 Global Burden of Disease Healthcare Access and Quality Index^3^ and seventh in the 2020 Bloomberg Global Health Index.^4^ However, the per-capita out-of-pocket health expenditure in Australia increased from US$343 in 2000 to $867 in 2019 and ranked eighth highest globally.^5^, ^6^, ^7^

Despite these positive health and health-care metrics, about half of Australia's population have at least one chronic health condition such as cancer, cardiovascular disease, diabetes, musculoskeletal problems, asthma, or poor mental health.^8^ A variety of risk factors contribute to the high burden of non-communicable diseases in the Australian population. For example, dietary choices influence the risk of obesity and hypertension.^9^ An estimated 95% of Australian adults do not consume sufficient amounts of fruit or vegetables, 63% have overweight or obesity, 52% are insufficiently physically active, and 17% do not adhere to guidelines for diet, physical activity, and alcohol consumption.^8^, ^10^, ^11^ Diseases and their risk factors have public health consequences and can negatively affect the health-care system and economic productivity. Understanding the burden and trends of diseases and their risk factors is therefore essential. Furthermore, monitoring these trends to assess the performance of initiatives aimed at reducing disease burden and anticipate future needs is crucial.

**Research in context**

**Evidence before this study**

Australia has one of the best health-care systems in the world, with every citizen having access to universal health coverage. Australians have one of the highest life expectancies, and their quality of life is one of the best globally. Health expenditure per capita has, however, increased substantially over the past few decades and currently is one of the highest in the world. To ensure continuous delivery of effective and high-quality care and guide preventive efforts to improve population health, understanding the burden of disease and its risk factors in Australia is crucial. Further, understanding how population health has changed over time is essential. The Australian Institute of Health and Welfare first released the Australian Burden of Disease Study (ABDS) in 1996 followed by 2003, 2011, 2015, 2018, and 2022 and compared the burden of disease in Australia with previous reports. The ABDS 2022 study included only point-in-time estimates for 220 conditions, providing incomplete information about trends over time. Additionally, this study did not consider the effect of confounders or differences in metadata between study periods. Finally, these estimates are not directly comparable with other international studies because they were not generated using a standardised methodology. To improve upon these previous studies, we used standardised, globally comparable, and robust estimates from the Global Burden of Disease Study to examine the burden and trends of diseases, injuries, and risk factors in Australia from 1990 to 2019.

**Added value of this study**

Our study represents the most extensive analysis of disease burden in Australia using standardised and globally comparable metrics. We present a comprehensive assessment of premature death, morbidity and disability, and the risk factors contributing to poor health in the Australian population by analysing 286 causes of death, 369 diseases and injuries, and 87 behavioural, metabolic, and environmental and occupational risks. To understand Australia's health system performance, we compared the burden of disease and life expectancy in Australia with that of similar Socio-demographic Index countries. Our study highlights key areas of progress, important challenges, and crucial aspects of the health systems in Australia.

**Implications of all the available evidence**

Estimates of disease burden in Australia provide valuable evidence to guide population health policies and health-care priorities. Non-communicable diseases and behavioural and metabolic risk factors contributed the most towards mortality and morbidity in the Australian population. As life expectancy at birth continues to increase and mortality decreases, functional health loss associated with ageing, such as falls, low back pain, and age-related hearing loss, continue to contribute to the burden of disease in Australia. Poor mental health and drug use disorders, as well as self-harm and interpersonal violence are among the emerging health issues in Australia. Preventive and remedial health policies and practices addressing these issues are needed to ensure the sustainability of the Australian health-care system.

Data on disease burden and mortality in Australia are primarily available from the Australian Institute of Health and Welfare (AIHW). In December, 2022, the AIHW released the Australian Burden of Disease Study (ABDS) 2022,^12^ which analysed the burden of 220 diseases in Australia from 2003 to 2022 using the most recent data available at the time of analysis and making inferences about the time periods that did not have complete data. Unfortunately, ABDS estimates cannot be directly compared with international studies because of methodological differences, including the absence of uncertainty estimation and the absence of a sociodemographic index within the ABDS. The Global Burden of Disease Study (GBD) quantifies the contribution of specific diseases, injuries, and risk factors to the overall burden of disease in 204 countries and territories over time and with regular updates.^13^ The GBD systematically accounts for variability between data sources and potential biases within the data and assesses the burden of disease in all populations using a common computational framework, generating estimates that are comparable across time, age groups, sex, and locations.^13^ Some countries, such as Brazil,^14^ Canada,^15^ China,^16^ Russia,^17^ the UK,^18^ and the USA,^19^ have used GBD estimates to assess national health outcomes. Other studies have assessed the burden of specific diseases^20^ or risk factors^21^ in the Australian population using GBD data; however, no previous study provides a comprehensive assessment of the prevalence and trends of all diseases, injuries, and risk factors using the most recent round of GBD estimates.

Understanding the major diseases and their risk factors and how they are changing over time is crucial to inform both national health policy and health-care delivery in Australia. Additionally, understanding the key risk factors has potential for multisectoral action outside the health-care system to improve health and reduce the cost and demand on the system. Therefore, this study aimed to systematically analyse the burden of disease and risk factors in Australia from 1990 to 2019 using the comprehensive GBD methodology. Comparisons with similar high Socio-demographic Index (SDI) countries were also made. This manuscript was produced as part of the GBD Collaborator Network and in accordance with the GBD Protocol.^13^

## Methods

### Overview

We extracted the country-specific burden of disease data for Australia from GBD 2019 using publicly available GBD resources: the Global Health Data Exchange (GHDx); GBD Results; and GBD Compare. Data sources included primarily vital registration systems with additional data from sample registrations, censuses, surveys, surveillance, registries, and verbal autopsies. The GBD 2019 data sources for Australian are listed in the appendix (pp 22–110) and on the GHDx. Using a comprehensive and systematic approach, GBD 2019 analysed 286 causes of death, 369 diseases and injuries, and 87 behavioural, metabolic, and environmental and occupational risk factors for 204 countries and territories, including Australia.^22^ A total of 1020 data sources were used in the analyses reported here (appendix pp 22–110). We employed the following metrics to evaluate the burden of disease in Australia between 1990 and 2019: all-cause mortality, cause-specific mortality, years of life lost (YLLs), years of life lived with disability (YLDs), disability-adjusted life-years (DALYs), and associated risk factors. We also estimated the life expectancy at birth and healthy life expectancy (HALE). Finally, we assessed GBD 2019 estimates for 14 similar countries and compared these estimates to those of Australia between 1990 and 2019. The GBD 2019 estimates did not undergo any additional processing or analysis.

### Mortality

The cause of death database, which was developed and implemented for GBD 2019, was used to produce cause-specific estimates of mortality.^22^ Data were evaluated for completeness and misclassification using a star rating, cleaned, disaggregated, and mapped to the International Classification of Diseases codes.^22^ Deaths with non-specific or impossible codes, termed garbage codes, were redistributed to appropriate disease categories in the GBD cause hierarchy before modelling. A complete list of the data sources used to generate mortality estimates for Australia can be found in the appendix (p 52). We used the Cause of Death Ensemble model to estimate cause-specific mortality for each combination of sex, age, location, and year. To calculate YLLs, a measure of premature death, the sum of each death within each age group was multiplied by the normative standard reference life expectancy at each age.^22^

### Morbidity

A Bayesian meta-regression method (DisMod-MR 2.1) was used to generate most of the prevalence and incidence estimates for each combination of sex, age, location, and year. Custom models were built for some specific diseases because DisMod-MR 2.1 was either unable to capture the complexity of the disease or the incidence and prevalence needed to be computed from other data, or both.^22^ Details about the DisMod-MR 2.1 tool and the custom models are available in the appendix (p 111). Subsequently, YLDs were calculated from prevalence estimates using a corresponding disability weight on the basis of disease severity and comorbidity.

### Life expectancy, DALYs, and HALE

Life expectancy was estimated on the basis of expected mortality rates computed using the meta-regression tool (meta-regression-Bayesian regularised trimmed) to analyse the relationship between log-mortality rates and SDI.^22^, ^23^ DALYs were computed as the sum of YLLs and YLDs, with one DALY representing 1 lost year of healthy life. HALE is a summary metric reflecting age-specific mortality and morbidity and represents the number of years a person of a given age can expect to live in good health. HALE was calculated using the standard GBD methodology.^23^

### GBD causes and risk factors hierarchy

GBD 2019 used a four-level hierarchy of causes of diseases and injuries and causes of deaths. There were three level-1 causes (ie, communicable, maternal, neonatal, and nutritional diseases, non-communicable diseases, and injuries), 22 level-2 causes (eg, cardiovascular diseases and mental disorders), 174 level-3 causes (eg, stroke and depressive disorders), and 301 level-4 causes (eg, ischaemic stroke and major depression).^22^ In this study, we estimated the extent of mortality and DALYs attributable to 87 risk factors, which are categorised using three level-1 groups (ie, behavioural risks, environmental and occupational risks, and metabolic risks), 20 level-2 groups, 52 level-3 groups, and 69 level-4 groups.^24^ Detailed descriptions of the causes and risk factors hierarchies and the calculation of risk-attributable burden can be found in previous GBD reports.^22^, ^24^ Unless otherwise specified, we present level-3 causes of diseases and level-4 risk factors in the Results section. The definitions and categorisation of diseases, injuries, and risk factors are based on the standard GBD methodology and have been agreed upon by the GBD 2019 Australia Collaborators as a consensus.

### Uncertainty levels

Uncertainty levels were estimated at several stages of the GBD modelling process.^22^ Uncertainty for mortality and YLLs reflected uncertainty in the levels of all-cause mortality. Uncertainty in the disability weight for each sequela was propagated into the estimates of YLDs for each disease and injury. We reported 95% uncertainty intervals (UIs) derived from 1000 draws from the posterior distribution of each step in the estimation process according to the established GBD methodology, with the lower and upper UIs defined as the 25th and 975th ranked values, respectively, from the 1000 ordered draws.

### SDI and expected mortality analysis

GBD 2019 used SDI to compare health progress between locations. SDI is a composite measure based on per-capita income, average educational attainment of individuals aged 15 years or older, and fertility rates of females under the age of 25 years. SDI indicates sociodemographic and economic development and is expressed on a scale from 0 (lowest SDI) to 100 (highest SDI).^25^ Using a Gaussian process regression with a linear before the mean function SDI was used to calculate expected mortality rates and YLDs. We compared these values with the observed values to identify locations and causes for which improvements were greater or less than what would be expected based on SDI alone.

### Disability weights, star rating of data sources, and annual rates of change

To compute YLDs for each disease and injury, prevalence estimates were multiplied by a disability weight representing the magnitude of health loss associated with the sequelae of each condition. Disability weights are based on survey responses and measured on a scale from 0 to 1, with 0 corresponding to full health and 1 representing death.^22^ Additionally, the quality of the input data used to generate causes of death estimates was assessed using a simple star rating system from 0 to 5. These ratings are based on the percentage of garbage codes and the level of completeness in death registries.^22^ Finally, to show changes over time, we present annual rates of change as the difference in the natural log of the values at the start and end of the time interval divided by the number of years in the interval.^22^

### Comparisons with countries with high SDI

We compared Australia with 14 similarly high-SDI countries in terms of age-standardised rates of all-cause mortality, YLLs, and YLDs as well as life expectancy and HALE between 1990 and 2019. These comparator countries were selected on the basis of high SDI ranging from 0·84 to 0·93 and included Canada, Denmark, Finland, Germany, Iceland, Ireland, the Netherlands, New Zealand, Norway, Singapore, Sweden, Switzerland, the USA, and the UK. All estimates and SDI values used in this comparative analysis were obtained from GBD 2019.^13^

### Data availability, transparency, and quality

Our study follows the Guidelines for Accurate and Transparent Health Estimates Reporting to ensure transparency and reproducibility of the results (appendix p 13). All data sources, computer codes, and results used here are publicly available via the GHDx. Limitations associated with the GBD methodology and information about data completeness and quality have been described previously.^23^, ^24^, ^26^ The complete GBD protocol is publicly available and all estimates can be further explored and visualised using GBD Compare and downloaded via GBD Results.^13^

### Role of the funding source

The funder of the study had no role in study design, data collection, data analysis, data interpretation, or writing of this report.

## Results

In Australia, life expectancy at birth increased from 77·0 years (95% UI 76·9–77·1) in 1990 to 82·9 years (82·7–83·1) in 2019. Females in Australia had a higher life expectancy at birth compared to males between 1990 (80·1 years, 95% UI 80·2–80·1 *vs* 73·9 years, 74·0–73·8) and 2019 (85·0 years, 85·2–84·8 *vs* 80·8 years, 81·0–80·6). In 2019, HALE at birth was 70·3 healthy years (95% UI 66·7–73·4), which was also higher for females (71·2 years, 67·4–74·6) than males (69·4 years, 66·1–72·2) compared with 1990, which was 66·1 healthy years (62·9–68·9) in both sexes; 64·2 healthy years (61·5–66·7) in males and 67·9 healthy years (64·3–70·1) in females.

The age-standardised death rate per 100 000 population declined by 39·0% (37·6–40·3) between 1990 and 2019 (table 1). The age-standardised rate of YLLs declined from 14 420·1 (95% UI 14 323·6–14 514·5) in 1990 to 8041·5 (7810·9–8295·3) in 2019 with a decrease of 44·2% (95% UI 42·5–45·9) during this period. Although during 1990–98 YLLs contributed relatively more to the total DALYs, in 1999 the contribution of YLDs exceeded that of YLLs in Australia. Between 1999 and 2019, the relative contribution of YLLs to DALYs continued to decline compared to the contribution of YLDs (table 1).

**Table 1:** Age-standardised rate of deaths, DALYs, YLDs, and YLLs per 100 000 population in Australia between 1990 and 2019

	**Deaths**	**DALYs**	**YLDs**	**YLLs**
1990	637·7 (634·1–641·3)	26 008·9 (23 079·0–29 310·8)	11 588·8 (8638·9–14 969·0)	14 420·1 (14 323·6–14 514·5)
1991	616·3 (612·7–619·7)	25 401·6 (22 438·5–28 815·2)	11 571·6 (8624·6–14 949·9)	13 830·0 (13 744·2–13 917·6)
1992	609·2 (605·8–612·7)	25 125·7 (22 184·4–28 523·1)	11 559·8 (8601·4–14 944·1)	13 565·9 (13 482·5–13 653·7)
1993	595·7 (592·2–599·2)	24 686·1 (21 739·9–28 053·1)	11 551·0 (8593·5–14 934·1)	13 135·0 (13 048·6–13 221·4)
1994	593·4 (589·9–596·7)	24 564·3 (21 597·9–27 937·6)	11 552·3 (8563·6–14 936·9)	13 012·0 (12 931·9–13 093·1)
1995	579·9 (576·8–583·0)	24 253·8 (21 265·6–27 644·8)	11 554·1 (8575·4–14 942·1)	12 699·7 (12 626·1–12 780·4)
1996	571·7 (568·0–575·3)	24 087·2 (21 154·9–27 470·0)	11 566·1 (8619·2–14 951·8)	12 521·1 (12 433·9–12 612·0)
1997	556·9 (553·7–560·3)	23 770·2 (20 834·7–27 140·2)	11 579·6 (8612·5–14 969·2)	12 190·7 (12 112·5–12 273·9)
1998	541·1 (538·0–544·5)	23 467·8 (20 514·1–26 861·2)	11 593·5 (8635·6–14 988·8)	11 874·3 (11 794·0–11 954·6)
1999	526·9 (523·7–530·1)	23 180·9 (20 228·1–26 590·6)	11 605·1 (8626·8–15 018·6)	11 575·8 (11 495·0–11 656·1)
2000	513·7 (510·7–517·0)	22 814·9 (19 841·9–26 206·3)	11 614·1 (8631·1–14 987·3)	11 200·8 (11 125·1–11 276·5)
2001	499·7 (496·9–502·8)	22 418·3 (19 447·8–25 823·1)	11 617·9 (8627·3–14 994·5)	10 800·4 (10 727·6–10 875·3)
2002	492·0 (488·8–494·9)	22 164·6 (19 173·5–25 529·0)	11 621·1 (8627·8–14 960·4)	10 543·5 (10 468·8–10 612·0)
2003	478·4 (475·5–481·4)	21 854·6 (18 858·9–25 201·9)	11 619·3 (8623·9–14 966·9)	10 235·3 (10 166·8–10 307·0)
2004	464·3 (461·4–467·1)	21 545·0 (18 559·0–24 896·3)	11 619·7 (8636·3–14 974·6)	9925·3 (9852·4–9991·8)
2005	450·7 (447·9–453·6)	21 262·7 (18 278·2–24 605·6)	11 616·0 (8664·9–14 947·7)	9646·7 (9573·1–9724·3)
2006	441·7 (438·8–444·3)	21 024·3 (18 054·1–24 337·8)	11 608·4 (8640·0–14 946·6)	9415·9 (9341·8–9490·5)
2007	439·7 (436·7–442·5)	20 923·7 (17 932·0–24 303·5)	11 598·8 (8615·5–14 950·9)	9324·9 (9251·7–9395·8)
2008	436·0 (433·4–438·9)	20 798·0 (17 848·6–24 206·8)	11 583·2 (8634·5–14 965·6)	9214·8 (9149·7–9279·2)
2009	426·7 (424·1–429·3)	20 643·9 (17 693·2–24 048·3)	11 570·1 (8601·4–14 961·8)	9073·8 (9011·5–9134·1)
2010	417·2 (414·8–419·7)	20 403·9 (17 468·7–23 775·3)	11 561·3 (8612·9–14 953·4)	8842·5 (8782·4–8900·1)
2011	411·5 (408·8–414·0)	20 201·4 (17 262·6–23 612·7)	11 541·7 (8592·5–14 917·8)	8659·7 (8595·3–8716·5)
2012	401·2 (398·9–403·8)	19 895·2 (16 971·9–23 267·7)	11 500·4 (8559·9–14 875·2)	8394·8 (8337·9–8454·5)
2013	395·0 (392·5–397·3)	19 744·5 (16 820·1–23 125·5)	11 454·3 (8536·1–14 822·6)	8290·2 (8233·2–8347·3)
2014	394·7 (392·3–397·2)	19 684·7 (16 768·5–23 022·3)	11 419·0 (8493·2–14 797·2)	8265·7 (8209·8–8324·0)
2015	394·7 (392·0–397·5)	19 666·8 (16 758·6–22 987·3)	11 405·5 (8483·0–14 748·8)	8261·3 (8194·9–8329·0)
2016	386·8 (383·3–390·6)	19 542·3 (16 631·3–22 932·4)	11 472·3 (8525·8–14 887·4)	8069·9 (7979·9–8165·5)
2017	385·0 (379·3–390·9)	19 564·4 (16 647·9–22 876·5)	11 548·4 (8601·6–14 929·8)	8015·9 (7863·0–8174·9)
2018	388·8 (382·4–395·8)	19 622·7 (16 671·2–22 991·8)	11 563·0 (8624·5–14 935·2)	8059·7 (7876·8–8261·0)
2019	389·2 (381·4–397·6)	19 607·6 (16 655·7–23 005·3)	11 566·2 (8612·7–14 935·9)	8041·5 (7810·9–8295·3)

Data are presented as age-standardised rate per 100 000 (95% UI). DALYs are the sum of YLDs and YLLs. DALYs=disability-adjusted life-years. YLDs=years of life lived with disability. YLLs=years of life lost. UI=uncertainty interval.

Non-communicable diseases remained the primary cause of mortality in Australia between 1990 and 2019 with 90·9% (95% UI 90·4–91·9) of total deaths attributable to non-communicable diseases, followed by injuries (5·7%, 5·3–6·1) and communicable, maternal, neonatal, and nutritional diseases (3·3%, 2·9–3·7) in 2019. Age-standardised mortality rates, however, declined by 39·4% (95% UI 38·1–40·7) for non-communicable diseases, 36·1% (30·1–42·5) for communicable, maternal, neonatal, and nutritional diseases, and 34·5% (31·7–37·6) for injuries from 1990 to 2019. Between 1990 and 2019, the age-standardised rate of YLLs declined by 43·2% (95% UI 41·5–44·8) for non-communicable diseases, 52·3% (45·6–58·8) for communicable, maternal, neonatal, and nutritional diseases, and 45·9% (43·8–48·0) for injuries.

Between 1990 and 2019, ischaemic heart disease was ranked consistently as the first leading cause of death, stroke as the second leading cause of death, and tracheal, bronchus, and lung cancer as the third leading causes of death, in terms of age-standardised death rates (table 2). In 2019, 11 of the top 25 causes of death in Australia were cancers (ranked on the basis of age-standardised rates). Between 1990 and 2019, age-standardised death rates decreased by more than 50% for three causes, comprising ischaemic heart disease, stroke, and road injuries. Road injuries declined by 54·3% (51·0–65·5) between 1990 and 2010 and 19·3% (14·3–24·5) between 2010 and 2019 with an overall decline of 63·2% (61·0–65·5) between 1990 and 2019. Age-standardised mortality rates increased substantially for five causes between 1990 and 2019, comprising pancreatic cancer, falls, Parkinson's disease, endocrine, metabolic, blood, and immune disorders, and liver cancer.

**Table 2:** Age-standardised rate (95% UI) of deaths with percentage changes between 1990 and 2010, 2010 and 2019, and 1990 and 2019 for the leading causes of diseases, disabilities, and injuries in Australia

	**Disease rank based on death rates**			**Deaths per 100 000 population (95% UI)**			**Age-standardised percentage change in deaths (95% UI)**		
	1990	2010	2019	1990	2010	2019	1990–2010	2010–19	1990–2019
Ischaemic heart disease	1	1	1	177·2 (163·8 to 184·4)	67·3 (58·8 to 71·9)	56·1 (48·6 to 60·4)	−62·0% (−64·0 to −60·5)	−16·7% (−20·0 to −13·7)	−68·4% (−70·4 to −66·7)
Stroke	2	2	2	62·7 (56·3 to 66·2)	30·5 (26·4 to 33·0)	26·2 (21·9 to 28·9)	−51·3% (−54·2 to −49·0)	−14·2% (−19·8 to −8·2)	−58·2% (−61·8 to −54·9)
Tracheal, bronchus, and lung cancer	3	3	3	33·1 (32·0 to 34·0)	25·9 (24·3 to 26·9)	23·5 (21·7 to 25·0)	−21·8% (−24·9 to −18·8)	−9·4% (−14·2 to −4·5)	−29·1% (−33·6 to −24·8)
Alzheimer's disease and other dementias	6	4	4	22·1 (5·6 to 55·4)	22·5 (5·6 to 59·8)	22·0 (5·7 to 55·9)	−2·0% (−7·9 to 6·6)	−0·2% (−4·7 to 5·1)	−2·2% (−7·9 to 6·5)
Chronic obstructive pulmonary disease	4	5	5	29·8 (27·6 to 31·7)	19·7 (17·0 to 22·0)	19·8 (16·6 to 22·7)	−33·9% (−40·0 to −26·0)	0·7% (−9·1 to 11·3)	−33·5% (−42·1 to −23·7)
Colon and rectum cancer	5	6	6	23·4 (22·2 to 24·2)	16·0 (14·7 to 16·8)	15·5 (14·1 to 16·7)	−31·5% (−34·6 to −28·9)	−3·2% (−8·7 to 2·5)	−33·7% (−37·9 to −29·6)
Chronic kidney disease	13	10	7	10·1 (8·7 to 11·0)	9·5 (8·6 to 10·1)	10·8 (9·0 to 12·1)	6·6% (−1·3 to 14·6)	6·3% (−2·6 to 16·2)	13·3% (1·9 to 26·4)
Self–harm	9	8	8	10·9 (10·6 to 11·2)	12·8 (12·5 to 13·2)	10·4 (9·9 to 11·1)	−14·8% (−18·2 to −11·6)	−4·7% (−9·8 to 2·0)	−18·8% (−23·3 to −13·3)
Prostate cancer	11	9	9	11·3 (8·7 to 13·8)	10·3 (8·3 to 13·6)	9·7 (8·0 to 13·9)	−9·1% (−19·3 to 6·6)	−5·3% (−15·0 to 4·0)	−13·9% (−24·8 to 9·5)
Diabetes	10	7	10	11·1 (9·9 to 11·8)	11·6 (10·7 to 12·1)	9·2 (8·2 to 10·0)	−4·3% (−9·8 to 0·8)	−16·5% (−21·9 to −10·9)	−20·1% (−25·8 to −14·0)
Breast cancer	8	11	11	13·8 (13·1 to 14·4)	9·9 (9·1 to 10·4)	9·1 (8·3 to 9·9)	−28·7% (−32·0 to −25·3)	−7·3% (−13·0 to −1·1)	−33·9% (−38·3 to −29·2)
Lower respiratory infections	12	12	12	10·7 (9·6 to 11·5)	8·2 (7·0 to 8·9)	8·7 (7·3 to 9·8)	−23·4% (−28·4 to −18·4)	6·6% (−2·3 to 16·4)	−18·3% (−26·9 to −10·4)
Pancreatic cancer	14	13	13	7·5 (7·2 to 7·8)	8·0 (7·4 to 8·4)	8·3 (7·4 to 9·1)	6·3% (1·9 to 10·7)	2·9% (−5·2 to 12·1)	9·5% (1·1 to 19·7)
Falls	24	14	14	5·3 (4·7 to 5·7)	7·3 (6·3 to 7·9)	7·5 (6·3 to 8·3)	36·5% (25·7 to 47·0)	3·3% (−4·4 to 11·7)	41·0% (27·0 to 55·5)
Atrial fibrillation	16	16	15	7·5 (5·9 to 8·4)	6·9 (5·4 to 8·2)	6·8 (5·4 to 8·2)	−7·6% (−14·2 to 10·1)	−1·7% (−6·7 to 3·3)	−9·2% (−16·7 to 8·4)
Cirrhosis and other chronic liver diseases	17	17	16	7·4 (7·0 to 7·7)	5·9 (5·5 to 6·2)	5·8 (5·3 to 6·3)	−20·5% (−25·2 to −15·0)	−2·0% (−9·0 to 5·5)	−22·1% (−28·5 to −15·0)
Road injuries	7	15	17	15·5 (15·2 to 15·9)	7·1 (6·9 to 7·3)	5·7 (5·4 to 6·1)	−54·3% (−56·0 to −52·6)	−19·3% (−24·5 to −14·3)	−63·2% (−65·5 to −61·0)
Parkinson's disease	28	18	18	5·1 (4·5 to 5·4)	4·6 (4·2 to 4·8)	5·1 (4·4 to 5·4)	11·0% (5·5 to 15·7)	−0·9% (−5·7 to 4·0)	10·1% (2·7 to 16·8)
Leukaemia	22	20	19	4·7 (4·3 to 4·9)	6·0 (5·7 to 6·3)	4·8 (4·3 to 5·2)	−22·1% (−25·9 to −18·5)	2·0% (−5·7 to 10·6)	−20·5% (−27·3 to −14·2)
Endocrine, metabolic, blood, and immune disorders	40	21	20	4·6 (3·6 to 5·5)	2·6 (2·1 to 3·4)	4·8 (3·7 to 5·7)	76·9% (51·4 to 90·0)	2·8% (−4·1 to 10·0)	81·9% (60·2 to 98·7)
Non–Hodgkin lymphoma	21	19	21	4·9 (4·5 to 5·3)	6·5 (6·1 to 6·9)	4·6 (4·0 to 5·1)	−24·6% (−29·4 to −20·0)	−7·4% (−17·0 to 3·3)	−30·2% (−38·5 to −22·2)
Brain and CNS cancer	25	22	22	4·5 (3·3 to 5·0)	5·0 (4·4 to 6·2)	4·4 (3·2 to 4·9)	−9·5% (−40·6 to −1·4)	−3·4% (−10·5 to 3·9)	−12·6% (−45·2 to −2·1)
Malignant skin melanoma	29	25	23	4·3 (3·3 to 5·9)	4·5 (2·9 to 5·2)	4·2 (2·9 to 5·4)	3·6% (−26·7 to 13·2)	−6·4% (−15·9 to 4·8)	−3·1% (−29·2 to 15·5)
Liver cancer	49	28	24	2·0 (1·9 to 2·0)	3·9 (3·6 to 4·1)	4·2 (3·8 to 4·6)	98·5% (86·5 to 110·6)	8·1% (0·0 to 17·4)	114·6% (95·9 to 133·8)
Other malignant neoplasms	26	23	25	4·9 (4·5 to 5·3)	4·5 (3·9 to 4·8)	4·1 (3·6 to 4·5)	−6·7% (−19·4 to −1·7)	−8·3% (−13·5 to −2·7)	−14·5% (−24·8 to −7·7)

Note that causes are ordered by their ranking in 2019. UI=uncertainty interval.

Ischaemic heart disease, self-harm, tracheal, bronchus, and lung cancer, stroke, and colorectal cancer were the five leading causes of YLLs, and ten of the top 25 causes of YLLs were cancers in 2019 (table 3). Although ischaemic heart disease remained the leading cause of YLLs in 1990, 2010, and 2019, the age-standardised rate of YLLs caused by ischaemic heart disease decreased by 71·9% (70·6–73·3) between 1990 and 2019. Ranked third in 1990, road injuries declined by 66·0% (63·6–68·4) in terms of the age-standardised rate of YLLs and were ranked eighth in 2019. By contrast, the ranking of self-harm based on YLLs increased from sixth in 1990 to third in 2010 and to second in 2019, although the age-standardised rate of YLLs decreased by 19·5% (14·0–19·5) between 1990 and 2019. Between 1990 and 2019, age-standardised YLLs declined by more than 50% for five of the top 25 causes, comprising ischaemic heart disease, stroke, neonatal disorders, road injuries, and congenital birth defects. Age-standardised YLLs increased substantially for four causes between 1990 and 2019, comprising drug use disorders, endocrine, metabolic, blood, and immune disorders, falls, and liver cancer.

**Table 3:** Age-standardised rate (95% UI) of YLLs with percentage changes between 1990 and 2010, 2010 and 2019, and 1990 and 2019 for the leading causes of diseases, disabilities, and injuries in Australia

	**YLL rank**			**YLLs per 100 000 population (95% UI)**			**Age-standardised percentage change in YLLs (95% UI)**		
	1990	2010	2019	1990	2010	2019	1990–2010	2010–19	1990–2019
Ischaemic heart disease	1	1	1	2963·0 (2821·8 to 3041·4)	1004·2 (925·2 to 1050·6)	831·6 (760·9 to 880·9)	−66·1% (−67·4 to −65·0)	−17·2% (−20·6 to −14·1)	−71·9% (−73·3 to −70·6)
Self-harm	6	3	2	623·3 (604·8 to 642·9)	523·0 (508·1 to 537·3)	501·4 (476·1 to 534·8)	−16·1% (−19·7 to −12·7)	−4·1% (−9·6 to 2·7)	−19·5% (−24·1 to −14·0)
Tracheal, bronchus, and lung cancer	4	2	3	758·3 (737·0 to 777·9)	537·8 (513·7 to 554·1)	483·6 (453·6 to 511·0)	−29·1% (−31·7 to −26·7)	−10·1% (−14·6 to −5·1)	−36·2% (−39·9 to −32·5)
Stroke	2	4	4	914·2 (848·9 to 953·2)	401·4 (362·7 to 426·9)	342·3 (304·6 to 372·6)	−56·1% (−58·4 to −53·9)	−14·7% (−20·3 to −8·9)	−62·6% (−65·3 to −59·6)
Colon and rectum cancer	7	7	5	497·4 (479·7 to 511·7)	323·1 (306·1 to 334·2)	308·4 (286·8 to 327·9)	−35·0% (−37·5 to −32·7)	−4·5% (−9·9 to 1·3)	−38·0% (−41·6 to −34·2)
Chronic obstructive pulmonary disease	8	8	6	494·9 (462·4 to 522·3)	294·7 (264·6 to 326·2)	296·1 (257·6 to 334·5)	−40·4% (−45·5 to −33·4)	0·5% (−8·9 to 10·3)	−40·2% (−47·5 to −32·1)
Neonatal disorders	5	5	7	625·8 (580·6 to 684·7)	400·6 (370·6 to 424·8)	291·2 (243·1 to 346·5)	−36·0% (−43·2 to −29·5)	−27·3% (−38·9 to −14·2)	−53·5% (−62·1 to −43·8)
Road injuries	3	6	8	839·7 (816·0 to 864·0)	366·3 (353·3 to 379·4)	285·8 (268·0 to 304·1)	−56·4% (−58·2 to −54·6)	−22·0% (−27·4 to −16·6)	−66·0% (−68·4 to −63·6)
Breast cancer	10	10	9	360·8 (348·4 to 372·0)	244·9 (233·4 to 254·7)	223·9 (207·7 to 239·3)	−32·1% (−34·9 to −29·2)	−8·6% (−14·5 to −2·3)	−37·9% (−42·1 to −33·8)
Alzheimer's disease and other dementias	12	11	10	227·8 (54·4 to 611·5)	222·3 (55·9 to 573·8)	222·0 (56·4 to 560·6)	−2·4% (−8·2 to 5·9)	−0·1% (−4·5 to 4·8)	−2·5% (−8·2 to 5·7)
Congenital birth defects	9	9	11	452·2 (360·4 to 491·2)	268·4 (240·7 to 313·3)	209·9 (174·7 to 264·1)	−40·6% (−46·9 to −19·9)	−21·8% (−32·7 to −8·0)	−53·6% (−61·7 to −29·9)
Drug-use disorders	24	17	12	140·2 (130·9 to 150·7)	145·9 (132·4 to 159·9)	197·7 (174·1 to 230·4)	4·1% (−6·8 to 17·9)	35·5% (17·1 to 57·5)	41·1% (21·4 to 68·2)
Pancreatic cancer	20	14	13	159·0 (152·9 to 164·3)	164·1 (155·8 to 170·3)	167·3 (153·4 to 182·5)	3·2% (−0·9 to 7·4)	2·0% (−5·9 to 10·8)	5·2% (−2·9 to 14·5)
Cirrhosis and other chronic liver diseases	13	13	14	213·9 (203·7 to 222·8)	167·1 (157·9 to 175·5)	160·7 (148·4 to 172·8)	−21·9% (−26·5 to −16·9)	−3·8% (−10·9 to 4·2)	−24·9% (−30·8 to −18·5)
Diabetes	14	12	15	210·7 (201·1 to 219·2)	182·7 (170·1 to 191·2)	154·0 (142·2 to 164·9)	−13·3% (−17·6 to −9·3)	−15·7% (−20·7 to −10·2)	−26·9% (−31·6 to −21·9)
Chronic kidney disease	25	19	16	139·7 (130·5 to 147·2)	133·6 (121·1 to 141·9)	144·2 (127·4 to 159·4)	−4·4% (−10·1 to 1·2)	7·9% (−0·2 to 16·3)	3·2% (−6·0 to 13·7)
Prostate cancer	16	15	17	180·5 (139·2 to 224·7)	158·5 (126·8 to 205·4)	143·7 (121·4 to 204·3)	−12·2% (−26·1 to 1·1)	−9·3% (−19·2 to 1·3)	−20·4% (−30·4 to 1·5)
Brain and central nervous system cancer	18	16	18	172·1 (151·2 to 212·4)	149·4 (114·4 to 164·7)	143·5 (107·5 to 160·3)	−13·2% (−40·8 to −5·4)	−3·9% (−11·0 to 2·8)	−16·6% (−46·0 to −7·2)
Endocrine, metabolic, blood, and immune disorders	36	20	19	86·9 (70·5 to 110·4)	126·6 (107·1 to 165·0)	130·2 (110·2 to 174·7)	45·7% (36·4 to 56·9)	2·9% (−4·0 to 9·9)	49·8% (37·3 to 65·2)
Lower respiratory infections	15	21	20	193·0 (181·4 to 203·8)	126·4 (116·3 to 134·9)	124·0 (110·1 to 135·2)	−34·5% (−38·8 to −30·3)	−1·9% (−9·9 to 6·5)	−35·8% (−42·0 to −29·7)
Other malignant neoplasms	23	18	21	151·1 (140·8 to 163·5)	134·5 (116·4 to 143·9)	120·9 (107·1 to 131·8)	−11·0% (−22·0 to −6·0)	−10·1% (−15·2 to −4·7)	−20·0% (−28·3 to −13·7)
Leukaemia	19	22	22	170·0 (164·1 to 176·2)	120·1 (114·8 to 124·6)	115·6 (107·3 to 122·8)	−29·4% (−32·6 to −26·2)	−3·7% (−10·3 to 3·0)	−32·0% (−36·9 to −27·6)
Falls	35	24	23	93·2 (87·5 to 98·0)	107·0 (97·5 to 113·2)	105·8 (94·0 to 114·4)	14·8% (8·1 to 22·0)	−1·1% (−7·3 to 5·4)	13·5% (4·6 to 22·3)
Malignant skin melanoma	27	23	24	122·0 (92·4 to 159·3)	113·2 (79·4 to 139·2)	104·2 (78·1 to 140·0)	−7·1% (−27·2 to 6·9)	−8·0% (−17·5 to 4·2)	−14·5% (−30·4 to 8·8)
Liver cancer	50	27	25	48·5 (46·6 to 50·7)	91·4 (86·7 to 96·4)	97·8 (89·1 to 107·2)	88·2% (76·7 to 99·9)	7·0% (−1·9 to 17·0)	101·5% (82·0 to 121·7)

Note that causes are ordered by their ranking in 2019. YLLs=years of life lost. UI=uncertainty interval.

Lower back pain and depressive disorders were ranked the top two causes of YLDs in 1990, 2010, and 2019 (table 4). Between 1990 and 2019, the age-standardised rates of YLDs increased substantially for falls, drug use disorders, diabetes, osteoarthritis, eating disorders, alcohol use disorders, and other musculoskeletal disorders; these conditions were among the leading causes of YLDs in 2019. From 1990 to 2019, YLDs decreased substantially for lower back pain, exposure to mechanical forces, age-related and other hearing loss, asthma, chronic obstructive pulmonary disease, psoriasis, and other unintentional injuries.

**Table 4:** Age-standardised rate (95% UI) of YLDs with percentage changes between 1990 and 2010, 2010 and 2019, and 1990 and 2019 for the leading causes of diseases, disabilities, and injuries in Australia

	**YLD rank**			**YLDs per 100 000 population (95% UI)**			**Age-standardised percentage change in YLDs (95% UI)**		
	1990	2010	2019	1990	2010	2019	1990–2010	2010–19	1990–2019
Lower back pain	1	1	1	1115·3 (784·1 to 1484·7)	1080·5 (763·6 to 1443·3)	986·6 (685·3 to 1334·1)	−3·1% (−6·7 to 0·6)	−8·7% (−14·8 to −2·8)	−11·5% (−17·5 to −5·6)
Depressive disorders	2	2	2	799·5 (550·6 to 1084·7)	861·6 (586·2 to 1182·1)	798·7 (549·4 to 1128·5)	7·8% (0·1 to 15·9)	−7·3% (−15·9 to 2·3)	−0·1% (−7·9 to 7·8)
Other musculoskeletal disorders	5	3	3	525·7 (356·8 to 738·5)	622·8 (426·9 to 862·1)	639·5 (427·9 to 891·1)	18·5% (10·3 to 26·7)	2·7% (−6·9 to 12·2)	21·6% (10·9 to 32·4)
Falls	6	6	4	464·5 (315·3 to 662·1)	521·9 (359·5 to 737·5)	564·8 (390·6 to 799·8)	12·4% (9·8 to 14·9)	8·2% (6·6 to 9·8)	21·6% (19·2 to 24·2)
Anxiety disorders	4	5	5	548·4 (362·0 to 775·8)	536·7 (381·0 to 724·2)	555·3 (366·7 to 785·0)	−2·1% (−18·7 to 19·1)	3·5% (−13·2 to 23·5)	1·3% (−5·4 to 9·1)
Headache disorders	3	4	6	550·3 (122·6 to 1188·8)	551·6 (123·3 to 1192·6)	551·5 (122·4 to 1178·5)	0·2% (−3·0 to 3·3)	0·0% (−2·7 to 3·1)	0·2% (−3·0 to 3·5)
Exposure to mechanical forces	8	7	7	445·7 (291·7 to 671·0)	407·9 (265·1 to 616·0)	419·4 (274·8 to 638·3)	−8·5% (−9·9 to −6·8)	2·8% (1·4 to 4·2)	−5·9% (−7·7 to −4·2)
Drug-use disorders	12	8	8	303·1 (209·7 to 409·4)	359·1 (251·9 to 476·2)	411·5 (288·3 to 547·0)	18·5% (2·6 to 37·5)	14·6% (1·8 to 29·3)	35·8% (19·9 to 55·6)
Age-related and other hearing loss	10	9	9	382·3 (265·9 to 542·8)	355·5 (245·1 to 511·4)	355·2 (240·5 to 508·3)	−7·0% (−10·8 to −3·3)	−0·1% (−5·0 to 5·4)	−7·1% (−12·4 to −1·7)
Asthma	7	10	10	459·3 (305·7 to 657·3)	334·5 (217·1 to 491·3)	344·3 (217·4 to 519·0)	−27·2% (−36·6 to −17·7)	2·9% (−11·1 to 16·4)	−25·0% (−37·8 to −8·9)
Gynaecological diseases	11	11	11	332·3 (229·5 to 465·8)	331·1 (227·3 to 466·1)	327·9 (226·2 to 463·5)	−0·4% (−3·5 to 3·2)	−0·9% (−4·2 to 2·2)	−1·3% (−4·6 to 2·1)
Diabetes	19	13	12	198·7 (133·5 to 275·5)	302·2 (202·3 to 426·4)	326·8 (216·3 to 460·7)	52·1% (36·9 to 69·2)	8·2% (−0·4 to 18·3)	64·5% (47·6 to 85·2)
Oral disorders	9	12	13	423·2 (272·3 to 618·0)	308·0 (192·7 to 461·7)	315·3 (193·6 to 482·2)	−27·2% (−32·2 to −21·5)	2·4% (−9·2 to 15·6)	−25·5% (−35·4 to −14·7)
Osteoarthritis	13	14	14	283·0 (143·0 to 565·8)	297·7 (152·2 to 590·2)	312·9 (158·4 to 634·0)	5·2% (−0·9 to 10·9)	5·1% (−0·1 to 11·3)	10·6% (6·9 to 14·5)
Schizophrenia	14	15	15	247·5 (186·9 to 298·1)	247·2 (186·8 to 300·3)	247·2 (187·5 to 300·3)	−0·1% (−7·1 to 7·0)	0·0% (−5·7 to 6·3)	−0·1% (−6·3 to 6·0)
Bipolar disorder	15	16	16	239·8 (145·4 to 363·5)	241·8 (147·5 to 370·1)	242·1 (146·1 to 370·2)	0·8% (−5·6 to 7·5)	0·1% (−4·2 to 4·6)	1·0% (−5·5 to 7·9)
Eating disorders	22	18	17	151·5 (93·5 to 228·8)	209·6 (136·6 to 295·8)	217·7 (142·6 to 307·4)	38·3% (17·0 to 67·5)	3·9% (−3·6 to 11·5)	43·7% (22·2 to 71·8)
Chronic obstructive pulmonary disease	16	17	18	225·3 (178·3 to 263·7)	222·6 (174·3 to 270·1)	201·1 (160·5 to 236·1)	−1·2% (−12·7 to 14·0)	−9·7% (−22·2 to 2·0)	−10·7% (−15·2 to −6·3)
Endocrine, metabolic, blood, and immune disorders	18	19	19	202·5 (134·6 to 283·2)	201·3 (136·3 to 283·8)	200·3 (135·1 to 281·3)	−0·6% (−5·6 to 4·4)	−0·5% (−5·6 to 4·5)	−1·1% (−5·8 to 3·7)
Other unintentional injuries	17	20	20	212·5 (135·4 to 317·2)	187·2 (119·4 to 280·3)	193·8 (123·5 to 290·1)	−11·9% (−13·8 to −9·7)	3·5% (1·5 to 5·4)	−8·8% (−10·6 to −6·8)
Alcohol use disorders	21	21	21	162·5 (107·5 to 233·6)	171·5 (113·2 to 241·5)	183·9 (121·5 to 259·4)	5·5% (−5·6 to 17·1)	7·2% (−2·5 to 17·9)	13·2% (1·4 to 26·2)
Neonatal disorders	20	22	22	179·6 (134·5 to 235·3)	163·0 (122·7 to 213·2)	158·1 (121·1 to 199·7)	−9·2% (−24·8 to 10·2)	−3·0% (−19·7 to 16·9)	−12·0% (−27·4 to 7·7)
Other mental disorders	25	23	23	140·9 (93·4 to 201·2)	140·9 (93·3 to 200·5)	140·7 (93·9 to 200·1)	0·0% (−4·4 to 4·5)	−0·2% (−4·3 to 4·3)	−0·2% (−4·2 to 4·2)
Psoriasis	23	24	24	145·4 (103·1 to 191·9)	139·7 (99·8 to 185·3)	127·0 (90·1 to 167·3)	−3·9% (−9·0 to 1·8)	−9·1% (−14·4 to −3·9)	−12·6% (−17·5 to −7·4)
Dermatitis	26	25	25	122·7 (68·1 to 205·4)	123·5 (69·1 to 202·0)	122·6 (68·3 to 201·8)	−0·7% (−5·8 to 5·2)	0·7% (−4·8 to 7·2)	0·0% (−5·4 to 5·8)

Note that causes are ordered by their ranking in 2019. YLDs=years of life lived with disability. UI=uncertainty interval.

**Figure 1:**
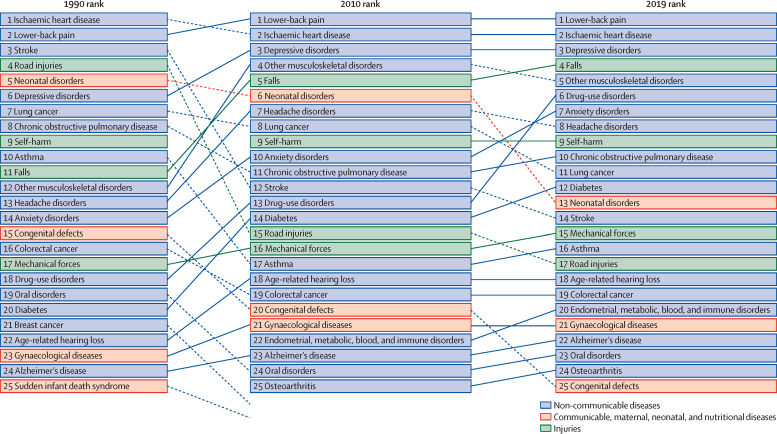
Changes in the ranking of age-standardised rate of disability-adjusted life-years between 1990 and 2010 and 2010 and 2019 for the leading causes of diseases, disabilities, and injuries in Australia

Lower back pain, ranked second in 1990, was ranked first in 2010 and remained the top-ranked cause of age-standardised DALYs in Australia in 2019 (figure 1). The top five leading causes of DALYs in 2010 remained in the top five in 2019 (details of changes in DALYs during 1990–2010, 2010–19, and 1990–2019 are presented in the appendix p 44). Between 1990 and 2019, the rate of age-standardised DALYs decreased by more than 50% for ischaemic heart disease (71·1%, 69·9–72·5), stroke (58·0%, 55·3–60·7), and road injuries (61·7%, 59·3–64·0). We observed notable changes in DALYs ranking for road injuries (from fourth in 1990 to 15th in 2010 and to 17th in 2019), stroke (from third in 1990 to 12th in 2010 and to 14th in 2019), and neonatal disorders (from fifth in 1990 to sixth in 2010 and to 13th in 2019). Between 1990 and 2019, the age-standardised number of DALYs increased for six causes: falls; drug use disorders; diabetes; endocrine, metabolic, blood, and immune disorders; osteoarthritis; and other musculoskeletal disorders. Among the leading causes of DALYs between 1990 and 2019, the largest increase in age-standardised rate was observed for drug use disorders (37·4%, 24·7–52·9) followed by falls (20·2%, 17·8–22·8).

**Figure 2:**
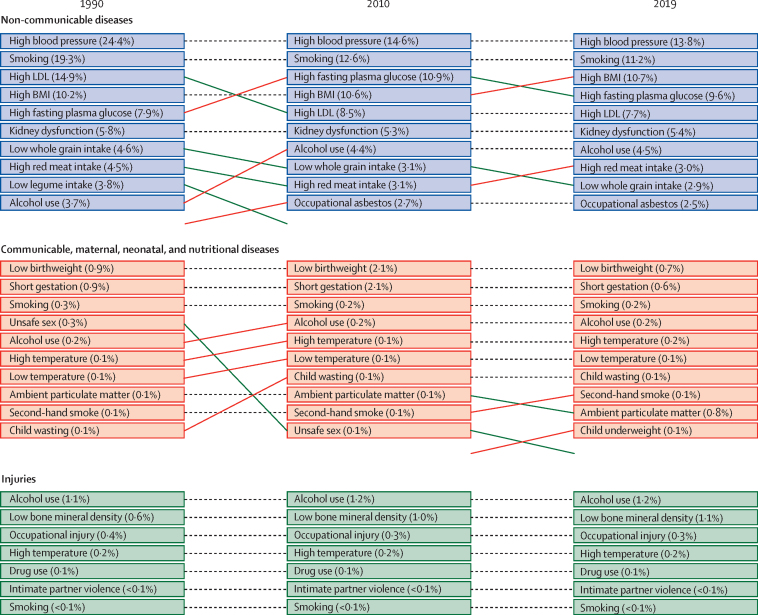
Leading level-4 risk factors for deaths associated with non-communicable and communicable diseases and injuries in Australia in 1990, 2010, and 2019 Values in parentheses indicate the percent contribution of the risk factor towards total deaths. Dotted lines indicate no change in the relative ranking between the years. Red lines indicate an increase in the relative ranking and green lines indicate a decrease in the relative ranking. Rankings are based on the percent contribution of the risk factor towards total deaths. Values in parentheses indicate the percent contribution of the risk factor towards total DALYs. Dotted lines indicate no change in the relative ranking between the years. Red lines indicate an increase in the relative ranking and green lines indicate a decrease in the relative ranking. Rankings are based on the percent contribution of the risk factor towards total DALYs. DALYs=disability-adjusted life-years.

The leading risk factors for deaths and DALYs in 1990, 2010, and 2019 are illustrated (figure 2). High systolic blood pressure, high body-mass index, and smoking were consistently ranked the leading risk factors associated with non-communicable disease-related deaths and DALYs in Australia between 1990 and 2019 and contributed to more than half of the total deaths in Australia. Low birthweight and short gestation were the top-ranked risk factors for deaths and DALYs related to communicable, maternal, neonatal, and nutritional diseases between 1990 and 2019, whereas alcohol use was ranked the number one risk factor for injury-related deaths and DALYs between 1990 and 2019. In 2019, diets high in red meat and low in whole grains were among the top ten leading risk factors for deaths (with diets high in red meat contributing 3·0% and those low in whole grains contributing 2·9% of the total deaths). Diets high in red meat (1·4%) and low in whole grains (1·1%) were also among the top ten leading risk factors for DALYs in 2019.

Compared with 14 other high-SDI countries, Australia's ranking improved slightly in terms of age-standardised rate of deaths (from fifth in 1990 to fourth in 2019) and life expectancy at birth (from sixth in 1990 to fifth in 2019). The relative ranking of HALE at birth in Australia, however, declined from eighth in 1990 to tenth in 2019. In both 1990 and 2019, Australia's ranking was sixth for age-standardised rate of YLLs and 13th for age-standardised YLDs (appendix p 41). Although most of the high-SDI countries displayed a similar pattern of causes of age-standardised DALYs between 1990 and 2019, Australia's ranking was higher than most comparator countries for tracheal, bronchus, and lung cancer, stroke, and road injuries in 2019. Australia's rankings for drug-use disorders, depressive disorders, self-harm, and exposure to mechanical forces, however, were lower than most comparator countries in 2019 (appendix p 46).

## Discussion

Overall, life expectancy at birth increased and age-standardised mortality rates declined between 1990 and 2019 in Australia. There was also a general decrease in age-standardised rates for YLLs and DALYs, with rates for YLDs remaining almost constant during this period. Several diseases, risk factors, and injuries increased between 1990 and 2019. Our findings are consistent with the ABDS 2022 study^12^ which reports that DALYs decreased by 11% and YLLs decreased by 23%, and YLDs increased by 2·1% between 2003 and 2022. These findings might be caused by the epidemiological transition associated with population ageing and the fact that as people are living longer, diseases prevalent in older age, such as Alzheimer's disease and dementia, are contributing more to the rate of YLDs than before.^27^ Continual increases in life expectancy can have important social and economic consequences. Key challenges for Australia will be to keep an ageing population healthy, provide adequate resources to promote healthy lifestyles, and manage non-communicable diseases.

Non-communicable disease-related mortality and YLLs declined substantially over the past three decades, which is consistent with the ABDS 2022 report.^12^ However, non-communicable diseases remain a substantial cause of mortality contributing to 90·8% of all deaths in 2019 and should remain a key target of health-care policy and practice. Additionally, trends for falls, drug-use disorders, and liver cancer have shown increases that are concerning and warrant further investigation. In 2019, ischaemic heart disease was the leading cause of YLLs despite the reduction in age-standardised rates. Ten of the 25 top causes of YLLs were cancers in 2019. The ABDS 2022 reported five non-communicable diseases causing the highest disease burden including cancer, musculoskeletal conditions, cardiovascular diseases, mental health conditions and substance use disorders, and neurological conditions; collectively, these non-communicable diseases accounted for 62% of the total disease burden in 2022.^12^

The steep declines in DALYs observed for road injuries and neonatal disorders between 1990 and 2019 might reflect firm policies and practices focused on these areas including public campaigns to promote safer driving, increased road and vehicle safety, legislation and fines for driving offences, and improved maternal health services and pregnancy care. However, large increases in rankings were seen for falls, drug use, and anxiety disorders. Lower back pain was the leading cause of DALYs in 2019. Other areas of concern in 2019 include ischaemic heart disease (ranked second), depressive disorders (ranked third), anxiety disorders (ranked seventh), self-harm behaviour (ranked nineth), and diabetes (ranked 12th); the rankings of these conditions have all increased relative to 1990. The ABDS 2022 described coronary heart disease as the leading cause of DALYs in 2022 followed by dementia, back pain, chronic obstructive pulmonary disease, and anxiety disorders.^12^ Non-communicable diseases were the leading cause of YLDs. Age-standardised rates for non-communicable diseases decreased, but the total number of cases increased, putting pressure on health-care systems in a manner consistent with reports from other OECD countries.^28^ The observed increases in the rankings for falls, drug use, anxiety disorders, and self-harm in Australia could be attributed to a number of factors. Falls might be on the rise because of an ageing population that is more susceptible to falls and related injuries.^29^ Additionally, changes in lifestyle and physical activity (eg, sedentary jobs and increased use of technology) and biological changes associated with ageing such as impaired gait, muscle weakness, and impaired vision might contribute to a higher incidence of falls.^30^ Regarding drug use, it is possible that increased availability and accessibility of drugs in Australia and socioeconomic factors including poverty, unemployment, anxiety, and homelessness could lead to higher rates of drug use.^31^ Increased self-harm might be related to underlying mental health conditions, trauma, bullying, and feelings of hopelessness and despair.^32^ Finally, the rise in anxiety disorders could be attributed to increased awareness and diagnosis of anxiety disorders, heightened societal pressures and expectations, and social isolation and loneliness.^33^ To address these issues, it is important to invest in preventive measures such as increasing access to health care, mental health services, and resources supporting physical activity and healthy lifestyles.^34^ Additionally, efforts are needed to address underlying social and economic factors that might contribute to these issues, such as poverty and social isolation, and raising awareness and promoting education on the risks and consequences of drug use and self-harm.^32^

Cardiometabolic and behavioural risk factors, such as high blood pressure, smoking, high fasting plasma glucose, and high body-mass index were the leading risk factors, accounting for more than half of all deaths in 2019. Smoking was the second leading risk factor contributing to deaths and DALYs for both sexes combined in Australia in 2019, which is in line with global data.^35^ Smoking prevalence is relatively high among certain groups of people, such as Indigenous populations (40·1%) and people from the least disadvantage groups (29·9%), and people living in regional and remote settings (19%).^36^, ^37^ For many years, Australia has been a world leader in tobacco control, with strong policy initiatives including smoking bans in public and workplace settings, increased taxes, government support for nicotine replacement, plain packaging, graphic warnings on packets, and advertising restrictions.^38^ Although such initiatives have seen decreases in smoking rates (from 24·3% in 1991 to 11·6% in 2019),^39^ the results of the present study indicate that smoking continues to pose a major health risk to the Australian population. A previous study showed that smoking contributed to 8·6% of the total burden of disease and injury in Australia and cancers accounted for 44% of disease burden.^40^ Approaches using a combination of smoking cessation tools^41^ with pharmacotherapy and behavioural intervention must be considered.

The burden of disease in Australia is strongly influenced by broader factors and systems including individual, social, lived, environmental, and food environments.^42^ A systematic review of 28 studies revealed that improving neighbourhood walkability, parks and playgrounds, and active transport infrastructure has a positive impact on physical activity and active transport.^43^ However, these interventions might mainly benefit socioeconomically advantaged groups, and the studies were limited by selection bias and insufficient control of confounding factors. Our findings related to dietary factors, such as consumption of diets high in red meat, low whole grain intake, and low fruit intake are in line with a GBD study.^44^ Fruit and whole grains are recommended foods in the Australian Dietary Guidelines; however, existing national data demonstrate that intake of these foods is lower than recommended and overall diet quality is poor.^9^ Such findings highlight the importance of greater national efforts for preventive action, including the promotion of healthy and sustainable diets, and advocacy from leading public health and nutrition organisations for new national nutrition policies and implementation plans (see Nourishing Australia and Call for a New National Nutrition Policy). A meta-ethnography systematic review found that various environmental and social factors were commonly reported as obstacles to healthy eating, with affordability of healthy food and limited availability of healthy food stores being particularly substantial barriers for individuals from lower socioeconomic backgrounds.^42^ These findings suggest that although barriers to healthy eating are generally applicable, certain factors might disproportionately affect vulnerable populations. In addition, commercial determinants of health including industry sectors such as tobacco, ultraprocessed food, fossil fuel, and alcohol account for most of the non-communicable disease-related deaths and disabilities.^45^ Australia has a wide availability of parks, gyms, and bicycle paths to facilitate physical activity and heath star ratings for diets and professional associations and media advocacy which leads to increased consumer awareness.^46^, ^47^, ^48^

Compared with other high-SDI countries, Australia's ranking improved in terms of age-standardised deaths and life expectancy at birth, although YLLs and YLDs remained unchanged in terms of their ranking between 1990 and 2019. Australia's ranking declined for HALE from eighth to tenth between 1990 and 2019. Although most of the high-SDI countries share a similar profile for age-standardised causes of DALYs between 1990 and 2019, Australia's ranking is better for diabetes and stroke than most comparator countries. That both conditions were included in the 2016 National Health Priority Area initiative is noteworthy. However, Australia's rankings for anxiety disorders, depressive disorders, and falls are worse than most comparator countries and are areas of concern. These findings suggest that primary prevention and health promotion services may have been insufficient to tackle these issues. Therefore, there is a need for strategies targeting mental health and the prevention of falls in Australia.

A key to sustaining health improvements in Australia is the universal health coverage system (Medicare) that combines free access to primary care and public hospital services and subsidised access to specialist services and pharmaceuticals, with higher subsidies to those with lower incomes. Through robust health systems including strong regulatory capacities for medicines, medical products, vaccines, the health workforce, health services, information systems, and the quality and safety of health care, Australia's health performance remains one of the best globally.^3^, ^4^ In 2018–19, health expenditure in Australia was estimated at $195·7 billion, or 10% of the gross domestic product (GDP), which was slightly higher than the OECD average of 9·3%, ranking Australia tenth out of the 22 countries.^49^ Almost 68% of the health expenditure in Australia is funded by governments, 16·3% through out-of-pocket expenses, 8·9% by private health insurers, and 6·7% through accident compensation schemes.^49^ The per-person health expenditure in Australia (AUS$7772) was higher than the OECD average (AUS$4561), placing Australia nineth out of 22 countries.^49^ However, spending on prevention was only 1·34% of all health expenditure in Australia compared with an average health spending on prevention activities of 2·8% in OECD countries.^50^ In 2015, Canada and the USA had the highest per-capita prevention expenditure at more than US$250, about two-and-a-half times the OECD average, whereas Australia spent just below the average of US$116 per capita and Greece and Latvia had the lowest spending.^50^ Australia spends only about 0·13% of GDP on prevention, presenting a strong case for increasing investment in preventive health in Australia.^51^

This study is subject to the limitations of the GBD methodology, which have been previously described.^22^, ^24^ Our report should be interpreted with the following limitations. First, despite comprehensive vital registration data, information on morbidity and health outcomes associated with behavioural and metabolic risk factors are comparatively limited in Australia, and data on inpatient and outpatient hospital admissions, health system access, and health financing were not available for the GBD 2019 study. Second, we could not present state-level and territory-level analyses for Australia because of the absence of such data in the GBD analytical tools. Because of the large geographical, demographic, ethnic, and cultural diversity of its population, and the state-level authority in the health system, subnational estimation of health and behavioural patterns should be a key future priority for Australia. Third, stratifying results by populations associated with disadvantages will be beneficial in terms of understanding disparities and directing appropriate resources to those most in need. Fourth, we used the GBD analytical tools and WHO Standard Population to determine rankings for disease burden. Using the Australian life table might have influenced the ranking of diseases. However, the GBD tools are robust and facilitate the comparison of our results with other countries. Fifth, although the 14 comparator countries have SDI values similar to Australia, it is beyond the scope of this report to describe the extent to which the observed differences between countries were influenced by social, environmental, political, or other factors. Sixth, we were not able to report gender-specific and age-specific differences in life expectancy and cause-specific mortality in this report but plan to make this the focus of future research. Seventh, health behaviour assessment methods are subject to biases, such as social desirability, leading to over-reporting of healthy behaviours and under-reporting of unhealthy behaviours.^52^ However, improved dietary and physical activity assessment methods that minimise these reporting issues are available and could be used in future research to overcome these limitations.^44^ Finally, we were unable to estimate the effects of the COVID-19 pandemic on disease burden in Australia because the GBD 2019 estimates used in the present study do not cover the time period associated with this pandemic.

The COVID-19 pandemic has had a substantial impact on global health and disease burden. Previous studies conducted during this pandemic have highlighted its global socioeconomic impacts, including food insecurity and barriers to health-care access, such as decreased disease screening, prevention activities, and routine health monitoring.^53^, ^54^, ^55^, ^56^ Heightened surveillance and treatment of new cases linked to pandemic-related disruptions will be necessary to maintain progress in reducing disease prevalence and avoid widening disparities in disease burden.^55^, ^57^ Future analyses should focus on the direct and indirect effects of the COVID-19 pandemic; although the pandemic threatened health-care access, it also fuelled innovation in the provisioning of health care and catalysed an expansion of telemedicine.^58^, ^59^ In addition, analyses examining the effects of vaccination and long-term effects of COVID-19 on health are needed. The post-COVID-19 era might observe a surge in the incidence of certain diseases because of diagnostic practices returning to normal. Subsequent analyses could assess the trends of diseases affected by different stages of the COVID-19 pandemic.^55^

In conclusion, Australia has increased life expectancy at birth by 6 years since 1990, primarily through reductions in communicable, maternal, neonatal, and nutritional diseases. Addressing non-communicable diseases and related risk factors is still a major concern. Further preventive and supportive treatment approaches for drug-use disorder, anxiety and mental health, and falls are required. Better health-care services and rehabilitation for the ageing population and targeted approaches for addressing several risk factors at the population level are needed. This study provides a better understanding of the crucial risk factors and opportunities for improvement. Given Australia's geographical spread and socioeconomic inequities, diverse patterns of health and disease burden will probably continue to be identified across the country. Subnational estimates of the burden of disease will be particularly valuable in tailoring health priorities and programmes to the needs of specific regions. Findings will help to identify gaps in service and develop a national level response to guide government policies, programmes, and services to improve the health and wellbeing of the Australian population. Monitoring disease prevalence and trends over time will also allow policy makers to set priorities, track changes, and assess health transitions relative to similar countries.

## Data sharing

Data used in the analyses of this manuscript is available publicly to download. Please visit the Global Health Data Exchange GBD 2019 website at https://ghdx.healthdata.org/gbd-2019.

Correspondence to: Dr Sheikh Mohammed Shariful Islam, Institute for Physical Activity and Nutrition, School of Exercise and Nutrition Sciences, Deakin University, Geelong, VIC 3125, Australia

**shariful.islam@deakin.edu.au**

## Declaration of interests

SMSI reports support for the present manuscript from a National Health and Medical Research Council (NHMRC) Investigator Grant and from a National Heart Foundation Vanguard Grant. BA reports grants or contracts form Rebecca Cooper Foundation for Investigator-initiated trial grant and a Nat Rem Grant for Investigator-initiated trial biomarkers assessment support, and payment or honoraria for lectures, presentations, speakers bureaus, manuscript writing or educational events from Nat Rem and IRACON, all outside the submitted work. PA reports support for the present manuscript from the Australian College of Applied Professions Discipline of Psychological Sciences, Sydney, Australia. BTB reports consulting fees from AstraZeneca, Bristol-Myers Squibb, Janssen, LivaNova, Lundbeck, Novartis, Otsuka, Pfizer, Servier, Wyeth, Biogen, Angelini, Sumitomo Pharma, and Boehringer-Ingelheim, and payment or honoraria for lectures, presentations, speakers bureaus, manuscript writing or educational events from AstraZeneca, Bristol-Myers Squibb, Janssen, LivaNova, Lundbeck, Novartis, Otsuka, Pfizer, Servier, Wyeth, Biogen, Angelini, Sumitomo Pharma, and Boehringer-Ingelheim, all outside the submitted work. AMB reports grants from Australian National Health and Medical Research Council, Bone and Joint Decade Foundation, Arthritis and Osteoporosis Western Australia, Department of Health Government of Western Australia through payments to their institution, consulting fees from WHO, payment or honoraria for lectures, presentations, speakers bureaus, manuscript writing or educational events from American College of Rheumatology, and support for attending meetings or travel from WHO, all outside the submitted work. RB reports grants from the NHMRC, Medical Research Future Fund, Arthritis Australia, HCF Research Foundation, Cabrini Foundation, and Australian Government Department of Health, all as payments to their institution, and royalties from UpToDate for Plantar fasciitis, all outside the submitted work. SRC reports grants or contracts from the Wellcome Trust, Australian Department of Veterans Affairs, National Institutes of Mental Health, Janssen-Cilag Australia, Australian Defense Science and Technology Group, and the Australian National Health and Medical Research Council, consulting fees from Lundbeck-Otsuka Australia, payment or honoraria for lectures, presentations, speakers bureaus, manuscript writing or educational events from Lundbeck-Otsuka Australia, support for attending meetings or travel from Janssen-Cilag Australia, participation on a Data Safety Monitoring Board or Advisory Board with Verbatim Trial Orygen Australia, and leadership or fiduciary role in other board, society, committee or advocacy group, unpaid, with executive Committee Member Society for Mental Health Research, all outside the submitted work. KML reports support for the present manuscript from an NHMRC Investigator Grant (APP1173803). PBM reports payment or honoraria for lectures, presentations, speakers bureaus, manuscript writing or educational events from Janssen (Australia), and Sanofi (Hangzhou), and participation on a Data Safety Monitoring Board or Advisory Board with Janssen (Australia), all outside the submitted work. AEP reports support for the present manuscript from the NHMRC as a grant paid to their institute. JS reports grants or contracts from the National Health and Medical Research Council, the Department of Education, Victoria, and the Medical Research Future Fund, and leadership or fiduciary role in other board, society, committee or advocacy group, unpaid, as President of the Asia Pacific Society for Physical Activity, a not-for-profit professional society. AES reports grants or contracts from the National Health and Medical Research Council of Australia, Leadership Investigator Grant ID2017504, personal payment or honoraria for lectures, presentations, speakers bureaus, manuscript writing or educational events from Servier, Novartis, Omron, Aktiia, and Medtronic, support for attending meetings or travel from Servier, participation on Abbot Advisory Board, and leadership or fiduciary role in other board, society, committee or advocacy group, paid or unpaid, as Secretary of Australian Cardiovascular Alliance and co-Chair of National Hypertension Taskforce of Australia, all outside the submitted work. HS reports grants to their institute from the Australian Government, Department of Health, Medical Research Future Fund, Western Australian Government Department of Health, Bone and Joint Decade Foundation (Sweden) Curtin University (Australia), Institute for Bone and Joint Research (Australia), and Canadian Memorial Chiropractic College (Canada), all outside the submitted work. SS reports payment or honoraria for lectures, presentations, speakers bureaus, manuscript writing or educational events for an online lecture on Cultural consideration for pain care delivered on Jan 26, 2023, and travel accommodation for delivering a talk on Technologies for pain education in developing countries conducted by the Pain Education SIG of the International Association for the Study of Pain, all outside the submitted work. MAS reports leadership or fiduciary role in other board, society, committee or advocacy group, unpaid, as Vice President of Kidsafe Victoria, Director Council of the Aged Victoria, and Director Australasian Society of Autism Research, outside the submitted work. JS reports stock or stock options in Anagram kommunikation, MinForskning, and Symptoms Europe, outside the submitted work. AGT reports grants or contracts from National Health and Medical Research Council (Australia; grant numbers 1143155, 1171966, and 1182071), and the Medical Research Future Fund (Australian Government; grant number 2015976), as payments to their institution outside the submitted work. MW reports consulting fees from Amgen and Freeline, participation on a Data Safety Monitoring Board or Advisory Board with Staree DSMB, all outside the submitted work. All other authors declare no competing interests.
