# Correction to *Lancet Public Health* 2023; 8: e585–99

**DOI:** 10.1016/S2468-2667(23)00184-6

**Published:** 2023-08-09

*GBD 2019 Australia Collaborators. The burden and trend of diseases and their risk factors in Australia, 1990–2019: a systematic analysis for the Global Burden of Disease Study 2019.* Lancet Public Health *2023;* 8: *e585–99*—In this Article, the members of the GBD 2019 Australia Collaborators are now listed on PubMed. This correction has been made to the online version as of Aug 9, 2023.

