# Correction to *Lancet Public Health* 2023; 8: e585–99

**DOI:** 10.1016/S2468-2667(23)00251-7

**Published:** 2023-11-22

*GBD 2019 Australia Collaborators. The burden and trend of diseases and their risk factors in Australia, 1990–2019: a systematic analysis for the Global Burden of Disease Study 2019.* Lancet Public Health *2023; **8:** e585–99*—In this Article, the third sentence of the fourth paragraph of the Discussion should have read “Smoking prevalence is relatively high among certain groups of people, such as Indigenous populations (40·1%) and people from the most disadvantaged groups (21·7%), and people living in regional and remote settings (19%)”. The appendix has also been updated to reflect the correct author order, with Riaz Uddin being the second author. These corrections have been made as of Nov 22, 2023.

