# Supplementary material for: The burden and trend of diseases and their risk factors in Australia, 1990–2019: a systematic analysis for the Global Burden of Disease Study 2019
Source: Lancet Public Health. 2023 Jul 27;8(8):e585–99. doi: 10.1016/S2468-2667(23)00123-8 (PMC10400798; doi:10.1016/S2468-2667(23)00123-8)
Supplement: Supplementary appendix [file mmc1.pdf]

THE LANCET
Public Health

Supplementary appendix

This appendix formed part of the original submission and has been peer reviewed. We post it as supplied by the authors.

Supplement to: GBD 2019 Australia Collaborators. The burden and trend of diseases and their risk factors in Australia, 1990–2019: a systematic analysis for the Global Burden of Disease Study 2019. *Lancet Public Health* 2023; **8:** e585–99.

The burden and trend of diseases and their risk factors in Australia, 1990–2019: a systematic analysis for the Global Burden of Disease Study 2019

GBD 2019 Australia Collaborators

Sheikh Mohammed Shariful Islam, Ralph Maddison, Riaz Uddin, Kylie Ball, Katherine M Livingstone, Asaduzzaman Khan, Jo Salmon, Ilana N Ackerman, Tim Adair, Oyelola A Adegboye, Zanfina Ademi, Ripon Kumar Adhikary, Bright Opoku Ahinkorah, Khurshid Alam, Kefyalew Addis Alene, Sheikh Mohammad Alif, Azmeraw T Amare, Edward Kwabena Ameyaw, Leopold N Aminde, Deanna Anderlini, Blake Angell, Adnan Ansar, Benny Antony, Anayochukwu Edward Anyasodor, Victoria Kiriaki Arnet, Thomas Astell-Burt, Prince Atorkey, Mamaru Ayenew Awoke, Beatriz Paulina Ayala Quintanilla, Getinet Ayano, Nasser Bagheri, Anthony Barnett, Bernhard T Baune, Dinesh Bhandari, Sonu Bhaskar, Raaj Kishore Biswas, Rohan Borschmann, Soufiane Boufous, Andrew M Briggs, Rachelle Buchbinder, Norma B Bulamu, Richard A Burns, Andre F Carvalho, Ester Cerin, Nicolas Cherbuin, Enayet Karim Chowdhury, Liliana G Ciobanu, Scott Richard Clark, Marita Cross, Abel Fekadu Dadi, Barbora de Courten, Diego De Leo, Katie de Luca, Kerrie E Doyle, David Edvardsson, Kristina Edvardsson, Ferry Efendi, Aklilu Endalamaw, Nelsensius Klau Fauk, Xiaoqi Feng, Bernadette Mary Fitzgibbon, Joanne Flavel, Eyob Alemayehu Alemayehu Gebreyohannes, Hailay Abrha Gesesew, Tiffany K Gill, Myron Anthony Godinho, Bhawna Gupta, Vivek Kumar Gupta, Mitiku Teshome Hambisa, Mohammad Hamiduzzaman, Graeme J Hankey, Hossein Hassanian-Moghaddam, Simon I Hay, Jeffrey J Hebert, M Mamun Huda, Tanvir M Huda, M Mofizul Islam, Mohammad Saidul Islam, Rakibul M Islam, Billingsley Kaambwa, Himal Kandel, Gizat M Kassie, Jaimon Terence Kelly, Jessica A Kerr, Girmay Tsegay Kiross, Luke D Knibbs, Vishnutheertha Vishnutheertha Kulkarni, Ratilal Lalloo, Long Khanh Dao Le, James Leigh, Janni Leung, Shanshan Li, Rashidul Alam Mahumud, Abdullah A Mamun, Melvin Barrientos Marzan, John J McGrath, Max L Mehlman, Atte Meretoja, Amanual Getnet Mersha, Ted R Miller, Philip B Mitchell, Ali H Mokdad, Lidia Morawska, Christine Mpundu-Kaambwa, William Mude, Christopher J L Murray, Sandhya Neupane Kandel, Tafadzwa Nyanhanda, Kehinde O Obamiro, Amy E Peden, Konrad Pesudovs, Kevan R Polkinghorne, Azizur Rahman, Muhammad Aziz Rahman, Zubair Ahmed Ratan, Lal Rawal, Lennart Reifels, Andre M N Renzaho, Stephen R Robinson, Danial Roshandel, Susan Fred Rumisha, Paul A Saunders, Susan M Sawyer, Markus P Schlaich, Aletta Elisabeth Schutte, Abdul-Aziz Seidu, Saurab Sharma, Seyed Afshin Shorofi, Soraya Siabani, Ambrish Singh, Balbir Bagicha Singh, Helen Slater, Jacqueline H Stephens, Mark A Stokes, Narayan Subedi Subedi, Chandra Datta Sumi, Jing Sun, Johan Sundström, Cassandra E I Szoeke, Santosh Kumar Tadakamadla, Ken Takahashi, Jo Taylor, Melkamu B Tessema Tessema, Amanda G Thrift, Quyen G To, Daniel Nigusse Tollosa, Mai Thi Ngoc Tran, Corneel Vandelanotte, Blesson Mathew Varghese, Lennert J Veerman, Ning Wang, Paul Ward, Mark Woodward, Befikadu Legesse Wubishet, Xiaoyue Xu, Pengpeng Ye, Sojib Bin Zaman, Amin Zarghami, Jianrong Zhang, and David A Crawford.

Affiliations

Institute for Physical Activity and Nutrition (S Islam PhD, Prof R Maddison PhD, R Uddin PhD, Prof K Ball PhD, K M Livingstone PhD, Prof J Salmon PhD), School of Exercise and Nutrition Sciences (N S Subedi MPH, Prof D A Crawford PhD), Deakin University, Melbourne, VIC, Australia; Sydney Medical School (S Islam PhD), Menzies Centre for Health Policy (Prof T Astell-Burt PhD), Faculty of Medicine and Health (M Cross PhD), Chapter of Addiction Medicine (Prof H Hassanian-Moghaddam MD), School of Public Health (T M Huda PhD, L D Knibbs PhD), Save Sight Institute (H Kandel PhD), Asbestos Diseases Research Institute (J Leigh MD), NHMRC Clinical Trials Centre (R A Mahumud PhD), School of Veterinary Science (B B Singh PhD), University of Sydney, Sydney, NSW, Australia (R Biswas MSc); School of Health and Rehabilitation Sciences (R Uddin PhD, A Khan PhD), Centre for Sensorimotor Performance (D Anderlini MD), School of Public Health (A

Endalamaw MSc, Prof L J Veerman PhD), Institute for Social Science Research (M Huda MSc), School of Dentistry (R Lalloo PhD), Queensland Brain Institute (Prof J J McGrath MD), The University of Queensland, Brisbane, QLD, Australia (J T Kelly PhD); School of Public Health and Preventive Medicine (I N Ackerman PhD, Z Ademi PhD, S M Alif PhD, S Li PhD), Department of Epidemiology and Preventive Medicine (Prof R Buchbinder PhD, B M Fitzgibbon PhD, R M Islam PhD), Department of Epidemiology and Preventative Medicine (E K Chowdhury PhD), The School of Clinical Sciences at Monash Health (Prof B de Courten PhD), Department of Medicine (Prof K R Polkinghorne PhD, Prof A G Thrift PhD, S Zaman MSc), Department of Public Health (J Taylor PhD), Monash University, Melbourne, VIC, Australia; Melbourne School of Population and Global Health (T Adair PhD), University of Melbourne, Carlton, VIC, Australia; Public Health and Tropical Medicine (O A Adegboye PhD), College of Public Health, Medical and Veterinary Sciences (A Seidu MPhil), James Cook University, Townsville, QLD, Australia; Department of Fisheries and Marine Bioscience (R K Adhikary MS), Jashore University of Science and Technology, Jashore, Bangladesh; Research School of Population Health (R K Adhikary MS, N Bagheri PhD, R A Burns PhD, Prof N Cherbuin PhD), Australian National University, Canberra, ACT, Australia; School of Public Health (B O Ahinkorah MPhil), School of Health (S Siabani PhD), School of Computing Sciences (Prof J Sun PhD), University of Technology Sydney, Sydney, NSW, Australia; Murdoch Business School (K Alam PhD), Murdoch University, Perth, WA, Australia; Faculty of Health Sciences (K A Alene MPH), School of Public Health (G Ayano MSc, E K Chowdhury PhD, T R Miller PhD), School of Physiotherapy and Exercise Science (Prof A M Briggs PhD, Prof H Slater PhD), Curtin University, Perth, WA, Australia; Wesfarmers Centre of Vaccines and Infectious Diseases (K A Alene MPH), Malaria Atlas Project (S F Rumisha PhD), Telethon Kids Institute, Perth, WA, Australia; School of Medicine (A Amare PhD), Discipline of Psychiatry (V K Arnet BHlthSc, S R Clark PhD), Adelaide Medical School (L G Ciobanu PhD, T K Gill PhD), School of Public Health (B M Varghese PhD), University of Adelaide, Adelaide, SA, Australia; College of Medicine and Health Science (A Amare PhD), Department of Pediatrics and Child Health Nursing (A Endalamaw MSc), Bahir Dar University, Bahir Dar, Ethiopia; School of Graduate Studies (E K Ameyaw MPhil), Lingnan University, Hong Kong, China; School of Medicine (L N Aminde PhD, Prof J Sun PhD, Prof L J Veerman PhD), School of Dentistry and Oral Health (S K Tadakamadla PhD), Griffith University, Gold Coast, QLD, Australia; Neurology Department (D Anderlini MD), Royal Brisbane and Women's Hospital, Brisbane, QLD, Australia; Institute of Global Health (B Angell PhD), University College London, Holbourne, UK; Health System Science Program (B Angell PhD), The George Institute for Global Health, Newtown, NSW, Australia; School of Nursing and Midwifery (A Ansar PhD, Prof D Edvardsson PhD, F Efendi PhD, M Rahman PhD), The Judith Lumley Centre (B Ayala Quintanilla PhD), School of Psychology and Public Health (M Islam PhD), Centre for Alcohol Policy Research (CAPR) (M B Marzan MSc), La Trobe University, Melbourne, VIC, Australia; Special Interest Group International Health (A Ansar PhD), Public Health Association of Australia, Canberra, ACT, Australia; Menzies Institute for Medical Research (B Antony PhD, A Singh Mtech), University of Tasmania, Hobart, TAS, Australia; School of Dentistry and Medical Sciences (A E Anyasodor PhD), Charles Sturt University, Orange, NSW, Australia; School of Health and Society (Prof T Astell-Burt PhD, Z Ratan MSc), University of Wollongong, Wollongong, NSW, Australia; School of Medicine and Public Health (P Atorkey MPhil, M T Hambisa MPH, A G Mersha MD), Newcastle Law School (M S Islam LLM), Department of Public Health and Medicine (D N Tollosa PhD), Research Centre for Generational Health and Ageing (B Wubishet MPH), University of Newcastle, Newcastle, NSW, Australia; Hunter New England Population Health, Wallsend, NSW, Australia (P Atorkey MPhil); Department of Epidemiology and Preventive Medicine (M A Awoke MPH), Justice Health Unit (R Borschmann PhD), School of Health Sciences (A Meretoja MD), Melbourne School of Population and Global Health (L Reifels PhD), Faculty of Medicine, Dentistry and Health Sciences (Prof C E I Szoeke PhD), Department of General Practice (J Zhang MD), University of Melbourne, Melbourne, VIC, Australia; San Martin de Porres University, Lima, Peru (B Ayala Quintanilla PhD); School of Indigenous Studies (G Ayano MSc), Division of

Pharmacy (E A Gebreyohannes MSc), Medical School (Prof G J Hankey MD), Centre for Ophthalmology and Visual Science (D Roshandel MD), Dobney Hypertension Centre (Prof M P Schlaich MD), University of Western Australia, Perth, WA, Australia; Health Research Institute (N Bagheri PhD), University of Canberra, Canberra, ACT, Australia; Mary MacKillop Institute for Health Research (A Barnett PhD, Prof E Cerin PhD), Australian Catholic University, Melbourne, VIC, Australia; Department of Psychiatry (Prof B T Baune PhD), University of Münster, Münster, Germany; Department of Psychiatry (Prof B T Baune PhD), Melbourne Medical School, Melbourne, VIC, Australia; School of Nursing and Midwifery (D Bhandari PhD), Monash University, Clayton, VIC, Australia; Public Health Research Laboratory (D Bhandari PhD), Central Department of Public Health (N S Subedi MPH), Tribhuvan University, Kathmandu, Nepal; Global Health Neurology Lab (S Bhaskar PhD), NSW Brain Clot Bank, Sydney, NSW, Australia; Department of Neurology and Neurophysiology (S Bhaskar PhD), South West Sydney Local Heath District and Liverpool Hospital, Sydney, NSW, Australia; Centre for Adolescent Health (R Borschmann PhD, J A Kerr PhD), Murdoch Childrens Research Institute, Melbourne, VIC, Australia; Transport and Road Safety (TARS) Research Centre (S Boufous PhD), School of Population Health (X Feng PhD, X Xu PhD), School of Psychiatry (Prof P B Mitchell MD), School of Public Health and Community Medicine (A E Peden PhD, Prof A E Schutte PhD), School of Optometry and Vision Science (Prof K Pesudovs PhD), Faculty of Medicine and Health (S Sharma PhD), The George Institute for Global Health (P Ye MPH), University of New South Wales, Sydney, NSW, Australia; Monash Department of Clinical Epidemiology at Cabrini Hospital (Prof R Buchbinder PhD), Cabrini Institute, Melbourne, VIC, Australia; Flinders Health and Medical Research Institute (N B Bulamu PhD), Discipline of Public Health (A F Dadi MPH), College of Medicine and Public Health (H A Gesesew PhD, B Kaambwa PhD), Health Economics Unit (B Kaambwa PhD), Health and Social Care Economics Group (C Mpundu-Kaambwa PhD), Nursing and Health Sciences (S Shorofi PhD), Flinders University, Adelaide, SA, Australia; The Institute for Mental and Physical Health and Clinical Translation Strategic Research Center (A F Carvalho MD), Deakin University, Geelong, VIC, Australia; School of Public Health (Prof E Cerin PhD), University of Hong Kong, Hong Kong, China; School of Pharmacy and Medical Sciences (L G Ciobanu PhD), Quality Use of Medicines and Pharmacy Research Centre (G M Kassie PhD), University of South Australia, Adelaide, SA, Australia; Institute of Public Health (A F Dadi MPH), Department of Clinical Pharmacy (E A Gebreyohannes MSc), School of Medicine (A G Mersha MD), University of Gondar, Gondar, Ethiopia; Australian Institute for Suicide Research and Prevention (Prof D De Leo DSc), Griffith University, Mount Gravatt, QLD, Australia; Discipline of Chiropractic (K de Luca PhD), CQ University, Brisbane, QLD, Australia; School of Medicine (Prof K E Doyle PhD), Western Sydney University, Sydney, NSW, Australia; School of Health and Biomedical Sciences (Prof K E Doyle PhD), Royal Melbourne Institute of Technology University, Melbourne, VIC, Australia; Department of Nursing (Prof D Edvardsson PhD), Umeå University, Umea, Sweden; College of Science, Health and Engineering (K Edvardsson PhD), La Trobe University, Bundoora, VIC, Australia; Community Health Nursing (F Efendi PhD), Universitas Airlangga (Airlangga University), Surabaya, Indonesia; Centre for Health Policy Research (Prof P Ward PhD), Torrens University Australia, Adelaide, SA, Australia (N K Fauk MSc); Institute of Resource Governance and Social Change, Kupang, Indonesia (N K Fauk MSc); National Institute of Environmental Health (X Feng PhD), National Center for Chronic and Noncommunicable Disease Control and Prevention (N Wang PhD, P Ye MPH), Chinese Center for Disease Control and Prevention, Beijing, China; School of Social Sciences (J Flavel PhD), Stretton Health Equity, Adelaide, SA, Australia; Department of Epidemiology (H A Gesesew PhD), School of Pharmacy (B Wubishet MPH), Mekelle University, Mekelle, Ethiopia; School of Public Health and Community Medicine (M A Godinho MBBS), The George Institute (Prof M Woodward PhD), University of New South Wales, Kensington, NSW, Australia; Department of Public Health (B Gupta PhD), Torrens University Australia, Melbourne, VIC, Australia; Faculty of Medicine Health and Human Sciences (Prof V K Gupta PhD), Macquarie University, Sydney, NSW, Australia; School of Public Health (M T Hambisa MPH), Haramaya University, Harar, Ethiopia; Faculty of Health

(M Hamiduzzaman PhD), Southern Cross University, Bilinga, QLD, Australia; Department of Neurology (Prof G J Hankey MD), Sir Charles Gairdner Hospital, Perth, WA, Australia; Social Determinants of Health Research Center (Prof H Hassanian-Moghaddam MD), Shahid Beheshti University of Medical Sciences, Tehran, Iran; Institute for Health Metrics and Evaluation (Prof S I Hay FMedSci, M L Mehlman PhD, A H Mokdad PhD, Prof C J L Murray DPhil), Department of Health Metrics Sciences, School of Medicine (Prof S I Hay FMedSci, A H Mokdad PhD, Prof C J L Murray DPhil), University of Washington, Seattle, WA, USA; Faculty of Kinesiology (Prof J J Hebert PhD), University of New Brunswick, Fredericton, NB, Canada; School of Psychology and Exercise Science (Prof J J Hebert PhD), Murdoch University, Murdoch, WA, Australia; Maternal and Child Health Division (T M Huda PhD, S Zaman MSc), International Centre for Diarrhoeal Disease Research, Bangladesh, Dhaka, Bangladesh; Sydney Eye Hospital (H Kandel PhD), South Eastern Sydney Local Health District, Sydney, NSW, Australia; Department of Psychological Medicine (J A Kerr PhD), University of Otago, Christchurch, New Zealand; Department of Public Health (G T Kiross MPH), Debre Markos University, East Gojjam, Ethiopia; Department of Women's Health (G T Kiross MPH), Newcastle University, Newcastle, NSW, Australia; Department of Medicine (V V Kulkarni MS), Queensland Health, Brisbane, QLD, Australia; Health Economics Division (L K D Le PhD), Monash University, Burwood, VIC, Australia; Center for Youth Substance Abuse Research (J Leung PhD), The University of Queensland, St. Lucia, QLD, Australia; Institute for Social Science Research (A A Mamun PhD), The University of Queensland, Indooroopilly, QLD, Australia; National Centre for Register-based Research (Prof J J McGrath MD), Aarhus University, Aarhus, Denmark; Neurology Unit (A Meretoja MD), Helsinki University Hospital, Helsinki, Finland; Pacific Institute for Research & Evaluation, Calverton, MD, USA (T R Miller PhD); International Laboratory for Air Quality and Health (Prof L Morawska PhD), School of Public Health and Social Work (M T N Tran PhD, N Wang PhD), Queensland University of Technology, Brisbane, QLD, Australia; School of Human, Health, and Social Sciences (W Mude PhD), School of Health, Medical and Applied Sciences (L Rawal PhD), CQ University, Sydney, NSW, Australia; Estia Health Blakehurst (S Neupane Kandel BSN), Estia Health, Sydney, NSW, Australia; College of Sport, Health and Engineering (T Nyanhanda PhD), Victoria University, Melbourne, VIC, Australia; College of Public Health, Medical, and Veterinary Sciences (A E Peden PhD), James Cook University, Townsville, NSW, Australia (K O Obamiro PhD); Data Mining Research Unit (A Rahman PhD), Charles Sturt University, Wagga Wagga, NSW, Australia; School of Nursing and Healthcare Professions (M Rahman PhD), Federation University Australia, Berwick, VIC, Australia; Department of Biomedical Engineering (Z Ratan MSc), Khulna University of Engineering and Technology, Khulna, Bangladesh; School of Medicine (Prof A M N Renzaho PhD, P A Saunders MPH), Translational Health Research Institute (Prof A M N Renzaho PhD), Western Sydney University, Campbelltown, NSW, Australia; Department of Psychology (Prof S R Robinson PhD), Royal Melbourne Institute of Technology University, Bundoora, VIC, Australia; Ocular Tissue Engineering Laboratory (D Roshandel MD), Lions Eye Institute, Perth, WA, Australia; Department of Health Statistics (S F Rumisha PhD), National Institute for Medical Research, Dar es Salaam, Tanzania; Department of Paediatrics (Prof S M Sawyer MD), University of Melbourne, Parkville, VIC, Australia; Centre for Adolescent Health (Prof S M Sawyer MD), Murdoch Childrens Research Institute, Parkville, VIC, Australia; Hypertension and Kidney Disease Laboratory (Prof M P Schlaich MD), Baker Heart and Diabetes Institute, Melbourne, VIC, Australia; Cardiovascular Program (X Xu PhD), The George Institute for Global Health, Sydney, NSW, Australia (Prof A E Schutte PhD, Prof J Sundström PhD); Department of Population and Health (A Seidu MPhil), University of Cape Coast, Cape Coast, Ghana; Department of Physiotherapy (S Sharma PhD), Kathmandu University, Dhulikhel, Nepal; Medical-Surgical Nursing (S Shorofi PhD), Mazandaran University of Medical Sciences, Sari, Iran; Department of Health Education and Health Promotion (S Siabani PhD), Kermanshah University of Medical Sciences, Kermanshah, Iran; School of Public Health & Zoonoses (B B Singh PhD), Guru Angad Dev Veterinary & Animal Sciences University, Ludhiana, India; College of Medicine and Public Health (J H Stephens PhD), Flinders University, Bedford

Park, SA, Australia; Department of Psychology (M A Stokes PhD), Deakin University, Burwood, VIC, Australia; The University of Queensland Centre for Clinical Research (C Sumi PhD), The University of Queensland, Woollongabba, QLD, Australia; Department of Medical Sciences (Prof J Sundström PhD), Uppsala University, Uppsala, Sweden; The Brain Institute (Prof C E I Szoeke PhD), Australian Healthy Ageing Organisation, Melbourne, VIC, Australia; University of Western Australia, Sydney, NSW, Australia (Prof K Takahashi PhD); University of Occupational and Environmental Health, Kitakyushu, Japan (Prof K Takahashi PhD); Department of Public Health and Medicine (J Taylor PhD), University of Newcastle, Callaghan, NSW, Australia; Microbiology and Immunology (M B T Tessema PhD), University of Maryland, Baltimore, MD, USA; Appleton Institute (Q G To PhD), Department of Medical and Applied Sciences (Prof C Vandelanotte PhD), Central Queensland University, Rockhampton, QLD, Australia; Health Informatics Department (M T N Tran PhD), Hanoi Medical University, Ha Noi, Viet Nam; The George Institute for Global Health (Prof M Woodward PhD), University of New South Wales, Camperdown, NSW, Australia; Menzies Institute for Medical Research (A Zarghami MD), Menzies Institute for Medical Research, Hobart, TAS, Australia; Victorian Comprehensive Cancer Centre, Melbourne, VIC, Australia (J Zhang MD)

Authors' Contributions

SMSI, RM, JS, and DC conceptualised the paper. SMSI and RU downloaded the data and created the tables and visualisations. SMSI, RM, RU, JS, and DC wrote the first draft. All other authors provided data, developed models, reviewed results, provided guidance on methodology, or reviewed and contributed to the manuscript. All authors approved the final version of the manuscript. All authors had full access to the data in the study and SMSI had the final responsibility for the decision to submit for publication. Please see appendix (pp 36-38) for more detailed information about individual author contributions to the research, divided into the following categories: providing data or critical feedback on data sources; developing methods or computational machinery; providing critical feedback on methods or results; drafting the manuscript or revising it critically for important intellectual content; and managing the estimation or publications process.

Acknowledgements: SM Shariful Islam is funded by the National Heart Foundation of Australia (102112) and a National Health and Medical Research Council (NHMRC) Emerging Leadership Fellowship (APP1195406). R Uddin is supported by Alfred Deakin Postdoctoral Research Fellowship. KM Livingstone is supported by a National Health and Medical Research Council (NHMRC) Emerging Leadership Fellowship (APP1173803). JS is supported by a NHMRC Leadership Level 2 Fellowship (APP 1176885). L N Aminde acknowledges funding by the Australian National Health and Medical Research Council (NHMRC) Emerging Leadership Fellowship (APP2018082) and holds an Honorary National Heart Foundation of Australia Fellowship (106682). B Angell acknowledges funding from the NHMRC Emerging Leadership Investigator Grant (GNT2010055). T Astell-Burt acknowledges support by an Australian Research Council Future Fellowship (FT220100857). R Borschmann acknowledges salary and research support from an NHMRC Emerging Leader Investigator Grant (EL2; GNT2008073). R Buchbinder is supported by an Australian National Health and Medical Research Council (NHMRC) Leadership 3 Fellowship (APP1194483). M Cross acknowledges support from the Global Alliance for Musculoskeletal Health. K E Doyle acknowledges support form the School of Medicine Western Sydney University. X Feng was supported by an NHMRC Career Development Fellowship (GNT1148792). V K Gupta acknowledges funding support from National Health and Medical Research Council (NHMRC), Australia. J J Hebert acknowledges salary support from the New Brunswick Health Research Foundation. H Kandel is supported by a Kornhauser Research Fellowship at The University of Sydney. J J McGrath is employed by the Queensland Centre for Mental Health Research, which received support from the Queensland Health Department. P B Mitchell is supported by an Australian NHMRC Investigator Grant. A Rahman acknowledges the support from the Data Science Research

Unit in Charles Sturt University, Wagga Wagga, NSW, Australia. S M Sawyer is supported by the Geoff and Helen Handbury Chair in Adolescent Health at The University of Melbourne, Australia, and a NHMRC Instigator Grant (GNT1196999). A E Schutte is supported by a NHMRC Leadership Investigator Grant (Application ID 2017504). S Sharma is supported by the International Association for the Study of Pain John J. Bonica Postdoctoral Fellowship. A Singh is supported by the International Graduate Research Scholarship, University of Tasmania. C E I Szoeke acknowledges support from the Royal Australasian College of Physicians. R Uddin acknowledges support by an Alfred Deakin Postdoctoral Research Fellowship, Deakin University, Australia. C Vandelanotte is supported by an Australian Research Council (ARC) Future Fellowship (ID: FT210100234). M Woodward is supported by NHMRC Program and Investigator Grants. X Xu is supported by Heart Foundation Post-doctoral Fellowship funded by the Heart Foundation of Australia (Award No. 102597), and Scientia Program at the University of New South Wales, Australia. S B Zaman acknowledges receiving a scholarship from the Australian Government Research Training Program (RTP) in support of his academic career.

Declarations

S M S Islam reports support for the present manuscript from a National Health and Medical Research Council (NHMRC) Investigator Grant and from a National Heart Foundation Vanguard Grant. B Antony reports grants or contracts form Rebecca Cooper Foundation for Investigator-initiated trial grant and Nat Rem Ltd Grant for Investigator-initiated trial biomarkers assessment support; payment or honoraria for lectures, presentations, speakers bureaus, manuscript writing or educational events from Nat Rem Ltd and IRACON; all outside the submitted work. P Atorkey reports support for the present manuscript from Australian College of Applied Professions Discipline of Psychological Sciences, Sydney, Australia. B T Baune reports consulting fees from AstraZeneca, Bristol-Myers Squibb, Janssen, LivaNova, Lundbeck, Novartis, Otsuka, Pfizer, Servier, Wyeth, Biogen, Angelini, Sumitomo Pharma, Boehringer-Ingelheim; payment or honoraria for lectures, presentations, speakers bureaus, manuscript writing or educational events from AstraZeneca, Bristol-Myers Squibb, Janssen, LivaNova, Lundbeck, Novartis, Otsuka, Pfizer, Servier, Wyeth, Biogen, Angelini, Sumitomo Pharma, Boehringer-Ingelheim; all outside the submitted work. A M Briggs reports grants from Australian National Health and Medical Research Council, Bone and Joint Decade Foundation, Arthritis and Osteoporosis Western Australia, Department of Health Government of Western Australia through payments to their institution; consulting fees from the World Health Organization; payment or honoraria for lectures, presentations, speakers bureaus, manuscript writing or educational events from American College of Rheumatology; support for attending meetings and/or travel from the World Health Organization; all outside the submitted work. R Buchbinder reports grants from Australian National Health and Medical Research (NHMRC), Medical Research Future Fund, Arthritis Australia, HCF Research Foundation, Cabrini Foundation, and Australian Government Department of Health, all as payments to their institution; royalties from UpToDate for Plantar fasciitis; all outside the submitted work. S R Clark reports grants or contracts from Wellcome Trust, Australian Department of Veterans Affairs, National Institutes of Mental Health, Janssen-Cilag Australia, Australian Defense Science & Technology Group, and Australian National Health & Medical Research Council; consulting fees from Lundbeck-Otsuka Australia; payment or honoraria for lectures, presentations, speakers bureaus, manuscript writing or educational events from Lundbeck-Otsuka Australia; support for attending meetings and/or travel from Janssen-Cilag Australia; participation on a Data Safety Monitoring Board or Advisory Board with Verbatim Trial – Orygen Australia; leadership or fiduciary role in other board, society, committee or advocacy group, unpaid, with executive Committee Member Society for Mental Health Research; all outside the submitted work. K M Livingston reports support for the present manuscript from a NHMRC Investigator Grant (APP1173803). P B Mitchell reports payment or honoraria for lectures, presentations, speakers bureaus, manuscript writing or educational events from Janssen (Australia) and Sanofi (Hangzhou); participation on a Data Safety Monitoring Board or Advisory Board with

Janssen (Australia); all outside the submitted work. A E Peden reports support for the present manuscript from the NHMRC as a grant paid to their institute. J Salmon reports grants or contracts from the National Health and Medical Research Council, the Department of Education, Victoria, and the Medical Research Future Fund; leadership or fiduciary role in other board, society, committee or advocacy group, unpaid, as President of the Asia Pacific Society for Physical Activity, a not-for-profit professional society. A E Schutte reports grants or contracts from the National Health and Medical Research Council of Australia, Leadership Investigator Grant ID2017504; personal payment or honoraria for lectures, presentations, speakers bureaus, manuscript writing or educational events from Servier, Novartis, Omron, Aktiia, and Medtronic; support for attending meetings and/or travel from Servier; participation on Abbot Advisory Board; leadership or fiduciary role in other board, society, committee or advocacy group, paid or unpaid, as Secretary of Australian Cardiovascular Alliance and co-Chair of National Hypertension Taskforce of Australia; all outside the submitted work. H Slater reports grants to their institute from Australian Government, Department of Health, Medical Research Future Fund (NH&MRC), Western Australian Government Department of Health, Bone and Joint Decade Foundation (Sweden) Curtin University (Australia), Institute for Bone and Joint Research (Australia), Canadian Memorial Chiropractic College (Canada), all outside the submitted work. S Sharma reports payment or honoraria for lectures, presentations, speakers bureaus, manuscript writing or educational events for an online lecture on “Cultural consideration for pain care” delivered on 26th January 2023, travel accommodation for delivering a talk on “Technologies for pain education in developing countries” conducted by the Pain Education SIG of the International Association for the Study of Pain; all outside the submitted work. M A Stokes reports leadership or fiduciary role in other board, society, committee or advocacy group, unpaid, as Vice President of Kidsafe Victoria, Director Council of the Aged Victoria, and Director Australasian Society of Autism Research, outside the submitted work. J Sundström reports stock or stock options in Anagram kommunikation AB, MinForskning AB, and Symptoms Europe AB, outside the submitted work. A G Thrift reports grants or contracts from National Health & Medical Research Council (Australia), grant numbers 1143155, 1171966, 1182071, and Medical Research Future Fund (Australian Government), grant number 2015976, as payments to their institution outside the submitted work. M Woodward reports consulting fees from Amgen and Freeline; participation on a Data Safety Monitoring Board or Advisory Board with Staree DSMB; all outside the submitted work.

Supplementary Appendix:

Table of Contents

Authors' Contributions 36

Supplementary Table 1 Guidelines for Accurate and Transparent Health Estimates Reporting (GATHER) checklist 39

Supplementary Table 2 Age-standardized death rates, YLL rates, YLD rates, life expectancy at birth, and health-adjusted life expectancy at <1 year of age for both sexes combined relative to comparator countries with high SDI, 1990 and 2019 41

Supplementary Table 3 Age-standardized rate (95% uncertainty interval [UI]) of DALYs with percentage changes between 1990-2010, 2010-2019, and 1990-2019 for the leading causes of diseases, disabilities, and injuries in Australia 44

Supplementary Figure 1 46

Supplementary Table 4 Data Sources for Australia: GBD Data input sources causes of death 49

Supplementary Table 5 GBD data input sources all covariates 55

Supplementary Table 6 GBD data input sources for mortality 80

Supplementary Table 7 GBD data input sources for neonatal health outcomes- all cause mortality 88

Supplementary Table 8 GBD data input sources for all risk factors 122

DisMod-MR 141

Authors' Contributions

Providing data or critical feedback on data sources

Tim Adair, Oyelola A Adegboye, Zanfina Ademi, Bright Opoku Ahinkorah, Kefyalew Addis Alene, Sheikh Mohammad Alif, Azmeraw T. Amare, Edward Kwabena Ameyaw, Deanna Anderlini, Prince Atorkey, Beatriz Paulina Ayala Quintanilla, Getinet Ayano, Dinesh Bhandari, Soufiane Boufous, Andre F Carvalho, Marita Cross, Abel Fekadu Dadi, Barbora de Courten, Kerrie E Doyle, David Edvardsson, Hailay Abrha Gesesew, Myron Anthony Godinho, Bhawna Gupta, Vivek Kumar Gupta, Mohammad Hamiduzzaman, Simon I Hay, Sheikh Mohammed Shariful Islam, Billingsley Kaambwa, Himal Kandel, Asaduzzaman Khan, Luke D Knibbs, James Leigh, Janni Leung, Rashidul Alam Mahumud, Abdullah A Mamun, Melvin Barrientos Marzan, Atte Meretoja, Ted R Miller, Ali H Mokdad, William Mude, Christopher J L Murray, Sandhya Neupane Kandel, Kehinde O Obamiro, Kevan R Polkinghorne, Lal Rawal, Lennart Reifels, Andre M. N. Renzaho, Stephen R Robinson, Markus P Schlaich, Abdul-Aziz Seidu, Soraya Siabani, Ambrish Singh, Narayan Subedi Subedi, Jing Sun, Cassandra E I Szoeke, Mai Thi Ngoc Tran, Riaz Uddin, Mark Woodward, and Xiaoyue Xu.

Developing methods or computational machinery

Mohammad Saidul Islam, Sheikh Mohammed Shariful Islam, James Leigh, Rashidul Alam Mahumud, Ali H Mokdad, Christopher J L Murray, and Ambrish Singh.

Providing critical feedback on methods or results

Ilana N Ackerman, Tim Adair, Oyelola A Adegboye, Zanfina Ademi, Ripon Kumar Adhikary, Bright Opoku Ahinkorah, Khurshid Alam, Kefyalew Addis Alene, Sheikh Mohammad Alif, Azmeraw T. Amare, Edward Kwabena Ameyaw, Leopold N Aminde, Deanna Anderlini, Blake Angell, Adnan Ansar, Benny Antony, Anayochukwu Edward Anyasodor, Victoria Kiriaki Arnet, Thomas Astell-Burt, Prince Atorkey, Mamaru Ayenew

Awoke, Beatriz Paulina Ayala Quintanilla, Getinet Ayano, Nasser Bagheri, Kylie Ball, Anthony Barnett, Bernhard T Baune, Dinesh Bhandari, Sonu Bhaskar, Raaj Kishore Biswas, Soufiane Boufous, Andrew M Briggs, Rachelle Buchbinder, Norma B. Bulamu, Richard A Burns, Andre F Carvalho, Ester Cerin, Nicolas Cherbuin, Enayet Karim Chowdhury, Liliana G Ciobanu, Scott Richard Clark, David A Crawford, Marita Cross, Abel Fekadu Dadi, Barbora de Courten, Diego De Leo, Katie de Luca, Kerrie E Doyle, David Edvardsson, Kristina Edvardsson, Xiaoqi Feng, Bernadette Mary Fitzgibbon, Joanne Flavel, Eyob Alemayehu Alemayehu Gebreyohannes, Hailay Abrha Gesesew, Tiffany K Gill, Myron Anthony Godinho, Bhawna Gupta, Vivek Kumar Gupta, Mitiku Teshome Hambisa, Mohammad Hamiduzzaman, Hossein Hassanian-Moghaddam, Simon I Hay, Jeffrey J Hebert, M Mamun Huda, Tanvir M Huda, M Mofizul Islam, Mohammad Saidul Islam, Sheikh Mohammed Shariful Islam, Billingsley Kaambwa, Himal Kandel, Gizat M. Kassie, Jaimon Terence Kelly, Jessica A Kerr, Asaduzzaman Khan, Girmay Tsegay Kiross, Luke D Knibbs, Ratilal Lalloo, Long Khanh Dao Le, Janni Leung, Shanshan Li, Ralph Maddison, Rashidul Alam Mahumud, Abdullah A Mamun, Melvin Barrientos Marzan, Atte Meretoja, Amanual Getnet Mersha, Ted R Miller, Philip B Mitchell, Ali H Mokdad, Lidia Morawska, Christine Mpundu-Kaambwa, William Mude, Christopher J L Murray, Sandhya Neupane Kandel, Tafadzwa Nyanhanda, Kehinde O Obamiro, Amy E Peden, Konrad Pesudovs, Kevan R Polkinghorne, Azizur Rahman, Muhammad Aziz Rahman, Zubair Ahmed Ratan, Lal Rawal, Lennart Reifels, Andre M. N. Renzaho, Stephen R Robinson, Danial Roshandel, Paul A Saunders, Susan M Sawyer, Markus P Schlaich, Aletta Elisabeth Schutte, Saurab Sharma, Seyed Afshin Shorofi, Soraya Siabani, Ambrish Singh, Balbir Bagicha Singh, Jacqueline H Stephens, Mark A Stokes, Narayan Subedi Subedi, Chandra Datta Sumi, Jing Sun, Cassandra E I Szoeke, Santosh Kumar Tadakamadla, Ken Takahashi, Jo Taylor, Melkamu B Tessema Tessema, Amanda G Thrift, Quyen G To, Daniel Nigusse Tollosa, Mai Thi Ngoc Tran, Riaz Uddin, Corneel Vandelanotte, Blesson Mathew Varghese, Ning Wang, Paul Ward, Mark Woodward, Befikadu Legesse Wubishet, Xiaoyue Xu, Pengpeng Ye, Sojib Bin Zaman, Amin Zarghami, and Jianrong Zhang.

Drafting the work or revising it critically for important intellectual content

Ilana N Ackerman, Ripon Kumar Adhikary, Bright Opoku Ahinkorah, Khurshid Alam, Kefyalew Addis Alene, Sheikh Mohammad Alif, Azmeraw T. Amare, Leopold N Aminde, Deanna Anderlini, Blake Angell, Benny Antony, Anayochukwu Edward Anyasodor, Beatriz Paulina Ayala

Quintanilla, Kylie Ball, Anthony Barnett, Bernhard T Baune, Dinesh Bhandari, Sonu Bhaskar, Rohan Borschmann, Soufiane Boufous, Norma B. Bulamu, Richard A Burns, Andre F Carvalho, Ester Cerin, Nicolas Cherbuin, Scott Richard Clark, David A Crawford, Marita Cross, Barbora de Courten, Katie de Luca, David Edvardsson, Kristina Edvardsson, Ferry Efendi, Aklilu Endalamaw, Nelsensius Klau Fauk, Bernadette Mary Fitzgibbon, Hailay Abrha Gesesew, Tiffany K Gill, Myron Anthony Godinho, Bhawna Gupta, Vivek Kumar Gupta, Mitiku Teshome Hambisa, Mohammad Hamiduzzaman, Graeme J Hankey, Simon I Hay, Jeffrey J Hebert, Rakibul M Islam, Sheikh Mohammed Shariful Islam, Himal Kandel, Gizat M. Kassie, Jessica A Kerr, Vishnutheertha Vishnutheertha Kulkarni, Long Khanh Dao Le, Janni Leung, Shanshan Li, Katherine M Livingstone, Ralph Maddison, Rashidul Alam Mahumud, Abdullah A Mamun, John J. McGrath, Max L Mehlman, Atte Meretoja, Ted R Miller, Philip B Mitchell, Ali H Mokdad, Christine Mpundu-Kaambwa, Christopher J L Murray, Sandhya Neupane Kandel, Kehinde O Obamiro, Amy E Peden, Konrad Pesudovs, Kevan R Polkinghorne, Azizur Rahman, Muhammad Aziz Rahman, Zubair Ahmed Ratan, Lal Rawal, Andre M. N. Renzaho, Susan Fred Rumisha, Jo Salmon, Susan M Sawyer, Aletta Elisabeth Schutte, Saurab Sharma, Seyed Afshin Shorofi, Helen Slater, Jacqueline H Stephens, Mark A Stokes, Narayan Subedi Subedi, Johan Sundström, Cassandra E I Szoeke, Santosh Kumar Tadakamadla, Quyen G To, Mai Thi Ngoc Tran, Riaz Uddin, Corneel Vandelanotte, Lennert J Veerman, Paul Ward, Mark Woodward, Sojib Bin Zaman, Amin Zarghami, and Jianrong Zhang.

Managing the estimation or publications process

Sheikh Mohammed Shariful Islam, Riaz Uddin, Ali H Mokdad, Christopher J L Murray, Kevan R Polkinghorne, Max L Mehlman and Simon I Hay.

Supplementary Table 1 Guidelines for Accurate and Transparent Health Estimates Reporting (GATHER) checklist

Checklist of information that should be included in new reports of global health estimates

Item #	Checklist item	Reported on page #
**Objectives and funding**		
1	Define the indicator(s), populations (including age, sex, and geographic entities), and time period(s) for which estimates were made.	Page 5
2	List the funding sources for the work.	Page 2
**Data Inputs**		
For all data inputs from multiple sources that are synthesized as part of the study:		
3	Describe how the data were identified and how the data were accessed.	Page 5
4	Specify the inclusion and exclusion criteria. Identify all ad-hoc exclusions.	Page 5
5	Provide information on all included data sources and their main characteristics. For each data source used, report reference information or contact name/institution, population represented, data collection method, year(s) of data collection, sex and age range, diagnostic criteria or measurement method, and sample size, as relevant.	Page 5
6	Identify and describe any categories of input data that have potentially important biases (e.g., based on characteristics listed in item 5).	Page 5-7
For data inputs that contribute to the analysis but were not synthesized as part of the study:		
7	Describe and give sources for any other data inputs.	Page 5
For all data inputs:		
8	Provide all data inputs in a file format from which data can be efficiently extracted (e.g., a spreadsheet rather than a PDF), including all relevant meta-data listed in item 5. For any data inputs that cannot be shared because of ethical or legal reasons, such as third-party ownership, provide a contact name or the name of the institution that retains the right to the data.	Page 5-7

Data analysis		
**9**	Provide a conceptual overview of the data analysis method. A diagram may be helpful.	Page 5-7
**10**	Provide a detailed description of all steps of the analysis, including mathematical formulae. This description should cover, as relevant, data cleaning, data pre-processing, data adjustments and weighting of data sources, and mathematical or statistical model(s).	Page 5-7 and Supplementary files
**11**	Describe how candidate models were evaluated and how the final model(s) were selected.	Page 5-7 and Supplementary files
**12**	Provide the results of an evaluation of model performance, if done, as well as the results of any relevant sensitivity analysis.	Page 5-7 and Supplementary file
**13**	Describe methods for calculating uncertainty of the estimates. State which sources of uncertainty were, and were not, accounted for in the uncertainty analysis.	Page 5-7 and Supplementary files
**14**	State how analytic or statistical source code used to generate estimates can be accessed.	Page 5-7 and Supplementary files
**Results and Discussion**		
**15**	Provide published estimates in a file format from which data can be efficiently extracted.	Page 7-9
**16**	Report a quantitative measure of the uncertainty of the estimates (e.g. uncertainty intervals).	Page 7-9
**17**	Interpret results in light of existing evidence. If updating a previous set of estimates, describe the reasons for changes in estimates.	Page 9-12
**18**	Discuss limitations of the estimates. Include a discussion of any modelling assumptions or data limitations that affect interpretation of the estimates.	Page 11-12

This checklist should be used in conjunction with the GATHER statement and Explanation and Elaboration document, found on gather-statement.org

Supplementary Table 2 Age-standardized death rates, YLL rates, YLD rates, life expectancy at birth, and health-adjusted life expectancy at <1 year of age for both sexes combined relative to comparator countries with high SDI, 1990 and 2019

Country	Death per 100,000 (age-standardized)				YLLs per 100,000 (age-standardized)				YLDs per 100,000 (age-standardized)				Life expectancy at birth				Health-adjusted life expectancy			
	1990		2019		1990		2019		1990		2019		1990		2019		1990		2019	
	Rate (95% UI)	Rank	Rate (95% UI)	Rank	Rate (95% UI)	Rank	Rate (95% UI)	Rank	Rate (95% UI)	Rank	Rate (95% UI)	Rank	LE (95% UI)	Rank	LE (95% UI)	Rank	HALE (95% UI)	Rank	HALE (95% UI)	Rank
NOR	651 (645 to 657)	7	394 (388 to 401)	5	14452 (14300 to 14602)	7	7536 (7391 to 7691)	4	10987 (8099 to 14218)	10	10925 (8055 to 14224)	9	76.9 (76.7 to 77.0)	7	82.9 (82.7 to 83.1)	4	66.4 (63.3 to 69.1)	7	70.8 (67.4 to 73.9)	5
CHE	591 (587 to 596)	2	353 (345 to 362)	3	13443 (13323 to 13566)	3	6907 (6678 to 7159)	2	11476 (8501 to 14786)	11	10728 (7945 to 13844)	6	77.9 (77.8 to 78.0)	2	84.0 (83.7 to 84.2)	3	66.8 (63.6 to 69.7)	5	72.0 (68.6 to 75.1)	3
IRL	796 (787 to 805)	15	431 (416 to 446)	10	16626 (16398 to 16846)	13	8320 (7935 to 8752)	7	10888 (8062 to 14110)	7	11081 (8177 to 14373)	11	75.0 (74.9 to 75.1)	15	82.0 (81.7 to 82.4)	8	65.3 (62.5 to 67.8)	12	70.4 (67.0 to 73.3)	8
DEU	726 (724 to 728)	13	463 (455 to 471)	13	15979 (15922 to 16036)	9	9126 (8946 to 9330)	12	10754 (7936 to 13982)	6	10949 (8072 to 14255)	10	75.7 (75.6 to 75.7)	9	81.2 (81.0 to 81.4)	12	65.8 (62.9 to 68.3)	9	69.7 (66.4 to 72.7)	12
ISL	588 (570 to 607)	1	348 (322 to 379)	2	12840 (12363 to 13336)	1	6973 (6307 to 7795)	3	10522 (7727 to 13680)	5	10448 (7710 to 13570)	3	78.1 (77.7 to 78.5)	1	84.1 (83.2 to 84.9)	2	67.9 (65.0 to 70.6)	1	72.3 (69.1 to 75.4)	2
SWE	605 (601 to 609)	3	398 (393 to 403)	6	12879 (12777 to 12986)	2	7595 (7477 to 7723)	5	10495 (7764 to 13633)	4	10474 (7727 to 13605)	4	77.9 (77.9 to 78.0)	3	82.8 (82.7 to 83.0)	6	67.8 (64.8 to 70.4)	2	71.4 (68.1 to 74.3)	4

SGP	719 (712 to 726)	11	324 (316 to 333)	1	16009 (15816 to 16210)	10	6191 (5965 to 6454)	1	9481 (7055 to 12396)	1	8854 (6537 to 11575)	1	75.6 (75.5 to 75.7)	11	84.9 (84.6 to 85.1)	1	66.7 (64.0 to 69.0)	6	74.5 (71.4 to 77.1)	1
**AUS**	638 (634 to 641)	**5**	389 (381 to 398)	**4**	14420 (14324 to 14514)	**6**	8041 (7811 to 8295)	**6**	11589 (8639 to 14969)	**13**	11566 (8613 to 14936)	13	77.0 (76.9 to 77.1)	6	82.9 (82.7 to 83.1)	5	66.1 (62.9 to 68.8)	8	70.3 (66.7 to 73.4)	10
NLD	647 (643 to 651)	6	443 (432 to 455)	11	13871 (13773 to 13971)	4	8503 (8200 to 8844)	8	10217 (7556 to 13204)	2	10385 (7710 to 13439)	2	77.2 (77.1 to 77.2)	5	81.7 (81.5 to 82.0)	11	67.4 (64.5 to 69.9)	3	70.6 (67.5 to 73.4)	6
DNK	735 (729 to 741)	14	462 (449 to 477)	12	16594 (16429 to 16754)	12	9162 (8798 to 9566)	13	10960 (8124 to 14165)	8	10768 (7956 to 13905)	7	75.2 (75.1 to 75.3)	14	81.1 (80.8 to 81.4)	13	65.3 (62.3 to 67.8)	13	69.9 (66.7 to 72.8)	11
CAN	611 (609 to 614)	4	410 (405 to 416)	7	13932 (13867 to 14001)	5	8964 (8773 to 9172)	10	10315 (7631 to 13379)	3	10720 (7955 to 13796)	5	77.4 (77.4 to 77.5)	4	82.2 (82.0 to 82.3)	7	67.3 (64.4 to 69.9)	4	70.5 (67.2 to 73.4)	7
USA	717 (715 to 720)	8	528 (524 to 533)	15	16804 (16771 to 16835)	15	12724 (12587 to 12868)	15	12553 (9394 to 16115)	15	13337 (9990 to 17050)	15	75.7 (75.7 to 75.7)	10	78.9 (78.8 to 79.0)	15	63.9 (60.6 to 66.8)	15	65.4 (61.8 to 68.8)	15
GBR	669 (668 to 670)	10	466 (462 to 470)	14	15587 (15529 to 15647)	8	9372 (9245 to 9507)	14	11520 (8523 to 14921)	12	11585 (8575 to 14984)	14	75.8 (75.8 to 75.9)	8	81.1 (81.0 to 81.2)	14	65.4 (62.3 to 68.1)	11	69.1 (65.7 to 72.2)	14
FIN	723 (714 to 732)	12	428 (415 to 443)	8	16375 (16174 to 16594)	11	8765 (8419 to 9144)	9	10963 (8075 to 14222)	9	10805 (7991 to 14005)	8	75.4 (75.3 to 75.6)	13	81.9 (81.5 to 82.2)	9	65.4 (62.5 to 68.0)	10	70.3 (67.0 to 73.3)	9
NZL	699 (692 to 705)	9	430 (423 to 437)	9	16667 (16490 to 16852)	14	9122 (8910 to 9340)	11	11884 (8756 to 15349)	14	11560 (8545 to 14904)	12	75.6 (75.4 to 75.7)	12	81.8 (81.6 to 82.0)	10	64.7 (61.5 to 67.5)	14	69.6 (66.1 to 72.7)	13

NOR=Norway, CHE=Switzerland, IRL=Ireland, DEU=Germany, ISL=Iceland, SWE=Sweden, SGP=Singapore, AUS=Australia, NLD=The Netherlands, DNK=Denmark, CAN=Canada, USA= The United States of America, GBR=United Kingdom of Great Britain and Northern Ireland, FIN=Finland, NZL=New Zealand. Note: 1 indicates the best rank and 15 the worst.

Supplementary Table 3 Age-standardized rate (95% uncertainity interval [UI]) of DALYs with percentage changes between 1990-2010, 2010-2019, and 1990-2019 for the leading causes of diseases, disabilities, and injuries in Australia

Cause	DALY rank			DALYs per 100 000 population (95% UI)			Age standardised % change in DALYs (95% UI)		
	1990	2010	2019	1990	2010	2019	1990-2010	2010-2019	1990-2019
Low back pain	2	1	1	1115.3 (784.1 to 1484.7)	1080.5 (763.6 to 1443.3)	986.6 (685.3 to 1334.1)	-3.1 (-6.7 to 0.6)	-8.7 (-14.8 to -2.8)	-11.5 (-17.5 to -5.6)
Ischemic heart disease	1	2	2	3045.6 (2903.0 to 3131.8)	1063.3 (982.4 to 1114.2)	879.0 (807.4 to 930.1)	-65.1 (-66.3 to -64.0)	-17.3 (-20.6 to -14.3)	-71.1 (-72.5 to -69.9)
Depressive disorders	6	3	3	799.5 (550.6 to 1084.7)	861.6 (586.2 to 1182.1)	798.7 (549.4 to 1128.5)	7.8 (0.1 to 15.9)	-7.3 (-15.9 to 2.3)	-0.1 (-7.9 to 7.8)
Falls	11	5	4	557.7 (409.5 to 752.2)	628.9 (465.4 to 839.8)	670.6 (493.2 to 898.7)	12.8 (10.2 to 15.2)	6.6 (4.7 to 8.4)	20.2 (17.8 to 22.8)
Other musculoskeletal disorders	12	4	5	555.4 (386.1 to 767.4)	651.8 (455.8 to 890.9)	667.1 (456.7 to 917.7)	17.4 (9.9 to 24.9)	2.3 (-6.9 to 11.3)	20.1 (9.8 to 30.4)
Drug use disorders	18	13	6	443.2 (347.7 to 551.0)	505.0 (397.0 to 619.7)	609.2 (482.1 to 753.2)	13.9 (2.7 to 28.3)	20.6 (9.2 to 32.8)	37.4 (24.7 to 52.9)
Anxiety disorders	14	10	7	548.4 (362.0 to 775.8)	536.7 (381.0 to 724.2)	555.3 (366.7 to 785.0)	-2.1 (-18.7 to 19.1)	3.5 (-13.2 to 23.5)	1.3 (-5.4 to 9.1)
Headache disorders	13	7	8	550.3 (122.6 to 1188.8)	551.6 (123.3 to 1192.6)	551.5 (122.4 to 1178.5)	0.2 (-3.0 to 3.3)	0.0 (-2.7 to 3.1)	0.2 (-3.0 to 3.5)
Self-harm	9	9	9	639.7 (620.1 to 660.7)	539.1 (523.6 to 555.2)	518.0 (491.9 to 551.0)	-15.7 (-19.1 to -12.4)	-3.9 (-9.2 to 2.7)	-19.0 (-23.5 to -13.6)
Chronic obstructive pulmonary disease	8	11	10	720.1 (665.4 to 769.3)	517.3 (459.8 to 574.6)	497.1 (440.4 to 553.0)	-28.2 (-33.5 to -21.6)	-3.9 (-11.7 to 3.5)	-31.0 (-36.6 to -24.8)
Tracheal, bronchus, and lung cancer	7	8	11	767.4 (745.9 to 788.1)	546.5 (522.0 to 563.0)	491.6 (461.2 to 520.1)	-28.8 (-31.3 to -26.3)	-10.1 (-14.8 to -5.0)	-35.9 (-39.7 to -32.0)
Diabetes mellitus	20	14	12	409.5 (343.5 to 486.2)	484.9 (386.0 to 610.1)	480.8 (365.6 to 613.0)	18.4 (9.7 to 29.2)	-0.8 (-6.9 to 5.7)	17.4 (6.1 to 30.6)
Neonatal disorders	5	6	13	805.4 (741.7 to	563.7 (513.4 to	449.3 (388.5 to	-30.0 (-36.7 to -	-20.3 (-30.2 to	-44.2 (-52.3 to -

				882.1)	620.3)	512.8)	23.6)	-9.8)	35.9)
Stroke	3	12	14	1058.9 (985.0 to 1115.9)	507.6 (457.4 to 545.6)	445.2 (398.8 to 487.9)	-52.1 (-54.3 to -50.1)	-12.3 (-16.9 to -7.4)	-58.0 (-60.7 to -55.3)
Exposure to mechanical forces	17	16	15	505.6 (350.9 to 728.9)	433.2 (290.3 to 640.7)	440.2 (294.4 to 658.1)	-14.3 (-17.3 to -12.1)	1.6 (0.0 to 3.1)	-12.9 (-16.4 to -10.2)
Asthma	10	17	16	576.5 (421.3 to 775.2)	368.9 (251.3 to 527.2)	373.8 (245.5 to 549.8)	-36.0 (-43.9 to -28.0)	1.3 (-11.1 to 13.5)	-35.2 (-46.0 to -22.6)
Road injuries	4	15	17	952.8 (912.7 to 997.9)	451.7 (423.4 to 482.7)	365.0 (335.0 to 397.0)	-52.6 (-54.5 to -50.5)	-19.2 (-23.8 to -14.8)	-61.7 (-64.0 to -59.3)
Age-related and other hearing loss	22	18	18	382.3 (265.9 to 542.8)	355.5 (245.1 to 511.4)	355.2 (240.5 to 508.3)	-7.0 (-10.8 to -3.3)	-0.1 (-5.0 to 5.4)	-7.1 (-12.4 to -1.7)
Colon and rectum cancer	16	19	19	522.9 (504.4 to 539.1)	350.4 (330.7 to 365.7)	334.6 (309.2 to 359.1)	-33.0 (-35.5 to -30.4)	-4.5 (-10.4 to 2.2)	-36.0 (-40.2 to -31.5)
Endocrine, metabolic, blood, and immune disorders	26	22	20	289.4 (220.1 to 370.9)	327.8 (258.1 to 411.1)	330.5 (261.0 to 415.1)	13.3 (7.7 to 21.0)	0.8 (-3.4 to 5.0)	14.2 (7.7 to 23.1)
Gynecological diseases	23	21	21	333.3 (230.6 to 466.7)	331.6 (227.8 to 466.6)	328.5 (226.7 to 464.1)	-0.5 (-3.6 to 3.0)	-0.9 (-4.2 to 2.2)	-1.5 (-4.7 to 1.9)
Alzheimer's disease and other dementias	24	23	22	326.5 (146.8 to 726.9)	319.3 (146.5 to 690.3)	318.9 (145.9 to 676.1)	-2.2 (-7.1 to 3.2)	-0.1 (-3.5 to 3.6)	-2.3 (-7.0 to 3.2)
Oral disorders	19	24	23	423.2 (272.3 to 618.0)	308.0 (192.7 to 461.7)	315.3 (193.6 to 482.2)	-27.2 (-32.2 to -21.5)	2.4 (-9.2 to 15.6)	-25.5 (-35.4 to -14.7)
Osteoarthritis	27	25	24	283.0 (143.0 to 565.8)	297.7 (152.2 to 590.2)	312.9 (158.4 to 634.0)	5.2 (-0.9 to 10.9)	5.1 (-0.1 to 11.3)	10.6 (6.9 to 14.5)
Congenital birth defects	15	20	25	528.5 (433.4 to 581.1)	344.6 (309.8 to 393.9)	288.5 (245.9 to 344.5)	-34.8 (-41.1 to -16.3)	-16.3 (-25.4 to -5.0)	-45.4 (-53.4 to -24.3)

Note causes are presented by DALYs ranking in 2019.
UI=uncertainty interval.

Supplementary Figure 1A Ranking of the leading causes of age-standardized rates of disability-adjusted life years (DALYs) compared to countries with high SDI, 1990

Both sexes, Age-standardized, 1990, DALYs per 100,000

	**Australia**	Norway	Switzerland	Ireland	Germany	Iceland	Sweden	Singapore	Netherlands	Denmark	Canada	United States of America	United Kingdom	Finland	New Zealand
Ischemic heart disease	1	1	1	1	1	1	1	1	1	1	1	1	1	1	1
Low back pain	2	3	2	3	3	2	3	6	4	3	4	2	3	4	4
Stroke	3	2	3	2	2	3	2	2	2	2	5	6	2	2	3
Road injuries	4	9	7	6	4	6	7	14	9	9	3	4	11	7	2
Neonatal disorders	5	5	11	11	7	7	10	10	5	11	7	5	5	11	7
Depressive disorders	6	14	8	8	16	10	5	13	10	8	11	11	6	6	14
Lung cancer	7	10	6	5	6	4	12	5	3	4	2	3	4	9	5
COPD	8	20	17	4	12	11	20	4	6	6	12	7	7	18	6
Self-harm	9	4	4	16	9	8	4	9	15	5	8	13	17	3	8
Asthma	10	12	18	18	21	16	11	18	21	25	28	23	9	25	9
Falls	11	7	5	14	8	9	8	16	17	10	17	20	16	5	12
Other musculoskeletal	12	26	22	20	22	18	23	11	19	22	6	10	22	26	31
Headache disorders	13	6	10	7	5	5	6	20	8	12	9	9	8	8	16
Anxiety disorders	14	8	12	12	13	12	13	24	11	16	14	16	20	16	10
Congenital defects	15	11	9	9	14	15	9	8	7	7	10	14	12	10	13
Colorectal cancer	16	16	25	13	17	23	18	12	16	14	13	19	15	23	11
Mechanical forces	17	32	27	36	48	38	33	29	43	40	32	34	51	40	15
Drug use disorders	18	28	15	40	33	28	44	55	41	27	22	17	25	28	32
Oral disorders	19	22	26	23	25	22	25	31	20	23	24	30	26	17	21
Diabetes	20	17	16	22	10	25	16	7	12	21	15	8	19	14	22
Breast cancer	21	21	14	17	19	17	21	25	13	13	16	18	13	20	18
Age-related hearing loss	22	25	31	27	28	29	29	19	26	31	18	21	23	29	23
Gynecological diseases	23	15	13	15	15	14	14	30	14	15	27	31	14	13	19
Alzheimer's disease	24	23	24	25	24	24	24	23	22	28	20	28	24	22	24
SIDS	25	31	43	29	32	52	38	136	52	56	41	36	31	73	17

Supplementary Figure 1B Ranking of the leading causes of age-standardized rates of disability-adjusted life years (DALYs) compared to countries with high SDI, 2010

Both sexes, Age-standardized, 2010, DALYs per 100,000

	**Australia**	Norway	Switzerland	Ireland	Germany	Iceland	Sweden	Singapore	Netherlands	Denmark	Canada	United States of America	United Kingdom	Finland	New Zealand
Low back pain	1	2	1	2	2	2	2	3	2	1	3	2	2	3	2
Ischemic heart disease	2	1	2	1	1	1	1	1	3	2	1	1	1	1	1
Depressive disorders	3	9	6	3	6	9	3	12	7	7	9	8	6	4	5
Other musculoskeletal	4	23	15	17	16	13	13	5	13	17	2	5	15	24	27
Falls	5	6	3	10	9	5	6	8	14	9	14	19	11	2	3
Neonatal disorders	6	16	8	16	12	16	18	16	9	11	5	9	8	19	6
Headache disorders	7	3	5	4	3	3	4	9	5	6	6	12	5	7	12
Lung cancer	8	7	7	5	4	4	9	7	1	3	4	6	3	11	11
Self-harm	9	10	10	9	14	12	7	14	15	18	8	13	22	5	8
Anxiety disorders	10	5	4	6	7	7	10	21	8	16	15	14	13	15	4
COPD	11	8	14	7	11	8	11	15	4	4	11	4	4	17	7
Stroke	12	4	11	8	5	6	5	4	6	5	10	11	7	6	9
Drug use disorders	13	12	18	13	25	18	19	44	34	21	7	3	10	14	26
Diabetes	14	13	12	19	8	14	8	6	12	13	12	7	9	9	17
Road injuries	15	24	22	23	20	25	24	22	22	22	13	10	28	16	10
Mechanical forces	16	37	30	42	47	37	36	28	44	44	41	45	50	33	13
Asthma	17	18	24	22	30	19	23	32	26	28	27	20	14	26	23
Age-related hearing loss	18	22	27	28	26	27	29	10	25	31	18	15	24	28	18
Colorectal cancer	19	14	23	18	15	22	14	11	11	12	17	25	20	25	15
Congenital defects	20	20	13	12	22	24	20	19	18	23	16	17	16	18	14
Gynecological diseases	21	17	9	11	10	11	15	25	10	10	25	30	12	13	16
Endo/metab/blood/immune	22	11	21	15	13	17	17	17	17	14	19	22	17	20	22
Alzheimer's disease	23	19	17	24	23	21	21	18	19	26	20	26	26	21	20
Oral disorders	24	15	26	26	24	20	25	24	21	25	22	32	30	23	24
Osteoarthritis	25	21	25	27	27	10	30	13	27	24	24	24	27	27	25

IHME

Supplementary Figure 1C Ranking of the leading causes of age-standardized rates of disability-adjusted life years (DALYs) compared to countries with high SDI, 2019

Both sexes, Age-standardized, 2019, DALYs per 100,000

	**Australia**	Norway	Switzerland	Ireland	Germany	Iceland	Sweden	Singapore	Netherlands	Denmark	Canada	United States of America	United Kingdom	Finland	New Zealand
Low back pain	1	1	1	2	2	1	2	2	1	1	2	3	2	2	2
Ischemic heart disease	2	2	2	1	1	2	1	1	3	2	1	2	1	1	1
Depressive disorders	3	6	6	3	8	7	3	11	7	7	8	7	3	4	5
Falls	4	4	3	8	7	5	5	9	9	8	13	16	10	3	3
Other musculoskeletal	5	21	12	13	15	13	15	4	14	17	3	4	16	16	28
Drug use disorders	6	14	17	11	25	12	13	39	29	19	4	1	9	10	26
Anxiety disorders	7	5	5	5	6	6	9	19	5	12	14	14	14	15	4
Headache disorders	8	3	4	4	3	3	4	7	4	5	6	12	4	5	7
Self-harm	9	13	11	14	14	14	7	16	15	18	9	13	22	7	8
COPD	10	7	16	7	11	9	11	21	6	4	12	5	5	18	6
Lung cancer	11	9	8	6	4	4	12	8	2	3	5	8	6	12	9
Diabetes	12	10	10	12	5	10	8	6	12	11	10	6	7	8	14
Neonatal disorders	13	17	9	20	13	18	19	17	11	14	7	9	11	23	13
Stroke	14	8	13	10	9	11	6	5	8	6	11	10	8	6	10
Mechanical forces	15	38	35	41	46	37	36	27	46	43	39	40	47	37	12
Asthma	16	22	22	23	32	21	18	32	22	33	29	18	18	25	27
Road injuries	17	28	26	29	23	27	25	26	26	26	15	11	30	21	11
Age-related hearing loss	18	19	27	27	27	23	29	10	25	29	18	19	23	28	18
Colorectal cancer	19	15	25	17	16	24	17	15	13	16	16	26	20	24	16
Endo/metab/blood/immune	20	11	19	15	12	17	16	14	16	13	19	24	15	20	21
Gynecological diseases	21	12	7	9	10	8	14	23	10	10	26	31	13	9	15
Alzheimer's disease	22	18	18	21	22	20	22	13	19	24	20	27	26	19	22
Oral disorders	23	16	21	24	20	19	20	22	18	20	23	30	28	17	19
Osteoarthritis	24	20	23	25	28	15	28	12	23	23	22	23	27	26	25
Congenital defects	25	24	15	18	24	25	23	20	21	22	17	22	21	22	17

Supplementary Table 4 Data Sources for Australia: GBD Data input sources causes of death

Citation
Australian Bureau of Statistics. Australia Vital Registration - Deaths 2005
Australia Vital Registration - Deaths 1980 ICD9 as it appears in World Health Organization (WHO). WHO Mortality Database Version November 2015. Geneva, Switzerland: World Health Organization (WHO)
Australia Vital Registration - Deaths 1981 ICD9 as it appears in World Health Organization (WHO). WHO Mortality Database Version November 2015. Geneva, Switzerland: World Health Organization (WHO)
Australia Vital Registration - Deaths 1982 ICD9 as it appears in World Health Organization (WHO). WHO Mortality Database Version November 2015. Geneva, Switzerland: World Health Organization (WHO)
Australia Vital Registration - Deaths 1983 ICD9 as it appears in World Health Organization (WHO). WHO Mortality Database Version November 2015. Geneva, Switzerland: World Health Organization (WHO)
Australia Vital Registration - Deaths 1984 ICD9 as it appears in World Health Organization (WHO). WHO Mortality Database Version November 2015. Geneva, Switzerland: World Health Organization (WHO)
Australia Vital Registration - Deaths 1985 ICD9 as it appears in World Health Organization (WHO). WHO Mortality Database Version November 2015. Geneva, Switzerland: World Health Organization (WHO)
Australia Vital Registration - Deaths 1986 ICD9 as it appears in World Health Organization (WHO). WHO Mortality Database Version November 2015. Geneva, Switzerland: World Health Organization (WHO)
Australia Vital Registration - Deaths 1987 ICD9 as it appears in World Health Organization (WHO). WHO Mortality Database Version November 2015. Geneva, Switzerland: World Health Organization (WHO)
Australia Vital Registration - Deaths 1988 ICD9 as it appears in World Health Organization (WHO). WHO Mortality Database Version November 2015. Geneva, Switzerland: World Health Organization (WHO)
Australia Vital Registration - Deaths 1989 ICD9 as it appears in World Health Organization (WHO). WHO Mortality Database Version November 2015. Geneva, Switzerland: World Health Organization (WHO)
Australia Vital Registration - Deaths 1990 ICD9 as it appears in World Health Organization (WHO). WHO Mortality Database Version November 2015. Geneva, Switzerland: World Health Organization (WHO)
Australia Vital Registration - Deaths 1991 ICD9 as it appears in World Health Organization (WHO). WHO Mortality Database Version November 2015. Geneva, Switzerland: World Health Organization (WHO)
Australia Vital Registration - Deaths 1992 ICD9 as it appears in World Health Organization (WHO). WHO Mortality Database Version November 2015. Geneva, Switzerland: World Health Organization (WHO)

Australia Vital Registration - Deaths 1993 ICD9 as it appears in World Health Organization (WHO). WHO Mortality Database Version November 2015. Geneva, Switzerland: World Health Organization (WHO)
Australia Vital Registration - Deaths 1994 ICD9 as it appears in World Health Organization (WHO). WHO Mortality Database Version November 2015. Geneva, Switzerland: World Health Organization (WHO)
Australia Vital Registration - Deaths 1995 ICD9 as it appears in World Health Organization (WHO). WHO Mortality Database Version November 2015. Geneva, Switzerland: World Health Organization (WHO)
Australia Vital Registration - Deaths 1996 ICD9 as it appears in World Health Organization (WHO). WHO Mortality Database Version November 2015. Geneva, Switzerland: World Health Organization (WHO)
Australia Vital Registration - Deaths 1997 ICD9 as it appears in World Health Organization (WHO). WHO Mortality Database Version November 2015. Geneva, Switzerland: World Health Organization (WHO)
Australia Vital Registration - Deaths 1998 ICD10 as it appears in World Health Organization (WHO). WHO Mortality Database Version October 2017. Geneva, Switzerland: World Health Organization (WHO)
Australia Vital Registration - Deaths 1999 ICD10 as it appears in World Health Organization (WHO). WHO Mortality Database Version October 2017. Geneva, Switzerland: World Health Organization (WHO)
Australia Vital Registration - Deaths 2000 ICD10 as it appears in World Health Organization (WHO). WHO Mortality Database Version October 2017. Geneva, Switzerland: World Health Organization (WHO)
Australia Vital Registration - Deaths 2001 ICD10 as it appears in World Health Organization (WHO). WHO Mortality Database Version October 2017. Geneva, Switzerland: World Health Organization (WHO)
Australia Vital Registration - Deaths 2002 ICD10 as it appears in World Health Organization (WHO). WHO Mortality Database Version October 2017. Geneva, Switzerland: World Health Organization (WHO)
Australia Vital Registration - Deaths 2003 ICD10 as it appears in World Health Organization (WHO). WHO Mortality Database Version October 2017. Geneva, Switzerland: World Health Organization (WHO)
Australia Vital Registration - Deaths 2004 ICD10 as it appears in World Health Organization (WHO). WHO Mortality Database Version October 2017. Geneva, Switzerland: World Health Organization (WHO)
Australia Vital Registration - Deaths 2006 ICD10 as it appears in World Health Organization (WHO). WHO Mortality Database Version October 2017. Geneva, Switzerland: World Health Organization (WHO)
Australia Vital Registration - Deaths 2007 ICD10 as it appears in World Health Organization (WHO). WHO Mortality Database Version October 2017. Geneva, Switzerland: World Health Organization (WHO)
Australia Vital Registration - Deaths 2008 ICD10 as it appears in World Health Organization (WHO). WHO Mortality Database Version October 2017. Geneva, Switzerland: World Health Organization (WHO)

Australia Vital Registration - Deaths 2009 ICD10 as it appears in World Health Organization (WHO). WHO Mortality Database Version October 2017. Geneva, Switzerland: World Health Organization (WHO)
Australia Vital Registration - Deaths 2010 ICD10 as it appears in World Health Organization (WHO). WHO Mortality Database Version October 2017. Geneva, Switzerland: World Health Organization (WHO)
Australia Vital Registration - Deaths 2011 ICD10 as it appears in World Health Organization (WHO). WHO Mortality Database Version October 2017. Geneva, Switzerland: World Health Organization (WHO)
Australia Vital Registration - Deaths 2012 ICD10 as it appears in World Health Organization (WHO). WHO Mortality Database Version October 2017. Geneva, Switzerland: World Health Organization (WHO)
Australia Vital Registration - Deaths 2013 ICD10 as it appears in World Health Organization (WHO). WHO Mortality Database Version October 2017. Geneva, Switzerland: World Health Organization (WHO)
Australia Vital Registration - Deaths 2014 ICD10 as it appears in World Health Organization (WHO). WHO Mortality Database Version December 2018. Geneva, Switzerland: World Health Organization (WHO)
Australia Vital Registration - Deaths 2015 ICD10 as it appears in World Health Organization (WHO). WHO Mortality Database Version December 2018. Geneva, Switzerland: World Health Organization (WHO)
Australia Vital Registration - Deaths 2016 ICD10 as it appears in World Health Organization (WHO). WHO Mortality Database Version May 2019. Geneva, Switzerland: World Health Organization (WHO)
Han N, Wright ST, O'Connor CC, Hoy J, Ponnampalavanar S, Grotowski M, Zhao HX, Kamarulzaman A, Australian HIV Observational Database (AHOD), TREAT Asia HIV Observational Database (TAHOD). HIV and aging: insights from the Asia Pacific HIV Observational Database (APHOD). HIV Med. 2015; 16(3): 152–60
World Health Organization (WHO). WHO Mortality Database Version November 2015. Geneva, Switzerland: World Health Organization (WHO)
Kirby Institute, University of New South Wales. Australia HIV, Viral Hepatitis and Sexually Transmissible Infections Annual Surveillance Report 2016. Darlinghurst, Australia: Kirby Institute, University of New South Wales, 2016
World Health Organization (WHO). WHO Mortality Database Version October 2017. Geneva, Switzerland: World Health Organization (WHO)
Kirby Institute. HIV, viral hepatitis and sexually transmissible infections in Australia: annual surveillance report 2017. Sydney: Kirby Institute, UNSW Sydney; 2017
World Health Organization (WHO). WHO Mortality Database Version December 2018. Geneva, Switzerland: World Health Organization (WHO)
World Health Organization (WHO). WHO Mortality Database Version May 2019. Geneva, Switzerland: World Health Organization (WHO)
Fagan RL, Hanna JN, Messer RD, Brookes DL, Murphy DM. The epidemiology of invasive pneumococcal disease in children in Far North Queensland. J Paediatr Child Health. 2001; 37(6): 571-5
McIntyre PB, Macintyre CR, Gilmour R, Wang H. A population based study of the impact of corticosteroid therapy and delayed diagnosis on the

outcome of childhood pneumococcal meningitis. Arch Dis Child. 2005; 90(4): 391-6
McIntyre P, Jepson R, Leeder S, Irwig L. The outcome of childhood Haemophilus influenzae meningitis. A population based study. Med J Aust. 1993; 159(11-12): 766-72
United Nations Children's Fund (UNICEF), World Health Organization (WHO). WHO and UNICEF Reported Disease Incidence Time Series. Geneva, Switzerland: World Health Organization (WHO)
Department of Health (Australia). Australia Notifications for All Diseases by Year. Canberra, Australia: Department of Health (Australia)
Whooping Cough Case Fatality Rate Estimates as provided by the Global Burden of Disease 2010 Pertussis Expert Group. [Unpublished]
World Health Organization (WHO). WHO Distribution of Measles Cases by Country and by Month 2011-2020
Pew Research Center. The Future of the Global Muslim Population. Washington, DC, United States: Pew Research Center, 2011
Cystic Echinococcosis Endemicity Estimates identified through systematic review and personal communication, as provided by GBD 2015 expert. [Unpublished]
World Health Organization (WHO). WHO International Travel and Health, Annex 1, Countries With Risk of Yellow Fever Transmission and Countries Requiring Yellow Fever Vaccination 2017 Update. Geneva, Switzerland: World Health Organization (WHO), 2017
Australian Institute of Health and Welfare. Australian Maternal Deaths 2003-2005. Canberra, Australia: Australian Institute of Health and Welfare, 2008
Australian Institute of Health and Welfare. Australia Maternal Deaths 1997-1999. Canberra, Australia: Australian Institute of Health and Welfare, 2004
Australian Institute of Health and Welfare. Australia Maternal Deaths 2000-2002. Canberra, Australia: Australian Institute of Health and Welfare, 2006
Australian Institute of Health and Welfare. Australia Maternal Deaths 2006-2010. Canberra, Australia: Australian Institute of Health and Welfare, 2014
Australian Institute of Health and Welfare. Australia Maternal Deaths 2008-2012. Canberra, Australia: Australian Institute of Health and Welfare, 2015
Australia Vital Registration - Deaths 2017 ICD10 as it appears in World Health Organization (WHO). WHO Mortality Database Version December 2019. Geneva, Switzerland: World Health Organization (WHO)
Australian Institute of Health and Welfare. Australia Report on Maternal Deaths 1994-1996. Canberra, Australia: Australian Institute of Health and Welfare, 2001
Cliffe S, Black D, Bryant J, Sullivan E. Maternal deaths in New South Wales, Australia: a data linkage project. Aust N Z J Obstet Gynaecol. 2008; 48(3): 255-60
Department of Health (Australia). Australia Notifications of Syphilis Infection by Age Group and Sex 1991-2017. Canberra, Australia: Department of Health (Australia)
Australasian Association of Cancer Registries, Australian Institute of Health and Welfare. Australia Cancer Incidence and Mortality Books 2012. Canberra, Australia: Australian Institute of Health and Welfare, 2012

Parkin DM, International Agency for Research on Cancer, International Association of Cancer Registries. Cancer Incidence in Five Continents. Vol. I to VIII. Lyon, France, IARCPress, 2005
Ferlay J, Parkin DM, Curado MP, Bray F, Edwards B, Shin HR and Forman D. Cancer Incidence in Five Continents Time Trends, Volumes I to X and Volume XI: IARC CancerBase No. 9 [Internet]. Lyon, France: International Agency for Research on Cancer; 2010. Available from: http://ci5.iarc.fr
Australasian Association of Cancer Registries, Australian Institute of Health and Welfare. Australia Cancer Incidence and Mortality Books 2017. Canberra, Australia: Australian Institute of Health and Welfare, 2017
International Agency for Research on Cancer (IARC), International Association of Cancer Registries (IACR), World Health Organization (WHO). International Incidence of Childhood Cancer Volume III. Lyon, France: International Agency for Research on Cancer (IARC), 2017
Hong TP, Gow P, Fink M, Dev A, Roberts S, Nicoll A, Lubel J, Kronborg I, Arachchi N, Ryan M, Kemp W, Knight V, Farrugia H, Thursfield V, Desmond P, Thompson AJ, Bell S. Novel population-based study finding higher than reported hepatocellular carcinoma incidence suggests an updated approach is needed. Hepatology. 2016; 63(4): 1205-12
Bray F, Colombet M, Mery L, Piñeros M, Znaor A, Zanetti R and Ferlay J, editors (2017). Cancer Incidence in Five Continents, Vol. XI (electronic version). Lyon: International Agency for Research on Cancer. Available from: http://ci5.iarc.fr
Australia and New Zealand Dialysis and Transplant Registry (ANZDATA). Australia and New Zealand Dialysis and Transplant Registry 1990
Australia and New Zealand Dialysis and Transplant Registry (ANZDATA). Australia and New Zealand Dialysis and Transplant Registry Data 1995
Australia and New Zealand Dialysis and Transplant Registry (ANZDATA). Australia and New Zealand Dialysis and Transplant Registry 1991
Australia and New Zealand Dialysis and Transplant Registry (ANZDATA). Australia and New Zealand Dialysis and Transplant Registry 1992
Australia and New Zealand Dialysis and Transplant Registry (ANZDATA). Australia and New Zealand Dialysis and Transplant Registry 1993
Australia and New Zealand Dialysis and Transplant Registry (ANZDATA). Australia and New Zealand Dialysis and Transplant Registry 1994
Australia and New Zealand Dialysis and Transplant Registry (ANZDATA). Australia and New Zealand Dialysis and Transplant Registry 1996
Australia and New Zealand Dialysis and Transplant Registry (ANZDATA). Australia and New Zealand Dialysis and Transplant Registry 1997
Australia and New Zealand Dialysis and Transplant Registry (ANZDATA). Australia and New Zealand Dialysis and Transplant Registry 1998
Australia and New Zealand Dialysis and Transplant Registry (ANZDATA). Australia and New Zealand Dialysis and Transplant Registry 1999
Australia and New Zealand Dialysis and Transplant Registry (ANZDATA). Australia and New Zealand Dialysis and Transplant Registry 2000
Australia and New Zealand Dialysis and Transplant Registry (ANZDATA). Australia and New Zealand Dialysis and Transplant Registry 2001
Australia and New Zealand Dialysis and Transplant Registry (ANZDATA). Australia and New Zealand Dialysis and Transplant Registry 2002
Australia and New Zealand Dialysis and Transplant Registry (ANZDATA). Australia and New Zealand Dialysis and Transplant Registry 2003
Australia and New Zealand Dialysis and Transplant Registry (ANZDATA). Australia and New Zealand Dialysis and Transplant Registry 2004

Australia and New Zealand Dialysis and Transplant Registry (ANZDATA). Australia and New Zealand Dialysis and Transplant Registry 2005
Australia and New Zealand Dialysis and Transplant Registry (ANZDATA). Australia and New Zealand Dialysis and Transplant Registry 2006
Australia and New Zealand Dialysis and Transplant Registry (ANZDATA). Australia and New Zealand Dialysis and Transplant Registry 2007
Australia and New Zealand Dialysis and Transplant Registry (ANZDATA). Australia and New Zealand Dialysis and Transplant Registry 2008
Australia and New Zealand Dialysis and Transplant Registry (ANZDATA). Australia and New Zealand Dialysis and Transplant Registry 2009
Weatherall D. Sickle Cell and Thalassemias Prevalence Data, Personal Correspondence with David Weatherall. [Unpublished]
Cowan J, Kerr C. Incidence of homozygous beta-thalassaemia in New South Wales, 1961-1976. Med J Aust. 1982; 1(13): 554-6
Budtz-Olsen O, Kidson C. Absence of red cell enzyme deficiency in Australian Aborigines. Nature. 1961; 192: 765
Smith MB. The incidence of glucose-6-phosphate dehydrogenase deficiency in a population of Greek, Italian and Yugoslav origin in Australia. Med J Aust. 1976; 2(13): 485-6
Department of Health (Australia). Overseas-acquired Vectorbourne Disease Notifications in Australia: Fortnight Surveillance Reports for 2018. Canberra, Australia: Department of Health (Australia), 2018
World Health Organization (WHO). WHO Mortality Database Version February 2014. Geneva, Switzerland: World Health Organization (WHO)
World Health Organization (WHO). WHO Tuberculosis Case Notifications. Geneva, Switzerland: World Health Organization (WHO)
World Health Organization (WHO). WHO Global Project on Anti-Tuberculosis Drug Resistance Surveillance Data 1988-2015
OzFoodNet Working Group. Monitoring the incidence and causes of diseases potentially transmitted by food in Australia: annual report of the OzFoodNet Network, 2009. Commun Dis Intell Q Rep. 2010; 34(4): 396-426
Laupland KB, Schønheyder HC, Kennedy KJ, Lyytikäinen O, Valiquette L, Galbraith J, Collignon P. Salmonella enterica bacteraemia: a multi-national population-based cohort study. BMC Infect Dis. 2010; 10(1): 95

Supplementary Table 5 GBD data input sources all covariates

Citation
International Diabetes Institute (IDI). Australia Diabetes, Obesity and Lifestyle Study 1999-2000. Melbourne, Australia: International Diabetes Institute (IDI)
Australia Vital Registration - Deaths 1998 ICD10
Australia Vital Registration - Deaths 1999 ICD10
Australia Vital Registration - Deaths 2000 ICD10
Australia Vital Registration - Deaths 2002 ICD10
Australian Bureau of Statistics. Australia National Health Survey 1995. Canberra, Australia: Australian Bureau of Statistics
Commonwealth Department of Health and Family Services. Australia Survey of Contraception Use and Ease 1995. Canberra, Australia: Australian Social Science Data Archive, The Australian National University, 2004
Department of Health and Ageing (Australia), World Health Organization (WHO). Australia World Health Survey 2003. Geneva, Switzerland: World Health Organization (WHO), 2005
World Health Organization (WHO). Laos World Health Survey 2003
Centre for Health Promotion Studies, National University of Ireland, Galway, Health Promotion Unit, Department of Health and Children (Ireland). Ireland Survey of Lifestyle Attitudes and Nutrition 1998. Dublin, Ireland: Health Promotion Unit, Department of Health and Children (Ireland)
International Road Federation. World Road Statistics 1963-1999. Geneva, Switzerland: International Road Federation
P.N. Lee Statistics and Computing Ltd. International Mortality and Smoking Statistics Version 4.04. Sutton, United Kingdom: P.N. Lee Statistics and Computing Ltd, 2009
World Health Organization (WHO). WHO Global Database on Vitamin A Deficiency. Geneva, Switzerland: World Health Organization (WHO)
Australian Bureau of Statistics, Australian Institute of Family Studies, Department of Families, Housing, Community Services and Indigenous Affairs (Australia). Australia Longitudinal Study of Children, Wave 4 2009-2010. Melbourne, Australia: Australian Institute of Family Studies
Organization for Economic Co-operation and Development (OECD). OECD Health Statistics. Paris, France: Organization for Economic Co-operation and Development (OECD)
International Monetary Fund (IMF). World Economic Outlook Database. Washington, D.C., United States of America: International Monetary Fund (IMF)
British Geological Survey, Natural Environment Research Council. World Mineral Production 2007-2011. Nottingham, United Kingdom: British Geological Survey, Natural Environment Research Council, 2013

United Nations Office on Drugs and Crime (UNODC). World Drug Report 2012. Vienna, Austria: United Nations Office on Drugs and Crime (UNODC), 2012
United Nations Statistics Division (UNSD). National Accounts Main Aggregates Database. New York City, United States of America: United Nations Statistics Division (UNSD)
Australian Bureau of Statistics. Australia National Health Survey 2001. Canberra, Australia: Australian Bureau of Statistics
Australian Bureau of Statistics. Australia National Health Survey 2004-2005
Australian Bureau of Statistics. Australia National Health Survey 2007-2008. Canberra, Australia: Australian Bureau of Statistics
World Health Organization (WHO). WHO Global Health Expenditure Database. Geneva, Switzerland: World Health Organization (WHO)
World Health Organization (WHO). WHO DengueNet. Geneva, Switzerland: World Health Organization (WHO)
World Health Organization (WHO). Global Status Report on Road Safety 2009. Geneva, Switzerland: World Health Organization (WHO), 2009
Australia Vital Registration - Deaths 2007 ICD10
Australia Vital Registration - Deaths 2008 ICD10
Australia Vital Registration - Deaths 2009 ICD10
Weatherall D. Sickle Cell and Thalassemias Prevalence Data, Personal Correspondence with David Weatherall. [Unpublished]
Australian Bureau of Statistics. Australia Vital Registration - Deaths 2005
Cowan J, Kerr C. Incidence of homozygous beta-thalassaemia in New South Wales, 1961-1976. Med J Aust. 1982; 1(13): 554-6
Gliksman MD, Dwyer T, Wlodarczyk J, Pierce JP. Cigarette smoking in Australian schoolchildren. Med J Aust. 1989; 150(2): 81–4
World Health Organization (WHO), WHO MONICA Project Investigators 2000. MONICA Population Survey Data Book 1979-1997. Geneva, Switzerland: World Health Organization (WHO), 2000
Australian Institute of Health and Welfare, Department of Health and Ageing (Australia), Roy Morgan Research, Social Research Centre (Australia). Australia National Drug Strategy Household Survey 2007
Australian Institute of Health and Welfare. Australia National Drug Strategy Household Survey 2004
Australian Institute of Health and Welfare. Australia National Drug Strategy Household Survey 2001
Australian Institute of Health and Welfare, Roy Morgan Research. Australia National Drug Strategy Household Survey 1998
Australian Institute of Health and Welfare, National Heart Foundation (Australia). Australia Risk Factor Prevalence Survey 1989. Acton, Australia: Australian Data Archive
Busselton Population Medical Research Institute (Australia). Australia - Busselton Health Study 1981
Simmons D, McKenzie A, Eaton S, Shaw J, Zimmet P. Prevalence of diabetes in rural Victoria. Diabetes Res Clin Pract. 2005; 70(3): 287-90

Australian Bureau of Statistics. Australia National Health Survey 2011-2013
International Diabetes Institute (IDI). Australia Diabetes, Obesity and Lifestyle Study 2004-2005
Ackerman IN, Osborne RH. Obesity and increased burden of hip and knee joint disease in Australia: results from a national survey. BMC Musculoskelet Disord. 2012; 13(1): 254
Andrews, Gary R., and George C. Myers. Australian [Adelaide] Longitudinal Study of Aging, Waves 1-5 [1992-1997]. ICPSR06707-v3. Ann Arbor, MI: Inter-university Consortium for Political and Social Research [distributor], 2000. doi:10.3886/ICPSR06707.v3
Australian Bureau of Statistics. Australia National Survey of Mental Health and Wellbeing 2007. Canberra, Australia: Australian Bureau of Statistics, 2007
Swift W, Hall W, Teesson M. Cannabis use and dependence among Australian adults: results from the National Survey of Mental Health and Wellbeing. Addiction. 2001; 96(5): 737-48
Coffey C, Carlin JB, Degenhardt L, Lynskey M, Sanci L, Patton GC. Cannabis dependence in young adults: an Australian population study. Addiction. 2002; 97(2): 187-94
Swift W, Coffey C, Carlin JB, Degenhardt L, Patton GC. Adolescent cannabis users at 24 years: trajectories to regular weekly use and dependence in young adulthood. Addiction. 2008; 103(8): 1361-70
Patton GC, Coffey C, Carlin JB, Degenhardt L, Lynskey M, Hall W. Cannabis use and mental health in young people: cohort study. BMJ. 2002; 325(7374): 1195-8
Bartu A, Freeman NC, Gawthorne GS, Codde JP, Holman CDJ. Mortality in a cohort of opiate and amphetamine users in Perth, Western Australia. Addiction. 2004; 99(1): 53-60
Guttinger R, Pascoe E, Rossi E, Kotecha R, Willis F. The Fremantle Lead Study Part 2. J Paediatr Child Health. 2008; 44(12): 722-6
Kelsall LM, de Gooyer TE, Carey M, Vaughan L, Ansari Z. Blood lead levels in the adult Victorian population: results from the Victorian Health Monitor. Aust N Z J Public Health. 2013; 37(3): 233-7
Mira M, Bawden-Smith J, Causer J, Alperstein G, Karr M, Snitch P, Waller G, Fett MJ. Blood lead concentrations of preschool children in central and southern Sydney. Med J Aust. 1996; 164(7): 399-402
Williams L, Jacka F, Pasco J, Henry M, Dodd S, Nicholson G, Kotowicz M, Berk M. The prevalence of mood and anxiety disorders in Australian women. Australas Psychiatry. 2010; 18(3): 250-5
Cancer Council Victoria (Australia), Department of Health and Ageing (Australia). Australia Secondary Students' Alcohol and Drug Survey 2005
Janus ED, Laatikainen T, Dunbar JA, Kilkkinen A, Bunker SJ, Philpot B, Tideman PA, Tirimacco R, Heistaro S. Overweight, obesity and metabolic syndrome in rural southeastern Australia. Med J Aust. 2007; 187(3): 147-52
Coomber K, Toumbourou JW, Miller P, Staiger PK, Hemphill SA, Catalano RF. Rural adolescent alcohol, tobacco, and illicit drug use: a comparison of students in Victoria, Australia, and Washington State, United States. J Rural Health. 2011; 27(4): 409-15

Australian Bureau of Statistics, Department of Health and Family Services (Australia). Australia National Nutrition Survey 1995-1996. Canberra, Australia: Australian Bureau of Statistics
Hall W, Teesson M, Lynskey M, Degenhardt L. The 12-month prevalence of substance use and ICD-10 substance use disorders in Australian adults: findings from the National Survey of Mental Health and Well-Being. Addiction. 1999; 94(10): 1541-50
Lynskey M, White V, Hill D, Letcher T, Hall W. Prevalence of illicit drug use among youth: results from the Australian School Students' Alcohol and Drugs Survey. Aust N Z J Public Health. 1999; 23(5): 519-24
Patton GC HM, Rosier MJ, Carlin JB, Caust J, Bowes G. Patterns of common drug use in teenagers. Aust J Public Health. 1995; 19(4): 393-9
Hawthorne G, Goldney R, Taylor AW. Depression prevalence: is it really increasing?. Aust N Z J Psychiatry. 2008; 42(7): 606-16
Jorm AF, Henderson AS, Kay DWK, Jacomb PA. Mortality in relation to dementia, depression and social integration in an elderly community sample. Int J Geriatr Psychiatry. 1991; 6(1): 11-May
Wilhelm K, Mitchell P, Slade T, Brownhill S, Andrews G. Prevalence and correlates of DSM-IV major depression in an Australian national survey. J Affect Disord. 2003; 75(2): 155-62
Goldney RD, Eckert KA, Hawthorne G, Taylor AW. Changes in the prevalence of major depression in an Australian community sample between 1998 and 2008. Aust N Z J Psychiatry. 2010; 44(10): 901-10
Gartner CE, Barendregt JJ, Hall WD. Predicting the future prevalence of cigarette smoking in Australia: how low can we go and by when?. Tob Control. 2009; 18(3): 183-9
White V, Hill D, Siahpush M, Bobevski I. How has the prevalence of cigarette smoking changed among Australian adults? Trends in smoking prevalence between 1980 and 1932. Tob Control. 2003; 12(Suppl 2): ii67-ii74
Australian Institute of Health and Welfare, Roy Morgan Research. Australia National Drug Strategy Household Survey 2010
National Drug and Alcohol Research Centre, University of New South Wales. Australia Estimating the Number of Current Regular Heroin Users in NSW and Australia 1997-2002. Randwick, Australia: National Drug and Alcohol Research Centre, University of New South Wales, 2004
Degenhardt L, Randall D, Hall W, Butler T, Law M, Burns L. Mortality among persons receiving opioid pharmacotherapy in New South Wales, Australia, 1985-2006: risk factors for elevated mortality and estimated mortality reductions of a statewide programme across 20 years
VanNewkirk MR, Weih L, McCarty CA, Taylor HR. Cause-specific prevalence of bilateral visual impairment in Victoria, Australia: the Visual Impairment Project. Ophthalmology. 2001; 108(5): 960-7
Australian Congenital Malformation Monitoring System Data 1993-1997 - WHO as it appears in European Surveillance of Congenital Anomalies (EUROCAT), International Centre on Birth Defects, World Health Organization (WHO). World Atlas of Birth Defects. 2nd ed. Geneva, Switzerland: World Health Organization (WHO), 2003
Australian Institute of Health and Welfare. Australian Maternal Deaths 2003-2005. Canberra, Australia: Australian Institute of Health and Welfare, 2008

Australia - Victoria Birth Defects Registry Data 2001 - ICBDMS as it appears in International Clearinghouse for Birth Defects Monitoring Systems. International Clearinghouse for Birth Defects Monitoring Systems Annual Report 2003. Rome, Italy: International Centre on Birth Defects
Sutton DL, Lyle DM, Pierce JP. Incidence and prevalence of insulin-dependent diabetes mellitus in the zero- to 19-years' age-group in Sydney. Med J Aust. 1989; 151(3): 140-6
Byrne A. Nine-year follow-up of 86 consecutive patients treated with methadone in general practice, Sydney, Australia. Drug Alcohol Rev. 2000; 19(2): 153-8
Digiusto E, Shakeshaft A, Ritter A, O'Brien S, Mattick RP, NEPOD Research Group. Serious adverse events in the Australian National Evaluation of Pharmacotherapies for Opioid Dependence (NEPOD). Addiction. 2004; 99(4): 450-60
Hall WD, Ross JE, Lynskey MT, Law MG, Degenhardt LJ. How many dependent heroin users are there in Australia?. Med J Aust. 2000; 173(10): 528-31
Law MG, Lynskey M, Ross J, Hall W. Back-projection estimates of the number of dependent heroin users in Australia. Addiction. 2001; 96(3): 433-43
Tait RJ, Ngo HTT, Hulse GK. Mortality in heroin users 3 years after naltrexone implant or methadone maintenance treatment. J Subst Abuse Treat. 2008; 35(2): 116-24
Teesson M, Baillie A, Lynskey M, Manor B, Degenhardt L. Substance use, dependence and treatment seeking in the United States and Australia: a cross-national comparison. Drug Alcohol Depend. 2006; 81(2): 149-55
Teesson M, Mills K, Ross J, DarkeS, Williamson A, Havard A. The impact of treatment on 3 years' outcome for heroin dependence: findings from the Australian Treatment Outcome Study (ATOS). Addiction. 2008; 103(1): 80-88
Australasian Association of Cancer Registries, Australian Institute of Health and Welfare. Australia Cancer Incidence and Mortality Books 2012. Canberra, Australia: Australian Institute of Health and Welfare, 2012
Sawyer M, Miller-Lewis L, Clark J. The Mental Health of 13-17 Year-Olds in Australia: Findings from the National Survey of Mental Health and Well-Being. J Youth Adolescence. 2007; 36(2): 185-94
Budtz-Olsen O, Kidson C. Absence of red cell enzyme deficiency in Australian Aborigines. Nature. 1961; 192: 765
Smith MB. The incidence of glucose-6-phosphate dehydrogenase deficiency in a population of Greek, Italian and Yugoslav origin in Australia. Med J Aust. 1976; 2(13): 485-6
Australia - Victoria Birth Defects Registry Data 2002 - ICBDMS as it appears in International Clearinghouse for Birth Defects Monitoring Systems. International Clearinghouse for Birth Defects Monitoring Systems Annual Report 2004. Rome, Italy: International Centre on Birth Defects, 2006
Australia - Victoria Birth Defects Registry Data 2003 - ICBDSR as it appears in International Clearinghouse for Birth Defects Surveillance and Research. International Clearinghouse for Birth Defects Surveillance and Research Annual Report 2005. Rome, Italy: International Clearinghouse for Birth Defects Surveillance and Research, 2007
Australia - Victoria Birth Defects Registry Data 2004 - ICBDSR as it appears in International Clearinghouse for Birth Defects Surveillance and Research. International Clearinghouse for Birth Defects Surveillance and Research Annual Report 2006. Rome, Italy: International Clearinghouse for Birth

Defects Surveillance and Research, 2007
Australia - Victoria Birth Defects Registry Data 2005 - ICBDSR as it appears in International Clearinghouse for Birth Defects Surveillance and Research. International Clearinghouse for Birth Defects Surveillance and Research Annual Report 2007. Rome, Italy: International Clearinghouse for Birth Defects Surveillance and Research, 2008
Australia - Victoria Birth Defects Registry Data 2007 - ICBDSR as it appears in International Clearinghouse for Birth Defects Surveillance and Research. International Clearinghouse for Birth Defects Surveillance and Research Annual Report 2009. Rome, Italy: International Clearinghouse for Birth Defects Surveillance and Research
Australia - Victoria Birth Defects Registry Data 2008 - ICBDSR as it appears in International Clearinghouse for Birth Defects Surveillance and Research. International Clearinghouse for Birth Defects Surveillance and Research Annual Report 2010. Rome, Italy: International Clearinghouse for Birth Defects Surveillance and Research, 2011
Australia - Western Australian Register of Developmental Anomalies Data 2009 - ICBDSR as it appears in International Clearinghouse for Birth Defects Surveillance and Research. International Clearinghouse for Birth Defects Surveillance and Research Annual Report 2011. Rome, Italy: International Clearinghouse for Birth Defects Surveillance and Research, 2012
Australia - Western Australian Register of Developmental Anomalies Data 2010 - ICBDSR as it appears in International Clearinghouse for Birth Defects Surveillance and Research. International Clearinghouse for Birth Defects Surveillance and Research Annual Report 2012. Rome, Italy: International Clearinghouse for Birth Defects Surveillance and Research, 2013
Australia - South Australia Cancer Registry 1977 - CI5 as it appears in Parkin DM, International Agency for Research on Cancer, International Association of Cancer Registries. Cancer Incidence in Five Continents. Vol. I to VIII. Lyon, France, IARCPress, 2005
Australian Congenital Malformation Monitoring System Data 1985 - ICBDMS as it appears in International Clearinghouse for Birth Defects Monitoring Systems. Congenital Malformations Worldwide: A Report from the International Clearinghouse for Birth Defects Monitoring Systems. Amsterdam, Netherlands: Elsevier, 1991
Bauman A, Bull F, Chey T, Craig CL, Ainsworth BE, Sallis JF, Bowles HR, Hagstromer M, Sjostrom M, Pratt M, IPS Group. The International Prevalence Study on Physical Activity: results from 20 countries. Int J Behav Nutr Phys Act. 2009; 21
Markwick A, Vaughan L, Ansari Z. Opposing socioeconomic gradients in overweight and obese adults. Aust N Z J Public Health. 2013; 37(1): 32-8
Davies CA, Vandelanotte C, Duncan MJ, van Uffelen JGZ. Associations of physical activity and screen-time on health related quality of life in adults. Prev Med. 2012; 55(1): 46-9
Australian Institute of Health and Welfare. Australia National Survey of Lead in Children 1995
Government of Western Australia. Response of the Western Australian Government to the Western Australian Legislative Assembly Education and Health Standing Committee in Relation to the Cause and Extent of Lead Pollution in the Esperance Area 2007. Perth, Western Australia: Government of Western Australia, 2007

Henry MJ, Pasco JA, Korn S, Gibson JE, Kotowicz MA, Nicholson GC. Bone mineral density reference ranges for Australian men: Geelong Osteoporosis Study. Osteoporos Int. 2010; 21(6): 909-17

Hopper JL, Balderas A, Mathews JD. Analysis of variation in blood lead levels in Melbourne families. Med J Aust. 1982; 2(12): 573-6

Bloom H, Lewis IC, Kishimoto R. A study of lead concentrations in blood of children (and some adults) of Southern Tasmania. Med J Aust. 1974; 2(8): 275-9

De Silva PE, Donnan MB. Blood lead levels in Victorian children. Med J Aust. 1980; 2(6): 315-8

Rathus M, Latham S, Golding G, Rowan C. Blood lead levels in Queensland children. Med J Aust. 1982; 2(4): 183-5

Threlfall T, Kent N, Garcia-Webb P, Byrnes E, Psaila-Savona P. Blood lead levels in children in Perth, Western Australia. Aust J Public Health. 1993; 17(4): 379-81

Cowie C, Black D, Fraser I. Blood lead levels in preschool children in eastern Sydney. Aust N Z J Public Health. 1997; 21(7): 755-61

Chiaradia M, Gulson BL, MacDonald K. Contamination of houses by workers occupationally exposed in a lead-zinc-copper mine and impact on blood lead concentrations in the families. Occup Environ Med. 1997; 54(2): 117-24

Devine A, Dick IM, Islam AF, Dhaliwal SS, Prince RL. Protein consumption is an important predictor of lower limb bone mass in elderly women. Am J Clin Nutr. 2005; 81(6): 1423-8

Henry MJ, Pasco JA, Nicholson GC, Seeman E, Kotowicz MA. Prevalence of osteoporosis in Australian women: Geelong Osteoporosis Study. J Clin Densitom. 2000; 3(3): 261-8

Jones G, Nguyen T, Sambrook P, Kelly PJ, Eisman JA. Progressive loss of bone in the femoral neck in elderly people: longitudinal findings from the Dubbo osteoporosis epidemiology study. BMJ. 1994; 309(6956): 691-5

Flicker L, Green R, Kaymakci B, Lichtenstein M, Buirski G, Wark JD. Do Australian women have greater spinal bone density than North American women?. Osteoporos Int. 1995; 5(1): 63-5

Slade T, Johnston A, Oakley Browne MA, Andrews G, Whiteford H. 2007 National Survey of Mental Health and Wellbeing: methods and key findings. Aust N Z J Psychiatry. 2009; 43(7): 594-605

Ferlay J, Parkin DM, Curado MP, Bray F, Edwards B, Shin HR and Forman D. Cancer Incidence in Five Continents Time Trends, Volumes I to X and Volume XI: IARC CancerBase No. 9 [Internet]. Lyon, France: International Agency for Research on Cancer; 2010. Available from: http://ci5.iarc.fr

Australia - South Australia Cancer Registry 1981 - CI5 as it appears in Ferlay J, Parkin DM, Curado MP, Bray F, Edwards B, Shin HR and Forman D. Cancer Incidence in Five Continents Time Trends, Volumes I to X and Volume XI: IARC CancerBase No. 9 [Internet]. Lyon, France: International Agency for Research on Cancer; 2010. Available from: http://ci5.iarc.fr

Australia - South Australia Cancer Registry 1980 - CI5 as it appears in Ferlay J, Parkin DM, Curado MP, Bray F, Edwards B, Shin HR and Forman D. Cancer Incidence in Five Continents Time Trends, Volumes I to X and Volume XI: IARC CancerBase No. 9 [Internet]. Lyon, France: International Agency for Research on Cancer; 2010. Available from: http://ci5.iarc.fr

Australia - Tasmania Cancer Registry 1981 - CI5 as it appears in Ferlay J, Parkin DM, Curado MP, Bray F, Edwards B, Shin HR and Forman D. Cancer Incidence in Five Continents Time Trends, Volumes I to X and Volume XI: IARC CancerBase No. 9 [Internet]. Lyon, France: International Agency for Research on Cancer; 2010. Available from: http://ci5.iarc.fr
Australia - Tasmania Cancer Registry 1980 - CI5 as it appears in Ferlay J, Parkin DM, Curado MP, Bray F, Edwards B, Shin HR and Forman D. Cancer Incidence in Five Continents Time Trends, Volumes I to X and Volume XI: IARC CancerBase No. 9 [Internet]. Lyon, France: International Agency for Research on Cancer; 2010. Available from: http://ci5.iarc.fr
Australian Institute of Health and Welfare, National Heart Foundation (Australia). Australia Risk Factor Prevalence Survey 1994
Commonwealth Scientific and Industrial Research Organisation (CSIRO) National Research Preventative Health Flagship (Australia), Department of Health and Ageing (Australia), I-view Pty Ltd, University of South Australia. Australia National Children's Nutrition and Physical Activity Survey 2007
Johnson H, Ollus N, Nevala S. Violence Against Women: An International Perspective. New York City, United States: Springer; 2008
Bolisetty S, Daftary A, Ewald D, Knight B, Wheaton G. Congenital heart defects in Central Australia. Med J Aust. 2004; 180(12): 614-7
Fitzgerald P, Leonard H, Pikora TJ, Bourke J, Hammond G. Hospital admissions in children with down syndrome: experience of a population-based cohort followed from birth. PLoS One. 2013; 8(8): e70401
Malone Q. Mortality and survival of the Down's syndrome population in Western Australia. J Ment Defic Res. 1988; 32 (Pt 1): 59-65
World Health Organization Regional Office for the Western Pacific (WPRO-WHO). Number of Reported Cases of Dengue Fever and Dengue Hemorrhagic Fever in the Western Pacific Region, by Country 2000. Manila, Philippines: World Health Organization Regional Office for the Western Pacific (WPRO-WHO)
World Health Organization Regional Office for the Western Pacific (WPRO-WHO). Number of Reported Cases of Dengue Fever and Dengue Hemorrhagic Fever in the Western Pacific Region, by Country 2001. Manila, Philippines: World Health Organization Regional Office for the Western Pacific (WPRO-WHO)
World Health Organization Regional Office for the Western Pacific (WPRO-WHO). Number of Reported Cases of Dengue Fever and Dengue Hemorrhagic Fever in the Western Pacific Region, by Country 2002. Manila, Philippines: World Health Organization Regional Office for the Western Pacific (WPRO-WHO)
World Health Organization Regional Office for the Western Pacific (WPRO-WHO). Number of Reported Cases of Dengue Fever and Dengue Hemorrhagic Fever in the Western Pacific Region, by Country 2003. Manila, Philippines: World Health Organization Regional Office for the Western Pacific (WPRO-WHO)
World Health Organization Regional Office for the Western Pacific (WPRO-WHO). Number of Reported Cases of Dengue Fever and Dengue Hemorrhagic Fever in the Western Pacific Region, by Country 2004. Manila, Philippines: World Health Organization Regional Office for the Western Pacific (WPRO-WHO)
World Health Organization Regional Office for the Western Pacific (WPRO-WHO). Number of Reported Cases of Dengue Fever and Dengue

Hemorrhagic Fever in the Western Pacific Region, by Country 2005. Manila, Philippines: World Health Organization Regional Office for the Western Pacific (WPRO-WHO)
World Health Organization Regional Office for the Western Pacific (WPRO-WHO). Number of Reported Cases of Dengue Fever and Dengue Hemorrhagic Fever in the Western Pacific Region, by Country 2006. Manila, Philippines: World Health Organization Regional Office for the Western Pacific (WPRO-WHO)
World Health Organization Regional Office for the Western Pacific (WPRO-WHO). Number of Reported Cases of Dengue Fever and Dengue Hemorrhagic Fever in the Western Pacific Region, by Country 2007. Manila, Philippines: World Health Organization Regional Office for the Western Pacific (WPRO-WHO)
World Health Organization Regional Office for the Western Pacific (WPRO-WHO). Number of Reported Cases of Dengue Fever and Dengue Hemorrhagic Fever in the Western Pacific Region, by Country 2008. Manila, Philippines: World Health Organization Regional Office for the Western Pacific (WPRO-WHO)
World Health Organization Regional Office for the Western Pacific (WPRO-WHO). Number of Reported Cases of Dengue Fever and Dengue Hemorrhagic Fever in the Western Pacific Region, by Country 2009. Manila, Philippines: World Health Organization Regional Office for the Western Pacific (WPRO-WHO)
World Health Organization Regional Office for the Western Pacific (WPRO-WHO). Number of Reported Cases of Dengue Fever and Dengue Hemorrhagic Fever in the Western Pacific Region, by Country 2010. Manila, Philippines: World Health Organization Regional Office for the Western Pacific (WPRO-WHO)
Australian Institute of Health and Welfare, Hunter Valley Research Foundation. Australia National Physical Activity Survey 1999
New South Wales Department of Health. Australia - New South Wales Population Health Survey 2002
New South Wales Department of Health. Australia - New South Wales Population Health Survey 2003
New South Wales Department of Health. Australia - New South Wales Population Health Survey 2004
New South Wales Department of Health. Australia - New South Wales Population Health Survey 2005
New South Wales Department of Health. Australia - New South Wales Population Health Survey 2006
New South Wales Department of Health. Australia - New South Wales Population Health Survey 2007
New South Wales Department of Health. Australia - New South Wales Population Health Survey 2008
New South Wales Department of Health. Australia - New South Wales Population Health Survey 2009
New South Wales Department of Health. Australia - New South Wales Population Health Survey 2010
New South Wales Department of Health. Australia - New South Wales Population Health Survey 2011
New South Wales Department of Health. Australia - New South Wales Population Health Survey 2012

Australia - Blue Mountains Eye Study 1992-1994 as it appears in Bourne R, Global Burden of Disease Vision Loss Expert Group. Vision Loss Database - Survey Data on Vision Loss by Severity and Etiology. [Unpublished]
Magarey AM, Daniels LA, Boulton TJ. Prevalence of overweight and obesity in Australian children and adolescents: reassessment of 1985 and 1995 data against new standard international definitions. Med J Aust. 2001; 174(11): 561-4
Dhaliwal SS, Howat P, Bejoy T, Welborn TA. Self-reported weight and height for evaluating obesity control programs. Am J Health Behav. 2010; 34(4): 489-99
Patton GC, Coffey C, Carlin JB, Sawyer SM, Williams J, Olsson CA, Wake M. Overweight and obesity between adolescence and young adulthood: a 10-year prospective cohort study. J Adolesc Health. 2011; 48(3): 275-80
Vaska V, Volkmer R. Increasing prevalence of obesity in South Australian 4-year-olds: 1995-2002. J Paediatr Child Health. 2004; 40(7): 353-5
Flicker L, McCaul KA, Hankey GJ, Jamrozik K, Brown WJ, Byles JE, Almeida OP. Body mass index and survival in men and women aged 70 to 75. J Am Geriatr Soc. 2010; 58(2): 234-41
Booth ML, Wake M, Armstrong T, Chey T, Hesketh K, Mathur S. The epidemiology of overweight and obesity among Australian children and adolescents, 1995-97. Aust N Z J Public Health. 2001; 25(2): 162-9
Hesketh K, Wake M, Waters E, Carlin J, Crawford D. Stability of body mass index in Australian children: a prospective cohort study across the middle childhood years. Public Health Nutr. 2004; 7(2): 303-9
Siu J, Giskes K, Shaw J, Turrell G. Perceived weight status may contribute to education inequalities in five-year weight change among mid-aged women. Aust N Z J Public Health. 2011; 35(3): 284-91
Renzaho A, Wooden M, Houng B. Associations between body mass index and health-related quality of life among Australian adults. Qual Life Res. 2010; 19(4): 515-20
Sanigorski AM, Bell AC, Kremer PJ, Swinburn BA. High childhood obesity in an Australian population. Obesity (Silver Spring). 2007; 15(8): 1908-12
Booth ML, Dobbins T, Okely AD, Denney-Wilson E, Hardy LL. Trends in the prevalence of overweight and obesity among young Australians, 1985, 1997, and 2004. Obesity (Silver Spring). 2007; 15(5): 1089-95
Shi Z, Taylor AW, Gill TK, Tuckerman J, Adams R, Martin J. Short sleep duration and obesity among Australian children. BMC Public Health. 2010; 609
Booth ML, Okely AD, Denney-Wilson E. Validation and application of a novel method of measuring non-response bias in school-based surveys of paediatric overweight and obesity. Int J Pediatr Obes. 2011; 6(2-2): e87-93
Kortt MA, Dollery B. Association between body mass index and health-related quality of life among an Australian sample. Clin Ther. 2011; 33(10): 1466-74
Holden CA, McLachlan RI, Pitts M, Cumming R, Wittert G, Ehsani JP, de Kretser DM, Handelsman DJ. Determinants of male reproductive health disorders: the Men in Australia Telephone Survey (MATeS). BMC Public Health. 2010; 96
Goldney RD, Dunn KI, Air TM, Dal Grande E, Taylor AW. Relationships between body mass index, mental health, and suicidal ideation: population

perspective using two methods. Aust N Z J Psychiatry. 2009; 43(7): 652-8
O'Dea JA, Amy NK. Perceived and desired weight, weight related eating and exercising behaviours, and advice received from parents among thin, overweight, obese or normal weight Australian children and adolescents. Int J Behav Nutr Phys Act. 2011; 68
Khambalia A, Hardy LL, Bauman A. Accuracy of weight perception, life-style behaviours and psychological distress among overweight and obese adolescents. J Paediatr Child Health. 2012; 48(3): 220-7
ISSP Research Group (2009): International Social Survey Programme: Health and Health Care - ISSP 2011. GESIS Data Archive, Cologne. ZA5800 Data file version 3.0.0, doi:10.4232/1.12252
ISSP Research Group (2009): International Social Survey Programme: Leisure Time and Sports - ISSP 2007. GESIS Data Archive, Cologne. ZA4850 Data file version 2.0.0, doi:10.4231/1.10079
Rangan AM, Aitkin I, Blight GD, Binns CW. Factors affecting iron status in 15-30 year old female students. Asia Pac J Clin Nutr. 1997; 6(4): 291-5
International Labour Organization (ILO). International Labour Organization Database (ILOSTAT) - Employment by Sex and Economic Activity. International Labour Organization (ILO)
International Labour Organization (ILO). International Labour Organization Database (ILOSTAT) - Employment by Sex and Occupation. International Labour Organization (ILO)
Simons LA, Friedlander Y, McCallum J, Simons J, Powell I, Heller R, Berry G. The Dubbo study of the health of elderly: correlates of coronary heart disease at study entry. J Am Geriatr Soc. 1991; 39(6): 584-90
Magliano DJ, Barr EL, Zimmet PZ, Cameron AJ, Dunstan DW, Colagiuri S, Jolley D, Owen N, Phillips P, Tapp RJ, Welborn TA, Shaw JE. Glucose indices, health behaviors, and incidence of diabetes in Australia: the Australian Diabetes, Obesity and Lifestyle Study. Diabetes Care. 2008; 31(2): 267-72
Mèantyselkèa P, Miettola J, Niskanen L, Kumpusalo E. Glucose regulation and chronic pain at multiple sites. Rheumatology (Oxford). 2008; 47(8): 1235-8
Simons LA, Simons J, McCallum J, Friedlander Y. Impact of smoking, diabetes and hypertension on survival time in the elderly: the Dubbo Study. Med J Aust. 2005; 182(5): 219-23
Razali K, Thein HH, Bell J, Cooper-Stanbury M, Dolan K, Dore G, George J, Kaldor J, Karvelas M, Li J, Maher L, McGregor S, Hellard M, Poeder F, Quaine J, Stewart K, Tyrrell H, Weltman M, Westcott O, Wodak A, Law M. Modelling the hepatitis C virus epidemic in Australia. Drug Alcohol Depend. 2007; 91(2-3): 228-35
Quan HL, Blizzard CL, Venn AJ, Thuy AB, Luc PH, Sharman JE. Blood pressure and body mass index: a comparison of the associations in the Caucasian and Asian populations. Hypertens Res. 2012; 35(5): 523-30
Janus ED, Tideman PA, Dunbar JA, Kilkkinen A, Bunker SJ, Philpot B, Tirimacco R, Mc Namara K, Heistaro S, Laatikainen T. Dyslipidaemia in rural Australia: prevalence, awareness, and adherence to treatment guidelines in the Greater Green Triangle Risk Factor Study. Med J Aust. 2010; 192(3): 127-32

Amin J, Gidding H, Gilbert G, Backhouse J, Kaldor J, Dore G, Burgess M. Hepatitis C prevalence - a nationwide survey. Commun Dis Intell. 2004; 28(4): 517-21
Ashwell MJ, Cossart YE. An autopsy survey of hepatitis B in Sydney. Pathology. 1995; 27(1): 43-7
Campbell DH, Plant AJ, Sargent JW, Mock PA, Barrett ER, Archer KH. Hepatitis B infection of children in a mixed-race community in western New South Wales. Med J Aust. 1991; 154(4): 253-6
Gidding HF, Warlow M, MacIntyre CR, Backhouse J, Gilbert GL, Quinn HE, McIntyre PB. The impact of a new universal infant and school-based adolescent hepatitis B vaccination program in Australia. Vaccine. 2007; 25(51): 8637-41
Moore DJ, Bucens MR, Holman CD, Ott AK, Wells JI. Prenatal screening for markers of hepatitis B in aboriginal mothers resident in non-metropolitan Western Australia. Med J Aust. 1987; 147(11-12): 557-8
O'Sullivan BG, Gidding HF, Law M, Kaldor JM, Gilbert GL, Dore GJ. Estimates of chronic hepatitis B virus infection in Australia, 2000. Aust N Z J Public Health. 2004; 28(3): 212-6
Patterson F, Bumak J, Batey R. Changing prevalence of hepatitis B virus in urbanized Australian Aborigines. J Gastroenterol Hepatol. 1993; 8(5): 410-3
Tawk HM, Vickery K, Bisset L, Selby W, Cossart YE, Infection in Endoscopy Study Group. The impact of hepatitis B vaccination in a Western country: recall of vaccination and serological status in Australian adults. Vaccine. 2006; 24(8): 1095-106
Langroo MK, Wise KN, Duggleby JC, Kotler LH. A nationwide survey of 222Rn and gamma radiation levels in Australian homes. Health Phys. 1991; 61(6): 753-61
Pew Research Center. The Future of the Global Muslim Population. Washington, DC, United States: Pew Research Center, 2011
Huggins CE, O'Reilly S, Brinkman M, Hodge A, Giles GG, English DR, Nowson CA. Relationship of urinary sodium and sodium-to-potassium ratio to blood pressure in older adults in Australia. Med J Aust. 2011; 195(3): 128-32
Australian Bureau of Statistics. Australia National Health Survey 1989-1990
Baker IDI Heart and Diabetes Institute, International Diabetes Institute (IDI), Monash University (Australia), University of Melbourne. Australia Diabetes, Obesity and Lifestyle Study 2011-2012
Australian Institute of Health and Welfare, Roy Morgan Research. Australia National Drug Strategy Household Survey 2013
Aarhus University, Addiction Switzerland Research Institute, Alcohol Research Group, Public Health Institute, Centre for Addiction and Mental Health (Canada), Centre for Alcohol Policy Research, Turning Point Alcohol and Drug Centre (Australia), Kettil Bruun Society for Social and Epidemiological Research on Alcohol, University of North Dakota. Australia Gender, Alcohol and Culture: An International Study (GENACIS) 1994
Aarhus University, Addiction Switzerland Research Institute, Alcohol Research Group, Public Health Institute, Centre for Addiction and Mental Health (Canada), Centre for Alcohol Policy Research, Turning Point Alcohol and Drug Centre (Australia), Kettil Bruun Society for Social and Epidemiological Research on Alcohol, University of North Dakota. Australia - Victoria Gender, Alcohol and Culture: An International Study (GENACIS) 2007
Australian Bureau of Statistics. Australia Population and Housing Census 2011

TQA Research, World Health Organization (WHO). Australia WHO Multi-country Survey Study on Health and Health System Responsiveness 2000-2001. Geneva, Switzerland: World Health Organization (WHO)
Han N, Wright ST, O'Connor CC, Hoy J, Ponnampalavanar S, Grotowski M, Zhao HX, Kamarulzaman A, Australian HIV Observational Database (AHOD), TREAT Asia HIV Observational Database (TAHOD). HIV and aging: insights from the Asia Pacific HIV Observational Database (APHOD). HIV Med. 2015; 16(3): 152–60
National Centre for Immunisation Research and Surveillance of Vaccine Preventable Diseases (NCRIS) (Australia). Australia Immunisation Coverage Annual Report 2007. Commun Dis Intell. 2009; 33(2): 170-87
National Centre for Immunisation Research and Surveillance of Vaccine Preventable Diseases (NCRIS) (Australia). Australia Immunisation Coverage Annual Report 2008. Commun Dis Intell. 2010; 34(3): 241-58
National Centre for Immunisation Research and Surveillance of Vaccine Preventable Diseases (NCRIS) (Australia). Australia Immunisation Coverage Annual Report 2009. Commun Dis Intell. 2011; 35(2): 132-48
National Centre for Immunisation Research and Surveillance of Vaccine Preventable Diseases (NCRIS) (Australia). Australia Immunisation Coverage Annual Report 2010. Commun Dis Intell. 2013; 37(1): E21-39
National Centre for Immunisation Research and Surveillance of Vaccine Preventable Diseases (NCRIS) (Australia). Australia Immunisation Coverage Annual Report 2011. Commun Dis Intell. 2013; 37(4): E291-312
National Centre for Immunisation Research and Surveillance of Vaccine Preventable Diseases (NCRIS) (Australia). Australia Immunisation Coverage Annual Report 2012. Commun Dis Intell. 2014; 38(3): E208-31
Department of Health (Australia). Australia Notifications of Syphilis Infection by Age Group and Sex 1991-2017. Canberra, Australia: Department of Health (Australia)
World Health Organization Regional Office for the Western Pacific (WPRO-WHO). WPRO-WHO Health Information and Intelligence Platform - Antenatal Care Coverage - At Least 1 Visit. Manila, Philippines: World Health Organization Regional Office for the Western Pacific (WPRO-WHO)
Australian Bureau of Statistics, Australian Health Survey Reference Group, Roy Morgan Research, University of Western Australia. Australia Child and Adolescent Survey of Mental Health and Wellbeing 2013-2014
Australian Institute of Health and Welfare. Australia Report on Maternal Deaths 1994-1996. Canberra, Australia: Australian Institute of Health and Welfare, 2001
Australian Institute of Health and Welfare. Australia Maternal Deaths 1997-1999. Canberra, Australia: Australian Institute of Health and Welfare, 2004
Australian Institute of Health and Welfare. Australia Maternal Deaths 2000-2002. Canberra, Australia: Australian Institute of Health and Welfare, 2006
Australian Institute of Health and Welfare. Australia Maternal Deaths 2006-2010. Canberra, Australia: Australian Institute of Health and Welfare, 2014
Australian Institute of Health and Welfare. Australia Maternal Deaths 2008-2012. Canberra, Australia: Australian Institute of Health and Welfare, 2015
Smirnov A, Kemp R, Wells H, Legosz M, Najman JM. Using population screening for recruitment of young adults engaged in illicit drug use:

methodological issues and sampling outcomes. Soc Sci Res. 2014; 45: 89–97
Tran F, Stone M, Huang CY, Lloyd M, Woodhead HJ, Elliott KD, Crock PA, Howard NJ, Craig ME. Population-based incidence of diabetes in Australian youth aged 10-18?yr: increase in type 1 diabetes but not type 2 diabetes. Pediatr Diabetes. 2014; 15(8): 585-90
Chong JW, Craig ME, Cameron FJ, Clarke CF, Rodda CP, Donath SM, Werther GA. Marked increase in type 1 diabetes mellitus incidence in children aged 0-14 yr in Victoria, Australia, from 1999 to 2002. Pediatr Diabetes. 2007; 8(2): 67-73
Craig ME, Femia G, Broyda V, Lloyd M, Howard NJ. Type 2 diabetes in Indigenous and non-Indigenous children and adolescents in New South Wales. Med J Aust. 2007; 186(10): 497-9
Williams ED, Tapp RJ, Magliano DJ, Shaw JE, Zimmet PZ, Oldenburg BF. Health behaviours, socioeconomic status and diabetes incidence: the Australian Diabetes Obesity and Lifestyle Study (AusDiab). Diabetologia. 2010; 53(12): 2538-45
Taplin CE, Craig ME, Lloyd M, Taylor C, Crock P, Silink M, Howard NJ. The rising incidence of childhood type 1 diabetes in New South Wales, 1990-2002. Med J Aust. 2005; 183(5): 243-6
Haynes A, Bower C, Bulsara MK, Jones TW, Davis EA. Continued increase in the incidence of childhood Type 1 diabetes in a population-based Australian sample (1985-2002). Diabetologia. 2004; 47(5): 866-70
Kelly HA, Russell MT, Jones TW, Byrne GC. Dramatic increase in incidence of insulin dependent diabetes mellitus in Western Australia. Med J Aust. 1994; 161(7): 426-9
Craig ME, Howard NJ, Silink M, Chan A. The rising incidence of childhood type 1 diabetes in New South Wales, Australia. J Pediatr Endocrinol Metab. 2000; 13(4): 363-72
World Health Organization (WHO). WHO Mortality Database Version November 2015. Geneva, Switzerland: World Health Organization (WHO)
Australia Vital Registration - Deaths 1980 ICD9 as it appears in World Health Organization (WHO). WHO Mortality Database Version November 2015. Geneva, Switzerland: World Health Organization (WHO)
Australia Vital Registration - Deaths 1981 ICD9 as it appears in World Health Organization (WHO). WHO Mortality Database Version November 2015. Geneva, Switzerland: World Health Organization (WHO)
Australia Vital Registration - Deaths 1982 ICD9 as it appears in World Health Organization (WHO). WHO Mortality Database Version November 2015. Geneva, Switzerland: World Health Organization (WHO)
Australia Vital Registration - Deaths 1983 ICD9 as it appears in World Health Organization (WHO). WHO Mortality Database Version November 2015. Geneva, Switzerland: World Health Organization (WHO)
Australia Vital Registration - Deaths 1984 ICD9 as it appears in World Health Organization (WHO). WHO Mortality Database Version November 2015. Geneva, Switzerland: World Health Organization (WHO)
Australia Vital Registration - Deaths 1985 ICD9 as it appears in World Health Organization (WHO). WHO Mortality Database Version November 2015. Geneva, Switzerland: World Health Organization (WHO)

Australia Vital Registration - Deaths 1986 ICD9 as it appears in World Health Organization (WHO). WHO Mortality Database Version November 2015. Geneva, Switzerland: World Health Organization (WHO)
Australia Vital Registration - Deaths 1987 ICD9 as it appears in World Health Organization (WHO). WHO Mortality Database Version November 2015. Geneva, Switzerland: World Health Organization (WHO)
Australia Vital Registration - Deaths 1988 ICD9 as it appears in World Health Organization (WHO). WHO Mortality Database Version November 2015. Geneva, Switzerland: World Health Organization (WHO)
Australia Vital Registration - Deaths 1989 ICD9 as it appears in World Health Organization (WHO). WHO Mortality Database Version November 2015. Geneva, Switzerland: World Health Organization (WHO)
Australia Vital Registration - Deaths 1990 ICD9 as it appears in World Health Organization (WHO). WHO Mortality Database Version November 2015. Geneva, Switzerland: World Health Organization (WHO)
Australia Vital Registration - Deaths 1991 ICD9 as it appears in World Health Organization (WHO). WHO Mortality Database Version November 2015. Geneva, Switzerland: World Health Organization (WHO)
Australia Vital Registration - Deaths 1992 ICD9 as it appears in World Health Organization (WHO). WHO Mortality Database Version November 2015. Geneva, Switzerland: World Health Organization (WHO)
Australia Vital Registration - Deaths 1993 ICD9 as it appears in World Health Organization (WHO). WHO Mortality Database Version November 2015. Geneva, Switzerland: World Health Organization (WHO)
Australia Vital Registration - Deaths 1994 ICD9 as it appears in World Health Organization (WHO). WHO Mortality Database Version November 2015. Geneva, Switzerland: World Health Organization (WHO)
Australia Vital Registration - Deaths 1995 ICD9 as it appears in World Health Organization (WHO). WHO Mortality Database Version November 2015. Geneva, Switzerland: World Health Organization (WHO)
Australia Vital Registration - Deaths 1996 ICD9 as it appears in World Health Organization (WHO). WHO Mortality Database Version November 2015. Geneva, Switzerland: World Health Organization (WHO)
Australia Vital Registration - Deaths 1997 ICD9 as it appears in World Health Organization (WHO). WHO Mortality Database Version November 2015. Geneva, Switzerland: World Health Organization (WHO)
World Health Organization (WHO). WHO Global Health Observatory - Recorded Alcohol Per Capita Consumption 1960-1979. Geneva, Switzerland: World Health Organization (WHO)
World Health Organization (WHO). WHO Global Health Observatory - Recorded Alcohol Per Capita Consumption 1980-1999. Geneva, Switzerland: World Health Organization (WHO)
World Health Organization (WHO). WHO Global Health Observatory - Recorded Alcohol Per Capita Consumption 2000-2009 by country. Geneva, Switzerland: World Health Organization (WHO)

FAO Supply Utilization Accounts 1961-2013. Personal Correspondence with Dr. Josef Schmidhuber, 2016. [Unpublished]
World Bank. World Development Indicators - Hospital Beds (per 1,000 People). Washington DC, United States of America: World Bank
International Road Federation. World Road Statistics 2015. Geneva, Switzerland: International Road Federation, 2015
Kortt MA, Dollery B. Religion and BMI in Australia. J Relig Health. 2014; 53(1): 217–28
Patterson KAE, Cleland V, Venn A, Blizzard L, Gall S. A cross-sectional study of geographic differences in health risk factors among young Australian adults: the role of socioeconomic position. BMC Public Health. 2014; 14: 1278
Buchmueller TC, Johar M. Obesity and health expenditures: evidence from Australia. Econ Hum Biol. 2015; 17: 42–58
Gill TK, Hill CL, Shanahan EM, Taylor AW, Appleton SL, Grant JF, Shi Z, Dal Grande E, Price K, Adams RJ. Vitamin D levels in an Australian population. BMC Public Health. 2014; 14: 1001
Russell J, Flood V, Yeatman H, Mitchell P. Prevalence and risk factors of food insecurity among a cohort of older Australians. J Nutr Health Aging. 2014; 18(1): 3–8
U.S. Department of Agriculture (USDA). USDA Global Tobacco Database 1960-2005. Washington D.C. , United States: U.S. Department of Agriculture (USDA)
Food and Agriculture Organization of the United Nations (FAO). FAOSTAT Commodity Balances - Crops Primary Equivalent. Rome, Italy: Food and Agriculture Organization of the United Nations (FAO)
Ghaem Maralani H, Tai BC, Wong TY, Tai ES, Li J, Wang JJ, Mitchell P. The prognostic role of body mass index on mortality amongst the middle-aged and elderly: a competing risk analysis. Diabetes Res Clin Pract. 2014; 103(1): 42–50
Mitchell RJ, Lord SR, Harvey LA, Close JCT. Obesity and falls in older people: mediating effects of disease, sedentary behavior, mood, pain and medication use. Arch Gerontol Geriatr. 2015; 60(1): 52–8
Smith KJ, Blizzard L, McNaughton SA, Gall SL, Dwyer T, Venn AJ. Daily eating frequency and cardiometabolic risk factors in young Australian adults: cross-sectional analyses. Br J Nutr. 2012; 108(6): 1086–94
Khambalia A, Phongsavan P, Smith BJ, Keke K, Dan L, Fitzhardinge A, Bauman AE. Prevalence and risk factors of diabetes and impaired fasting glucose in Nauru. BMC Public Health. 2011; 11: 719
Gall SL, Jamrozik K, Blizzard L, Dwyer T, Venn A. Healthy lifestyles and cardiovascular risk profiles in young Australian adults: the Childhood Determinants of Adult Health Study. Eur J Cardiovasc Prev Rehabil. 2009; 16(6): 684–9
Chittleborough CR, Baldock KL, Phillips PJ, Taylor AW, North West Adelaide Health Study Team. Achievement of management targets associated with incident and long-term diagnosed diabetes among a representative population sample. Diabetes Res Clin Pract. 2010; 88(3): 322–7
Bleicher K, Cumming RG, Naganathan V, Seibel MJ, Blyth FM, Le Couteur DG, Handelsman DJ, Creasey HM, Waite LM. Predictors of the rate of BMD loss in older men: findings from the CHAMP study. Osteoporos Int. 2013; 24(7): 1951-63
Cystic Echinococcosis Endemicity Estimates identified through systematic review and personal communication, as provided by GBD 2015 expert.

[Unpublished]
World Health Organization (WHO). WHO Global Health Observatory - Population Living in Trachoma Endemic Areas. Geneva, Switzerland: World Health Organization (WHO)
Cochrane T, Davey RC, de Castella FR. Influence of school community and fitness on prevalence of overweight in Australian school children. Prev Med. 2015; 81: 433–7
Leech RM, McNaughton SA, Timperio A. Clustering of diet, physical activity and sedentary behaviour among Australian children: cross-sectional and longitudinal associations with overweight and obesity. Int J Obes (Lond). 2015; 39(7): 1079–85
Achat HM, Stubbs JM. Socio-economic and ethnic differences in the prevalence of overweight and obesity among school children. J Paediatr Child Health. 2014; 50(10): E77-84
O'Dea JA, Chiang H, Peralta LR. Socioeconomic patterns of overweight, obesity but not thinness persist from childhood to adolescence in a 6-year longitudinal cohort of Australian schoolchildren from 2007 to 2012. BMC Public Health. 2014; 14: 222
Niven P, Scully M, Morley B, Baur L, Crawford D, Pratt IS, Wakefield M, NaSSDA Study Team. What factors are associated with frequent unhealthy snack-food consumption among Australian secondary-school students?. Public Health Nutr. 2015; 18(12): 2153–60
O'Dea JA, Dibley MJ. Prevalence of obesity, overweight and thinness in Australian children and adolescents by socioeconomic status and ethnic cultural group in 2006 and 2012. Int J Public Health. 2014; 59(5): 819–28
Australia - Western Australian Register of Developmental Anomalies Data 2012 - ICBDSR as it appears in International Clearinghouse for Birth Defects Surveillance and Research. International Clearinghouse for Birth Defects Surveillance and Research Annual Report 2014. Rome, Italy: International Clearinghouse for Birth Defects Surveillance and Research, 2016
Australian Bureau of Statistics. Australia National Health Survey 2014-2015. Canberra, Australia: Australian Bureau of Statistics
Almeida OP, Hankey GJ, Yeap BB, Golledge J, Norman PE, Flicker L. Mortality among people with severe mental disorders who reach old age: a longitudinal study of a community-representative sample of 37,892 men. PLoS One. 2014; 9(10): e111882
United Nations Population Division. Abortion Policies: A Global Review. New York, United States of America: United Nations (UN), 2002
United Nations Population Division. World Abortion Policies 2007. New York, United States of America: United Nations (UN), 2007
United Nations Population Division. World Abortion Policies 2013. New York, United States of America: United Nations (UN), 2013
Popova S, Lange S, Probst C, Gmel G, Rehm J, Centre for Addiction and Mental Health (Canada). Estimation of national, regional and global prevalence of alcohol use during pregnancy and fetal alcohol syndrome: a systematic review and meta-analysis. Lancet Glob Health. [Forthcoming]
Wong E, Woodward M, Stevenson C, Backholer K, Sarink D, Peeters A. Prevalence of disability in Australian elderly: Impact of trends in obesity and diabetes. Prev Med. 2016; 82: 105-12
International Social Survey Programme (ISSP). Australia International Social Survey Programme: Role of Government II 1990. Mannheim, Germany: GESIS - Leibniz Institute for the Social Sciences, 1992

International Social Survey Programme (ISSP). Australia International Social Survey Programme: Social Inequality II 1993. Mannheim, Germany: GESIS - Leibniz Institute for the Social Sciences, 1994
Maxwell S, Bower C, O'Leary P. Impact of prenatal screening and diagnostic testing on trends in Down syndrome births and terminations in Western Australia 1980 to 2013. Prenat Diagn. 2015; 35(13): 1324-30
Glasson EJ, Jacques A, Wong K, Bourke J, Leonard H. Improved Survival in Down Syndrome over the Last 60 Years and the Impact of Perinatal Factors in Recent Decades. J Pediatr. 2016; 169: 214-20e1
Lee MG, Brizard CP, Galati JC, Iyengar AJ, Rakhra SS, Konstantinov IE, Pflaumer A, d'Udekem Y. Outcomes of patients born with single-ventricle physiology and aortic arch obstruction: the 26-year Melbourne experience. J Thorac Cardiovasc Surg. 2014; 148(1): 194-201
Warrington NM, Zhu G, Dy V, Heath AC, Madden PA, Hemani G, Kemp JP, Mcmahon G, St Pourcain B, Timpson NJ, Taylor CM, Golding J, Lawlor DA, Steer C, Montgomery GW, Martin NG, Davey Smith G, Evans DM, Whitfield JB. Genome-wide association study of blood lead shows multiple associations near ALAD. Hum Mol Genet. 2015; 24(13): 3871-9
Gulson B, Anderson P, Taylor A. Surface dust wipes are the best predictors of blood leads in young children with elevated blood lead levels. Environ Res. 2013; 126: 171-8
Toumbourou JW, Evans-Whipp TJ, Smith R, Hemphill SA, Herrenkohl TI, Catalano RF. Adolescent Predictors and Environmental Correlates of Young Adult Alcohol Use Problems. Addiction. 2014; 109(3): 417–24
Zeleke BM, Bell RJ, Billah B, Davis SR. Symptomatic pelvic floor disorders in community-dwelling older Australian women. Maturitas. 2016; 85: 34–41
Lombard C, Harrison C, Kozica S, Zoungas S, Ranasinha S, Teede H. Preventing Weight Gain in Women in Rural Communities: A Cluster Randomised Controlled Trial. PLoS Med. 2016; 13(1): e1001941
Euromonitor International. Euromonitor Passport - Fresh Foods Market Statistics. London, United Kingdom: Euromonitor International
Kirby Institute, University of New South Wales. Australia HIV, Viral Hepatitis and Sexually Transmissible Infections Annual Surveillance Report 2016. Darlinghurst, Australia: Kirby Institute, University of New South Wales, 2016
Rosilene WVR, Cumming R, Travison T, Blyth F, Naganathan V, Allman-Farinelli M, Hirani V. Relative Validity of a Diet History Questionnaire Against a Four-Day Weighed Food Record among Older Men in Australia: The Concord Health and Ageing in Men Project (CHAMP). J Nutr Health Aging. 2015; 19(6): 603–10
Rangan AM, Samman S. Zinc intake and its dietary sources: results of the 2007 Australian National Children’s Nutrition and Physical Activity Survey. Nutrients. 2012; 4(7): 611–24
Burrows TL, Collins K, Watson J, Guest M, Boggess MM, Neve M, Rollo M, Duncanson K, Collins CE. Validity of the Australian Recommended Food Score as a diet quality index for Pre-schoolers. Nutr J. 2014; 13: 87
Larney S, Hickman M, Guy R, Grebely J, Dore GJ, Gray RT, Day CA, Kimber J, Dagenhardt L. Estimating the Number of People who Inject Drugs in Australia. BMC Public Health. 2017; 17: 757

World Health Organization (WHO). Sodium Intakes Around the World. 2007
Baillie AJ, Teesson M. Continuous, categorical and mixture models of DSM-IV alcohol and cannabis use disorders in the Australian community. Addiction. 2010; 105(7): 1246–53
Teesson M, Slade T, Swift W, Mills K, Memedovic S, Mewton L, Grove R, Newton N, Hall W. Prevalence, correlates and comorbidity of DSM-IV Cannabis Use and Cannabis Use Disorders in Australia. Aust N Z J Psychiatry. 2012; 46(12): 1182–92
Flood VM, Burlutsky G, Webb KL, Wang JJ, Smith WT, Mitchell P. Food and nutrient consumption trends in older Australians: a 10-year cohort study. Eur J Clin Nutr. 2010; 64(6): 603–13
Australian National University. Australia Family Project 1986. Acton, Australia: Australian Data Archive
International Road Federation. World Road Statistics 2009 Database and World Road Statistics 2007
BP. BP Statistical Review of World Energy 2016. London, United Kingdom: BP, 2016
International Labour Organization (ILO). International Labour Organization Database (ILOSTAT) - Employment to Population Ratio by Sex and Age. International Labour Organization (ILO)
Global Burden of Disease Collaborative Network. Global Burden of Disease Study 2016 (GBD 2016) Covariates 1980-2016. Seattle, United States of America: Institute for Health Metrics and Evaluation (IHME), 2017
ISSP Research Group (1997): International Social Survey Programme: Family and Changing Gender Roles II - ISSP 1994. GESIS Data Archive, Cologne. ZA2620 Data file Version 1.0.0, doi:10.4232/1.2620
International Social Survey Programme (ISSP). Australia International Social Survey Programme: National Identity I 1994-1996. Mannheim, Germany: GESIS - Leibniz Institute for the Social Sciences, 1998
International Social Survey Programme (ISSP). Australia International Social Survey Programme: Role of Government III 1997. Mannheim, Germany: GESIS - Leibniz Institute for the Social Sciences, 1999
International Social Survey Programme (ISSP). Australia International Social Survey Programme: Social Inequality III 1999-2000. Mannheim, Germany: GESIS - Leibniz Institute for the Social Sciences, 2002
ISSP Research Group (2013): International Social Survey Programme: Family and Changing Gender Roles III - ISSP 2002. GESIS Data Archive, Cologne. ZA3880 Data file Version 1.1.0, doi:10.4232/1.11564
ISSP Research Group (2012): International Social Survey Programme: National Identity II - ISSP 2003. GESIS Data Archive, Cologne. ZA3910 Data file Version 2.1.0, doi:10.4232/1.11449
International Social Survey Programme (ISSP). Australia International Social Survey Programme: Citizenship 2005. Mannheim, Germany: GESIS - Leibniz Institute for the Social Sciences, 2012
International Social Survey Programme (ISSP). Australia International Social Survey Programme: Work Orientation III 2005. Mannheim, Germany: GESIS - Leibniz Institute for the Social Sciences, 2013

ISSP Research Group (2008): International Social Survey Programme: Role of Government IV - ISSP 2006. GESIS Data Archive, Cologne. ZA4700 Data file Version 1.0.0, doi:10.4232/1.4700
International Social Survey Programme (ISSP). Australia International Social Survey Programme: Religion III 2009-2010. Mannheim, Germany: GESIS - Leibniz Institute for the Social Sciences, 2012
ISSP Research Group (2017): International Social Survey Programme: Social Inequality IV - ISSP 2009. GESIS Data Archive, Cologne. ZA5400 Data file Version 4.0.0, doi:10.4232/1.12777.
ISSP Research Group (2016): International Social Survey Programme: Family and Changing Gender Roles IV - ISSP 2012. GESIS Data Archive, Cologne. ZA5900 Data file Version 4.0.0, doi:10.4232/1.12661
ISSP Research Group (2000): International Social Survey Programme: Religion II - ISSP 1998. GESIS Data Archive, Cologne. ZA3190 Data file Version 1.0.0, https://doi.org/10.4232/1.3190
International Social Survey Programme (ISSP). Australia International Social Survey Program: Environment I 1992-1994. Mannheim, Germany: GESIS - Leibniz Institute for the Social Sciences, 1995
ISSP Research Group (2003): International Social Survey Programme: Social Relations and Support Systems / Social Networks II - ISSP 2001. GESIS Data Archive, Cologne. ZA3680 Data file Version 1.0.0, doi:10.4232/1.3680
World Health Organization (WHO). WHO Mortality Database Version October 2017. Geneva, Switzerland: World Health Organization (WHO)
Australia Vital Registration - Deaths 2001 ICD10 as it appears in World Health Organization (WHO). WHO Mortality Database Version October 2017. Geneva, Switzerland: World Health Organization (WHO)
Australia Vital Registration - Deaths 2003 ICD10 as it appears in World Health Organization (WHO). WHO Mortality Database Version October 2017. Geneva, Switzerland: World Health Organization (WHO)
Australia Vital Registration - Deaths 2004 ICD10 as it appears in World Health Organization (WHO). WHO Mortality Database Version October 2017. Geneva, Switzerland: World Health Organization (WHO)
Australia Vital Registration - Deaths 2006 ICD10 as it appears in World Health Organization (WHO). WHO Mortality Database Version October 2017. Geneva, Switzerland: World Health Organization (WHO)
Australia Vital Registration - Deaths 2010 ICD10 as it appears in World Health Organization (WHO). WHO Mortality Database Version October 2017. Geneva, Switzerland: World Health Organization (WHO)
Australia Vital Registration - Deaths 2011 ICD10 as it appears in World Health Organization (WHO). WHO Mortality Database Version October 2017. Geneva, Switzerland: World Health Organization (WHO)
Australia Vital Registration - Deaths 2012 ICD10 as it appears in World Health Organization (WHO). WHO Mortality Database Version October 2017. Geneva, Switzerland: World Health Organization (WHO)
Australia Vital Registration - Deaths 2013 ICD10 as it appears in World Health Organization (WHO). WHO Mortality Database Version October 2017.

Geneva, Switzerland: World Health Organization (WHO)
Hong TP, Gow P, Fink M, Dev A, Roberts S, Nicoll A, Lubel J, Kronborg I, Arachchi N, Ryan M, Kemp W, Knight V, Farrugia H, Thursfield V, Desmond P, Thompson AJ, Bell S. Novel population-based study finding higher than reported hepatocellular carcinoma incidence suggests an updated approach is needed. Hepatology. 2016; 63(4): 1205-12
Food Fortification Initiative (United States), Global Alliance for Improved Nutrition (GAIN), Iodine Global Network (Canada), Micronutrient Forum (Canada). Global Fortification Data Exchange: Legislation and Standards, Year When Food Fortification Mandated, and Count of Nutrients In Fortification Standards
ISSP Research Group (2016): International Social Survey Programme: Citizenship II - ISSP 2014. GESIS Data Archive, Cologne. ZA6670 Data file Version 2.0.0, doi:10.4232/1.12590
The International Social Survey Programme (ISSP) represents a series of cross-national collaborations between organizations conducting similar social science research. ISSP researchers focus on developing survey questions that are relevant and comprehensible in all countries and languages, with survey results that are comparable across nations.Topics covered on this round of the ISSP include: working conditions; labor relations and conflict; and employment and unemployment
Gurjav U, Jelfs P, Hill-Cawthorne GA, Marais BJ, Sintchenko V. Genotype heterogeneity of Mycobacterium tuberculosis within geospatial hotspots suggests foci of imported infection in Sydney, Australia. Infect Genet Evol. 2016; 40: 346–51
Australian Institute of Health and Welfare, National Diabetes Register (NDR) (Australia). Australia Incidence of Insulin-Treated Diabetes 2015. Canberra, Australia: Australian Institute of Health and Welfare, 2017
Australian Institute of Health and Welfare, National Diabetes Register (NDR) (Australia). Australia Incidence of Insulin-Treated Diabetes 2014. Canberra, Australia: Australian Institute of Health and Welfare, 2016
Gallego B, Sintchenko V, Jelfs P, Coiera E, Gilbert GL. Three-year longitudinal study of genotypes of Mycobacterium tuberculosis in a low prevalence population. Pathology. 2010; 42(3): 267–72
World Health Organization Regional Office for the Western Pacific (WPRO-WHO). WPRO Dengue Situation Update Number 533, 2 January 2018. Manila, Philippines: World Health Organization Regional Office for the Western Pacific (WPRO-WHO), 2018
Kirby Institute. HIV, viral hepatitis and sexually transmissible infections in Australia: annual surveillance report 2017. Sydney: Kirby Institute, UNSW Sydney; 2017
National Centre for Immunisation Research and Surveillance of Vaccine Preventable Diseases (NCRIS) (Australia). Australia Immunisation Coverage Annual Report 2013. Commun Dis Intell. 2016; 40(1): E146-69
National Centre for Immunisation Research and Surveillance of Vaccine Preventable Diseases (NCRIS) (Australia). Australia Immunisation Coverage Annual Report 2014. Commun Dis Intell. 2017; 41(1): E68-90
Gallup. Australia World Poll 2007

World Health Organization (WHO). Global Status Report on Road Safety 2013. Geneva, Switzerland: World Health Organization (WHO), 2013
World Health Organization (WHO). Global Status Report on Road Safety 2015. Geneva, Switzerland: World Health Organization (WHO), 2015
International Fuel Quality Center (IFQC). Overview of Leaded Gasoline and Sulfur Levels in Gasoline and Diesel . 2002
Australasian Association of Cancer Registries, Australian Institute of Health and Welfare. Australia Cancer Incidence and Mortality Books 2017. Canberra, Australia: Australian Institute of Health and Welfare, 2017
Australia Cancer Registry 1992-2014 - IICC-3 as it appears in International Agency for Research on Cancer (IARC), International Association of Cancer Registries (IACR), World Health Organization (WHO). International Incidence of Childhood Cancer Volume III. Lyon, France: International Agency for Research on Cancer (IARC), 2017
Organization for Economic Co-operation and Development (OECD). Organization for Economic Co-operation and Development Data - Hospital Beds. Paris, France: Organization for Economic Co-operation and Development (OECD)
World Health Organization (WHO). WHO Global Health Observatory - Hospital Bed Density Data by Country . Geneva, Switzerland: World Health Organization (WHO)
World Health Organization (WHO). WHO Mortality Database Version December 2018. Geneva, Switzerland: World Health Organization (WHO)
Australia Vital Registration - Deaths 2014 ICD10 as it appears in World Health Organization (WHO). WHO Mortality Database Version December 2018. Geneva, Switzerland: World Health Organization (WHO)
Australia Vital Registration - Deaths 2015 ICD10 as it appears in World Health Organization (WHO). WHO Mortality Database Version December 2018. Geneva, Switzerland: World Health Organization (WHO)
World Health Organization Regional Office for the Western Pacific (WPRO-WHO). WPRO Dengue Situation Updates 2019. Manila, Philippines: World Health Organization Regional Office for the Western Pacific (WPRO-WHO), 2019
Gautam SS, Mac Aogain M, Cooley LA, Haug G, Fyfe JA, Globan M, O'Toole RF. Molecular epidemiology of tuberculosis in Tasmania and genomic characterisation of its first known multi-drug resistant case. PLoS One. 2018; 13(2): e0192351
World Health Organization (WHO). Global Status Report on Road Safety 2018. Geneva, Switzerland: World Health Organization (WHO), 2018
World Health Organization (WHO). WHO Mortality Database Version May 2019. Geneva, Switzerland: World Health Organization (WHO)
Australia Vital Registration - Deaths 2016 ICD10 as it appears in World Health Organization (WHO). WHO Mortality Database Version May 2019. Geneva, Switzerland: World Health Organization (WHO)
Gallup. Gallup Analytics - World Poll Negative Experience Index. Washington, D.C., United States of America: Gallup, 2019
Australia World Poll 2015 as it appears in Gallup. Gallup Analytics - World Poll Negative Experience Index. Washington, D.C., United States of America: Gallup, 2019
Australia World Poll 2016 as it appears in Gallup. Gallup Analytics - World Poll Negative Experience Index. Washington, D.C., United States of America: Gallup, 2019

Australia World Poll 2017 as it appears in Gallup. Gallup Analytics - World Poll Negative Experience Index. Washington, D.C., United States of America: Gallup, 2019
Australia World Poll 2008 as it appears in Gallup. Gallup Analytics - World Poll Negative Experience Index. Washington, D.C., United States of America: Gallup, 2019
Australia World Poll 2010 as it appears in Gallup. Gallup Analytics - World Poll Negative Experience Index. Washington, D.C., United States of America: Gallup, 2019
Australia World Poll 2011 as it appears in Gallup. Gallup Analytics - World Poll Negative Experience Index. Washington, D.C., United States of America: Gallup, 2019
Australia World Poll 2012 as it appears in Gallup. Gallup Analytics - World Poll Negative Experience Index. Washington, D.C., United States of America: Gallup, 2019
Australia World Poll 2013 as it appears in Gallup. Gallup Analytics - World Poll Negative Experience Index. Washington, D.C., United States of America: Gallup, 2019
Australia World Poll 2014 as it appears in Gallup. Gallup Analytics - World Poll Negative Experience Index. Washington, D.C., United States of America: Gallup, 2019
Australia World Poll 2018 as it appears in Gallup. Gallup Analytics - World Poll Negative Experience Index. Washington, D.C., United States of America: Gallup, 2019
International Road Federation. World Road Statistics 2018. Geneva, Switzerland: International Road Federation, 2018
United Nations Children's Fund (UNICEF), World Health Organization (WHO). WHO and UNICEF Reported Official Target Population, Number of Doses Administered and Official Coverage 1966-2018. Geneva, Switzerland: World Health Organization (WHO), 2019
Malaria Atlas Project. Sickle Haemoglobin (Hbs) Allele Frequency Layer. Oxford, United Kingdom: Malaria Atlas Project, 2013
Turnour CE, Cretikos MA, Conaty SJ. Prevalence of chronic hepatitis B in South Western Sydney: evaluation of the country of birth method using maternal seroprevalence data. Aust N Z J Public Health. 2011; 35(1): 226
Bowden FJ, OKeefe EJ, Primrose R, Currie MJ. Sexually transmitted infections, blood-borne viruses and risk behaviour in an Australian senior high school population-the SHLiRP study. Sex Health. 2005; 2(4): 22936
Burgess MA, McIntosh ED, Allars HM, Kenrick KG. Hepatitis B in urban Australian schoolchildren. No evidence of horizontal transmission between high-risk and low-risk groups. Med J Aust. 1993; 159(5): 3159
World Health Organization (WHO). WHO Report on the Global Tobacco Epidemic 2019. Geneva, Switzerland: World Health Organization (WHO), 2019
Amin J, Gidding H, Gilbert G, Backhouse J, Kaldor J, Dore G, Burgess M. Hepatitis C prevalence-a nationwide serosurvey. Commun Dis Intell Q Rep. 2004; 28(4): 51721
Euromonitor International. Euromonitor Passport - Smoking Tobacco Statistics . London, United Kingdom: Euromonitor International

Euromonitor International. Euromonitor Passport - Alcoholic Drinks Statistics . London, United Kingdom: Euromonitor International
Australia - South Australia Air Monitoring Network 2014 as it appears in Shaddick G, Thomas ML. Particulate Matter 2.5 and 10 Surface Monitor Station Expanded Database 2008-2017. [Unpublished]
Australia - Western Australia Air Quality Monitoring Network PM2.5 Data 2014 as it appears in Shaddick G, Thomas ML. Particulate Matter 2.5 and 10 Surface Monitor Station Expanded Database 2008-2017. [Unpublished]
Australia - New South Wales Air Quality PM2.5 and PM 10 Particulate Data 2014 as it appears in Shaddick G, Thomas ML. Particulate Matter 2.5 and 10 Surface Monitor Station Expanded Database 2008-2017. [Unpublished]
Australia - Northern Territory Ambient Air Quality Monitoring Report 2014 as it appears in Shaddick G, Thomas ML. Particulate Matter 2.5 and 10 Surface Monitor Station Expanded Database 2008-2017. [Unpublished]
Australia - Western Australia Air Monitoring Report 2016 as it appears in Shaddick G, Thomas ML. Particulate Matter 2.5 and 10 Surface Monitor Station Expanded Database 2008-2017. [Unpublished]
Australia - Queensland Air Monitoring 2015 as it appears in Shaddick G, Thomas ML. Particulate Matter 2.5 and 10 Surface Monitor Station Expanded Database 2008-2017. [Unpublished]
Australia - Queensland Air Monitoring 2016 as it appears in Shaddick G, Thomas ML. Particulate Matter 2.5 and 10 Surface Monitor Station Expanded Database 2008-2017. [Unpublished]
Australia - Northern Territory Air Quality Monitoring Network All Available Air Quality Index Values 2012 as it appears in Shaddick G, Thomas ML. Particulate Matter 2.5 and 10 Surface Monitor Station Expanded Database 2008-2017. [Unpublished]
Australia - Northern Territory Air Quality Monitoring Network All Available Air Quality Index Values 2013 as it appears in Shaddick G, Thomas ML. Particulate Matter 2.5 and 10 Surface Monitor Station Expanded Database 2008-2017. [Unpublished]
Australia - Northern Territory Air Quality Monitoring Network All Available Air Quality Index Values 2014 as it appears in Shaddick G, Thomas ML. Particulate Matter 2.5 and 10 Surface Monitor Station Expanded Database 2008-2017. [Unpublished]
Australia - Northern Territory Air Quality Monitoring Network All Available Air Quality Index Values 2015 as it appears in Shaddick G, Thomas ML. Particulate Matter 2.5 and 10 Surface Monitor Station Expanded Database 2008-2017. [Unpublished]
Australia - Northern Territory Air Quality Monitoring Network All Available Air Quality Index Values 2016 as it appears in Shaddick G, Thomas ML. Particulate Matter 2.5 and 10 Surface Monitor Station Expanded Database 2008-2017. [Unpublished]
Australia - New South Wales Air Quality PM2.5 and PM 10 Particulate Data 2013 as it appears in Shaddick G, Thomas ML. Particulate Matter 2.5 and 10 Surface Monitor Station Expanded Database 2008-2017. [Unpublished]
Australia - New South Wales Air Quality PM2.5 and PM 10 Particulate Data 2015 as it appears in Shaddick G, Thomas ML. Particulate Matter 2.5 and 10 Surface Monitor Station Expanded Database 2008-2017. [Unpublished]
Australia - New South Wales Air Quality PM2.5 and PM 10 Particulate Data 2016 as it appears in Shaddick G, Thomas ML. Particulate Matter 2.5 and

10 Surface Monitor Station Expanded Database 2008-2017. [Unpublished]
Australia - South Australia Air Monitoring Network 2012 as it appears in Shaddick G, Thomas ML. Particulate Matter 2.5 and 10 Surface Monitor Station Expanded Database 2008-2017. [Unpublished]
Australia - South Australia Air Monitoring Network 2013 as it appears in Shaddick G, Thomas ML. Particulate Matter 2.5 and 10 Surface Monitor Station Expanded Database 2008-2017. [Unpublished]
Australia - South Australia Air Monitoring Network 2015 as it appears in Shaddick G, Thomas ML. Particulate Matter 2.5 and 10 Surface Monitor Station Expanded Database 2008-2017. [Unpublished]
Australia - South Australia Air Monitoring Network 2016 as it appears in Shaddick G, Thomas ML. Particulate Matter 2.5 and 10 Surface Monitor Station Expanded Database 2008-2017. [Unpublished]
United Nations Children's Fund (UNICEF). UNICEF Maternal and Newborn Health Coverage Database as of November 2019. New York, United States of America: United Nations Children's Fund (UNICEF), 2019
Global Burden of Disease Collaborative Network. Global Health Spending 1995-2017. Seattle, United States of America: Institute for Health Metrics and Evaluation (IHME), 2020
International Road Federation. World Road Statistics 2019. Geneva, Switzerland: International Road Federation, 2019
Feenstra, Robert C., Robert Inklaar and Marcel P. Timmer (2015), "The Next Generation of the Penn World Table" American Economic Review, 105(10), 3150-3182, available for download at www.ggdc.net/pwt
Maddison Project Database, version 2018. Bolt, Jutta, Robert Inklaar, Herman de Jong and Jan Luiten van Zanden (2018), “Rebasing ‘Maddison’: new income comparisons and the shape of long-run economic development”, Maddison Project Working paper 10
World Bank. World Development Indicators - Gross Domestic Product (GDP). Washington DC, United States of America: World Bank
United Nations Environment Programme. Leaded Petrol Phase-out: Global Status, March 2018. Nairobi, Kenya: United Nations Environment Programme
World Health Organization (WHO). WHO Mortality Database Version February 2014. Geneva, Switzerland: World Health Organization (WHO)

Supplementary Table 6 GBD data input sources for mortality

Citation
Australia Vital Registration - Deaths 1968 ICD8 as it appears in World Health Organization (WHO). WHO Mortality Database Version July 2012. Geneva, Switzerland: World Health Organization (WHO)
Australia Vital Registration - Deaths 1969 ICD8 as it appears in World Health Organization (WHO). WHO Mortality Database Version July 2012. Geneva, Switzerland: World Health Organization (WHO)
Australia Vital Registration - Deaths 1970 ICD8 as it appears in World Health Organization (WHO). WHO Mortality Database Version July 2012. Geneva, Switzerland: World Health Organization (WHO)
Australia Vital Registration - Deaths 1971 ICD8 as it appears in World Health Organization (WHO). WHO Mortality Database Version July 2012. Geneva, Switzerland: World Health Organization (WHO)
Australia Vital Registration - Deaths 1972 ICD8 as it appears in World Health Organization (WHO). WHO Mortality Database Version July 2012. Geneva, Switzerland: World Health Organization (WHO)
Australia Vital Registration - Deaths 1973 ICD8 as it appears in World Health Organization (WHO). WHO Mortality Database Version July 2012. Geneva, Switzerland: World Health Organization (WHO)
Australia Vital Registration - Deaths 1974 ICD8 as it appears in World Health Organization (WHO). WHO Mortality Database Version July 2012. Geneva, Switzerland: World Health Organization (WHO)
Australia Vital Registration - Deaths 1975 ICD8 as it appears in World Health Organization (WHO). WHO Mortality Database Version July 2012. Geneva, Switzerland: World Health Organization (WHO)
Australia Vital Registration - Deaths 1976 ICD8 as it appears in World Health Organization (WHO). WHO Mortality Database Version July 2012. Geneva, Switzerland: World Health Organization (WHO)
Australia Vital Registration - Deaths 1977 ICD8 as it appears in World Health Organization (WHO). WHO Mortality Database Version July 2012. Geneva, Switzerland: World Health Organization (WHO)
Australia Vital Registration - Deaths 1978 ICD8 as it appears in World Health Organization (WHO). WHO Mortality Database Version July 2012. Geneva, Switzerland: World Health Organization (WHO)
Australian Bureau of Statistics. Australia Vital Registration - Deaths 2005
Australian Bureau of Statistics. Australia Vital Registration - Deaths 2010. Canberra, Australia: Australian Bureau of Statistics
World Health Organization (WHO). WHO Mortality Database Version February 2014. Geneva, Switzerland: World Health Organization (WHO)
Australia Vital Registration - Deaths 1950 ICD7 as it appears in World Health Organization (WHO). WHO Mortality Database Version February 2014. Geneva, Switzerland: World Health Organization (WHO)

Australia Vital Registration - Deaths 1951 ICD7 as it appears in World Health Organization (WHO). WHO Mortality Database Version February 2014. Geneva, Switzerland: World Health Organization (WHO)
Australia Vital Registration - Deaths 1952 ICD7 as it appears in World Health Organization (WHO). WHO Mortality Database Version February 2014. Geneva, Switzerland: World Health Organization (WHO)
Australia Vital Registration - Deaths 1953 ICD7 as it appears in World Health Organization (WHO). WHO Mortality Database Version February 2014. Geneva, Switzerland: World Health Organization (WHO)
Australia Vital Registration - Deaths 1954 ICD7 as it appears in World Health Organization (WHO). WHO Mortality Database Version February 2014. Geneva, Switzerland: World Health Organization (WHO)
Australia Vital Registration - Deaths 1955 ICD7 as it appears in World Health Organization (WHO). WHO Mortality Database Version February 2014. Geneva, Switzerland: World Health Organization (WHO)
Australia Vital Registration - Deaths 1956 ICD7 as it appears in World Health Organization (WHO). WHO Mortality Database Version February 2014. Geneva, Switzerland: World Health Organization (WHO)
Australia Vital Registration - Deaths 1957 ICD7 as it appears in World Health Organization (WHO). WHO Mortality Database Version February 2014. Geneva, Switzerland: World Health Organization (WHO)
Australia Vital Registration - Deaths 1958 ICD7 as it appears in World Health Organization (WHO). WHO Mortality Database Version February 2014. Geneva, Switzerland: World Health Organization (WHO)
Australia Vital Registration - Deaths 1959 ICD7 as it appears in World Health Organization (WHO). WHO Mortality Database Version February 2014. Geneva, Switzerland: World Health Organization (WHO)
Australia Vital Registration - Deaths 1960 ICD7 as it appears in World Health Organization (WHO). WHO Mortality Database Version February 2014. Geneva, Switzerland: World Health Organization (WHO)
Australia Vital Registration - Deaths 1961 ICD7 as it appears in World Health Organization (WHO). WHO Mortality Database Version February 2014. Geneva, Switzerland: World Health Organization (WHO)
Australia Vital Registration - Deaths 1962 ICD7 as it appears in World Health Organization (WHO). WHO Mortality Database Version February 2014. Geneva, Switzerland: World Health Organization (WHO)
Australia Vital Registration - Deaths 1963 ICD7 as it appears in World Health Organization (WHO). WHO Mortality Database Version February 2014. Geneva, Switzerland: World Health Organization (WHO)
Australia Vital Registration - Deaths 1964 ICD7 as it appears in World Health Organization (WHO). WHO Mortality Database Version February 2014. Geneva, Switzerland: World Health Organization (WHO)
Australia Vital Registration - Deaths 1965 ICD7 as it appears in World Health Organization (WHO). WHO Mortality Database Version February 2014. Geneva, Switzerland: World Health Organization (WHO)

Australia Vital Registration - Deaths 1966 ICD7 as it appears in World Health Organization (WHO). WHO Mortality Database Version February 2014. Geneva, Switzerland: World Health Organization (WHO)
Australia Vital Registration - Deaths 1967 ICD7 as it appears in World Health Organization (WHO). WHO Mortality Database Version February 2014. Geneva, Switzerland: World Health Organization (WHO)
Australia Vital Registration Death Data 1950 as it appears in United Nations Statistics Division (UNSD). United Nations Demographic Yearbook - Historical Supplement 1997. New York, United States of America: United Nations (UN)
Australia Vital Registration Death Data 1951 as it appears in United Nations Statistics Division (UNSD). United Nations Demographic Yearbook - Historical Supplement 1997. New York, United States of America: United Nations (UN)
Australia Vital Registration Death Data 1952 as it appears in United Nations Statistics Division (UNSD). United Nations Demographic Yearbook - Historical Supplement 1997. New York, United States of America: United Nations (UN)
Australia Vital Registration Death Data 1953 as it appears in United Nations Statistics Division (UNSD). United Nations Demographic Yearbook - Historical Supplement 1997. New York, United States of America: United Nations (UN)
Australia Vital Registration Death Data 1954 as it appears in United Nations Statistics Division (UNSD). United Nations Demographic Yearbook - Historical Supplement 1997. New York, United States of America: United Nations (UN)
Australia Vital Registration Death Data 1955 as it appears in United Nations Statistics Division (UNSD). United Nations Demographic Yearbook - Historical Supplement 1997. New York, United States of America: United Nations (UN)
Australia Vital Registration Death Data 1956 as it appears in United Nations Statistics Division (UNSD). United Nations Demographic Yearbook - Historical Supplement 1997. New York, United States of America: United Nations (UN)
Australia Vital Registration Death Data 1957 as it appears in United Nations Statistics Division (UNSD). United Nations Demographic Yearbook - Historical Supplement 1997. New York, United States of America: United Nations (UN)
Australia Vital Registration Death Data 1958 as it appears in United Nations Statistics Division (UNSD). United Nations Demographic Yearbook - Historical Supplement 1997. New York, United States of America: United Nations (UN)
Australia Vital Registration Death Data 1959 as it appears in United Nations Statistics Division (UNSD). United Nations Demographic Yearbook - Historical Supplement 1997. New York, United States of America: United Nations (UN)
Australia Vital Registration Death Data 1960 as it appears in United Nations Statistics Division (UNSD). United Nations Demographic Yearbook - Historical Supplement 1997. New York, United States of America: United Nations (UN)
Australia Vital Registration Death Data 1961 as it appears in United Nations Statistics Division (UNSD). United Nations Demographic Yearbook - Historical Supplement 1997. New York, United States of America: United Nations (UN)
Australia Vital Registration Death Data 1962 as it appears in United Nations Statistics Division (UNSD). United Nations Demographic Yearbook - Historical Supplement 1997. New York, United States of America: United Nations (UN)

Australia Vital Registration Death Data 1963 as it appears in United Nations Statistics Division (UNSD). United Nations Demographic Yearbook - Historical Supplement 1997. New York, United States of America: United Nations (UN)
Australia Vital Registration Death Data 1964 as it appears in United Nations Statistics Division (UNSD). United Nations Demographic Yearbook - Historical Supplement 1997. New York, United States of America: United Nations (UN)
Australia Vital Registration Death Data 1965 as it appears in United Nations Statistics Division (UNSD). United Nations Demographic Yearbook - Historical Supplement 1997. New York, United States of America: United Nations (UN)
Australia Vital Registration Death Data 1966 as it appears in United Nations Statistics Division (UNSD). United Nations Demographic Yearbook - Historical Supplement 1997. New York, United States of America: United Nations (UN)
Australia Vital Registration Death Data 1967 as it appears in United Nations Statistics Division (UNSD). United Nations Demographic Yearbook - Historical Supplement 1997. New York, United States of America: United Nations (UN)
Australia Vital Registration Death Data 1968 as it appears in United Nations Statistics Division (UNSD). United Nations Demographic Yearbook - Historical Supplement 1997. New York, United States of America: United Nations (UN)
Australia Vital Registration Death Data 1969 as it appears in United Nations Statistics Division (UNSD). United Nations Demographic Yearbook - Historical Supplement 1997. New York, United States of America: United Nations (UN)
Australia Vital Registration Death Data 1970 as it appears in United Nations Statistics Division (UNSD). United Nations Demographic Yearbook - Historical Supplement 1997. New York, United States of America: United Nations (UN)
Australia Vital Registration Death Data 1971 as it appears in United Nations Statistics Division (UNSD). United Nations Demographic Yearbook - Historical Supplement 1997. New York, United States of America: United Nations (UN)
Australia Vital Registration Death Data 1972 as it appears in United Nations Statistics Division (UNSD). United Nations Demographic Yearbook - Historical Supplement 1997. New York, United States of America: United Nations (UN)
Australia Vital Registration Death Data 1973 as it appears in United Nations Statistics Division (UNSD). United Nations Demographic Yearbook - Historical Supplement 1997. New York, United States of America: United Nations (UN)
Australia Vital Registration Death Data 1974 as it appears in United Nations Statistics Division (UNSD). United Nations Demographic Yearbook - Historical Supplement 1997. New York, United States of America: United Nations (UN)
Australia Vital Registration Death Data 1975 as it appears in United Nations Statistics Division (UNSD). United Nations Demographic Yearbook - Historical Supplement 1997. New York, United States of America: United Nations (UN)
Australia Vital Registration Death Data 1976 as it appears in United Nations Statistics Division (UNSD). United Nations Demographic Yearbook - Historical Supplement 1997. New York, United States of America: United Nations (UN)
Australia Vital Registration Death Data 1977 as it appears in United Nations Statistics Division (UNSD). United Nations Demographic Yearbook - Historical Supplement 1997. New York, United States of America: United Nations (UN)

Australia Vital Registration Death Data 1978 as it appears in United Nations Statistics Division (UNSD). United Nations Demographic Yearbook - Historical Supplement 1997. New York, United States of America: United Nations (UN)
Australia Vital Registration Death Data 1979 as it appears in United Nations Statistics Division (UNSD). United Nations Demographic Yearbook - Historical Supplement 1997. New York, United States of America: United Nations (UN)
Australia Vital Registration Death Data 1980 as it appears in United Nations Statistics Division (UNSD). United Nations Demographic Yearbook - Historical Supplement 1997. New York, United States of America: United Nations (UN)
Australia Vital Registration Death Data 1981 as it appears in United Nations Statistics Division (UNSD). United Nations Demographic Yearbook - Historical Supplement 1997. New York, United States of America: United Nations (UN)
Australia Vital Registration Death Data 1982 as it appears in United Nations Statistics Division (UNSD). United Nations Demographic Yearbook - Historical Supplement 1997. New York, United States of America: United Nations (UN)
Australia Vital Registration Death Data 1983 as it appears in United Nations Statistics Division (UNSD). United Nations Demographic Yearbook - Historical Supplement 1997. New York, United States of America: United Nations (UN)
Australia Vital Registration Death Data 1984 as it appears in United Nations Statistics Division (UNSD). United Nations Demographic Yearbook - Historical Supplement 1997. New York, United States of America: United Nations (UN)
Australia Vital Registration Death Data 1985 as it appears in United Nations Statistics Division (UNSD). United Nations Demographic Yearbook - Historical Supplement 1997. New York, United States of America: United Nations (UN)
Australia Vital Registration Death Data 1986 as it appears in United Nations Statistics Division (UNSD). United Nations Demographic Yearbook - Historical Supplement 1997. New York, United States of America: United Nations (UN)
Australia Vital Registration Death Data 1987 as it appears in United Nations Statistics Division (UNSD). United Nations Demographic Yearbook - Historical Supplement 1997. New York, United States of America: United Nations (UN)
Australia Vital Registration Death Data 1988 as it appears in United Nations Statistics Division (UNSD). United Nations Demographic Yearbook - Historical Supplement 1997. New York, United States of America: United Nations (UN)
Australia Vital Registration Death Data 1989 as it appears in United Nations Statistics Division (UNSD). United Nations Demographic Yearbook - Historical Supplement 1997. New York, United States of America: United Nations (UN)
Australia Vital Registration Death Data 1990 as it appears in United Nations Statistics Division (UNSD). United Nations Demographic Yearbook - Historical Supplement 1997. New York, United States of America: United Nations (UN)
Australia Vital Registration Death Data 1991 as it appears in United Nations Statistics Division (UNSD). United Nations Demographic Yearbook - Historical Supplement 1997. New York, United States of America: United Nations (UN)
Australia Vital Registration Death Data 1992 as it appears in United Nations Statistics Division (UNSD). United Nations Demographic Yearbook - Historical Supplement 1997. New York, United States of America: United Nations (UN)

Australia Vital Registration Death Data 1993 as it appears in United Nations Statistics Division (UNSD). United Nations Demographic Yearbook - Historical Supplement 1997. New York, United States of America: United Nations (UN)
Australia Vital Registration Death Data 1994 as it appears in United Nations Statistics Division (UNSD). United Nations Demographic Yearbook - Historical Supplement 1997. New York, United States of America: United Nations (UN)
Australia Vital Registration Death Data 1995 as it appears in United Nations Statistics Division (UNSD). United Nations Demographic Yearbook - Historical Supplement 1997. New York, United States of America: United Nations (UN)
Australia Vital Registration Death Data 1996 as it appears in United Nations Statistics Division (UNSD). United Nations Demographic Yearbook - Historical Supplement 1997. New York, United States of America: United Nations (UN)
United Nations Statistics Division (UNSD). United Nations Demographic Yearbook. New York City, United States of America: United Nations Statistics Division (UNSD)
Australia Vital Registration - Deaths 1979 ICD9 as it appears in World Health Organization (WHO). WHO Mortality Database Version November 2015. Geneva, Switzerland: World Health Organization (WHO)
Australia Vital Registration - Deaths 1980 ICD9 as it appears in World Health Organization (WHO). WHO Mortality Database Version November 2015. Geneva, Switzerland: World Health Organization (WHO)
Australia Vital Registration - Deaths 1981 ICD9 as it appears in World Health Organization (WHO). WHO Mortality Database Version November 2015. Geneva, Switzerland: World Health Organization (WHO)
Australia Vital Registration - Deaths 1982 ICD9 as it appears in World Health Organization (WHO). WHO Mortality Database Version November 2015. Geneva, Switzerland: World Health Organization (WHO)
Australia Vital Registration - Deaths 1983 ICD9 as it appears in World Health Organization (WHO). WHO Mortality Database Version November 2015. Geneva, Switzerland: World Health Organization (WHO)
Australia Vital Registration - Deaths 1984 ICD9 as it appears in World Health Organization (WHO). WHO Mortality Database Version November 2015. Geneva, Switzerland: World Health Organization (WHO)
Australia Vital Registration - Deaths 1985 ICD9 as it appears in World Health Organization (WHO). WHO Mortality Database Version November 2015. Geneva, Switzerland: World Health Organization (WHO)
Australia Vital Registration - Deaths 1986 ICD9 as it appears in World Health Organization (WHO). WHO Mortality Database Version November 2015. Geneva, Switzerland: World Health Organization (WHO)
Australia Vital Registration - Deaths 1987 ICD9 as it appears in World Health Organization (WHO). WHO Mortality Database Version November 2015. Geneva, Switzerland: World Health Organization (WHO)
Australia Vital Registration - Deaths 1988 ICD9 as it appears in World Health Organization (WHO). WHO Mortality Database Version November 2015. Geneva, Switzerland: World Health Organization (WHO)

Australia Vital Registration - Deaths 1989 ICD9 as it appears in World Health Organization (WHO). WHO Mortality Database Version November 2015. Geneva, Switzerland: World Health Organization (WHO)
Australia Vital Registration - Deaths 1990 ICD9 as it appears in World Health Organization (WHO). WHO Mortality Database Version November 2015. Geneva, Switzerland: World Health Organization (WHO)
Australia Vital Registration - Deaths 1991 ICD9 as it appears in World Health Organization (WHO). WHO Mortality Database Version November 2015. Geneva, Switzerland: World Health Organization (WHO)
Australia Vital Registration - Deaths 1992 ICD9 as it appears in World Health Organization (WHO). WHO Mortality Database Version November 2015. Geneva, Switzerland: World Health Organization (WHO)
Australia Vital Registration - Deaths 1993 ICD9 as it appears in World Health Organization (WHO). WHO Mortality Database Version November 2015. Geneva, Switzerland: World Health Organization (WHO)
Australia Vital Registration - Deaths 1994 ICD9 as it appears in World Health Organization (WHO). WHO Mortality Database Version November 2015. Geneva, Switzerland: World Health Organization (WHO)
Australia Vital Registration - Deaths 1995 ICD9 as it appears in World Health Organization (WHO). WHO Mortality Database Version November 2015. Geneva, Switzerland: World Health Organization (WHO)
Australia Vital Registration - Deaths 1996 ICD9 as it appears in World Health Organization (WHO). WHO Mortality Database Version November 2015. Geneva, Switzerland: World Health Organization (WHO)
Australia Vital Registration - Deaths 1997 ICD9 as it appears in World Health Organization (WHO). WHO Mortality Database Version November 2015. Geneva, Switzerland: World Health Organization (WHO)
Australian Bureau of Statistics. Australia Vital Registration - Deaths 2014. Canberra, Australia: Australian Bureau of Statistics
Max Planck Institute for Demographic Research, University of California, Berkeley. Australia Human Mortality Database Deaths Period Data
World Health Organization (WHO). WHO Mortality Database Version March 2017. Geneva, Switzerland: World Health Organization (WHO)
Australia Vital Registration - Deaths 1998 ICD10 as it appears in World Health Organization (WHO). WHO Mortality Database Version October 2017. Geneva, Switzerland: World Health Organization (WHO)
Australia Vital Registration - Deaths 1999 ICD10 as it appears in World Health Organization (WHO). WHO Mortality Database Version October 2017. Geneva, Switzerland: World Health Organization (WHO)
Australia Vital Registration - Deaths 2000 ICD10 as it appears in World Health Organization (WHO). WHO Mortality Database Version October 2017. Geneva, Switzerland: World Health Organization (WHO)
Australia Vital Registration - Deaths 2001 ICD10 as it appears in World Health Organization (WHO). WHO Mortality Database Version October 2017. Geneva, Switzerland: World Health Organization (WHO)
Australia Vital Registration - Deaths 2002 ICD10 as it appears in World Health Organization (WHO). WHO Mortality Database Version October 2017.

Geneva, Switzerland: World Health Organization (WHO)
Australia Vital Registration - Deaths 2003 ICD10 as it appears in World Health Organization (WHO). WHO Mortality Database Version October 2017. Geneva, Switzerland: World Health Organization (WHO)
Australia Vital Registration - Deaths 2004 ICD10 as it appears in World Health Organization (WHO). WHO Mortality Database Version October 2017. Geneva, Switzerland: World Health Organization (WHO)
Australia Vital Registration - Deaths 2006 ICD10 as it appears in World Health Organization (WHO). WHO Mortality Database Version October 2017. Geneva, Switzerland: World Health Organization (WHO)
Australia Vital Registration - Deaths 2007 ICD10 as it appears in World Health Organization (WHO). WHO Mortality Database Version October 2017. Geneva, Switzerland: World Health Organization (WHO)
Australia Vital Registration - Deaths 2008 ICD10 as it appears in World Health Organization (WHO). WHO Mortality Database Version October 2017. Geneva, Switzerland: World Health Organization (WHO)
Australia Vital Registration - Deaths 2009 ICD10 as it appears in World Health Organization (WHO). WHO Mortality Database Version October 2017. Geneva, Switzerland: World Health Organization (WHO)
Australia Vital Registration - Deaths 2010 ICD10 as it appears in World Health Organization (WHO). WHO Mortality Database Version October 2017. Geneva, Switzerland: World Health Organization (WHO)
Australia Vital Registration - Deaths 2011 ICD10 as it appears in World Health Organization (WHO). WHO Mortality Database Version October 2017. Geneva, Switzerland: World Health Organization (WHO)
Australia Vital Registration - Deaths 2012 ICD10 as it appears in World Health Organization (WHO). WHO Mortality Database Version October 2017. Geneva, Switzerland: World Health Organization (WHO)
Australia Vital Registration - Deaths 2013 ICD10 as it appears in World Health Organization (WHO). WHO Mortality Database Version October 2017. Geneva, Switzerland: World Health Organization (WHO)
Australia Vital Registration - Deaths 2014 ICD10 as it appears in World Health Organization (WHO). WHO Mortality Database Version December 2018. Geneva, Switzerland: World Health Organization (WHO)
Australia Vital Registration - Deaths 2015 ICD10 as it appears in World Health Organization (WHO). WHO Mortality Database Version December 2018. Geneva, Switzerland: World Health Organization (WHO)
Australia Vital Registration - Deaths 2016 ICD10 as it appears in World Health Organization (WHO). WHO Mortality Database Version May 2019. Geneva, Switzerland: World Health Organization (WHO)

Supplementary Table 7 GBD data input sources for neonatal health outcomes- all cause mortality

Citation
International Diabetes Institute (IDI). Australia Diabetes, Obesity and Lifestyle Study 1999-2000. Melbourne, Australia: International Diabetes Institute (IDI)
Australian Bureau of Statistics. Australia National Health Survey 1995. Canberra, Australia: Australian Bureau of Statistics
Department of Health and Ageing (Australia), World Health Organization (WHO). Australia World Health Survey 2003. Geneva, Switzerland: World Health Organization (WHO), 2005
McDonnell CM, Harris M, Zacharin MR. Iodine deficiency and goitre in schoolchildren in Melbourne, 2001. Med J Aust. 2003; 178(4): 159-62 as it appears in World Health Organization (WHO). WHO Global Database on Iodine Deficiency. Geneva, Switzerland: World Health Organization (WHO)
World Health Organization (WHO). WHO Global Database on Vitamin A Deficiency. Geneva, Switzerland: World Health Organization (WHO)
United Nations Children's Fund (UNICEF), World Health Organization (WHO). WHO and UNICEF Reported Disease Incidence Time Series. Geneva, Switzerland: World Health Organization (WHO)
Australian Bureau of Statistics. Australia National Health Survey 2001. Canberra, Australia: Australian Bureau of Statistics
Australian Bureau of Statistics. Australia National Health Survey 2004-2005
Australian Bureau of Statistics. Australia National Health Survey 2007-2008. Canberra, Australia: Australian Bureau of Statistics
Australian Bureau of Statistics. Australia Disability, Ageing, and Carers Survey 2003
Australian Bureau of Statistics. Australia Disability, Ageing, and Carers Survey 2009
World Health Organization (WHO). WHO DengueNet. Geneva, Switzerland: World Health Organization (WHO)
Lake FR, Cullen KJ, de Klerk NH, McCall MG, Rosman DL. Atrial fibrillation and mortality in an elderly population. Aust N Z J Med. 1989; 19(4): 321-6
Weatherall D. Sickle Cell and Thalassemias Prevalence Data, Personal Correspondence with David Weatherall. [Unpublished]
Kilkenny M, Stathakis V, Jolley D, Marks R. Maryborough skin health survey: prevalence and sources of advice for skin conditions. Australas J Dermatol. 1998; 39(4): 233-7
Thrift AG, Dewey HM, Sturm JW, Srikanth VK, Gilligan AK, Gall SL, Macdonell RAL, McNeil JJ, Donnan GA. Incidence of stroke subtypes in the North East Melbourne Stroke Incidence Study (NEMESIS): differences between men and women. Neuroepidemiology. 2009; 32(1): 11-8
Islam MS, Anderson CS, Hankey GJ, Hardie K, Carter K, Broadhurst R, Jamrozik K. Trends in Incidence and Outcome of Stroke in Perth, Western Australia During 1989 to 2001. Stroke. 2008; 39(3): 776-82

Thrift AG, Dewey HM, Macdonell RAL, McNeil JJ, Donnan GA. Incidence of the Major Stroke Subtypes: Initial Findings From the North East Melbourne Stroke Incidence Study (NEMESIS). Stroke. 2001; 32(8): 1732-8
Thrift AG, Dewey HM, Macdonell RAL, McNeil JJ, Donnan GA. Stroke Incidence on the East Coast of Australia: The North East Melbourne Stroke Incidence Study (NEMESIS). Stroke. 2000; 31(9): 2087-92
Williams H, Stewart A, Von Mutius E, Cookson W, Anderson HR. Is eczema really on the increase worldwide. J Allergy Clin Immunol. 2008; 121(4): 947-954
Australian Bureau of Statistics. Australia Vital Registration - Deaths 2005
Raasch BA, Buettner PG. Multiple nonmelanoma skin cancer in an exposed Australian population. Int J Dermatol. 2002; 41(10): 652-8
Staples MP, Elwood M, Burton RC, Williams JL, Marks R, Giles GG. Non-melanoma skin cancer in Australia: the 2002 national survey and trends since 1985. Med J Aust. 2006; 184(1): 6-10
Cowan J, Kerr C. Incidence of homozygous beta-thalassaemia in New South Wales, 1961-1976. Med J Aust. 1982; 1(13): 554-6
Gillman A, Steinfort C. Sarcoidosis in Australia. Intern Med J. 2007; 37(6): 356-9
Jg M, Sr H, Jf H. Epidemiology of multiple sclerosis in Australia. With NSW and SA survey results. Med J Aust. 1994; 160(3): 117-22
Hammond SR, de Wytt C, Maxwell IC, Landy PJ, English D, McLeod JG, McCall MG. The epidemiology of multiple sclerosis in Queensland, Australia. J Neurol Sci. 1987; 80(2-3): 185-204
Worldwide variations in the prevalence of asthma symptoms: the International Study of Asthma and Allergies in Childhood (ISAAC). Eur Respir J. 1998; 12(2): 315-35
Lai CK, Beasley R, Crane J, Foliaki S, Shah J, Weiland S. Global variation in the prevalence and severity of asthma symptoms: phase three of the International Study of Asthma and Allergies in Childhood (ISAAC). Thorax. 2009; 64(6): 476-483
Burgess JA, Matheson MC, Gurrin LC, Byrnes GB, Adams KS, Wharton CL, Giles GG, Jenkins MA, Hopper JL, Abramson MJ, Walters EH, Dharmage SC. Factors influencing asthma remission: a longitudinal study from childhood to middle age. Thorax. 2011; 66(6): 508-13
Wilson DH, Adams RJ, Tucker G, Appleton S, Taylor AW, Ruffin RE. Trends in asthma prevalence and population changes in South Australia, 1990-2003. Med J Aust. 2006; 184(5): 226-9
James AL, Knuiman MW, Divitini ML, Hui J, Hunter M, Palmer LJ, Maier G, Musk AW. Changes in the prevalence of asthma in adults since 1966: the Busselton health study. Eur Respir J. 2010; 35(2): 273-278
Davis TM, Yeap BB, Davis WA, Bruce DG. Lipid-lowering therapy and peripheral sensory neuropathy in type 2 diabetes: the Fremantle Diabetes Study. Diabetologia. 2008; 51(4): 562-6
Australian Institute of Health and Welfare, Department of Health and Ageing (Australia), Roy Morgan Research, Social Research Centre (Australia). Australia National Drug Strategy Household Survey 2007
Australian Institute of Health and Welfare, Roy Morgan Research. Australia National Drug Strategy Household Survey 1998

Badawi N, Kurinczuk JJ, Keogh JM, Alessandri LM, O'Sullivan F, Burton PR, Pemberton PJ, Stanley FJ. Antepartum risk factors for newborn encephalopathy: the Western Australian case-control study. BMJ. 1998; 317(7172): 1549-53
Busselton Population Medical Research Institute (Australia). Australia - Busselton Health Study 1981
Simmons D, McKenzie A, Eaton S, Shaw J, Zimmet P. Prevalence of diabetes in rural Victoria. Diabetes Res Clin Pract. 2005; 70(3): 287-90
Chittleborough CR, Taylor AW, Dal Grande E, Gill TK, Grant JF, Adams RJ, Wilson DH, Ruffin RE, North West Adelaide Health Study Team. Gender differences in asthma prevalence: variations with socioeconomic disadvantage. Respirol. 2010; 15(1): 107-14
Ponsonby A-L, Glasgow N, Pezic A, Dwyer T, Ciszek K, Kljakovic M. A temporal decline in asthma but not eczema prevalence from 2000 to 2005 at school entry in the Australian Capital Territory with further consideration of country of birth. Int J Epidemiol. 2008; 37(3): 559-69
Tai A, Volkmer R, Burton A. Association between asthma symptoms and obesity in preschool (4-5 year old) children. J Asthma. 2009; 46(4): 362-5
Tai A, Volkmer R, Burton A. Prevalence of asthma symptoms and atopic disorders in preschool children and the trend over a decade. J Asthma. 2009; 46(4): 343-6
Odhiambo JA, Williams HC, Clayton TO, Robertson CF, Asher MI, ISAAC Phase Three Study Group. Global variations in prevalence of eczema symptoms in children from ISAAC Phase Three. J Allergy Clin Immunol. 2009; 124(6): 1251-1258
Arora M, Schwarz E, Sivaneswaran S, Banks E. Cigarette smoking and tooth loss in a cohort of older Australians: the 45 and up study. J Am Dent Assoc. 2010; 141(10): 1242-9
Dogar F, Kruger E, Dyson K, Tennant M. Oral health of pre-school children in rural and remote Western Australia. Rural Remote Health. 2011; 11(4): 1869
Arora A, Evans RW. Dental caries in children: a comparison of one non-fluoridated and two fluoridated communities in NSW. N S W Public Health Bull. 2010; 21(11-12): 257-62
Powell RN, McEniery TM. The Brisbane Statistical Division Survey of Adult Dental Health 1984. 3. Dental health status and treatment needs. Aust Dent J. 1988; 33(2): 109-15
Heywood AE, Newall AT, Gao Z, Wood JG, Breschkin A, Nicholson S, Gidding HF, Dwyer DE, Gilbert GL, Macintyre CR. Changes in seroprevalence to hepatitis A in Victoria, Australia: a comparison of three time points. Vaccine. 2012; 30(42): 6020-6
Broom AF, Kirby ER, Sibbritt DW, Adams J, Refshauge KM. Back pain amongst mid-age Australian women: A longitudinal analysis of provider use and self-prescribed treatments. Complement Ther Med. 2012; 20(5): 275-82
O'Sullivan PB, Beales DJ, Smith AJ, Straker LM. Low back pain in 17 year olds has substantial impact and represents an important public health disorder: a cross-sectional study. BMC Public Health. 2012; 12(1): 100
Katzenellenbogen JM, Sanfilippo FM, Hobbs MST, Briffa TG, Ridout SC, Knuiman MW, Dimer L, Taylor KP, Thompson PL, Thompson SC. Incidence of and case fatality following acute myocardial infarction in Aboriginal and non-Aboriginal Western Australians (2000-2004): a

linked data study. Heart Lung Circ. 2010; 19(12): 717-25
Crocombe LA, Mejia GC, Koster CR, Slade GD. Comparison of adult oral health in Australia, the USA, Germany and the UK. Aust Dent J. 2009; 54(2): 147-53
Hallett KB, O'Rourke PK. Dental caries experience of preschool children from the North Brisbane region. Aust Dent J. 2002; 47(4): 331-8
Kruger E, Dyson K, Tennant M. Pre-school child oral health in rural Western Australia. Aust Dent J. 2005; 50(4): 258-62
Australia and New Zealand Dialysis and Transplant Registry (ANZDATA). Australia and New Zealand Dialysis and Transplant Registry Annual Report 2009. Adelaide, Australia: Australia and New Zealand Dialysis and Transplant Registry (ANZDATA), 2009
Mitchell PB, Slade T, Andrews G. Twelve-month prevalence and disability of DSM-IV bipolar disorder in an Australian general population survey. Psychol Med. 2004; 34(05): 777-85
Ben-Tovim DI, Walker K, Gilchrist P, Freeman R, Kalucy R, Esterman A. Outcome in patients with eating disorders: a 5-year study. Lancet. 2001; 357(9264): 1254-7
Sawyer MG, Arney FM, Baghurst PA, Clark JJ, Graetz BW, Kosky RJ, Nurcombe B, Patton GC, Prior MR, Raphael B, Rey JM, Whaites LC, Zubrick SR. The mental health of young people in Australia: key findings from the child and adolescent component of the national survey of mental health and well-being. Aust N Z J Psychiatry. 2001; 35(6): 806-14
Norman PE, Flicker L, Almeida OP, Hankey GJ, Hyde Z, Jamrozik K. Cohort Profile: The Health In Men Study (HIMS). Int J Epidemiol. 2009; 38(1): 48-52
Australian Bureau of Statistics, Department of Health and Family Services (Australia). Australia National Survey of Mental Health and Wellbeing 1997
Australian Bureau of Statistics. Australia National Survey of Mental Health and Wellbeing 2007. Canberra, Australia: Australian Bureau of Statistics, 2007
Swift W, Hall W, Teesson M. Cannabis use and dependence among Australian adults: results from the National Survey of Mental Health and Wellbeing. Addiction. 2001; 96(5): 737-48
Coffey C, Carlin JB, Degenhardt L, Lynskey M, Sanci L, Patton GC. Cannabis dependence in young adults: an Australian population study. Addiction. 2002; 97(2): 187-94
Swift W, Coffey C, Carlin JB, Degenhardt L, Patton GC. Adolescent cannabis users at 24 years: trajectories to regular weekly use and dependence in young adulthood. Addiction. 2008; 103(8): 1361-70
Patton GC, Coffey C, Carlin JB, Degenhardt L, Lynskey M, Hall W. Cannabis use and mental health in young people: cohort study. BMJ. 2002; 325(7374): 1195-8
de Marco R, Accordini S, Cerveri I, Corsico A, Sunyer J, Neukirch F, Künzli N, Leynaert B, Janson C, Gislason T, Vermeire P, Svanes C, Anto JM, Burney P, European Community Respiratory Health Survey Study Group. An international survey of chronic obstructive pulmonary disease

in young adults according to GOLD stages. Thorax. 2004; 59(2): 120-125
Buist AS, McBurnie MA, Vollmer WM, Gillespie S, Burney P, Mannino DM, Menezes AM, Sullivan SD, Lee TA, Weiss KB, Jensen RL, Marks GB, Gulsvik A,Nizankowska-Mogilnicka E, BOLD Collaborative Research Group. International variation in the prevalence of COPD (the BOLD Study): a population-based prevalence study. Lancet. 2007; 9589(9589): 741-50
Australia and New Zealand Dialysis and Transplant Registry (ANZDATA). Australia and New Zealand Dialysis and Transplant Registry Annual Report 2008. Adelaide, Australia: Australia and New Zealand Dialysis and Transplant Registry (ANZDATA), 2008
Australia and New Zealand Dialysis and Transplant Registry (ANZDATA). Australia and New Zealand Dialysis and Transplant Registry Annual Report 2007. Adelaide, Australia: Australia and New Zealand Dialysis and Transplant Registry (ANZDATA), 2008
Australia and New Zealand Dialysis and Transplant Registry (ANZDATA). Australia and New Zealand Dialysis and Transplant Registry Annual Report 2006. Adelaide, Australia: Australia and New Zealand Dialysis and Transplant Registry (ANZDATA), 2006
Australia and New Zealand Dialysis and Transplant Registry (ANZDATA). Australia and New Zealand Dialysis and Transplant Registry Annual Report 2005. Adelaide, Australia: Australia and New Zealand Dialysis and Transplant Registry (ANZDATA), 2005
Australia and New Zealand Dialysis and Transplant Registry (ANZDATA). Australia and New Zealand Dialysis and Transplant Registry Annual Report 2004. Adelaide, Australia: Australia and New Zealand Dialysis and Transplant Registry (ANZDATA), 2005
Australia and New Zealand Dialysis and Transplant Registry (ANZDATA). Australia and New Zealand Dialysis and Transplant Registry Annual Report 2003. Adelaide, Australia: Australia and New Zealand Dialysis and Transplant Registry (ANZDATA), 2003
Australia and New Zealand Dialysis and Transplant Registry (ANZDATA). Australia and New Zealand Dialysis and Transplant Registry Annual Report 2002. Adelaide, Australia: Australia and New Zealand Dialysis and Transplant Registry (ANZDATA)
Australia and New Zealand Dialysis and Transplant Registry (ANZDATA). Australia and New Zealand Dialysis and Transplant Registry Annual Report 2001. Adelaide, Australia: Australia and New Zealand Dialysis and Transplant Registry (ANZDATA), 2001
Australia and New Zealand Dialysis and Transplant Registry (ANZDATA). Australia and New Zealand Dialysis and Transplant Registry Annual Report 2000. Adelaide, Australia: Australia and New Zealand Dialysis and Transplant Registry (ANZDATA), 2001
Australia and New Zealand Dialysis and Transplant Registry (ANZDATA). Australia and New Zealand Dialysis and Transplant Registry Annual Report 1999. Adelaide, Australia: Australia and New Zealand Dialysis and Transplant Registry (ANZDATA), 1999
Australia and New Zealand Dialysis and Transplant Registry (ANZDATA). Australia and New Zealand Dialysis and Transplant Registry Annual Report 1998. Adelaide, Australia: Australia and New Zealand Dialysis and Transplant Registry (ANZDATA), 1998
Australia and New Zealand Dialysis and Transplant Registry (ANZDATA). Australia and New Zealand Dialysis and Transplant Registry Annual Report 1997. Adelaide, Australia: Australia and New Zealand Dialysis and Transplant Registry (ANZDATA), 1997
Australia and New Zealand Dialysis and Transplant Registry (ANZDATA). Australia and New Zealand Dialysis and Transplant Registry Annual Report 2010. Adelaide, Australia: Australia and New Zealand Dialysis and Transplant Registry (ANZDATA), 2010

Cheney K, Chen MY, Donovan B. Chlamydia trachomatis infection among antenatal women in Sydney. Aust N Z J Public Health. 2006; 30(1): 85-7
Bateson DJ, Weisberg E, Lota H. Chlamydia trachomatis infection in the family planning clinical setting across New South Wales. Sex Health. 2006; 3(1): 15-20
Lenton J-A, Freedman E, Hoskin K, Knight V, Turley D, Balding B, Kennedy C, Chen MY, McNulty A. Chlamydia trachomatis infection among antenatal women in remote far west New South Wales, Australia. Sex Health. 2007; 4(2): 139-40
Chen MY, Fairley CK, De Guingand D, Hocking J, Tabrizi S, Wallace EM, Grover S, Gurrin L, Carter R, Pirotta M, Garland S. Screening pregnant women for chlamydia: what are the predictors of infection?. Sex Transm Infect. 2009; 85(1): 31-5
Walker J, Fairley CK, Bradshaw CS, Tabrizi SN, Chen MY, Twin J, Taylor N, Donovan B, Kaldor JK, McNamee K, Urban E, Walker S, Currie M, Birden H, Bowden F, Gunn J, Pirotta M, Gurrin L, Harindra V, Garland S, Hocking JS. The difference in determinants of Chlamydia trachomatis and Mycoplasma genitalium in a sample of young Australian women. BMC Infect Dis. 2011; 11: 35
Chan DKY, Cordato D, Karr M, Ong B, Lei H, Liu J, Hung WT. Prevalence of Parkinson's disease in Sydney. Acta Neurol Scand. 2005; 111(1): 7-11
Waite LM, Broe GA, Creasey H, Grayson DA, Cullen JS, O'Toole B, Edelbrock D, Dobson M. Neurodegenerative and other chronic disorders among people aged 75 years and over in the community. Med J Aust. 1997; 167(8): 429-33
McCann SJ, LeCouteur DG, Green AC, Brayne C, Johnson AG, Chan D, McManus ME, Pond SM. The Epidemiology of Parkinson’s Disease in an Australian Population. Neuroepidemiology. 1998; 17(6): 310-7
Lawrence D, Jablensky AV, Holman CD, Pinder TJ. Mortality in Western Australian psychiatric patients. Soc Psychiatry Psychiatr Epidemiol. 2000; 35(8): 341-7
Ruschena D, Mullen PE, Burgess P, Cordner SM, Barry-Walsh J, Drummer OH, Palmer S, Browne C, Wallace C. Sudden death in psychiatric patients. Br J Psychiatry. 1998; 172(4): 331-6
University of Western Australia. Australia National Study of Low Prevalence (Psychotic) Disorders 1997-1998
Bartu A, Freeman NC, Gawthorne GS, Codde JP, Holman CDJ. Mortality in a cohort of opiate and amphetamine users in Perth, Western Australia. Addiction. 2004; 99(1): 53-60
Walker BF, Muller R, Grant WD. Low back pain in Australian adults: prevalence and associated disability. J Manipulative Physiol Ther. 2004; 27(4): 238-44
Grimmer K, Nyland L, Milanese S. Longitudinal investigation of low back pain in Australian adolescents: a five-year study. Physiother Res Int. 2006; 11(3): 161-72
Gidding HF, MacIntyre CR, Burgess MA, Gilbert GL. The seroepidemiology and transmission dynamics of varicella in Australia. Epidemiol Infect. 2003; 131(3): 1085-9
Gilbert GL, Gidding HF, Backhouse J, McIntyre PB. Varicella seroprevalence and vaccine uptake in preschool children. Med J Aust. 2005;

182(1): 42
O'Grady KA, Merianos A, Patel M, Gilbert L. High seroprevalence of antibodies to varicella zoster virus in adult women in a tropical climate. Trop Med Int Health. 2000; 5(10): 732-6
Chant KG, Sullivan EA, Burgess MA, Ferson MJ, Forrest JM, Baird LM, Tudehope DI, Tilse M. Varicella-zoster virus infection in Australia. Aust N Z J Public Health. 1998; 22(4): 413-8
Bittles AH, Petterson BA, Sullivan SG, Hussain R, Glasson EJ, Montgomery PD. The influence of intellectual disability on life expectancy. J Gerontol A Biol Sci Med Sci. 2002; 57(7): M470-472
Ponsonby A-L, Catto-Smith AG, Pezic A, Dupuis S, Halliday J, Cameron D, Morley R, Carlin J, Dwyer T. Association between early-life factors and risk of child-onset Crohn's disease among Victorian children born 1983-1998: a birth cohort study. Inflamm Bowel Dis. 2009; 15(6): 858-66
Wilson J, Hair C, Knight R, Catto-Smith A, Bell S, Kamm M, Desmond P, McNeil J, Connell W. High incidence of inflammatory bowel disease in Australia: a prospective population-based Australian incidence study. Inflamm Bowel Dis. 2010; 16(9): 1550-6
Zutshi A, Eckert KA, Hawthorne G, Taylor AW, Goldney RD. Changes in the prevalence of bipolar disorders between 1998 and 2008 in an Australian population. Bipolar Disord. 2011; 13(2): 182-8
Williams L, Jacka F, Pasco J, Henry M, Dodd S, Nicholson G, Kotowicz M, Berk M. The prevalence of mood and anxiety disorders in Australian women. Australas Psychiatry. 2010; 18(3): 250-5
Cancer Council Victoria (Australia), Department of Health and Ageing (Australia). Australia Secondary Students' Alcohol and Drug Survey 2005
Coomber K, Toumbourou JW, Miller P, Staiger PK, Hemphill SA, Catalano RF. Rural adolescent alcohol, tobacco, and illicit drug use: a comparison of students in Victoria, Australia, and Washington State, United States. J Rural Health. 2011; 27(4): 409-15
Hall W, Teesson M, Lynskey M, Degenhardt L. The 12-month prevalence of substance use and ICD-10 substance use disorders in Australian adults: findings from the National Survey of Mental Health and Well-Being. Addiction. 1999; 94(10): 1541-50
Lynskey M, White V, Hill D, Letcher T, Hall W. Prevalence of illicit drug use among youth: results from the Australian School Students' Alcohol and Drugs Survey. Aust N Z J Public Health. 1999; 23(5): 519-24
Patton GC HM, Rosier MJ, Carlin JB, Caust J, Bowes G. Patterns of common drug use in teenagers. Aust J Public Health. 1995; 19(4): 393-9
Carolan MC, Davey M-A, Biro M, Kealy M. Very advanced maternal age and morbidity in Victoria, Australia: a population based study. BMC Pregnancy Childbirth. 2013; 13(1): 80
Hammond G, Langridge A, Leonard H, Hagan R, Jacoby P, Deklerk N, Pennell C, Stanley F. Changes in risk factors for preterm birth in Western Australia 1984-2006. BJOG. 2013; 120(9): 1051-60
Rozen G, Ugoni AM, Sheehan PM. A new perspective on VBAC: a retrospective cohort study. Women Birth. 2011; 24(1): 3-9
Von Katterfeld B, Li J, McNamara B, Langridge AT. Obstetric profiles of foreign-born women in Western Australia using data linkage, 1998-

2006. Aust N Z J Obstet Gynaecol. 2011; 51(3): 225-32
Chen JS, Ford JB, Roberts CL, Simpson JM, March LM. Pregnancy outcomes in women with juvenile idiopathic arthritis: a population-based study. Rheumatology (Oxford). 2013; 52(6): 1119-25
Roberts CL, Ford JB, Algert CS, Antonsen S, Chalmers J, Cnattingius S, Gokhale M, Kotelchuck M, Melve KK, Langridge A, Morris C, Morris JM, Nassar N, Norman JE, Norrie J, Sørensen HT, Walker R, Weir CJ. Population-based trends in pregnancy hypertension and pre-eclampsia: an international comparative study. BMJ Open. 2011; 1(1): e000101
Thornton C, Dahlen H, Korda A, Hennessy A. The incidence of preeclampsia and eclampsia and associated maternal mortality in Australia from population-linked datasets: 2000-2008. Am J Obstet Gynecol. 2013; 208(6): 476e1-5
Schildkraut V, Alex G, Cameron DJS, Hardikar W, Lipschitz B, Oliver MR, Simpson DM, Catto-Smith AG. Sixty-year study of incidence of childhood ulcerative colitis finds eleven-fold increase beginning in 1990s. Inflamm Bowel Dis. 2013; 19(1): 1-6
Leyden JM, Kleinig TJ, Newbury J, Castle S, Cranefield J, Anderson CS, Crotty M, Whitford D, Jannes J, Lee A, Greenhill J. Adelaide stroke incidence study: declining stroke rates but many preventable cardioembolic strokes. Stroke. 2013; 44(5): 1226-31
Simpson S Jr, Pittas F, van der Mei I, Blizzard L, Ponsonby A-L, Taylor B. Trends in the epidemiology of multiple sclerosis in Greater Hobart, Tasmania: 1951 to 2009. J Neurol Neurosurg Psychiatry. 2011; 82(2): 180-7
Beran RG, Hall L, Michelazzi J. An accurate assessment of the prevalence ratio of epilepsy adequately adjusted by influencing factors. Neuroepidemiology. 1985; 4(2): 71-81
D'Souza WJ, Fryer JL, Quinn SJ, Taylor BV, Ficker DM, O'Brien TJ, Pearce NE, Cook MJ. The Tasmanian Epilepsy Register – a community-based cohort. Background and methodology for patient recruitment from the Australian national prescription database. Neuroepidemiology. 2007; 29(3-4): 255-63
Harvey AS, Nolan T, Carlin JB. Community-based study of mortality in children with epilepsy. Epilepsia. 1993; 34(4): 597-603
Hawthorne G, Goldney R, Taylor AW. Depression prevalence: is it really increasing?. Aust N Z J Psychiatry. 2008; 42(7): 606-16
Jorm AF, Henderson AS, Kay DWK, Jacomb PA. Mortality in relation to dementia, depression and social integration in an elderly community sample. Int J Geriatr Psychiatry. 1991; 6(1): 11-May
Wilhelm K, Mitchell P, Slade T, Brownhill S, Andrews G. Prevalence and correlates of DSM-IV major depression in an Australian national survey. J Affect Disord. 2003; 75(2): 155-62
Goldney RD, Eckert KA, Hawthorne G, Taylor AW. Changes in the prevalence of major depression in an Australian community sample between 1998 and 2008. Aust N Z J Psychiatry. 2010; 44(10): 901-10
Parner ET, Thorsen P, Dixon G, de Klerk N, Leonard H, Nassar N, Bourke J, Bower C, Glasson EJ. A comparison of autism prevalence trends in Denmark and Western Australia. J Autism Dev Disord. 2011; 41(12): 1601-8
Australian Institute of Health and Welfare, Roy Morgan Research. Australia National Drug Strategy Household Survey 2010

McEvoy PM, Grove R, Slade T. Epidemiology of anxiety disorders in the Australian general population: findings of the 2007 Australian National Survey of Mental Health and Wellbeing. Aust N Z J Psychiatry. 2011; 45(11): 957-67
National Drug and Alcohol Research Centre, University of New South Wales. Australia Estimating the Number of Current Regular Heroin Users in NSW and Australia 1997-2002. Randwick, Australia: National Drug and Alcohol Research Centre, University of New South Wales, 2004
Degenhardt L, Randall D, Hall W, Butler T, Law M, Burns L. Mortality among persons receiving opioid pharmacotherapy in New South Wales, Australia, 1985-2006: risk factors for elevated mortality and estimated mortality reductions of a statewide programme across 20 years
World Health Organization (WHO). WHO Tuberculosis Case Notifications. Geneva, Switzerland: World Health Organization (WHO)
Graetz BW, Sawyer MG, Hazell PL, Arney F, Baghurst P. Validity of DSM-IVADHD subtypes in a nationally representative sample of Australian children and adolescents. J Am Acad Child Adolesc Psychiatry. 2001; 40(12): 1410-7
Mein J, Bowden FJ. A profile of inpatient STD-related pelvic inflammatory disease in the Top End of the Northern Territory of Australia. Med J Aust. 1997; 166(9): 464-7
Kwan KSH, Giele CM, Combs B, Mak DB. Improvement in antenatal testing for sexually transmissible infections and blood-borne viruses in Western Australian hospitals, 2007 to 2010. Sex Health. 2012; 9(4): 349-54
VanNewkirk MR, Weih L, McCarty CA, Taylor HR. Cause-specific prevalence of bilateral visual impairment in Victoria, Australia: the Visual Impairment Project. Ophthalmology. 2001; 108(5): 960-7
Australian Congenital Malformation Monitoring System Data 1993-1997 - WHO as it appears in European Surveillance of Congenital Anomalies (EUROCAT), International Centre on Birth Defects, World Health Organization (WHO). World Atlas of Birth Defects. 2nd ed. Geneva, Switzerland: World Health Organization (WHO), 2003
Roberts CL, Ford JB, Algert CS, Bell JC, Simpson JM, Morris JM. Trends in adverse maternal outcomes during childbirth: a population-based study of severe maternal morbidity. BMC Pregnancy Childbirth. 2009; 7
Carmody C, Garland S, Kent HE, Denham I. Prevalence of type-specific herpes simplex virus antibodies in partners of patients with recurrent genital herpes. Venereology. 2001; 14(4): 160
Song B, Dwyer DE, Mindel A. HSV type specific serology in sexual health clinics: use, benefits, and who gets tested. Sex Transm Infect. 2004; 80(2): 113-7
Mindel A, Taylor J, Tideman RL, Seifert C, Berry G, Wagner K, Page J, Marks C, Trudinger B, Cunningham A. Neonatal herpes prevention: a minor public health problem in some communities. Sex Transm Infect. 2000; 76(4): 287-91
Taylor HR, Livingston PM, Stanislavsky YL, McCarty CA. Visual impairment in Australia: distance visual acuity, near vision, and visual field findings of the Melbourne Visual Impairment Project. Am J Ophthalmol. 1997; 123(3): 328-37
Adamson SJ, Alessandri LM, Badawi N, Burton PR, Pemberton PJ, Stanley F. Predictors of neonatal encephalopathy in full-term infants. BMJ. 1995; 311(7005): 598-602

Jacobs SE, Morley CJ, Inder TE, Stewart MJ, Smith KR, McNamara PJ, Wright IMR, Kirpalani HM, Darlow BA, Doyle LW, Infant Cooling Evaluation Collaboration. Whole-body hypothermia for term and near-term newborns with hypoxic-ischemic encephalopathy: a randomized controlled trial. Arch Pediatr Adolesc Med. 2011; 165(8): 692-700
Carli G, Reiger I, Evans N. One-year neurodevelopmental outcome after moderate newborn hypoxic ischaemic encephalopathy. J Paediatr Child Health. 2004; 40(4): 217-20
Dixon G, Badawi N, Kurinczuk JJ, Keogh JM, Silburn SR, Zubrick SR, Stanley FJ. Early developmental outcomes after newborn encephalopathy. Pediatrics. 2002; 109(1): 26-33
Gray PH, Tudehope DI, Masel JP, Burns YR, Mohay HA, O'Callaghan MJ, Williams GM. Perinatal hypoxic-ischaemic brain injury: prediction of outcome. Dev Med Child Neurol. 1993; 35(11): 965-73
Yeo CL, Tudehope DI. Outcome of resuscitated apparently stillborn infants: a ten year review. J Paediatr Child Health. 1994; 30(2): 129-33
Australia - Victoria Birth Defects Registry Data 2001 - ICBDMS as it appears in International Clearinghouse for Birth Defects Monitoring Systems. International Clearinghouse for Birth Defects Monitoring Systems Annual Report 2003. Rome, Italy: International Centre on Birth Defects
Sutton DL, Lyle DM, Pierce JP. Incidence and prevalence of insulin-dependent diabetes mellitus in the zero- to 19-years' age-group in Sydney. Med J Aust. 1989; 151(3): 140-6
Byrne A. Nine-year follow-up of 86 consecutive patients treated with methadone in general practice, Sydney, Australia. Drug Alcohol Rev. 2000; 19(2): 153-8
Digiusto E, Shakeshaft A, Ritter A, O'Brien S, Mattick RP, NEPOD Research Group. Serious adverse events in the Australian National Evaluation of Pharmacotherapies for Opioid Dependence (NEPOD). Addiction. 2004; 99(4): 450-60
Hall WD, Ross JE, Lynskey MT, Law MG, Degenhardt LJ. How many dependent heroin users are there in Australia?. Med J Aust. 2000; 173(10): 528-31
Law MG, Lynskey M, Ross J, Hall W. Back-projection estimates of the number of dependent heroin users in Australia. Addiction. 2001; 96(3): 433-43
Tait RJ, Ngo HTT, Hulse GK. Mortality in heroin users 3 years after naltrexone implant or methadone maintenance treatment. J Subst Abuse Treat. 2008; 35(2): 116-24
Teesson M, Baillie A, Lynskey M, Manor B, Degenhardt L. Substance use, dependence and treatment seeking in the United States and Australia: a cross-national comparison. Drug Alcohol Depend. 2006; 81(2): 149-55
Teesson M, Mills K, Ross J, DarkeS, Williamson A, Havard A. The impact of treatment on 3 years' outcome for heroin dependence: findings from the Australian Treatment Outcome Study (ATOS). Addiction. 2008; 103(1): 80-88
Australasian Association of Cancer Registries, Australian Institute of Health and Welfare. Australia Cancer Incidence and Mortality Books

2012. Canberra, Australia: Australian Institute of Health and Welfare, 2012
Sawyer M, Miller-Lewis L, Clark J. The Mental Health of 13-17 Year-Olds in Australia: Findings from the National Survey of Mental Health and Well-Being. J Youth Adolescence. 2007; 36(2): 185-94
Budtz-Olsen O, Kidson C. Absence of red cell enzyme deficiency in Australian Aborigines. Nature. 1961; 192: 765
Smith MB. The incidence of glucose-6-phosphate dehydrogenase deficiency in a population of Greek, Italian and Yugoslav origin in Australia. Med J Aust. 1976; 2(13): 485-6
Australia - Victoria Birth Defects Registry Data 2002 - ICBDMS as it appears in International Clearinghouse for Birth Defects Monitoring Systems. International Clearinghouse for Birth Defects Monitoring Systems Annual Report 2004. Rome, Italy: International Centre on Birth Defects, 2006
Australia - Western Australian Birth Defects Registry Data 2002 - ICBDMS as it appears in International Clearinghouse for Birth Defects Monitoring Systems. International Clearinghouse for Birth Defects Monitoring Systems Annual Report 2004. Rome, Italy: International Centre on Birth Defects, 2006
Australia - Western Australian Birth Defects Registry Data 2003 - ICBDSR as it appears in International Clearinghouse for Birth Defects Surveillance and Research. International Clearinghouse for Birth Defects Surveillance and Research Annual Report 2005. Rome, Italy: International Clearinghouse for Birth Defects Surveillance and Research, 2007
Australia - Victoria Birth Defects Registry Data 2003 - ICBDSR as it appears in International Clearinghouse for Birth Defects Surveillance and Research. International Clearinghouse for Birth Defects Surveillance and Research Annual Report 2005. Rome, Italy: International Clearinghouse for Birth Defects Surveillance and Research, 2007
Australia - Western Australian Birth Defects Registry Data 2004 - ICBDSR as it appears in International Clearinghouse for Birth Defects Surveillance and Research. International Clearinghouse for Birth Defects Surveillance and Research Annual Report 2006. Rome, Italy: International Clearinghouse for Birth Defects Surveillance and Research, 2007
Australia - Victoria Birth Defects Registry Data 2004 - ICBDSR as it appears in International Clearinghouse for Birth Defects Surveillance and Research. International Clearinghouse for Birth Defects Surveillance and Research Annual Report 2006. Rome, Italy: International Clearinghouse for Birth Defects Surveillance and Research, 2007
Australia - Victoria Birth Defects Registry Data 2005 - ICBDSR as it appears in International Clearinghouse for Birth Defects Surveillance and Research. International Clearinghouse for Birth Defects Surveillance and Research Annual Report 2007. Rome, Italy: International Clearinghouse for Birth Defects Surveillance and Research, 2008
Australia - Western Australian Birth Defects Registry Data 2005 - ICBDSR as it appears in International Clearinghouse for Birth Defects Surveillance and Research. International Clearinghouse for Birth Defects Surveillance and Research Annual Report 2007. Rome, Italy: International Clearinghouse for Birth Defects Surveillance and Research, 2008

Australia - Western Australian Birth Defects Registry Data 2007 - ICBDSR as it appears in International Clearinghouse for Birth Defects Surveillance and Research. International Clearinghouse for Birth Defects Surveillance and Research Annual Report 2009. Rome, Italy: International Clearinghouse for Birth Defects Surveillance and Research
Australia - Victoria Birth Defects Registry Data 2007 - ICBDSR as it appears in International Clearinghouse for Birth Defects Surveillance and Research. International Clearinghouse for Birth Defects Surveillance and Research Annual Report 2009. Rome, Italy: International Clearinghouse for Birth Defects Surveillance and Research
Australia - Western Australian Register of Developmental Anomalies Data 2008 - ICBDSR as it appears in International Clearinghouse for Birth Defects Surveillance and Research. International Clearinghouse for Birth Defects Surveillance and Research Annual Report 2010. Rome, Italy: International Clearinghouse for Birth Defects Surveillance and Research, 2011
Australia - Victoria Birth Defects Registry Data 2008 - ICBDSR as it appears in International Clearinghouse for Birth Defects Surveillance and Research. International Clearinghouse for Birth Defects Surveillance and Research Annual Report 2010. Rome, Italy: International Clearinghouse for Birth Defects Surveillance and Research, 2011
Australia - Western Australian Register of Developmental Anomalies Data 2009 - ICBDSR as it appears in International Clearinghouse for Birth Defects Surveillance and Research. International Clearinghouse for Birth Defects Surveillance and Research Annual Report 2011. Rome, Italy: International Clearinghouse for Birth Defects Surveillance and Research, 2012
Australia - Western Australian Register of Developmental Anomalies Data 2010 - ICBDSR as it appears in International Clearinghouse for Birth Defects Surveillance and Research. International Clearinghouse for Birth Defects Surveillance and Research Annual Report 2012. Rome, Italy: International Clearinghouse for Birth Defects Surveillance and Research, 2013
Lowe P, Kovacs G, Howlett D. Incidence of polycystic ovaries and polycystic ovary syndrome amongst women in Melbourne, Australia. Aust N Z J Obstet Gynaecol. 2005; 45(1): 17-9
March WA, Moore VM, Willson KJ, Phillips DIW, Norman RJ, Davies MJ. The prevalence of polycystic ovary syndrome in a community sample assessed under contrasting diagnostic criteria. Hum Reprod. 2010; 25(2): 544-51
Parkin DM, International Agency for Research on Cancer, International Association of Cancer Registries. Cancer Incidence in Five Continents. Vol. I to VIII. Lyon, France, IARCPress, 2005
Australian Congenital Malformation Monitoring System Data 1981 - ICBDMS as it appears in International Clearinghouse for Birth Defects Monitoring Systems. Congenital Malformations Worldwide: A Report from the International Clearinghouse for Birth Defects Monitoring Systems. Amsterdam, Netherlands: Elsevier, 1991
Australian Congenital Malformation Monitoring System Data 1984 - ICBDMS as it appears in International Clearinghouse for Birth Defects Monitoring Systems. Congenital Malformations Worldwide: A Report from the International Clearinghouse for Birth Defects Monitoring Systems. Amsterdam, Netherlands: Elsevier, 1991

Australian Congenital Malformation Monitoring System Data 1987 - ICBDMS as it appears in International Clearinghouse for Birth Defects Monitoring Systems. Congenital Malformations Worldwide: A Report from the International Clearinghouse for Birth Defects Monitoring Systems. Amsterdam, Netherlands: Elsevier, 1991
Australian Congenital Malformation Monitoring System Data 1988 - ICBDMS as it appears in International Clearinghouse for Birth Defects Monitoring Systems. Congenital Malformations Worldwide: A Report from the International Clearinghouse for Birth Defects Monitoring Systems. Amsterdam, Netherlands: Elsevier, 1991
Australian Congenital Malformation Monitoring System Data 1985 - ICBDMS as it appears in International Clearinghouse for Birth Defects Monitoring Systems. Congenital Malformations Worldwide: A Report from the International Clearinghouse for Birth Defects Monitoring Systems. Amsterdam, Netherlands: Elsevier, 1991
Australian Congenital Malformation Monitoring System Data 1982 - ICBDMS as it appears in International Clearinghouse for Birth Defects Monitoring Systems. Congenital Malformations Worldwide: A Report from the International Clearinghouse for Birth Defects Monitoring Systems. Amsterdam, Netherlands: Elsevier, 1991
Australian Congenital Malformation Monitoring System Data 1983 - ICBDMS as it appears in International Clearinghouse for Birth Defects Monitoring Systems. Congenital Malformations Worldwide: A Report from the International Clearinghouse for Birth Defects Monitoring Systems. Amsterdam, Netherlands: Elsevier, 1991
Australian Congenital Malformation Monitoring System Data 1986 - ICBDMS as it appears in International Clearinghouse for Birth Defects Monitoring Systems. Congenital Malformations Worldwide: A Report from the International Clearinghouse for Birth Defects Monitoring Systems. Amsterdam, Netherlands: Elsevier, 1991
Brennan D, Spencer A, Slade G. Prevalence of periodontal conditions among public-funded dental patients in Australia. Aust Dent J. 2001; 46(2): 114-21
Wright FA, Hammond RH, Lewis JM. Changes in periodontal conditions of adults from Melbourne, Australia. Int Dent J. 1994; 44(3): 207-14
Teesson M, Hall W, Slade T, Mills K, Grove R, Mewton L, Baillie A, Haber P. Prevalence and correlates of DSM-IV alcohol abuse and dependence in Australia: findings of the 2007 National Survey of Mental Health and Wellbeing. Addiction. 2010; 105(12): 2085-94
Fleming J. The epidemiology of alcohol use in Australian women: findings from a national survey of women's drinking. Addiction. 1996; 91(9): 1325-34
Proudfoot H, Baillie AJ, Teesson M. The structure of alcohol dependence in the community. Drug Alcohol Depend. 2006; 81(1): 21-6
Mehta P, Kifley A, Wang JJ, Rochtchina E, Mitchell P, Sue CM. Population prevalence and incidence of Parkinson's disease in an Australian community. Intern Med J. 2007; 37(12): 812-4
Allen K, Riley M, Goldfeld S, Halliday J. Estimating the prevalence of fetal alcohol syndrome in Victoria using routinely collected administrative data. Aust N Z J Public Health. 2007; 31(1): 62-6

Bell R, Lumley J. Alcohol consumption, cigarette smoking and fetal outcome in Victoria, 1985. Community Health Stud. 1989; 13(4): 484-91
Bower C, Silva D, Henderson TR, Ryan A, Rudy E. Ascertainment of birth defects: the effect on completeness of adding a new source of data. J Paediatr Child Health. 2000; 36(6): 574-6
Durvasula S, Beange H, Baker W. Mortality of people with intellectual disability in northern Sydney. J Intellect Dev Disabil. 2002; 27(4): 255-64
Elliott EJ, Payne J, Morris A, Haan E, Bower C. Fetal alcohol syndrome: a prospective national surveillance study. Arch Dis Child. 2008; 93(9): 732-7
Harris KR, Bucens IK. Prevalence of fetal alcohol syndrome in the Top End of the Northern Territory. J Paediatr Child Health. 2003; 39(7): 528-33
Slade T, Johnston A, Oakley Browne MA, Andrews G, Whiteford H. 2007 National Survey of Mental Health and Wellbeing: methods and key findings. Aust N Z J Psychiatry. 2009; 43(7): 594-605
Hankey GJ. Guillain-Barré syndrome in Western Australia, 1980-1985. Med J Aust. 1987; 146(3): 130-3
Storey E, Cook M, Peppard R, Newton-John H, Byrne E. Guillain-Barré syndrome and related conditions in Victorian teaching hospitals 1980-84. Aust N Z J Med. 1989; 19(6): 687-93
OzFoodNet Working Group. Monitoring the incidence and causes of diseases potentially transmitted by food in Australia: annual report of the OzFoodNet Network, 2009. Commun Dis Intell Q Rep. 2010; 34(4): 396-426
Ferlay J, Parkin DM, Curado MP, Bray F, Edwards B, Shin HR and Forman D. Cancer Incidence in Five Continents Time Trends, Volumes I to X and Volume XI: IARC CancerBase No. 9 [Internet]. Lyon, France: International Agency for Research on Cancer; 2010. Available from: http://ci5.iarc.fr
Stein AN, Britt H, Harrison C, Conway EL, Cunningham A, Macintyre CR. Herpes zoster burden of illness and health care resource utilisation in the Australian population aged 50 years and older. Vaccine. 2009; 27(4): 520-9
Weichbold V, Nekahm-Heis D, Welzl-Mueller K. Universal newborn hearing screening and postnatal hearing loss. Pediatrics. 2006; 117(4): e631-636
Australia National Nutrition Survey 1995-1996 as it appears in World Health Organization (WHO). WHO Global Database on Child Growth and Malnutrition. Geneva, Switzerland: World Health Organization (WHO)
Australia National Children's Nutrition and Physical Activity Survey 2007 as it appears in World Health Organization (WHO). WHO Global Database on Child Growth and Malnutrition. Geneva, Switzerland: World Health Organization (WHO)
Waite LM, Broe GA, Grayson DA, Creasey H. The incidence of dementia in an Australian community population: the Sydney Older Persons Study. Int J Geriatr Psychiatry. 2001; 16(7): 680-9
World Health Organization (WHO). Global leprosy situation, 2004. Wkly Epidemiol Rec. 2005: 80(13): 118-24
World Health Organization (WHO). Global leprosy situation, 2005. Wkly Epidemiol Rec. 2005: 80(34): 289-95

World Health Organization (WHO). Global leprosy situation, 2007. Wkly Epidemiol Rec. 2007: 82(25): 225-32
World Health Organization (WHO). Global leprosy situation, 2009. Wkly Epidemiol Rec. 2009: 84(33): 333-40
World Health Organization (WHO). Global leprosy situation, 2010. Wkly Epidemiol Rec. 2010: 85(35): 337-48
World Health Organization (WHO). Global leprosy situation, 2012. Wkly Epidemiol Rec. 2012: 87(34): 317-28
World Health Organization (WHO). Global leprosy: update on the 2012 situation. Wkly Epidemiol Rec. 2013: 88(35): 365-79
World Health Organization (WHO). Leprosy update, 2011. Wkly Epidemiol Rec. 2011: 86(36): 389-99
Tideman RL, Taylor J, Marks C, Seifert C, Berry G, Trudinger B, Cunningham A, Mindel A. Sexual and demographic risk factors for herpes simplex type 1 and 2 in women attending an antenatal clinic. Sex Transm Infect. 2001; 77(6): 413-5
World Health Organization (WHO). WHO Global Health Observatory - Cholera: Number of Reported Cases by Country. Geneva, Switzerland: World Health Organization (WHO)
Bolisetty S, Daftary A, Ewald D, Knight B, Wheaton G. Congenital heart defects in Central Australia. Med J Aust. 2004; 180(12): 614-7
Bower C, Ramsay JM. Congenital heart disease: a 10 year cohort. J Paediatr Child Health. 1994; 30(5): 414-8
Chew C, Halliday JL, Riley MM, Penny DJ. Population-based study of antenatal detection of congenital heart disease by ultrasound examination. Ultrasound Obstet Gynecol. 2007; 29(6): 619-24
Fitzgerald P, Leonard H, Pikora TJ, Bourke J, Hammond G. Hospital admissions in children with down syndrome: experience of a population-based cohort followed from birth. PLoS One. 2013; 8(8): e70401
Kidd SA, Lancaster PA, McCredie RM. The incidence of congenital heart defects in the first year of life. J Paediatr Child Health. 1993; 29(5): 344-9
Malone Q. Mortality and survival of the Down's syndrome population in Western Australia. J Ment Defic Res. 1988; 32 (Pt 1): 59-65
World Health Organization Regional Office for the Western Pacific (WPRO-WHO). Number of Reported Cases of Dengue Fever and Dengue Hemorrhagic Fever in the Western Pacific Region, by Country 2000. Manila, Philippines: World Health Organization Regional Office for the Western Pacific (WPRO-WHO)
World Health Organization Regional Office for the Western Pacific (WPRO-WHO). Number of Reported Cases of Dengue Fever and Dengue Hemorrhagic Fever in the Western Pacific Region, by Country 2001. Manila, Philippines: World Health Organization Regional Office for the Western Pacific (WPRO-WHO)
World Health Organization Regional Office for the Western Pacific (WPRO-WHO). Number of Reported Cases of Dengue Fever and Dengue Hemorrhagic Fever in the Western Pacific Region, by Country 2002. Manila, Philippines: World Health Organization Regional Office for the Western Pacific (WPRO-WHO)
World Health Organization Regional Office for the Western Pacific (WPRO-WHO). Number of Reported Cases of Dengue Fever and Dengue

Hemorrhagic Fever in the Western Pacific Region, by Country 2003. Manila, Philippines: World Health Organization Regional Office for the Western Pacific (WPRO-WHO)
World Health Organization Regional Office for the Western Pacific (WPRO-WHO). Number of Reported Cases of Dengue Fever and Dengue Hemorrhagic Fever in the Western Pacific Region, by Country 2004. Manila, Philippines: World Health Organization Regional Office for the Western Pacific (WPRO-WHO)
World Health Organization Regional Office for the Western Pacific (WPRO-WHO). Number of Reported Cases of Dengue Fever and Dengue Hemorrhagic Fever in the Western Pacific Region, by Country 2005. Manila, Philippines: World Health Organization Regional Office for the Western Pacific (WPRO-WHO)
World Health Organization Regional Office for the Western Pacific (WPRO-WHO). Number of Reported Cases of Dengue Fever and Dengue Hemorrhagic Fever in the Western Pacific Region, by Country 2006. Manila, Philippines: World Health Organization Regional Office for the Western Pacific (WPRO-WHO)
World Health Organization Regional Office for the Western Pacific (WPRO-WHO). Number of Reported Cases of Dengue Fever and Dengue Hemorrhagic Fever in the Western Pacific Region, by Country 2007. Manila, Philippines: World Health Organization Regional Office for the Western Pacific (WPRO-WHO)
World Health Organization Regional Office for the Western Pacific (WPRO-WHO). Number of Reported Cases of Dengue Fever and Dengue Hemorrhagic Fever in the Western Pacific Region, by Country 2008. Manila, Philippines: World Health Organization Regional Office for the Western Pacific (WPRO-WHO)
World Health Organization Regional Office for the Western Pacific (WPRO-WHO). Number of Reported Cases of Dengue Fever and Dengue Hemorrhagic Fever in the Western Pacific Region, by Country 2009. Manila, Philippines: World Health Organization Regional Office for the Western Pacific (WPRO-WHO)
World Health Organization Regional Office for the Western Pacific (WPRO-WHO). Number of Reported Cases of Dengue Fever and Dengue Hemorrhagic Fever in the Western Pacific Region, by Country 2010. Manila, Philippines: World Health Organization Regional Office for the Western Pacific (WPRO-WHO)
Australia and New Zealand Dialysis and Transplant Registry (ANZDATA). Australia and New Zealand Dialysis and Transplant Registry 1990
Australia and New Zealand Dialysis and Transplant Registry (ANZDATA). Australia and New Zealand Dialysis and Transplant Registry Data 1995
May M, Daley A, Donath S, Isaacs D, on b. Early onset neonatal meningitis in Australia and New Zealand, 1992-2002. Arch Dis Child Fetal Neonatal Ed. 2005; 90(4): F324-F327
Hanna JN, Wild BE. Bacterial meningitis in children under five years of age in Western Australia. Med J Aust. 1991; 155(3): 160-4
Australian Bureau of Statistics, Australian Dental Association, Department of Health, Housing and Community Services (Australia). Australia National Oral Health Survey 1987-1988

Teede HJ, Joham AE, Paul E, Moran LJ, Loxton D, Jolley D, Lombard C. Longitudinal weight gain in women identified With polycystic ovary syndrome: Results of an observational study in young women. Obesity (Silver Spring). 2013; 21(8): 1526-32
European Community Respiratory Health Survey Study Group. European Community Respiratory Health Survey 1992-2003 as provided by the Global Burden of Disease 2010 Respiratory Diseases Expert Group. [Unpublished]
Medcalf GW. Busselton dental survey 1978. Aust Dent J. 1981; 26(5): 304-8
Australia and New Zealand Dialysis and Transplant Registry (ANZDATA). Australia and New Zealand Dialysis and Transplant Registry Annual Report 2011. Adelaide, Australia: Australia and New Zealand Dialysis and Transplant Registry (ANZDATA), 2011
Australia and New Zealand Dialysis and Transplant Registry (ANZDATA). Australia and New Zealand Dialysis and Transplant Registry Annual Report 2012. Adelaide, Australia: Australia and New Zealand Dialysis and Transplant Registry (ANZDATA), 2012
Huppatz C, Durrheim DN, Levi C, Dalton C, Williams D, Clements MS, Kelly PM. Etiology of Encephalitis in Australia, 1990-2007. Emerg Infect Dis. 2009; 15(9): 1359-65
Beange H, McElduff A, Baker W. Medical disorders of adults with mental retardation: a population study. Am J Ment Retard. 1995; 99(6): 595-604
Martin PE, Koplin JJ, Eckert JK, Lowe AJ, Ponsonby A-L, Osborne NJ, Gurrin LC, Robinson MN, Hill DJ, Tang MLK, Dharmage SC, Allen KJ, HealthNuts Study Investigators. The prevalence and socio-demographic risk factors of clinical eczema in infancy: a population-based observational study. Clin Exp Allergy. 2013; 43(6): 642-51
Clucas DB, Carville KS, Connors C, Currie BJ, Carapetis JR, Andrews RM. Disease burden and health-care clinic attendances for young children in remote aboriginal communities of northern Australia. Bull World Health Organ. 2008; 86(4): 275-81
Australia - Blue Mountains Eye Study 1992-1994 as it appears in Bourne R, Global Burden of Disease Vision Loss Expert Group. Vision Loss Database - Survey Data on Vision Loss by Severity and Etiology. [Unpublished]
Chitsulo L, Engels D, Montresor A, Savioli L. The global status of schistosomiasis and its control. Acta Trop. 2000; 77(1): 41-51
Williams H, Robertson C, Stewart A, Aït-Khaled N, Anabwani G, Anderson R, Asher I, Beasley R, Björkstén B, Burr M, Clayton T, Crane J, Ellwood P, Keil U, Lai C, Mallol J, Martinez F, Mitchell E, Montefort S, Pearce N, Shah J, Sibbald B, Strachan D, von Mutius E, Weiland SK. Worldwide variations in the prevalence of symptoms of atopic eczema in the International Study of Asthma and Allergies in Childhood. J Allergy Clin Immunol. 1999; 103(1 Pt 1): 125-38
Simons LA, Simons J, McCallum J, Friedlander Y. Impact of smoking, diabetes and hypertension on survival time in the elderly: the Dubbo Study. Med J Aust. 2005; 182(5): 219-23
Crofts N, Cooper G, Stewart T, Kiely P, Coghlan P, Hearne P, Hocking J. Exposure to hepatitis A virus among blood donors, injecting drug users and prison entrants in Victoria. J Viral Hepat. 1997; 4(5): 333-8
Quan HL, Blizzard CL, Venn AJ, Thuy AB, Luc PH, Sharman JE. Blood pressure and body mass index: a comparison of the associations in the

Caucasian and Asian populations. Hypertens Res. 2012; 35(5): 523-30
Dobson AJ, McElduff P, Heller R, Alexander H, Colley P, D'Este K. Changing Patterns of Coronary Heart Disease in the Hunter Region of New South Wales, Australia. J Clin Epidemiol. 1999; 52(8): 761-71
Amin J, Gidding H, Gilbert G, Backhouse J, Kaldor J, Dore G, Burgess M. Hepatitis C prevalence - a nationwide survey. Commun Dis Intell. 2004; 28(4): 517-21
Ashwell MJ, Cossart YE. An autopsy survey of hepatitis B in Sydney. Pathology. 1995; 27(1): 43-7
Campbell DH, Plant AJ, Sargent JW, Mock PA, Barrett ER, Archer KH. Hepatitis B infection of children in a mixed-race community in western New South Wales. Med J Aust. 1991; 154(4): 253-6
Gidding HF, Warlow M, MacIntyre CR, Backhouse J, Gilbert GL, Quinn HE, McIntyre PB. The impact of a new universal infant and school-based adolescent hepatitis B vaccination program in Australia. Vaccine. 2007; 25(51): 8637-41
Moore DJ, Bucens MR, Holman CD, Ott AK, Wells JI. Prenatal screening for markers of hepatitis B in aboriginal mothers resident in non-metropolitan Western Australia. Med J Aust. 1987; 147(11-12): 557-8
O'Sullivan BG, Gidding HF, Law M, Kaldor JM, Gilbert GL, Dore GJ. Estimates of chronic hepatitis B virus infection in Australia, 2000. Aust N Z J Public Health. 2004; 28(3): 212-6
Patterson F, Bumak J, Batey R. Changing prevalence of hepatitis B virus in urbanized Australian Aborigines. J Gastroenterol Hepatol. 1993; 8(5): 410-3
Tawk HM, Vickery K, Bisset L, Selby W, Cossart YE, Infection in Endoscopy Study Group. The impact of hepatitis B vaccination in a Western country: recall of vaccination and serological status in Australian adults. Vaccine. 2006; 24(8): 1095-106
Doyle LW, Roberts G, Anderson PJ, Victorian Infant Collaborative Study Group. Outcomes at age 2 years of infants < 28 weeks' gestational age born in Victoria in 2005. J Pediatr. 2010; 156(1): 49-53
Marks R, Jolley D, Dorevitch AP, Selwood TS. The incidence of non-melanocytic skin cancers in an Australian population: results of a five-year prospective study. Med J Aust. 1989; 150(9): 475-8
English DR, Kricker A, Heenan PJ, Randell PL, Winter MG, Armstrong BK. Incidence of non-melanocytic skin cancer in Geraldton, Western Australia. Int J Cancer. 1997; 73(5): 629-33
Kay DWK, Henderson AS, Scott R, Wilson J, Rickwood D, Grayson DA. Dementia and depression among the elderly living in the Hobart community: the effect of the diagnostic criteria on the prevalence rates. Psychol Med. 1985; 15(04): 771-88
Australia - Victoria Birth Defects Registry 2006 - ICBDSR as it appears in International Clearinghouse for Birth Defects Surveillance and Research. International Clearinghouse for Birth Defects Surveillance and Research Annual Report 2008. Rome, Italy: International Clearinghouse for Birth Defects Surveillance and Research
Australia - Western Australia Birth Defects Registry 2006 - ICBDSR as it appears in International Clearinghouse for Birth Defects Surveillance

and Research. International Clearinghouse for Birth Defects Surveillance and Research Annual Report 2008. Rome, Italy: International Clearinghouse for Birth Defects Surveillance and Research
Katzenellenbogen JM, Vos T, Somerford P, Begg S, Semmens JB, Codde JP. Excess Mortality Rates for Estimating the Non-Fatal Burden of Stroke in Western Australia: A Data Linkage Study. Cerebrovasc Dis. 2010; 30(1): 57-64
The ACROSS Group. Epidemiology of Aneurysmal Subarachnoid Hemorrhage in Australia and New Zealand Incidence and Case Fatality From the Australasian Cooperative Research on Subarachnoid Hemorrhage Study (ACROSS). Stroke. 2000; 31(8): 1843–1850
Cobiac LJ, Magnus A, Lim S, Barendregt JJ, Carter R, Vos T. Which interventions offer best value for money in primary prevention of cardiovascular disease?. PLoS One. 2012; 7(7): e41842
The Victorian Infant Collaborative Study Group. Improved outcome into the 1990s for infants weighing 500-999 g at birth. Arch Dis Child Fetal Neonatal Ed. 1997; 77(2): F91–94
Roberts G, Anderson PJ, De Luca C, Doyle LW, Victorian Infant Collaborative Study Group. Changes in neurodevelopmental outcome at age eight in geographic cohorts of children born at 22-27 weeks' gestational age during the 1990s. Arch Dis Child Fetal Neonatal Ed. 2010; 95(2): F90–94
Doyle LW, Roberts G, Anderson PJ, Victorian Infant Collaborative Study Group. Changing long-term outcomes for infants 500-999 g birth weight in Victoria, 1979-2005. Arch Dis Child Fetal Neonatal Ed. 2011; 96(6): F443–47
Baker IDI Heart and Diabetes Institute, International Diabetes Institute (IDI), Monash University (Australia), University of Melbourne. Australia Diabetes, Obesity and Lifestyle Study 2011-2012
Astbury J, Orgill AA, Bajuk B, Yu VY. Neurodevelopmental outcome, growth and health of extremely low-birthweight survivors: how soon can we tell?. Dev Med Child Neurol. 1990; 32(7): 582-9
World Health Organization Regional Office for Europe (EURO-WHO). Health Behaviour in School-aged Children: WHO Collaborative Cross-National survey/study (HBSC) 2010
Australia and New Zealand Dialysis and Transplant Registry (ANZDATA). Australia and New Zealand Dialysis and Transplant Registry Annual Report 2013. Adelaide, Australia: Australia and New Zealand Dialysis and Transplant Registry (ANZDATA), 2013
Australia and New Zealand Dialysis and Transplant Registry (ANZDATA). Australia and New Zealand Dialysis and Transplant Registry Annual Report 2014. Adelaide, Australia: Australia and New Zealand Dialysis and Transplant Registry (ANZDATA), 2014
Australian Institute of Health and Welfare, University of Adelaide. Australia National Survey of Adult Oral Health 2004-2006
Han N, Wright ST, O'Connor CC, Hoy J, Ponnampalavanar S, Grotowski M, Zhao HX, Kamarulzaman A, Australian HIV Observational Database (AHOD), TREAT Asia HIV Observational Database (TAHOD). HIV and aging: insights from the Asia Pacific HIV Observational Database (APHOD). HIV Med. 2015; 16(3): 152–60
Li SQ, Guthridge SL, Eswara Aratchige P, Lowe MP, Wang Z, Zhao Y, Krause V. Dementia prevalence and incidence among the Indigenous and

non-Indigenous populations of the Northern Territory. Med J Aust. 2014; 200(8): 465–9
Department of Health (Australia). Australia Notifications of Chlamydial Infection by Age Group and Sex 1991-2017. Canberra, Australia: Department of Health (Australia)
Department of Health (Australia). Australia Notifications of Gonorrhea Infection by Age Group and Sex 1991-2017. Canberra, Australia: Department of Health (Australia)
Department of Health (Australia). Australia Notifications of Syphilis Infection by Age Group and Sex 1991-2017. Canberra, Australia: Department of Health (Australia)
Australian Bureau of Statistics, Australian Health Survey Reference Group, Roy Morgan Research, University of Western Australia. Australia Child and Adolescent Survey of Mental Health and Wellbeing 2013-2014
Pendleton A, Galic M, Clarke C, Ng SP, Ledesma E, Ramakrishnan G, Liu Y. Impact of rotavirus vaccination in Australian children below 5 years of age: a database study. Hum Vaccin Immunother. 2013; 9(8): 1617-25
Davis WA, Norman PE, Bruce DG, Davis TM. Predictors, consequences and costs of diabetes-related lower extremity amputation complicating type 2 diabetes: the Fremantle Diabetes Study. Diabetologia. 2006; 49(11): 2634-41
Ng SC, Tang W, Ching JY, Wong M, Chow CM, Hui AJ, Wong TC, Leung VK, Tsang SW, Yu HH, Li MF, Ng KK, Kamm MA, Studd C, Bell S, Leong R, de Silva HJ, Kasturiratne A, Mufeena MNF, Ling KL, Ooi CJ, Tan PS, Ong D, Goh KL, Hilmi I, Pisespongsa P, Manatsathit S, Rerknimitr R, Aniwan S, Wang YF, Ouyang Q, Zeng Z, Zhu Z, Chen MH, Hu PJ, Wu K, Wang X, Simadibrata M, Abdullah M, Wu JC, Sung JJY, Chan FKL, Asia–Pacific Crohn’s and Colitis Epidemiologic Study (ACCESS) Study Group. Incidence and phenotype of inflammatory bowel disease based on results from the Asia-pacific Crohn’s and colitis epidemiology study. Gastroenterology. 2013; 145(1): 158–65e2
Martin AJ, Darlow BA, Salt A, Hague W, Sebastian L, Mann K, Tarnow-Mordi W. Identification of infants with major cognitive delay using parental report. Dev Med Child Neurol. 2012; 54(3): 254-9
Byford S, Weaver E, Anstey C. Has the incidence of hypoxic ischaemic encephalopathy in Queensland been reduced with improved education in fetal surveillance monitoring?. Aust N Z J Obstet Gynaecol. 2014; 54(4): 348-53
Wilson-Ching M, Molloy CS, Anderson VA, Burnett A, Roberts G, Cheong JL, Doyle LW, Anderson PJ. Attention difficulties in a contemporary geographic cohort of adolescents born extremely preterm/extremely low birth weight. J Int Neuropsychol Soc. 2013; 19(10): 1097-108
Reidy N, Morgan A, Thompson DK, Inder TE, Doyle LW, Anderson PJ. Impaired language abilities and white matter abnormalities in children born very preterm and/or very low birth weight. J Pediatr. 2013; 162(4): 719-24
Hutchinson EA, De Luca CR, Doyle LW, Roberts G, Anderson PJ. School-age outcomes of extremely preterm or extremely low birth weight children. Pediatrics. 2013; 131(4): e1053-61
Doyle LW, Anderson PJ, Haslam R, Lee KJ, Crowther C. School-age outcomes of very preterm infants after antenatal treatment with magnesium sulfate vs placebo. JAMA. 2014; 312(11): 1105-13

Bolisetty S, Dhawan A, Abdel-Latif M, Bajuk B, Stack J, Lui K. Intraventricular hemorrhage and neurodevelopmental outcomes in extreme preterm infants. Pediatrics. 2014; 133(1): 55-62
Cretikos M, Mayne D, Reynolds R, Spokes P, Madeddu D. Testing-adjusted Chlamydia Notification Trends in New South Wales, Australia, 2000 to 2010. West Pac Surveill Response J. 2014; 5(3): 7–17
Yeung AH, Temple-Smith M, Fairley CK, Vaisey AM, Guy R, Law MG, Low N, Bingham AL, Gunn J, Kaldor J, Donovan B, Hocking JS. Chlamydia prevalence in young attenders of rural and regional primary care services in Australia: a cross-sectional survey. Med J Aust. 2014; 200(3): 170–5
Moore HC, de Klerk N, Richmond P, Lehmann D. A retrospective population-based cohort study identifying target areas for prevention of acute lower respiratory infections in children. BMC Public Health. 2010; 757
Williams P, Gracey M, Smith P. Hospitalization of aboriginal and non-aboriginal patients for respiratory tract diseases in Western Australia, 1988-1993. Int J Epidemiol. 1997; 26(4): 797–805
Cunningham AL, Taylor R, Taylor J, Marks C, Shaw J, Mindel A. Prevalence of infection with herpes simplex virus types 1 and 2 in Australia: a nationwide population based survey. Sex Transm Infect. 2006; 82(2): 164–8
Page A, Taylor R, Richters J, Shaw J, Taylor J, Cunningham A, Mindel A. Upstairs and downstairs: socio-economic and gender interactions in herpes simplex virus type 2 seroprevalence in australia. Sex Transm Dis. 2009; 36(6): 344–9
Sasadeusz JJ, Silvers JE, Kent HE, Devenish W, Hocking J, Garland SM. Prevalence of HSV-2 antibody in a Melbourne antenatal population attending a tertiary obstetric hospital. Aust N Z J Obstet Gynaecol. 2008; 48(3): 266–72
Allen KL, Byrne SM, Oddy WH, Crosby RD. DSM-IV-TR and DSM-5 eating disorders in adolescents: prevalence, stability, and psychosocial correlates in a population-based sample of male and female adolescents. J Abnorm Psychol. 2013; 122(3): 720–32
Degenhardt L, Larney S, Chan G, Dobbins T, Weier M, Roxburgh A, Hall W, McKetin R. Estimating the number of regular and dependent methamphetamine users in Australia, 2002-2014. Med J Aust
Giles GG, Marks R, Foley P. Incidence of non-melanocytic skin cancer treated in Australia. Br Med J (Clin Res Ed). 1988; 296(6614): 13–7
World Health Organization Regional Office for the Western Pacific (WPRO-WHO). WPRO Dengue Situation Update, 08 January 2013. Manila, Philippines: World Health Organization Regional Office for the Western Pacific (WPRO-WHO), 2013
World Health Organization Regional Office for the Western Pacific (WPRO-WHO). WPRO Dengue Situation Update, 28 January 2014. Manila, Philippines: World Health Organization Regional Office for the Western Pacific (WPRO-WHO), 2014
World Health Organization Regional Office for the Western Pacific (WPRO-WHO). WPRO Dengue Situation Update 457, 27 January 2015. Manila, Philippines: World Health Organization Regional Office for the Western Pacific (WPRO-WHO), 2015
Smirnov A, Kemp R, Wells H, Legosz M, Najman JM. Using population screening for recruitment of young adults engaged in illicit drug use: methodological issues and sampling outcomes. Soc Sci Res. 2014; 45: 89–97

de Wytt CN, Jackson RV, Hockings GI, Joyner JM, Strakosch CR. Polyneuropathy in Australian outpatients with type II diabetes mellitus. J Diabetes Complicat. 1999; 13(2): 74–8
Robertson CF, Roberts MF, Kappers JH, others. Asthma prevalence in Melbourne schoolchildren: have we reached the peak?. Med J Aust. 2004; 180(6): 273–6
Toelle BG, Ng K, Belousova E, Salome CM, Peat JK, Marks GB. Prevalence of asthma and allergy in schoolchildren in Belmont, Australia: three cross sectional surveys over 20 years. BMJ. 2004; 328(7436): 386–7
Fitzpatrick JP, Latimer J, Carter M, Oscar J, Ferreira ML, Carmichael Olson H, Lucas BR, Doney R, Salter C, Try J, Hawkes G, Fitzpatrick E, Hand M, Watkins RE, Martiniuk AL, Bower C, Boulton J, Elliott EJ. Prevalence of fetal alcohol syndrome in a population-based sample of children living in remote Australia: the Lililwan Project. J Paediatr Child Health. 2015; 51(4): 450–7
King Edward Memorial Hospital for Women (Australia). Australia - Western Australia Report of the Birth Defects Registry 1980-2009. Subiaco, Australia: King Edward Memorial Hospital for Women (Australia), 2010
Women's and Children's Hospital (Australia). Australia - South Australian Birth Defects Register Annual Report 2007. Adelaide, Australia: Women's and Children's Hospital (Australia), 2010
King Edward Memorial Hospital for Women (Australia). Australia - Western Australia Report of the Birth Defects Registry 1980-2005. Subiaco, Australia: King Edward Memorial Hospital for Women (Australia), 2006
Women's and Children's Hospital (Australia). Australia - South Australian Birth Defects Register Annual Report 2002. Adelaide, Australia: Women's and Children's Hospital (Australia), 2005
Women's and Children's Hospital (Australia). Australia - South Australian Birth Defects Register Annual Report 2006. Adelaide, Australia: Women's and Children's Hospital (Australia), 2009
Women's and Children's Hospital (Australia). Australia - South Australian Birth Defects Register Annual Report 2011. Adelaide, Australia: Women's and Children's Hospital (Australia), 2015
Tran F, Stone M, Huang CY, Lloyd M, Woodhead HJ, Elliott KD, Crock PA, Howard NJ, Craig ME. Population-based incidence of diabetes in Australian youth aged 10-18?yr: increase in type 1 diabetes but not type 2 diabetes. Pediatr Diabetes. 2014; 15(8): 585-90
Chong JW, Craig ME, Cameron FJ, Clarke CF, Rodda CP, Donath SM, Werther GA. Marked increase in type 1 diabetes mellitus incidence in children aged 0-14 yr in Victoria, Australia, from 1999 to 2002. Pediatr Diabetes. 2007; 8(2): 67-73
Craig ME, Femia G, Broyda V, Lloyd M, Howard NJ. Type 2 diabetes in Indigenous and non-Indigenous children and adolescents in New South Wales. Med J Aust. 2007; 186(10): 497-9
Williams ED, Tapp RJ, Magliano DJ, Shaw JE, Zimmet PZ, Oldenburg BF. Health behaviours, socioeconomic status and diabetes incidence: the Australian Diabetes Obesity and Lifestyle Study (AusDiab). Diabetologia. 2010; 53(12): 2538-45
Taplin CE, Craig ME, Lloyd M, Taylor C, Crock P, Silink M, Howard NJ. The rising incidence of childhood type 1 diabetes in New South Wales,

1990-2002. Med J Aust. 2005; 183(5): 243-6
Haynes A, Bower C, Bulsara MK, Jones TW, Davis EA. Continued increase in the incidence of childhood Type 1 diabetes in a population-based Australian sample (1985-2002). Diabetologia. 2004; 47(5): 866-70
Kelly HA, Russell MT, Jones TW, Byrne GC. Dramatic increase in incidence of insulin dependent diabetes mellitus in Western Australia. Med J Aust. 1994; 161(7): 426-9
Craig ME, Howard NJ, Silink M, Chan A. The rising incidence of childhood type 1 diabetes in New South Wales, Australia. J Pediatr Endocrinol Metab. 2000; 13(4): 363-72
Li M, McDermott R. Smoking, poor nutrition, and sexually transmitted infections associated with pelvic inflammatory disease in remote North Queensland Indigenous communities, 1998-2005. BMC Womens Health. 2015; 31
World Health Organization (WHO). WHO Mortality Database Version November 2015. Geneva, Switzerland: World Health Organization (WHO)
Australian Institute of Health and Welfare. Australia's Mothers and Babies 1991. Canberra, Australia: Australian Institute of Health and Welfare, 1994
Australian Institute of Health and Welfare. Australia's Mothers and Babies 1992. Canberra, Australia: Australian Institute of Health and Welfare, 1995
Australian Institute of Health and Welfare. Australia's Mothers and Babies 1993. Canberra, Australia: Australian Institute of Health and Welfare, 1996
Australian Institute of Health and Welfare. Australia's Mothers and Babies 1994. Canberra, Australia: Australian Institute of Health and Welfare, 1997
Australian Institute of Health and Welfare. Australia's Mothers and Babies 1995. Canberra, Australia: Australian Institute of Health and Welfare, 1997
Australian Institute of Health and Welfare. Australia's Mothers and Babies 1996. Canberra, Australia: Australian Institute of Health and Welfare, 1999
Australian Institute of Health and Welfare, National Perinatal Epidemiology and Statistics Unit (NPESU), University of New South Wales. Australia's Mothers and Babies 1997. Canberra, Australia: Australian Institute of Health and Welfare, 1999
Australian Institute of Health and Welfare, National Perinatal Epidemiology and Statistics Unit (NPESU), University of New South Wales. Australia's Mothers and Babies 1998. Canberra, Australia: Australian Institute of Health and Welfare, 2000
Australian Institute of Health and Welfare, National Perinatal Epidemiology and Statistics Unit (NPESU), University of New South Wales. Australia's Mothers and Babies 1999. Canberra, Australia: Australian Institute of Health and Welfare, 2001
Australian Institute of Health and Welfare, National Perinatal Epidemiology and Statistics Unit (NPESU), University of New South Wales.

Australia's Mothers and Babies 2000. Canberra, Australia: Australian Institute of Health and Welfare, 2003
Australian Institute of Health and Welfare, National Perinatal Epidemiology and Statistics Unit (NPESU), University of New South Wales. Australia's Mothers and Babies 2001. Canberra, Australia: Australian Institute of Health and Welfare, 2004
Australian Institute of Health and Welfare, National Perinatal Epidemiology and Statistics Unit (NPESU), University of New South Wales. Australia's Mothers and Babies 2002. Canberra, Australia: Australian Institute of Health and Welfare, 2004
Australian Institute of Health and Welfare, National Perinatal Epidemiology and Statistics Unit (NPESU), University of New South Wales. Australia's Mothers and Babies 2003. Canberra, Australia: Australian Institute of Health and Welfare, 2005
Australian Institute of Health and Welfare, National Perinatal Epidemiology and Statistics Unit (NPESU), University of New South Wales. Australia's Mothers and Babies 2004. Canberra, Australia: Australian Institute of Health and Welfare, 2006
Australian Institute of Health and Welfare, National Perinatal Epidemiology and Statistics Unit (NPESU), University of New South Wales. Australia's Mothers and Babies 2005. Canberra, Australia: Australian Institute of Health and Welfare, 2007
Australian Institute of Health and Welfare, National Perinatal Epidemiology and Statistics Unit (NPESU), University of New South Wales. Australia's Mothers and Babies 2006. Canberra, Australia: Australian Institute of Health and Welfare, 2008
Australian Institute of Health and Welfare, National Perinatal Epidemiology and Statistics Unit (NPESU), University of New South Wales. Australia's Mothers and Babies 2007. Canberra, Australia: Australian Institute of Health and Welfare, 2009
Australian Institute of Health and Welfare, National Perinatal Epidemiology and Statistics Unit (NPESU), University of New South Wales. Australia's Mothers and Babies 2008. Canberra, Australia: Australian Institute of Health and Welfare, 2010
Australian Institute of Health and Welfare, National Perinatal Epidemiology and Statistics Unit (NPESU), University of New South Wales. Australia's Mothers and Babies 2010. Canberra, Australia: Australian Institute of Health and Welfare, 2012
Australian Institute of Health and Welfare, National Perinatal Epidemiology and Statistics Unit (NPESU), University of New South Wales. Australia's Mothers and Babies 2009. Canberra, Australia: Australian Institute of Health and Welfare, 2011
Australian Institute of Health and Welfare, National Perinatal Epidemiology and Statistics Unit (NPESU), University of New South Wales. Australia's Mothers and Babies 2011. Canberra, Australia: Australian Institute of Health and Welfare, 2013
Australian Institute of Health and Welfare, National Perinatal Epidemiology and Statistics Unit (NPESU), University of New South Wales. Australia's Mothers and Babies 2012. Canberra, Australia: Australian Institute of Health and Welfare, 2014
World Health Organization (WHO). Global leprosy update, 2013; reducing disease burden. Wkly Epidemiol Rec. 2014; 89(36): 389-400
World Health Organization (WHO). Global leprosy update, 2014: need for early case detection. Wkly Epidemiol Rec. 2015; 90(36): 461–74
Australia - Western Australia Register of Developmental Anomalies 2011 - ICBDSR as it appears in International Clearinghouse for Birth Defects Surveillance and Research. International Clearinghouse for Birth Defects Surveillance and Research Annual Report 2013. Rome, Italy: International Clearinghouse for Birth Defects Surveillance and Research, 2015

Australia and New Zealand Dialysis and Transplant Registry (ANZDATA). Australia and New Zealand Dialysis and Transplant Registry Annual Report 2015. Adelaide, Australia: Australia and New Zealand Dialysis and Transplant Registry (ANZDATA), 2016
Smith KJ, Blizzard L, McNaughton SA, Gall SL, Dwyer T, Venn AJ. Daily eating frequency and cardiometabolic risk factors in young Australian adults: cross-sectional analyses. Br J Nutr. 2012; 108(6): 1086–94
Chittleborough CR, Baldock KL, Phillips PJ, Taylor AW, North West Adelaide Health Study Team. Achievement of management targets associated with incident and long-term diagnosed diabetes among a representative population sample. Diabetes Res Clin Pract. 2010; 88(3): 322–7
Chow EP, Dutt K, Fehler G, Denham I, Chen MY, Batrouney C, Peel J, Read TR, Bradshaw CS, Fairley CK. Duration of syphilis symptoms at presentations in men who have sex with men in Australia: are current public health campaigns effective?. Epidemiol Infect. 2016; 144(1): 113-22
Cystic Echinococcosis Endemicity Estimates identified through systematic review and personal communication, as provided by GBD 2015 expert. [Unpublished]
Morgan VA, Mitchell PB, Jablensky AV. The epidemiology of bipolar disorder: sociodemographic, disability and service utilization data from the Australian National Study of Low Prevalence (Psychotic) Disorders. Bipolar Disord. 2005; 7(4): 326-37 as it appears in Ferrari AJ, Saha S, McGrath JJ, Norman R, Baxter AJ, Vos T, Whiteford HA. Health states for schizophrenia and bipolar disorder within the Global Burden of Disease 2010 Study. Popul Health Metr. 2012; 10(1): 16
World Health Organization (WHO). Global leprosy update, 2015: time for action, accountability and inclusion. Wkly Epidemiol Rec. 2016; 92(35): 405–20
Australia - Western Australian Register of Developmental Anomalies Data 2012 - ICBDSR as it appears in International Clearinghouse for Birth Defects Surveillance and Research. International Clearinghouse for Birth Defects Surveillance and Research Annual Report 2014. Rome, Italy: International Clearinghouse for Birth Defects Surveillance and Research, 2016
Queensland Health. Australia - Queensland Community Diarrhoeal Illness Among Adults and Young Children 2001
Scallan E, Majowicz SE, Hall G, Banerjee A, Bowman CL, Daly L, Jones T, Kirk MD, Fitzgerald M, Angulo FJ. Prevalence of diarrhoea in the community in Australia, Canada, Ireland, and the United States. Int J Epidemiol. 2005; 34(2): 454–60
Almeida OP, Hankey GJ, Yeap BB, Golledge J, Norman PE, Flicker L. Mortality among people with severe mental disorders who reach old age: a longitudinal study of a community-representative sample of 37,892 men. PLoS One. 2014; 9(10): e111882
Ford JB, Patterson JA, Seeho SK, Roberts CL. Trends and outcomes of postpartum haemorrhage, 2003-2011. BMC Pregnancy Childbirth. 2015; 15: 334
Roberts CL, Algert CS, Morris JM, Ford JB. Increased planned delivery contributes to declining rates of pregnancy hypertension in Australia: a population-based record linkage study. BMJ Open. 2015; 5(10): e009313

Wang YA, Chughtai AA, Farquhar CM, Pollock W, Lui K, Sullivan EA. Increased incidence of gestational hypertension and preeclampsia after assisted reproductive technology treatment. Fertil Steril. 2016; 105(4): 920-926e2
Kearns TM, Speare R, Cheng AC, McCarthy J, Carapetis JR, Holt DC, Currie BJ, Page W, Shield J, Gundjirryirr R, Bundhala L, Mulholland E, Chatfield M, Andrews RM. Impact of an Ivermectin Mass Drug Administration on Scabies Prevalence in a Remote Australian Aboriginal Community. PLoS Negl Trop Dis. 2015; 9(10): e0004151
Leach AJ, Wigger C, Beissbarth J, Woltring D, Andrews R, Chatfield MD, Smith-Vaughan H, Morris PS. General health, otitis media, nasopharyngeal carriage and middle ear microbiology in Northern Territory Aboriginal children vaccinated during consecutive periods of 10-valent or 13-valent pneumococcal conjugate vaccines. Int J Pediatr Otorhinolaryngol. 2016; 86: 224-32
Tasani M, Tong SY, Andrews RM, Holt DC, Currie BJ, Carapetis JR, Bowen AC. The Importance of Scabies Coinfection in the Treatment Considerations for Impetigo. Pediatr Infect Dis J. 2016; 35(4): 374-8
Osborne NJ, Ukoumunne OC, Wake M, Allen KJ. Prevalence of eczema and food allergy is associated with latitude in Australia. J Allergy Clin Immunol. 2012; 129(3): 865-7
Levison J, Halliday R, Holland AJ, Walker K, Williams G, Shi E, Badawi N. A population-based study of congenital diaphragmatic hernia outcome in New South Wales and the Australian Capital Territory, Australia, 1992-2001. J Pediatr Surg. 2006; 41(6): 1049-53
Giele H, Giele C, Bower C, Allison M. The incidence and epidemiology of congenital upper limb anomalies: a total population study. J Hand Surg Am. 2001; 26(4): 628-34
Studd C, Cameron G, Beswick L, Knight R, Hair C, McNeil J, Desmond P, Wilson J, Connell W, Bell S. Never underestimate inflammatory bowel disease: High prevalence rates and confirmation of high incidence rates in Australia. J Gastroenterol Hepatol. 2016; 31(1): 81-6
Maxwell S, Bower C, O'Leary P. Impact of prenatal screening and diagnostic testing on trends in Down syndrome births and terminations in Western Australia 1980 to 2013. Prenat Diagn. 2015; 35(13): 1324-30
Glasson EJ, Jacques A, Wong K, Bourke J, Leonard H. Improved Survival in Down Syndrome over the Last 60 Years and the Impact of Perinatal Factors in Recent Decades. J Pediatr. 2016; 169: 214-20e1
Quinn B, Stoové M, Dietze P. One-year changes in methamphetamine use, dependence and remission in a community-recruited cohort. J Subst Use. 2016; 21(3): 279–86
Williams LJ, Jacka FN, Pasco JA, Coulson CE, Quirk SE, Stuart AL, Berk M. The prevalence and age of onset of psychiatric disorders in Australian men. Aust N Z J Psychiatry. 2016; 50(7): 678–84
James S, Pezic A, Ponsonby AL, Lafferty A, Glasgow N, Ciszek K, Kljakovic M, Douglas K. Obesity and asthma at school entry: co-morbidities and temporal trends. J Paediatr Child Health. 2013; 49(4): E273-80
Almeida OP, McCaul K, Hankey GJ, Yeap BB, Golledge J, Flicker L. Affective Disorders, Psychosis and Dementia in a Community Sample of Older Men with and without Parkinson's Disease. PLoS One. 2016; 11(9): e0163781

Lee MG, Brizard CP, Galati JC, Iyengar AJ, Rakhra SS, Konstantinov IE, Pflaumer A, d'Udekem Y. Outcomes of patients born with single-ventricle physiology and aortic arch obstruction: the 26-year Melbourne experience. J Thorac Cardiovasc Surg. 2014; 148(1): 194-201
Toumbourou JW, Evans-Whipp TJ, Smith R, Hemphill SA, Herrenkohl TI, Catalano RF. Adolescent Predictors and Environmental Correlates of Young Adult Alcohol Use Problems. Addiction. 2014; 109(3): 417–24
Atkins R, Polkinghorne K, Chadban S, Chronic Kidney Disease Prognosis Consortium (CKD-PC). Australia Diabetes, Obesity and Lifestyle (AusDiab) Chronic Kidney Disease Prevalence Data 1999-2000 [Unpublished]
Collin M, Trinder S, Minutillo C, Rao S, Dickinson J, Samnakay N. A modern era comparison of right versus left sided congenital diaphragmatic hernia outcomes. J Pediatr Surg. 2016; 51(9): 1409-13
Smith GH, Glasson M. Intestinal atresia: factors affecting survival. Aust N Z J Surg. 1989; 59(2): 151-6
Tu CG, Khurana S, Couper R, Ford AW. Kasai hepatoportoenterostomy in South Australia: a case for 'centralized decentralization'. ANZ J Surg. 2015; 85(11): 865-8
Kong JY, Yeo KT, Abdel-Latif ME, Bajuk B, Holland AJ, Adams S, Jiwane A, Heck S, Yeong M, Lui K, Oei JL. Outcomes of infants with abdominal wall defects over 18years. J Pediatr Surg. 2016; 1644-9
Curcio AL, Mak AS. Adolescent Drinking and Delinquent Activities: Associations and Gender Differences. J Psychol Couns Sch. 2016; 26(1): 100–14
Kirby Institute, University of New South Wales. Australia HIV, Viral Hepatitis and Sexually Transmissible Infections Annual Surveillance Report 2016. Darlinghurst, Australia: Kirby Institute, University of New South Wales, 2016
Australia and New Zealand Dialysis and Transplant Registry (ANZDATA). Australia and New Zealand Dialysis and Transplant Registry Annual Report 2016. Adelaide, Australia: Australia and New Zealand Dialysis and Transplant Registry (ANZDATA), 2017
MacIntyre CR, McIntyre PB, Cagney M. Community-based estimates of incidence and risk factors for childhood pneumonia in Western Sydney. Epidemiol Infect. 2003; 131(3): 1091–6
Harris EL, Minutillo C, Hart S, Warner TM, Ravikumara M, Nathan EA, Dickinson JE. The long term physical consequences of gastroschisis. J Pediatr Surg. 2014; 49(10): 1466–70
Chen Y, Glass K, Liu B, Riley TV, Korda R, Kirk MD. A population-based longitudinal study of Clostridium difficile infection-related hospitalization in mid-age and older Australians. Epidemiol Infect. 2017; 145(3): 575–82
World Health Organization (WHO). WHO Global Project on Anti-Tuberculosis Drug Resistance Surveillance Data 1988-2015
Ribbons K, Lea R, Tiedeman C, Mackenzie L, Lechner-Scott J. Ongoing increase in incidence and prevalence of multiple sclerosis in Newcastle, Australia: A 50-year study. Mult Scler. 2016
Kricker A, English DR, Randell PL, Heenan PJ, Clay CD, Delaney TA, Armstrong BK. Skin cancer in Geraldton, Western Australia: a survey of incidence and prevalence. Med J Aust. 1990; 152(8): 399-407

Baillie AJ, Teesson M. Continuous, categorical and mixture models of DSM-IV alcohol and cannabis use disorders in the Australian community. Addiction. 2010; 105(7): 1246–53
Teesson M, Slade T, Swift W, Mills K, Memedovic S, Mewton L, Grove R, Newton N, Hall W. Prevalence, correlates and comorbidity of DSM-IV Cannabis Use and Cannabis Use Disorders in Australia. Aust N Z J Psychiatry. 2012; 46(12): 1182–92
Marks GB, Bai JUN, Simpson SE, Sullivan EA, Stewart GJ. Incidence of tuberculosis among a cohort of tuberculin-positive refugees in Australia: reappraising the estimates of risk. Am J Respir Crit Care Med. 2000; 162(5): 1851–4
Daniels S, Robson D, Flatley C, Kumar S. Demographic characteristics and pregnancy outcomes in adolescents - Experience from an Australian perinatal centre. Aust N Z J Obstet Gynaecol. 2017
Jayaratnam S, Burton A, Connan KF, de Costa C. Maternal 'near miss' at Royal Darwin Hospital: An analysis of severe maternal morbidity at an Australian regional tertiary maternity unit. Aust N Z J Obstet Gynaecol. 2016; 56(4): 381-6\
Foo XY, Greer RM, Kumar S. Impact of Maternal Body Mass Index on Intrapartum and Neonatal Outcomes in Brisbane, Australia, 2007 to 2013. Birth. 2016; 43(4): 358-365
Lack BM, Smith RM, Arundell MJ, Homer CS. Narrowing the Gap? Describing women's outcomes in Midwifery Group Practice in remote Australia. Women Birth. 2016; 29(5): 465-470
Brodaty H, Aerts L, Crawford JD, Heffernan M, Kochan NA, Reppermund S, Kang K, Maston K, Draper B, Trollor JN, Sachdev PS. Operationalizing the Diagnostic Criteria for Mild Cognitive Impairment: The Salience of Objective Measures in Predicting Incident Dementia. Am J Geriatr Psychiatry. 2017; 25(5): 485-497
Noguchi N, Blyth FM, Waite LM, Naganathan V, Cumming RG, Handelsman DJ, Seibel MJ, Le Couteur DG. Prevalence of the Geriatric Syndromes and Frailty in Older Men Living in the Community: The Concord Health and Ageing in Men Project. Australas J Ageing. 2016; 35(4): 255-61
Tai A, Tran H, Roberts M, Clarke N, Wilson J, Robertson CF. The association between childhood asthma and adult chronic obstructive pulmonary disease. Thorax. 2014; 69(9): 805-10
World Health Organization (WHO). Global leprosy update, 2016: accelerating reduction of disease burden. Wkly Epidemiol Rec. 2017; 92(35): 501-20
Sharma S, Sneath E, Cheng AC, Friedman ND. Community-acquired syndromes causing morbidity and mortality in Australia. Commun Dis Intell Q Rep. 2017; 41(1): E49-E57
World Health Organization (WHO). WHO Mortality Database Version October 2017. Geneva, Switzerland: World Health Organization (WHO)
Hong TP, Gow P, Fink M, Dev A, Roberts S, Nicoll A, Lubel J, Kronborg I, Arachchi N, Ryan M, Kemp W, Knight V, Farrugia H, Thursfield V, Desmond P, Thompson AJ, Bell S. Novel population-based study finding higher than reported hepatocellular carcinoma incidence suggests an updated approach is needed. Hepatology. 2016; 63(4): 1205-12

Laupland KB, Schønheyder HC, Kennedy KJ, Lyytikäinen O, Valiquette L, Galbraith J, Collignon P. Salmonella enterica bacteraemia: a multi-national population-based cohort study. BMC Infect Dis. 2010; 10(1): 95
Bray F, Colombet M, Mery L, Piñeros M, Znaor A, Zanetti R and Ferlay J, editors (2017). Cancer Incidence in Five Continents, Vol. XI (electronic version). Lyon: International Agency for Research on Cancer. Available from: http://ci5.iarc.fr
Pandeya N, Green AC, Whiteman DC. Prevalence and Determinants of Frequent Gastroesophageal Reflux Symptoms in the Australian Community. Dis Esophagus. 2012; 25(7): 573-83
Prakash R, Horsfall M, Markwick A, Pumar M, Lee L, Sinhal A, Joseph MX, Chew DP. Prognostic impact of moderate or severe mitral regurgitation (MR) irrespective of concomitant comorbidities: a retrospective matched cohort study. BMJ Open. 2014; 4(7): e004984
Chen Z, Thompson SK, Jamieson GG, Devitt PG, Watson DI. Effect of sex on symptoms associated with gastroesophageal reflux. Arch Surg. 2011; 146(10): 1164-9
Eslick GD, Talley NJ. Gastroesophageal reflux disease (GERD): risk factors, and impact on quality of life-a population-based study. J Clin Gastroenterol. 2009; 43(2): 111-7
Bolin TD, Korman MG, Hansky J, Stanton R. Heartburn: community perceptions. J Gastroenterol Hepatol. 2000; 15(1): 35-9
Bytzer P, Talley NJ, Leemon M, Young LJ, Jones MP, Horowitz M. Prevalence of gastrointestinal symptoms associated with diabetes mellitus: a population-based survey of 15,000 adults. Arch Intern Med. 2001; 1610(16): 1989-96
Boyce PM, Talley NJ, Burke C, Koloski NA. Epidemiology of the functional gastrointestinal disorders diagnosed according to Rome II criteria: an Australian population-based study. Intern Med J. 2006; 36(1): 28-36
Farrant BM, Stanley FJ, Hardelid P, Shepherd CC. Stillbirth and neonatal death rates across time: the influence of pregnancy terminations and birth defects in a Western Australian population-based cohort study. BMC Pregnancy Childbirth. 2016; 16: 112
Australia and New Zealand Dialysis and Transplant Registry (ANZDATA). Australia and New Zealand Dialysis and Transplant Registry 1991
Australia and New Zealand Dialysis and Transplant Registry (ANZDATA). Australia and New Zealand Dialysis and Transplant Registry 1992
Australia and New Zealand Dialysis and Transplant Registry (ANZDATA). Australia and New Zealand Dialysis and Transplant Registry 1993
Australia and New Zealand Dialysis and Transplant Registry (ANZDATA). Australia and New Zealand Dialysis and Transplant Registry 1994
Australia and New Zealand Dialysis and Transplant Registry (ANZDATA). Australia and New Zealand Dialysis and Transplant Registry 1996
Australia and New Zealand Dialysis and Transplant Registry (ANZDATA). Australia and New Zealand Dialysis and Transplant Registry 1997
Australia and New Zealand Dialysis and Transplant Registry (ANZDATA). Australia and New Zealand Dialysis and Transplant Registry 1998
Australia and New Zealand Dialysis and Transplant Registry (ANZDATA). Australia and New Zealand Dialysis and Transplant Registry 1999
Australia and New Zealand Dialysis and Transplant Registry (ANZDATA). Australia and New Zealand Dialysis and Transplant Registry 2000
Australia and New Zealand Dialysis and Transplant Registry (ANZDATA). Australia and New Zealand Dialysis and Transplant Registry 2001

Australia and New Zealand Dialysis and Transplant Registry (ANZDATA). Australia and New Zealand Dialysis and Transplant Registry 2002
Australia and New Zealand Dialysis and Transplant Registry (ANZDATA). Australia and New Zealand Dialysis and Transplant Registry 2003
Australia and New Zealand Dialysis and Transplant Registry (ANZDATA). Australia and New Zealand Dialysis and Transplant Registry 2004
Australia and New Zealand Dialysis and Transplant Registry (ANZDATA). Australia and New Zealand Dialysis and Transplant Registry 2005
Australia and New Zealand Dialysis and Transplant Registry (ANZDATA). Australia and New Zealand Dialysis and Transplant Registry 2006
Australia and New Zealand Dialysis and Transplant Registry (ANZDATA). Australia and New Zealand Dialysis and Transplant Registry 2007
Australia and New Zealand Dialysis and Transplant Registry (ANZDATA). Australia and New Zealand Dialysis and Transplant Registry 2008
Australia and New Zealand Dialysis and Transplant Registry (ANZDATA). Australia and New Zealand Dialysis and Transplant Registry 2009
Newbury J, Kleinig T, Leyden J, Arima H, Castle S, Cranefield J, Paterson T, Jannes J, Crotty M, Anderson C. Stroke Epidemiology in an Australian Rural Cohort (SEARCH). Int J Stroke. 2017; 12(2): 161-168
Vilayur E, Gopinath B, Harris DC, Burlutsky G, McMahon CM, Mitchell P. The association between reduced GFR and hearing loss: a cross-sectional population-based study. Am J Kidney Dis. 2010; 56(4): 661-9
Australian Institute of Health and Welfare, National Diabetes Register (NDR) (Australia). Australia Incidence of Insulin-Treated Diabetes 2015. Canberra, Australia: Australian Institute of Health and Welfare, 2017
Australian Institute of Health and Welfare, National Diabetes Register (NDR) (Australia). Australia Incidence of Insulin-Treated Diabetes 2014. Canberra, Australia: Australian Institute of Health and Welfare, 2016
Williams K, MacDermott S, Ridley G, Glasson EJ, Wray JA. The prevalence of autism in Australia. Can it be established from existing data?. J Paediatr Child Health. 2008; 44(9): 504–10
Nassar N, Dixon G, Bourke J, Bower C, Glasson E, de Klerk N, Leonard H. Autism spectrum disorders in young children: effect of changes in diagnostic practices. Int J Epidemiol. 2009; 38(5): 1245–54
Hay P, Girosi F, Mond J. Prevalence and sociodemographic correlates of DSM-5 eating disorders in the Australian population. J Eat Disord. 2015; 3(19): 1–7
Hay P, Mitchison D, Collado AEL, González-Chica DA, Stocks N, Touyz S. Burden and health-related quality of life of eating disorders, including Avoidant/Restrictive Food Intake Disorder (ARFID), in the Australian population. J Eat Disord. 2017; 5(21): 1–10
Hagan R, Benninger H, Chiffings D, Evans S, French N. Very preterm birth--a regional study. Part 2: The very preterm infant. Br J Obstet Gynaecol. 1996; 103(3): 239-45
Anderson C, Jamrozik K, Burvill P, Chakera T, Johnson G, Stewart-Wynne E. Determining the incidence of different subtypes of stroke: results from the Perth Community Stroke Study, 1989-1990. Med J Aust. 1993; 158(2): 85-9
Lai L, Morgan M. Incidence of subarachnoid haemorrhage: an Australian national hospital morbidity database analysis. J Clin Neurosci. 2012;

19(5): 733-9
World Health Organization Regional Office for the Western Pacific (WPRO-WHO). WPRO Dengue Situation Update Number 533, 2 January 2018. Manila, Philippines: World Health Organization Regional Office for the Western Pacific (WPRO-WHO), 2018
Ward G, Jamrozik K, Stewart-Wynne E. Incidence and outcome of cerebrovascular disease in Perth, Western Australia. Stroke. 1988; 19(12): 1501-6
Fairthorne J, de Klerk N, Leonard HM, Schieve LA, Yeargin-Allsopp M. Maternal Race-Ethnicity, Immigrant Status, Country of Birth, and the Odds of a Child With Autism. Child Neurol Open. 2017; 4: 1-9
Drew JH, Beischer NA, Walstab JE, Liang ST, Stronge JM. Contribution of prematurity and low birth-weight to neonatal death in Chinese and Western populations. Aust N Z J Obstet Gynaecol. 1988; 28(3): 190-2
Karvonen M, Viik-Kajander M, Moltchanova E, Libman I, LaPorte R, Tuomilehto J. Incidence of childhood type 1 diabetes worldwide. Diabetes Mondiale (DiaMond) Project Group. Diabetes Care. 2000; 23(10): 1516–26
Kirby Institute. HIV, viral hepatitis and sexually transmissible infections in Australia: annual surveillance report 2017. Sydney: Kirby Institute, UNSW Sydney; 2017
World Health Organization (WHO). WHO International Travel and Health, Annex 1, Countries With Risk of Yellow Fever Transmission and Countries Requiring Yellow Fever Vaccination 2017 Update. Geneva, Switzerland: World Health Organization (WHO), 2017
Knox SA, Harrison CM, Britt HC, Henderson JV. Estimating prevalence of common chronic morbidities in Australia. Med J Aust. 2008; 189(2): 66-70
Brennan DS, Spencer AJ, Roberts-Thomson KF. Work-Family Conflict Modifies the Association of Smoking and Periodontal Disease. Int J Behav Med. 2017; 24(1): 77-82
Wright F, Chu SY, Milledge KL, Valdez E, Law G, Hsu B, Naganathan V, Hirani V, Blyth FM, Le Couteur DG, Harford J, Waite LM, Handelsman DJ, Seibel MJ, Cumming RG. Oral health of community-dwelling older Australian men: the Concord Health and Ageing in Men Project (CHAMP). Aust Dent J. 2017
Australian Institute of Health and Welfare, University of Adelaide. Population Estimates, Standard Errors and Hypothesis Tests from the 1987-88 National Oral Health Survey of Australia. Adelaide, Australia: University of Adelaide, 1998
Gattellari M, Goumas C, Worthington J. Declining rates of fatal and nonfatal intracerebral hemorrhage: epidemiological trends in Australia. J Am Heart Assoc. 2014; 3(6): e001161
Department of Health (Australia). Overseas-acquired Vectorbourne Disease Notifications in Australia: Fortnight Surveillance Reports for 2018. Canberra, Australia: Department of Health (Australia), 2018
Haby M, Peat J, Marks G, Woolcock A, Leeder S. Asthma in preschool children: prevalence and risk factors. Thorax. 2001; 56(8): 589–95
Downs SH, Marks GB, Sporik R, Belosouva EG, Car NG, Peat JK. Continued increase in the prevalence of asthma and atopy. Arch Dis Child.

2001; 84(1): 20–3
Pearce N, Weiland S, Keil U, Langridge P, Anderson HR, Strachan D, Bauman A, Young L, Gluyas P, Ruffin D, Et A. Self-reported prevalence of asthma symptoms in children in Australia, England, Germany and New Zealand: an international comparison using the ISAAC protocol. Eur Respir J. 1993; 6(10): 1455–61
Australasian Association of Cancer Registries, Australian Institute of Health and Welfare. Australia Cancer Incidence and Mortality Books 2017. Canberra, Australia: Australian Institute of Health and Welfare, 2017
Johnson NW, Lalloo R, Kroon J, Fernando S, Tut O. Effectiveness of water fluoridation in caries reduction in a remote Indigenous community in Far North Queensland. Aust Dent J. 2014; 59(3): 366-71
Koh R, Pukallus ML, Newman B, Foley M, Walsh LJ, Seow WK. Effects of water fluoridation on caries experience in the primary dentition in a high caries risk community in Queensland, Australia. Caries Res. 2015; 49(2): 184-91
International Agency for Research on Cancer (IARC), International Association of Cancer Registries (IACR), World Health Organization (WHO). International Incidence of Childhood Cancer Volume III. Lyon, France: International Agency for Research on Cancer (IARC), 2017
World Health Organization (WHO). Global leprosy update, 2017: reducing the disease burden due to leprosy. Wkly Epidemiol Rec. 2018; 93(35): 445-56
World Health Organization (WHO). WHO Mortality Database Version December 2018. Geneva, Switzerland: World Health Organization (WHO)
World Health Organization Regional Office for the Western Pacific (WPRO-WHO). WPRO Dengue Situation Updates 2019. Manila, Philippines: World Health Organization Regional Office for the Western Pacific (WPRO-WHO), 2019
Fagan RL, Hanna JN, Messer RD, Brookes DL, Murphy DM. The epidemiology of invasive pneumococcal disease in children in Far North Queensland. J Paediatr Child Health. 2001; 37(6): 571-5
McIntyre PB, Macintyre CR, Gilmour R, Wang H. A population based study of the impact of corticosteroid therapy and delayed diagnosis on the outcome of childhood pneumococcal meningitis. Arch Dis Child. 2005; 90(4): 391-6
Szoeke CEI, Cicuttini FM, Guthrie JR, Clark MS, Dennerstein L. Factors affecting the prevalence of osteoarthritis in healthy middle-aged women: data from the longitudinal Melbourne Women's Midlife Health Project. Bone. 2006; 39(5): 1149-1155
Vegh Z, Burisch J, Pedersen N, Kaimakliotis I, Duricova D, Bortlik M, Avnstrøm S, Vinding KK, Olsen J, Nielsen KR, Katsanos KH, Tsianos EV, Lakatos L, Schwartz D, Odes S, Lupinacci G, De Padova A, Jonaitis L, Kupcinskas L, Turcan S, Tighineanu O, Mihu I, Barros LF, Magro F, Lazar D, Goldis A, Fernandez A, Hernandez V, Niewiadomski O, Bell S, Langholz E, Munkholm P, Lakatos PL, EpiCom-group. Incidence and initial disease course of inflammatory bowel diseases in 2011 in Europe and Australia: results of the 2011 ECCO-EpiCom inception cohort. J Crohns Colitis. 2014; 8(11): 1506-15
Kodama R, Muraki S, Oka H, Iidaka T, Teraguchi M, Kagotani R, Asai Y, Yoshida M, Morizaki Y, Tanaka S, Kawaguchi H, Nakamura K, Akune T, Yoshimura N. Prevalence of hand osteoarthritis and its relationship to hand pain and grip strength in Japan: The third survey of the ROAD

study. Mod Rheumatol. 2016; 26(5): 767-73
World Health Organization (WHO). WHO Mortality Database Version May 2019. Geneva, Switzerland: World Health Organization (WHO)
Almeida OP, Marsh K, Flicker L, Hickey M, Sim M, Ford A. Depressive symptoms in midlife: the role of reproductive stage. Menopause. 2016; 23(6): 669-75
Zeleke BM, Bell RJ, Billah B, Davis SR. Vasomotor symptoms are associated with depressive symptoms in community-dwelling older women. Menopause. 2017; 24(12): 1365-1371
Connors MH, Ames D, Boundy K, Clarnette R, Kurrle S, Mander A, Ward J, Woodward M, Brodaty H. Predictors of Mortality in Dementia: The PRIME Study. J Alzheimers Dis. 2016; 52(3): 967-74
LoGiudice D, Hassett A, Cook R, Flicker L, Ames D. Equity of access to a memory clinic in Melbourne? Non-English speaking background attenders are more severely demented and have increased rates of psychiatric disorders. Int J Geriatr Psychiatry. 2001; 16(3): 327-34
Broe GA, Henderson AS, Creasey H, McCusker E, Korten AE, Jorm AF, Longley W, Anthony JC. A case-control study of Alzheimers disease in Australia. Neurology. 1990; 40(11): 1698707
Smith K, Flicker L, Dwyer A, Atkinson D, Almeida OP, Lautenschlager NT, LoGiudice D. Factors associated with dementia in Aboriginal Australians. Aust N Z J Psychiatry. 2010; 44(10): 88893
Simons LA, Simons J, McCallum J, Friedlander Y. Lifestyle factors and risk of dementia: Dubbo Study of the elderly. Med J Aust. 2006; 184(2): 6870
Srikanth VK, Anderson JFI, Donnan GA, Saling MM, Didus E, Alpitsis R, Dewey HM, Macdonell R a L, Thrift AG. Progressive dementia after first-ever stroke: a community-based follow-up study. Neurology. 2004; 63(5): 78592
Sawyer MG, Reece CE, Sawyer ACP, Johnson SE, Lawrence D. Has the Prevalence of Child and Adolescent Mental Disorders in Australia Changed Between 1998 and 2013to 2014?. J Am Acad Child Adolesc Psychiatry. 2018; 57(5): 343-350e5
Lawrence BJ, Gasson N, Loftus AM. Prevalence and Subtypes of Mild Cognitive Impairment in Parkinsons Disease. Sci Rep. 2016; 6: 33929
Loftus AM, Bucks RS, Thomas M, Kane R, Timms C, Barker RA, Gasson N. Retrospective assessment of movement disorder society criteria for mild cognitive impairment in Parkinsons disease. J Int Neuropsychol Soc. 2015; 21(2): 13745
Goller JL, De Livera AM, Guy RJ, Low N, Donovan B, Law M, Kaldor JM, Fairley CK, Hocking JS. Rates of pelvic inflammatory disease and ectopic pregnancy in Australia, 2009-2014: ecological analysis of hospital data. Sex Transm Infect. 2018; 94(7): 534-541
Baldwin HJ, Patterson JA, Nippita TA, Torvaldsen S, Ibiebele I, Simpson JM, Ford JB. Maternal and neonatal outcomes following abnormally invasive placenta: a population-based record linkage study. Acta Obstet Gynecol Scand. 2017; 96(11): 1373-1381
Jayaratnam S, Kua S, deCosta C, Franklin R. Maternal 'near miss' collection at an Australian tertiary maternity hospital. BMC Pregnancy Childbirth. 2018; 18(1): 221
McIver CJ, Hansman G, White P, Doultree JC, Catton M, Rawlinson WD. Diagnosis of enteric pathogens in children with gastroenteritis.

Pathology. 2001; 33(3): 353-8
McIntyre P, Jepson R, Leeder S, Irwig L. The outcome of childhood Haemophilus influenzae meningitis. A population based study. Med J Aust. 1993; 159(11-12): 766-72
Turnour CE, Cretikos MA, Conaty SJ. Prevalence of chronic hepatitis B in South Western Sydney: evaluation of the country of birth method using maternal seroprevalence data. Aust N Z J Public Health. 2011; 35(1): 226
World Health Organization (WHO). WHO Distribution of Measles Cases by Country and by Month 2011-2020
Bowden FJ, OKeefe EJ, Primrose R, Currie MJ. Sexually transmitted infections, blood-borne viruses and risk behaviour in an Australian senior high school population-the SHLiRP study. Sex Health. 2005; 2(4): 22936
Burgess MA, McIntosh ED, Allars HM, Kenrick KG. Hepatitis B in urban Australian schoolchildren. No evidence of horizontal transmission between high-risk and low-risk groups. Med J Aust. 1993; 159(5): 3159
The Kirby Institute. Monitoring hepatitis C treatment uptake in Australia (Issue 7). The Kirby Institute, UNSW Sydney, Sydney, NSW, Australia, July 2017
Amin J, Gidding H, Gilbert G, Backhouse J, Kaldor J, Dore G, Burgess M. Hepatitis C prevalence-a nationwide serosurvey. Commun Dis Intell Q Rep. 2004; 28(4): 51721
Sobey CG, Judkins CP, Sundararajan V, Phan TG, Drummond GR, Srikanth VK. Risk of Major Cardiovascular Events in People with Down Syndrome. PLoS One. 2015; 10(9): e0137093

Supplementary Table 7 GBD data input sources for all risk factors

Citation
Rodrigo S, Sinclair M, Forbes A, Cunliffe D, Leder K. Drinking Rainwater: A Double-Blinded, Randomized Controlled Study of Water Treatment Filters and Gastroenteritis Incidence. Am J Public Health. 2011; 101(5): 842–7
McElduff P, Dobson AJ, Jackson R, Beaglehole R, Heller RF, Lay-Yee R. Coronary events and exposure to environmental tobacco smoke: a case-control study from Australia and New Zealand. Tob Control. 1998; 7(1): 41–6
You RX, Thrift AG, McNeil JJ, Davis SM, Donnan GA. Ischemic stroke risk and passive exposure to spouses’ cigarette smoking. Melbourne Stroke Risk Factor Study (MERFS) Group. Am J Public Health. 1999; 89(4): 572–5
Pereira G, Bell ML, Belanger K, de Klerk N. Fine particulate matter and risk of preterm birth and pre-labor rupture of membranes in Perth, Western Australia 1997-2007: a longitudinal study. Environ Int. 2014; 73: 143-9
Australia - South Australia Air Monitoring Network 2014 as it appears in Shaddick G, Thomas ML. Particulate Matter 2.5 and 10 Surface Monitor Station Expanded Database 2008-2017. [Unpublished]
Australia - Western Australia Air Quality Monitoring Network PM2.5 Data 2014 as it appears in Shaddick G, Thomas ML. Particulate Matter 2.5 and 10 Surface Monitor Station Expanded Database 2008-2017. [Unpublished]
Australia - New South Wales Air Quality PM2.5 and PM 10 Particulate Data 2014 as it appears in Shaddick G, Thomas ML. Particulate Matter 2.5 and 10 Surface Monitor Station Expanded Database 2008-2017. [Unpublished]
Australia - Northern Territory Ambient Air Quality Monitoring Report 2014 as it appears in Shaddick G, Thomas ML. Particulate Matter 2.5 and 10 Surface Monitor Station Expanded Database 2008-2017. [Unpublished]
Australia - Western Australia Air Monitoring Report 2016 as it appears in Shaddick G, Thomas ML. Particulate Matter 2.5 and 10 Surface Monitor Station Expanded Database 2008-2017. [Unpublished]
Australia - Queensland Air Monitoring 2015 as it appears in Shaddick G, Thomas ML. Particulate Matter 2.5 and 10 Surface Monitor Station Expanded Database 2008-2017. [Unpublished]
Australia - Queensland Air Monitoring 2016 as it appears in Shaddick G, Thomas ML. Particulate Matter 2.5 and 10 Surface Monitor Station Expanded Database 2008-2017. [Unpublished]
Australia - Northern Territory Air Quality Monitoring Network All Available Air Quality Index Values 2012 as it appears in Shaddick G, Thomas ML. Particulate Matter 2.5 and 10 Surface Monitor Station Expanded Database 2008-2017. [Unpublished]
Australia - Northern Territory Air Quality Monitoring Network All Available Air Quality Index Values 2013 as it appears in Shaddick G, Thomas ML. Particulate Matter 2.5 and 10 Surface Monitor Station Expanded Database 2008-2017. [Unpublished]
Australia - Northern Territory Air Quality Monitoring Network All Available Air Quality Index Values 2014 as it appears in Shaddick G, Thomas ML.

Particulate Matter 2.5 and 10 Surface Monitor Station Expanded Database 2008-2017. [Unpublished]
Australia - Northern Territory Air Quality Monitoring Network All Available Air Quality Index Values 2015 as it appears in Shaddick G, Thomas ML. Particulate Matter 2.5 and 10 Surface Monitor Station Expanded Database 2008-2017. [Unpublished]
Australia - Northern Territory Air Quality Monitoring Network All Available Air Quality Index Values 2016 as it appears in Shaddick G, Thomas ML. Particulate Matter 2.5 and 10 Surface Monitor Station Expanded Database 2008-2017. [Unpublished]
Australia - New South Wales Air Quality PM2.5 and PM 10 Particulate Data 2013 as it appears in Shaddick G, Thomas ML. Particulate Matter 2.5 and 10 Surface Monitor Station Expanded Database 2008-2017. [Unpublished]
Australia - New South Wales Air Quality PM2.5 and PM 10 Particulate Data 2015 as it appears in Shaddick G, Thomas ML. Particulate Matter 2.5 and 10 Surface Monitor Station Expanded Database 2008-2017. [Unpublished]
Australia - New South Wales Air Quality PM2.5 and PM 10 Particulate Data 2016 as it appears in Shaddick G, Thomas ML. Particulate Matter 2.5 and 10 Surface Monitor Station Expanded Database 2008-2017. [Unpublished]
Australia - South Australia Air Monitoring Network 2012 as it appears in Shaddick G, Thomas ML. Particulate Matter 2.5 and 10 Surface Monitor Station Expanded Database 2008-2017. [Unpublished]
Australia - South Australia Air Monitoring Network 2013 as it appears in Shaddick G, Thomas ML. Particulate Matter 2.5 and 10 Surface Monitor Station Expanded Database 2008-2017. [Unpublished]
Australia - South Australia Air Monitoring Network 2015 as it appears in Shaddick G, Thomas ML. Particulate Matter 2.5 and 10 Surface Monitor Station Expanded Database 2008-2017. [Unpublished]
Australia - South Australia Air Monitoring Network 2016 as it appears in Shaddick G, Thomas ML. Particulate Matter 2.5 and 10 Surface Monitor Station Expanded Database 2008-2017. [Unpublished]
Langroo MK, Wise KN, Duggleby JC, Kotler LH. A nationwide survey of 222Rn and gamma radiation levels in Australian homes. Health Phys. 1991; 61(6): 753-61
Rangan AM, Aitkin I, Blight GD, Binns CW. Factors affecting iron status in 15-30 year old female students. Asia Pac J Clin Nutr. 1997; 6(4): 291-5
World Health Organization (WHO). WHO Global Database on Vitamin A Deficiency. Geneva, Switzerland: World Health Organization (WHO)
FAO Supply Utilization Accounts 1961-2013. Personal Correspondence with Dr. Josef Schmidhuber, 2016. [Unpublished]
Rangan AM, Samman S. Zinc intake and its dietary sources: results of the 2007 Australian National Children’s Nutrition and Physical Activity Survey. Nutrients. 2012; 4(7): 611–24
Burrows TL, Collins K, Watson J, Guest M, Boggess MM, Neve M, Rollo M, Duncanson K, Collins CE. Validity of the Australian Recommended Food Score as a diet quality index for Pre-schoolers. Nutr J. 2014; 13: 87
U.S. Department of Agriculture (USDA). USDA Global Tobacco Database 1960-2005. Washington D.C. , United States: U.S. Department of Agriculture (USDA)

Food and Agriculture Organization of the United Nations (FAO). FAOSTAT Commodity Balances - Crops Primary Equivalent. Rome, Italy: Food and Agriculture Organization of the United Nations (FAO)
Euromonitor International. Euromonitor Passport - Smoking Tobacco Statistics . London, United Kingdom: Euromonitor International
International Diabetes Institute (IDI). Australia Diabetes, Obesity and Lifestyle Study 1999-2000. Melbourne, Australia: International Diabetes Institute (IDI)
Australian Bureau of Statistics. Australia National Health Survey 1995. Canberra, Australia: Australian Bureau of Statistics
P.N. Lee Statistics and Computing Ltd. International Mortality and Smoking Statistics Version 4.04. Sutton, United Kingdom: P.N. Lee Statistics and Computing Ltd, 2009
Australian Bureau of Statistics. Australia National Health Survey 2001. Canberra, Australia: Australian Bureau of Statistics
Australian Bureau of Statistics. Australia National Health Survey 2004-2005
Australian Bureau of Statistics. Australia National Health Survey 2007-2008. Canberra, Australia: Australian Bureau of Statistics
Gliksman MD, Dwyer T, Wlodarczyk J, Pierce JP. Cigarette smoking in Australian schoolchildren. Med J Aust. 1989; 150(2): 81–4
Australian Institute of Health and Welfare, Department of Health and Ageing (Australia), Roy Morgan Research, Social Research Centre (Australia). Australia National Drug Strategy Household Survey 2007
Australian Institute of Health and Welfare. Australia National Drug Strategy Household Survey 2004
Australian Institute of Health and Welfare. Australia National Drug Strategy Household Survey 2001
Australian Institute of Health and Welfare, Roy Morgan Research. Australia National Drug Strategy Household Survey 1998
Australian Institute of Health and Welfare, National Heart Foundation (Australia). Australia Risk Factor Prevalence Survey 1989. Acton, Australia: Australian Data Archive
Australian Bureau of Statistics. Australia National Health Survey 2011-2013
Gartner CE, Barendregt JJ, Hall WD. Predicting the future prevalence of cigarette smoking in Australia: how low can we go and by when?. Tob Control. 2009; 18(3): 183-9
White V, Hill D, Siahpush M, Bobevski I. How has the prevalence of cigarette smoking changed among Australian adults? Trends in smoking prevalence between 1980 and 1932. Tob Control. 2003; 12(Suppl 2): ii67-ii74
Australian Institute of Health and Welfare, Roy Morgan Research. Australia National Drug Strategy Household Survey 2010
Australian Institute of Health and Welfare, National Heart Foundation (Australia). Australia Risk Factor Prevalence Survey 1994
ISSP Research Group (2009): International Social Survey Programme: Health and Health Care - ISSP 2011. GESIS Data Archive, Cologne. ZA5800 Data file version 3.0.0, doi:10.4232/1.12252
Australian Bureau of Statistics. Australia National Health Survey 1989-1990

Australian Institute of Health and Welfare, Roy Morgan Research. Australia National Drug Strategy Household Survey 2013
Australian Bureau of Statistics, Australian Health Survey Reference Group, Roy Morgan Research, University of Western Australia. Australia Child and Adolescent Survey of Mental Health and Wellbeing 2013-2014
Australian Bureau of Statistics. Australia National Health Survey 2014-2015. Canberra, Australia: Australian Bureau of Statistics
Australia - Western Australia Health in Men Study as it appears in Jamrozik K, McLaughlin D, McCaul K, Almeida OP, Wong KY, Vagenas D, Dobson A. Women who smoke like men die like men who smoke: findings from two Australian cohort studies. Tob Control. 2011; 20(4): 258-65
Australia Longitudinal Study on Women's Health 1996-2016 as it appears in Jamrozik K, McLaughlin D, McCaul K, Almeida OP, Wong KY, Vagenas D, Dobson A. Women who smoke like men die like men who smoke: findings from two Australian cohort studies. Tob Control. 2011; 20(4): 258-65
Gallup. Australia World Poll 2007
World Health Organization (WHO). WHO Report on the Global Tobacco Epidemic 2019. Geneva, Switzerland: World Health Organization (WHO), 2019
Blizzard L, Ponsonby A-L, Dwyer T, Venn A, Cochrane JA. Parental smoking and infant respiratory infection: how important is not smoking in the same room with the baby?. Am J Public Health. 2003; 482-8
Gun RT, Pratt N, Ryan P, Gordon I, Roder D. Tobacco and alcohol-related mortality in men: estimates from the Australian cohort of petroleum industry workers. Aust N Z J Public Health. 2006; 30(4): 318-24
Jamrozik K, McLaughlin D, McCaul K, Almeida OP, Wong KY, Vagenas D, Dobson A. Women who smoke like men die like men who smoke: findings from two Australian cohort studies. Tob Control. 2011; 20(4): 258-65
Aarhus University, Addiction Switzerland Research Institute, Alcohol Research Group, Public Health Institute, Centre for Addiction and Mental Health (Canada), Centre for Alcohol Policy Research, Turning Point Alcohol and Drug Centre (Australia), Kettil Bruun Society for Social and Epidemiological Research on Alcohol, University of North Dakota. Australia Gender, Alcohol and Culture: An International Study (GENACIS) 1994
Aarhus University, Addiction Switzerland Research Institute, Alcohol Research Group, Public Health Institute, Centre for Addiction and Mental Health (Canada), Centre for Alcohol Policy Research, Turning Point Alcohol and Drug Centre (Australia), Kettil Bruun Society for Social and Epidemiological Research on Alcohol, University of North Dakota. Australia - Victoria Gender, Alcohol and Culture: An International Study (GENACIS) 2007
World Health Organization Regional Office for Europe (EURO-WHO). Health Behaviour in School-aged Children: WHO Collaborative Cross-National survey/study (HBSC) 2010
World Health Organization (WHO). WHO Global Health Observatory - Recorded Alcohol Per Capita Consumption 1960-1979. Geneva, Switzerland: World Health Organization (WHO)
World Health Organization (WHO). WHO Global Health Observatory - Recorded Alcohol Per Capita Consumption 1980-1999. Geneva, Switzerland: World Health Organization (WHO)
World Health Organization (WHO). WHO Global Health Observatory - Recorded Alcohol Per Capita Consumption 2000-2009 by country. Geneva, Switzerland: World Health Organization (WHO)

Euromonitor International. Euromonitor Passport - Alcoholic Drinks Statistics . London, United Kingdom: Euromonitor International
Bartu A, Freeman NC, Gawthorne GS, Codde JP, Holman CDJ. Mortality in a cohort of opiate and amphetamine users in Perth, Western Australia. Addiction. 2004; 99(1): 53-60
Digiusto E, Shakeshaft A, Ritter A, O'Brien S, Mattick RP, NEPOD Research Group. Serious adverse events in the Australian National Evaluation of Pharmacotherapies for Opioid Dependence (NEPOD). Addiction. 2004; 99(4): 450-60
Tait RJ, Ngo HTT, Hulse GK. Mortality in heroin users 3 years after naltrexone implant or methadone maintenance treatment. J Subst Abuse Treat. 2008; 35(2): 116-24
Razali K, Thein HH, Bell J, Cooper-Stanbury M, Dolan K, Dore G, George J, Kaldor J, Karvelas M, Li J, Maher L, McGregor S, Hellard M, Poeder F, Quaine J, Stewart K, Tyrrell H, Weltman M, Westcott O, Wodak A, Law M. Modelling the hepatitis C virus epidemic in Australia. Drug Alcohol Depend. 2007; 91(2-3): 228-35
Maher L, Jalaludin B, Chant KG, Jayasuriya R, Sladden T, Kaldor JM, Sargent PL. Incidence and risk factors for hepatitis c seroconversion in injecting drug users in Australia . Addiction. 2006; 101(10): 1499-508
Larney S, Hickman M, Guy R, Grebely J, Dore GJ, Gray RT, Day CA, Kimber J, Dagenhardt L. Estimating the Number of People who Inject Drugs in Australia. BMC Public Health. 2017; 17: 757
Degenhardt L, Randall D, Hall W, Law M, Butler T, Burns L. Mortality among clients of a state-wide opioid pharmacotherapy program over 20 years: risk factors and lives saved. Drug Alcohol Depend. 2009; 105(1): 9–15
Winter RJ, Dietze PM, Gouillou M, Hellard ME, Robinson P, Aitken CK. Hepatitis B virus exposure and vaccination in a cohort of people who inject drugs: what has been the impact of targeted free vaccination?. J Gastroenterol Hepatol. 2013; 28(2): 314-22
Busselton Population Medical Research Institute (Australia). Australia - Busselton Health Study 1981
Simmons D, McKenzie A, Eaton S, Shaw J, Zimmet P. Prevalence of diabetes in rural Victoria. Diabetes Res Clin Pract. 2005; 70(3): 287-90
Quan HL, Blizzard CL, Venn AJ, Thuy AB, Luc PH, Sharman JE. Blood pressure and body mass index: a comparison of the associations in the Caucasian and Asian populations. Hypertens Res. 2012; 35(5): 523-30
Baker IDI Heart and Diabetes Institute, International Diabetes Institute (IDI), Monash University (Australia), University of Melbourne. Australia Diabetes, Obesity and Lifestyle Study 2011-2012
Williams ED, Tapp RJ, Magliano DJ, Shaw JE, Zimmet PZ, Oldenburg BF. Health behaviours, socioeconomic status and diabetes incidence: the Australian Diabetes Obesity and Lifestyle Study (AusDiab). Diabetologia. 2010; 53(12): 2538-45
Smith KJ, Blizzard L, McNaughton SA, Gall SL, Dwyer T, Venn AJ. Daily eating frequency and cardiometabolic risk factors in young Australian adults: cross-sectional analyses. Br J Nutr. 2012; 108(6): 1086–94
Khambalia A, Phongsavan P, Smith BJ, Keke K, Dan L, Fitzhardinge A, Bauman AE. Prevalence and risk factors of diabetes and impaired fasting glucose in Nauru. BMC Public Health. 2011; 11: 719

Chittleborough CR, Baldock KL, Phillips PJ, Taylor AW, North West Adelaide Health Study Team. Achievement of management targets associated with incident and long-term diagnosed diabetes among a representative population sample. Diabetes Res Clin Pract. 2010; 88(3): 322–7
World Health Organization (WHO), WHO MONICA Project Investigators 2000. MONICA Population Survey Data Book 1979-1997. Geneva, Switzerland: World Health Organization (WHO), 2000
Andrews, Gary R., and George C. Myers. Australian [Adelaide] Longitudinal Study of Aging, Waves 1-5 [1992-1997]. ICPSR06707-v3. Ann Arbor, MI: Inter-university Consortium for Political and Social Research [distributor], 2000. doi:10.3886/ICPSR06707.v3
Janus ED, Tideman PA, Dunbar JA, Kilkkinen A, Bunker SJ, Philpot B, Tirimacco R, Mc Namara K, Heistaro S, Laatikainen T. Dyslipidaemia in rural Australia: prevalence, awareness, and adherence to treatment guidelines in the Greater Green Triangle Risk Factor Study. Med J Aust. 2010; 192(3): 127-32
Huggins CE, O'Reilly S, Brinkman M, Hodge A, Giles GG, English DR, Nowson CA. Relationship of urinary sodium and sodium-to-potassium ratio to blood pressure in older adults in Australia. Med J Aust. 2011; 195(3): 128-32
Gall SL, Jamrozik K, Blizzard L, Dwyer T, Venn A. Healthy lifestyles and cardiovascular risk profiles in young Australian adults: the Childhood Determinants of Adult Health Study. Eur J Cardiovasc Prev Rehabil. 2009; 16(6): 684–9
Department of Health and Ageing (Australia), World Health Organization (WHO). Australia World Health Survey 2003. Geneva, Switzerland: World Health Organization (WHO), 2005
Australian Bureau of Statistics, Australian Institute of Family Studies, Department of Families, Housing, Community Services and Indigenous Affairs (Australia). Australia Longitudinal Study of Children, Wave 4 2009-2010. Melbourne, Australia: Australian Institute of Family Studies
International Diabetes Institute (IDI). Australia Diabetes, Obesity and Lifestyle Study 2004-2005
Ackerman IN, Osborne RH. Obesity and increased burden of hip and knee joint disease in Australia: results from a national survey. BMC Musculoskelet Disord. 2012; 13(1): 254
Janus ED, Laatikainen T, Dunbar JA, Kilkkinen A, Bunker SJ, Philpot B, Tideman PA, Tirimacco R, Heistaro S. Overweight, obesity and metabolic syndrome in rural southeastern Australia. Med J Aust. 2007; 187(3): 147-52
Australian Bureau of Statistics, Department of Health and Family Services (Australia). Australia National Nutrition Survey 1995-1996. Canberra, Australia: Australian Bureau of Statistics
Markwick A, Vaughan L, Ansari Z. Opposing socioeconomic gradients in overweight and obese adults. Aust N Z J Public Health. 2013; 37(1): 32-8
Davies CA, Vandelanotte C, Duncan MJ, van Uffelen JGZ. Associations of physical activity and screen-time on health related quality of life in adults. Prev Med. 2012; 55(1): 46-9
Commonwealth Scientific and Industrial Research Organisation (CSIRO) National Research Preventative Health Flagship (Australia), Department of Health and Ageing (Australia), I-view Pty Ltd, University of South Australia. Australia National Children's Nutrition and Physical Activity Survey 2007
Magarey AM, Daniels LA, Boulton TJ. Prevalence of overweight and obesity in Australian children and adolescents: reassessment of 1985 and 1995

data against new standard international definitions. Med J Aust. 2001; 174(11): 561-4
Dhaliwal SS, Howat P, Bejoy T, Welborn TA. Self-reported weight and height for evaluating obesity control programs. Am J Health Behav. 2010; 34(4): 489-99
Patton GC, Coffey C, Carlin JB, Sawyer SM, Williams J, Olsson CA, Wake M. Overweight and obesity between adolescence and young adulthood: a 10-year prospective cohort study. J Adolesc Health. 2011; 48(3): 275-80
Vaska V, Volkmer R. Increasing prevalence of obesity in South Australian 4-year-olds: 1995-2002. J Paediatr Child Health. 2004; 40(7): 353-5
Flicker L, McCaul KA, Hankey GJ, Jamrozik K, Brown WJ, Byles JE, Almeida OP. Body mass index and survival in men and women aged 70 to 75. J Am Geriatr Soc. 2010; 58(2): 234-41
Booth ML, Wake M, Armstrong T, Chey T, Hesketh K, Mathur S. The epidemiology of overweight and obesity among Australian children and adolescents, 1995-97. Aust N Z J Public Health. 2001; 25(2): 162-9
Hesketh K, Wake M, Waters E, Carlin J, Crawford D. Stability of body mass index in Australian children: a prospective cohort study across the middle childhood years. Public Health Nutr. 2004; 7(2): 303-9
Siu J, Giskes K, Shaw J, Turrell G. Perceived weight status may contribute to education inequalities in five-year weight change among mid-aged women. Aust N Z J Public Health. 2011; 35(3): 284-91
Renzaho A, Wooden M, Houng B. Associations between body mass index and health-related quality of life among Australian adults. Qual Life Res. 2010; 19(4): 515-20
Sanigorski AM, Bell AC, Kremer PJ, Swinburn BA. High childhood obesity in an Australian population. Obesity (Silver Spring). 2007; 15(8): 1908-12
Booth ML, Dobbins T, Okely AD, Denney-Wilson E, Hardy LL. Trends in the prevalence of overweight and obesity among young Australians, 1985, 1997, and 2004. Obesity (Silver Spring). 2007; 15(5): 1089-95
Shi Z, Taylor AW, Gill TK, Tuckerman J, Adams R, Martin J. Short sleep duration and obesity among Australian children. BMC Public Health. 2010; 609
Booth ML, Okely AD, Denney-Wilson E. Validation and application of a novel method of measuring non-response bias in school-based surveys of paediatric overweight and obesity. Int J Pediatr Obes. 2011; 6(2-2): e87-93
Kortt MA, Dollery B. Association between body mass index and health-related quality of life among an Australian sample. Clin Ther. 2011; 33(10): 1466-74
Holden CA, McLachlan RI, Pitts M, Cumming R, Wittert G, Ehsani JP, de Kretser DM, Handelsman DJ. Determinants of male reproductive health disorders: the Men in Australia Telephone Survey (MATeS). BMC Public Health. 2010; 96
Goldney RD, Dunn KI, Air TM, Dal Grande E, Taylor AW. Relationships between body mass index, mental health, and suicidal ideation: population perspective using two methods. Aust N Z J Psychiatry. 2009; 43(7): 652-8
O'Dea JA, Amy NK. Perceived and desired weight, weight related eating and exercising behaviours, and advice received from parents among thin, overweight, obese or normal weight Australian children and adolescents. Int J Behav Nutr Phys Act. 2011; 68

Khambalia A, Hardy LL, Bauman A. Accuracy of weight perception, life-style behaviours and psychological distress among overweight and obese adolescents. J Paediatr Child Health. 2012; 48(3): 220-7
Australia - Busselton Health Study as it appears in Singh GM, Danaei G, Farzadfar F, Stevens GA, Woodward M, Wormser D, Kaptoge S, Whitlock G, Qiao Q, Lewington S, Di Angelantonio E, Vander Hoorn S, Lawes CM, Ali MK, Mozaffarian D, Ezzati M; Global Burden of Metabolic Risk Factors of Chronic Diseases Collaborating Group; Asia-Pacific Cohort Studies Collaboration (APCSC); Diabetes Epidemiology: Collaborative analysis of Diagnostic criteria in Europe (DECODE); Emerging Risk Factor Collaboration (ERFC); Prospective Studies Collaboration (PSC). The age-specific quantitative effects of metabolic risk factors on cardiovascular diseases and diabetes: a pooled analysis. PLoS One. 2013; 8(7): e65174
Australia - Western Australia Abdominal Aortic Aneurysm Screening Program as it appears in Singh GM, Danaei G, Farzadfar F, Stevens GA, Woodward M, Wormser D, Kaptoge S, Whitlock G, Qiao Q, Lewington S, Di Angelantonio E, Vander Hoorn S, Lawes CM, Ali MK, Mozaffarian D, Ezzati M; Global Burden of Metabolic Risk Factors of Chronic Diseases Collaborating Group; Asia-Pacific Cohort Studies Collaboration (APCSC); Diabetes Epidemiology: Collaborative analysis of Diagnostic criteria in Europe (DECODE); Emerging Risk Factor Collaboration (ERFC); Prospective Studies Collaboration (PSC). The age-specific quantitative effects of metabolic risk factors on cardiovascular diseases and diabetes: a pooled analysis. PLoS One. 2013; 8(7): e65174
Australia - Adelaide Longitudinal Study of Aging as it appears in Singh GM, Danaei G, Farzadfar F, Stevens GA, Woodward M, Wormser D, Kaptoge S, Whitlock G, Qiao Q, Lewington S, Di Angelantonio E, Vander Hoorn S, Lawes CM, Ali MK, Mozaffarian D, Ezzati M; Global Burden of Metabolic Risk Factors of Chronic Diseases Collaborating Group; Asia-Pacific Cohort Studies Collaboration (APCSC); Diabetes Epidemiology: Collaborative analysis of Diagnostic criteria in Europe (DECODE); Emerging Risk Factor Collaboration (ERFC); Prospective Studies Collaboration (PSC). The age-specific quantitative effects of metabolic risk factors on cardiovascular diseases and diabetes: a pooled analysis. PLoS One. 2013; 8(7): e65174
Australia - Canberra Longitudinal Survey 1990-2002 as it appears in Singh GM, Danaei G, Farzadfar F, Stevens GA, Woodward M, Wormser D, Kaptoge S, Whitlock G, Qiao Q, Lewington S, Di Angelantonio E, Vander Hoorn S, Lawes CM, Ali MK, Mozaffarian D, Ezzati M; Global Burden of Metabolic Risk Factors of Chronic Diseases Collaborating Group; Asia-Pacific Cohort Studies Collaboration (APCSC); Diabetes Epidemiology: Collaborative analysis of Diagnostic criteria in Europe (DECODE); Emerging Risk Factor Collaboration (ERFC); Prospective Studies Collaboration (PSC). The age-specific quantitative effects of metabolic risk factors on cardiovascular diseases and diabetes: a pooled analysis. PLoS One. 2013; 8(7): e65174
Australia - Melbourne Collaborative Cohort Study as it appears in Singh GM, Danaei G, Farzadfar F, Stevens GA, Woodward M, Wormser D, Kaptoge S, Whitlock G, Qiao Q, Lewington S, Di Angelantonio E, Vander Hoorn S, Lawes CM, Ali MK, Mozaffarian D, Ezzati M; Global Burden of Metabolic Risk Factors of Chronic Diseases Collaborating Group; Asia-Pacific Cohort Studies Collaboration (APCSC); Diabetes Epidemiology: Collaborative analysis of Diagnostic criteria in Europe (DECODE); Emerging Risk Factor Collaboration (ERFC); Prospective Studies Collaboration (PSC). The age-specific quantitative effects of metabolic risk factors on cardiovascular diseases and diabetes: a pooled analysis. PLoS One. 2013; 8(7): e65174
Australia - Perth Multinational MONItoring of trends and determinants in CArdiovascular disease as it appears in Singh GM, Danaei G, Farzadfar F, Stevens GA, Woodward M, Wormser D, Kaptoge S, Whitlock G, Qiao Q, Lewington S, Di Angelantonio E, Vander Hoorn S, Lawes CM, Ali MK, Mozaffarian D, Ezzati M; Global Burden of Metabolic Risk Factors of Chronic Diseases Collaborating Group; Asia-Pacific Cohort Studies Collaboration

(APCSC); Diabetes Epidemiology: Collaborative analysis of Diagnostic criteria in Europe (DECODE); Emerging Risk Factor Collaboration (ERFC); Prospective Studies Collaboration (PSC). The age-specific quantitative effects of metabolic risk factors on cardiovascular diseases and diabetes: a pooled analysis. PLoS One. 2013; 8(7): e65174
United Kingdom - Newcastle Heart Project as it appears in Singh GM, Danaei G, Farzadfar F, Stevens GA, Woodward M, Wormser D, Kaptoge S, Whitlock G, Qiao Q, Lewington S, Di Angelantonio E, Vander Hoorn S, Lawes CM, Ali MK, Mozaffarian D, Ezzati M; Global Burden of Metabolic Risk Factors of Chronic Diseases Collaborating Group; Asia-Pacific Cohort Studies Collaboration (APCSC); Diabetes Epidemiology: Collaborative analysis of Diagnostic criteria in Europe (DECODE); Emerging Risk Factor Collaboration (ERFC); Prospective Studies Collaboration (PSC). The age-specific quantitative effects of metabolic risk factors on cardiovascular diseases and diabetes: a pooled analysis. PLoS One. 2013; 8(7): e65174
MacInnis RJ, English DR, Hopper JL, Giles GG. Body size and composition and the risk of gastric and oesophageal adenocarcinoma. Int J Cancer. 2006; 118(10): 2628–31
Kortt MA, Dollery B. Religion and BMI in Australia. J Relig Health. 2014; 53(1): 217–28
Patterson KAE, Cleland V, Venn A, Blizzard L, Gall S. A cross-sectional study of geographic differences in health risk factors among young Australian adults: the role of socioeconomic position. BMC Public Health. 2014; 14: 1278
Buchmueller TC, Johar M. Obesity and health expenditures: evidence from Australia. Econ Hum Biol. 2015; 17: 42–58
Gill TK, Hill CL, Shanahan EM, Taylor AW, Appleton SL, Grant JF, Shi Z, Dal Grande E, Price K, Adams RJ. Vitamin D levels in an Australian population. BMC Public Health. 2014; 14: 1001
Russell J, Flood V, Yeatman H, Mitchell P. Prevalence and risk factors of food insecurity among a cohort of older Australians. J Nutr Health Aging. 2014; 18(1): 3–8
Ghaem Maralani H, Tai BC, Wong TY, Tai ES, Li J, Wang JJ, Mitchell P. The prognostic role of body mass index on mortality amongst the middle-aged and elderly: a competing risk analysis. Diabetes Res Clin Pract. 2014; 103(1): 42–50
Mitchell RJ, Lord SR, Harvey LA, Close JCT. Obesity and falls in older people: mediating effects of disease, sedentary behavior, mood, pain and medication use. Arch Gerontol Geriatr. 2015; 60(1): 52–8
Wang Y, Simpson JA, Wluka AE, Teichtahl AJ, English DR, Giles GG, Graves S, Cicuttini FM. Relationship between body adiposity measure and risk of primary knee replacement for osteoarthritis: a prospective cohort study. Arthritis Res Ther. 2009; 11(2): r31
Cochrane T, Davey RC, de Castella FR. Influence of school community and fitness on prevalence of overweight in Australian school children. Prev Med. 2015; 81: 433–7
Leech RM, McNaughton SA, Timperio A. Clustering of diet, physical activity and sedentary behaviour among Australian children: cross-sectional and longitudinal associations with overweight and obesity. Int J Obes (Lond). 2015; 39(7): 1079–85
Achat HM, Stubbs JM. Socio-economic and ethnic differences in the prevalence of overweight and obesity among school children. J Paediatr Child Health. 2014; 50(10): E77-84

O’Dea JA, Chiang H, Peralta LR. Socioeconomic patterns of overweight, obesity but not thinness persist from childhood to adolescence in a 6-year longitudinal cohort of Australian schoolchildren from 2007 to 2012. BMC Public Health. 2014; 14: 222
Niven P, Scully M, Morley B, Baur L, Crawford D, Pratt IS, Wakefield M, NaSSDA Study Team. What factors are associated with frequent unhealthy snack-food consumption among Australian secondary-school students?. Public Health Nutr. 2015; 18(12): 2153–60
O’Dea JA, Dibley MJ. Prevalence of obesity, overweight and thinness in Australian children and adolescents by socioeconomic status and ethnic cultural group in 2006 and 2012. Int J Public Health. 2014; 59(5): 819–28
Wong E, Woodward M, Stevenson C, Backholer K, Sarink D, Peeters A. Prevalence of disability in Australian elderly: Impact of trends in obesity and diabetes. Prev Med. 2016; 82: 105-12
Zeleke BM, Bell RJ, Billah B, Davis SR. Symptomatic pelvic floor disorders in community-dwelling older Australian women. Maturitas. 2016; 85: 34–41
Lombard C, Harrison C, Kozica S, Zoungas S, Ranasinha S, Teede H. Preventing Weight Gain in Women in Rural Communities: A Cluster Randomised Controlled Trial. PLoS Med. 2016; 13(1): e1001941
Olsen CM, Nagle CM, Whiteman DC, Purdie DM, Green AC, Webb PM. Body size and risk of epithelial ovarian and related cancers: a population-based case-control study. Int J Cancer. 2008; 123(2): 450-56
Nagle CM, Marquart L, Bain CJ, O\xe2\x80\x99Brien S, Lahmann PH, Quinn M, Oehler MK, Obermair A, Spurdle AB, Webb PM. Impact of Weight Change and Weight Cycling on Risk of Different Subtypes of Endometrial Cancer. Eur J Cancer. 2013; 49(12): 2717-26
MacInnis RJ, English DR, Hopper JL, Giles GG. Body Size and Composition and the Risk of Lymphohematopoietic Malignancies. J Natl Cancer Inst. 2005; 97(15): 1154–7
Parr CL, Batty GD, Lam TH, Barzi F, Fang X, Ho SC, Jee SH, Ansary-Moghaddam A, Jamrozik K, Ueshima H, Woodward M, Huxley RR. Body-mass index and cancer mortality in the Asia-Pacific Cohort Studies Collaboration: pooled analyses of 424 519 participants. Lancet Oncol. 2010; 11(8): 741–52
Teras LR, Kitahara CM, Birmann BM, Hartge PA, Wang SS, Robien K, Patel AV, Adami H-O, Weiderpass E, Giles GG, Singh PN, Alavanja M, Beane Freeman LE, Bernstein L, Buring JE, Colditz GA, Fraser GE, Gapstur SM, Gaziano JM, Giovannucci E, Hofmann JN, Linet MS, Neta G, Park Y, Peters U, Rosenberg PS, Schairer C, Sesso HD, Stampfer MJ, Visvanathan K, White E, Wolk A, Zeleniuch-Jacquotte A, de González AB, Purdue MP. Body size and multiple myeloma mortality: a pooled analysis of 20 prospective studies. Br J Haematol. 2014; 166(5): 667–76 as it appears in Simpson JA, MacInnis RJ, Peeters A, Hopper JL, Giles GG, English DR. A Comparison of Adiposity Measures as Predictors of All-cause Mortality: The Melbourne Collaborative Cohort Study. Obesity (Silver Spring). 2007; 15(4): 994
Yap CH, Zimmet A, Mohajeri M, Yii M. Effect of obesity on early morbidity and mortality following cardiac surgery. Heart Lung Circ. 2007; 16(1): 31-6
Tan JSL, Wang JJ, Mitchell P. Influence of diabetes and cardiovascular disease on the long-term incidence of cataract: the Blue Mountains eye study. Ophthalmic Epidemiol. 2008; 15(5): 317–27
Henry MJ, Pasco JA, Korn S, Gibson JE, Kotowicz MA, Nicholson GC. Bone mineral density reference ranges for Australian men: Geelong Osteoporosis Study. Osteoporos Int. 2010; 21(6): 909-17

Devine A, Dick IM, Islam AF, Dhaliwal SS, Prince RL. Protein consumption is an important predictor of lower limb bone mass in elderly women. Am J Clin Nutr. 2005; 81(6): 1423-8
Henry MJ, Pasco JA, Nicholson GC, Seeman E, Kotowicz MA. Prevalence of osteoporosis in Australian women: Geelong Osteoporosis Study. J Clin Densitom. 2000; 3(3): 261-8
Jones G, Nguyen T, Sambrook P, Kelly PJ, Eisman JA. Progressive loss of bone in the femoral neck in elderly people: longitudinal findings from the Dubbo osteoporosis epidemiology study. BMJ. 1994; 309(6956): 691-5
Flicker L, Green R, Kaymakci B, Lichtenstein M, Buirski G, Wark JD. Do Australian women have greater spinal bone density than North American women?. Osteoporos Int. 1995; 5(1): 63-5
Nguyen TV, Eisman JA, Kelly PJ, Sambrook PN. Risk factors for osteoporotic fractures in elderly men. Am J Epidemiol. 1996; 144(3): 255–63
Bleicher K, Cumming RG, Naganathan V, Seibel MJ, Blyth FM, Le Couteur DG, Handelsman DJ, Creasey HM, Waite LM. Predictors of the rate of BMD loss in older men: findings from the CHAMP study. Osteoporos Int. 2013; 24(7): 1951-63
Euromonitor International. Euromonitor Passport - Fresh Foods Market Statistics. London, United Kingdom: Euromonitor International
Hendrie GA, Ridoutt BG, Wiedmann TO, Noakes M. Greenhouse gas emissions and the Australian diet--comparing dietary recommendations with average intakes. Nutrients. 2014; 6(1): 289–303
Hendrie GA, Riley MD. Performance of short food questions to assess aspects of the dietary intake of Australian children. Nutrients. 2013; 5(12): 4822–35
Gopinath B, Flood VM, Rochtchina E, Baur LA, Louie JCY, Smith W, Mitchell P. Carbohydrate nutrition and development of adiposity during adolescence. Obesity (Silver Spring). 2013; 21(9): 1884–90
Giskes K, Turrell G, Patterson C, Newman B. Socioeconomic differences among Australian adults in consumption of fruit and vegetables and intakes of vitamins A, C and folate. J Hum Nutr Diet. 2002; 15(5): 375-85
Hodge AM, English DR, O'Dea K, Giles GG. Glycemic index and dietary fiber and the risk of type 2 diabetes. Diabetes Care. 2004; 27(11): 2701-6
Food and Agriculture Organization of the United Nations (FAO). FAOSTAT Food Balance Sheets, October 2014. Rome, Italy: Food and Agriculture Organization of the United Nations (FAO)
Galea LM, Beck EJ, Probst YC, Cashman CJ. Whole grain intake of Australians estimated from a cross-sectional analysis of dietary intake data from the 2011-13 Australian Health Survey. Public Health Nutr. 2017; 1–7
Ollis TE, Meyer BJ, Howe PR. Australian food sources and intakes of omega-6 and omega-3 polyunsaturated fatty acids. Ann Nutr Metab. 1999; 43(6): 346-55
Gopinath B, Flood VM, Burlutksy G, Mitchell P. Consumption of nuts and risk of total and cause-specific mortality over 15 years. Nutr Metab Cardiovasc Dis. 2015; 25(12): 1125-31
Liu J, Arcot J, Cunningham J, Greenfield H, Hsu J, Padula D, Strobel N, Fraser DR. New data for vitamin D in Australian foods of animal origin: impact on

estimates of national adult vitamin D intakes in 1995 and 2011-13. Asia Pac J Clin Nutr. 2015; 24(3): 464–71
English DR, MacInnis RJ, Hodge AM, Hopper JL, Haydon AM, Giles GG. Red meat, chicken, and fish consumption and risk of colorectal cancer. Cancer Epidemiol Biomarkers Prev. 2004; 13(9): 1509–14
Jamrozik K, Broadhurst RJ, Forbes S, Hankey GJ, Anderson CS. Predictors of death and vascular events in the elderly : the Perth Community Stroke Study. Stroke. 2000; 31(4): 863-8
Burke V, Zhao Y, Lee AH, Hunter E, Spargo RM, Gracey M, Smith RM, Beilin LJ, Puddey IB. Health-related behaviours as predictors of mortality and morbidity in Australian Aborigines. Prev Med. 2007; 44(2): 135–42
Rosilene WVR, Cumming R, Travison T, Blyth F, Naganathan V, Allman-Farinelli M, Hirani V. Relative Validity of a Diet History Questionnaire Against a Four-Day Weighed Food Record among Older Men in Australia: The Concord Health and Ageing in Men Project (CHAMP). J Nutr Health Aging. 2015; 19(6): 603–10
Williams P. Breakfast and the diets of Australian children and adolescents: an analysis of data from the 1995 National Nutrition Survey. Int J Food Sci Nutr. 2007; 58(3): 201–16
Flood VM, Burlutsky G, Webb KL, Wang JJ, Smith WT, Mitchell P. Food and nutrient consumption trends in older Australians: a 10-year cohort study. Eur J Clin Nutr. 2010; 64(6): 603–13
Rangan AM, Randall D, Hector DJ, Gill TP, Webb KL. Consumption of “extra” foods by Australian children: types, quantities and contribution to energy and nutrient intakes. Eur J Clin Nutr. 2008; 62(3): 356–64
Hopping BN, Erber E, Grandinetti A, Verheus M, Kolonel LN, Maskarinec G. Dietary fiber, magnesium, and glycemic load alter risk of type 2 diabetes in a multiethnic cohort in Hawaii. J Nutr. 2010; 140(1): 68-74
Barclay AW, Flood VM, Rochtchina E, Mitchell P, Brand-Miller JC. Glycemic index, dietary fiber, and risk of type 2 diabetes in a cohort of older Australians. Diabetes Care. 2007; 30(11): 2811-3
Kaushik S, Wang JJ, Wong TY, Flood V, Barclay A, Brand-Miller J, Mitchell P. Glycemic index, retinal vascular caliber, and stroke mortality. Stroke. 2009; 40(1): 206-12
Buyken AE, Flood V, Empson M, Rochtchina E, Barclay AW, Brand-Miller J, Mitchell P. Carbohydrate nutrition and inflammatory disease mortality in older adults. Am J Clin Nutr. 2010; 92(3): 634-43
Fayet-Moore F, Cassettari T, Tuck K, McConnell A, Petocz P. Dietary Fibre Intake in Australia. Paper I: Associations with Demographic, Socio-Economic, and Anthropometric Factors. Nutrients. 2018; 10(5)
Swierk M, Williams PG, Wilcox J, Russell KG, Meyer BJ. Validation of an Australian electronic food frequency questionnaire to measure polyunsaturated fatty acid intake. Nutrition. 2011; 27(6): 641–6
Howe P, Meyer B, Record S, Baghurst K. Dietary intake of long-chain omega-3 polyunsaturated fatty acids: contribution of meat sources. Nutrition. 2006; 22(1): 47-53

Kinlay S, Elliott H, Heller RF. Identifying individuals with high fat levels and low P:S ratios, in their diets, for intensive dietary intervention. J Intern Med. 1997; 241(5): 407-14
Olstad DL, Leech RM, Livingstone KM, Ball K, Thomas B, Potter J, Cleanthous X, Reynolds R, McNaughton SA. Are dietary inequalities among Australian adults changing? a nationally representative analysis of dietary change according to socioeconomic position between 1995 and 2011-13. Int J Behav Nutr Phys Act. 2018; 15(1): 30
Ramsden CE, Zamora D, Leelarthaepin B, Majchrzak-Hong SF, Faurot KR, Suchindran CM, Ringel A, Davis JM, Hibbeln JR. Use of dietary linoleic acid for secondary prevention of coronary heart disease and death: evaluation of recovered data from the Sydney Diet Heart Study and updated meta-analysis. BMJ. 2013; 346: e8707
World Health Organization (WHO). Sodium Intakes Around the World. 2007
Ekinci EI, Clarke S, Thomas MC, Moran JL, Cheong K, MacIsaac RJ, Jerums G. Dietary salt intake and mortality in patients with type 2 diabetes. Diabetes Care. 2011; 34(3): 703-9
Bauman A, Bull F, Chey T, Craig CL, Ainsworth BE, Sallis JF, Bowles HR, Hagstromer M, Sjostrom M, Pratt M, IPS Group. The International Prevalence Study on Physical Activity: results from 20 countries. Int J Behav Nutr Phys Act. 2009; 21
Australian Institute of Health and Welfare, Hunter Valley Research Foundation. Australia National Physical Activity Survey 1999
New South Wales Department of Health. Australia - New South Wales Population Health Survey 2002
New South Wales Department of Health. Australia - New South Wales Population Health Survey 2003
New South Wales Department of Health. Australia - New South Wales Population Health Survey 2004
New South Wales Department of Health. Australia - New South Wales Population Health Survey 2005
New South Wales Department of Health. Australia - New South Wales Population Health Survey 2006
New South Wales Department of Health. Australia - New South Wales Population Health Survey 2007
New South Wales Department of Health. Australia - New South Wales Population Health Survey 2008
New South Wales Department of Health. Australia - New South Wales Population Health Survey 2009
New South Wales Department of Health. Australia - New South Wales Population Health Survey 2010
New South Wales Department of Health. Australia - New South Wales Population Health Survey 2011
New South Wales Department of Health. Australia - New South Wales Population Health Survey 2012
Magliano DJ, Barr EL, Zimmet PZ, Cameron AJ, Dunstan DW, Colagiuri S, Jolley D, Owen N, Phillips P, Tapp RJ, Welborn TA, Shaw JE. Glucose indices, health behaviors, and incidence of diabetes in Australia: the Australian Diabetes, Obesity and Lifestyle Study. Diabetes Care. 2008; 31(2): 267-72
Lucke J, Waters B, Hockey R, Spallek M, Gibson R, Byles J, Dobson A. Trends in women's risk factors and chronic conditions: findings from the Australian Longitudinal Study on Women's Health. Women Health. 2007; 3(4): 423-32

ISSP Research Group (2009): International Social Survey Programme: Leisure Time and Sports - ISSP 2007. GESIS Data Archive, Cologne. ZA4850 Data file version 2.0.0, doi:10.4231/1.10079
International Labour Organization (ILO). International Labour Organization Database (ILOSTAT) - Employment by Sex and Economic Activity. International Labour Organization (ILO)
International Labour Organization (ILO). International Labour Organization Database (ILOSTAT) - Employment by Sex and Occupation. International Labour Organization (ILO)
International Social Survey Programme (ISSP). Australia International Social Survey Programme: Social Inequality II 1993. Mannheim, Germany: GESIS - Leibniz Institute for the Social Sciences, 1994
International Labour Organization (ILO). International Labour Organization Database (ILOSTAT) - Employment to Population Ratio by Sex and Age. International Labour Organization (ILO)
ISSP Research Group (1997): International Social Survey Programme: Family and Changing Gender Roles II - ISSP 1994. GESIS Data Archive, Cologne. ZA2620 Data file Version 1.0.0, doi:10.4232/1.2620
International Social Survey Programme (ISSP). Australia International Social Survey Programme: National Identity I 1994-1996. Mannheim, Germany: GESIS - Leibniz Institute for the Social Sciences, 1998
International Social Survey Programme (ISSP). Australia International Social Survey Programme: Role of Government III 1997. Mannheim, Germany: GESIS - Leibniz Institute for the Social Sciences, 1999
International Social Survey Programme (ISSP). Australia International Social Survey Programme: Social Inequality III 1999-2000. Mannheim, Germany: GESIS - Leibniz Institute for the Social Sciences, 2002
ISSP Research Group (2012): International Social Survey Programme: National Identity II - ISSP 2003. GESIS Data Archive, Cologne. ZA3910 Data file Version 2.1.0, doi:10.4232/1.11449
International Social Survey Programme (ISSP). Australia International Social Survey Programme: Citizenship 2005. Mannheim, Germany: GESIS - Leibniz Institute for the Social Sciences, 2012
International Social Survey Programme (ISSP). Australia International Social Survey Programme: Work Orientation III 2005. Mannheim, Germany: GESIS - Leibniz Institute for the Social Sciences, 2013
ISSP Research Group (2008): International Social Survey Programme: Role of Government IV - ISSP 2006. GESIS Data Archive, Cologne. ZA4700 Data file Version 1.0.0, doi:10.4232/1.4700
International Social Survey Programme (ISSP). Australia International Social Survey Programme: Religion III 2009-2010. Mannheim, Germany: GESIS - Leibniz Institute for the Social Sciences, 2012
ISSP Research Group (2017): International Social Survey Programme: Social Inequality IV - ISSP 2009. GESIS Data Archive, Cologne. ZA5400 Data file Version 4.0.0, doi:10.4232/1.12777.

ISSP Research Group (2016): International Social Survey Programme: Family and Changing Gender Roles IV - ISSP 2012. GESIS Data Archive, Cologne. ZA5900 Data file Version 4.0.0, doi:10.4232/1.12661
ISSP Research Group (2000): International Social Survey Programme: Religion II - ISSP 1998. GESIS Data Archive, Cologne. ZA3190 Data file Version 1.0.0, https://doi.org/10.4232/1.3190
International Social Survey Programme (ISSP). Australia International Social Survey Program: Environment I 1992-1994. Mannheim, Germany: GESIS - Leibniz Institute for the Social Sciences, 1995
ISSP Research Group (2003): International Social Survey Programme: Social Relations and Support Systems / Social Networks II - ISSP 2001. GESIS Data Archive, Cologne. ZA3680 Data file Version 1.0.0, doi:10.4232/1.3680
ISSP Research Group (2016): International Social Survey Programme: Citizenship II - ISSP 2014. GESIS Data Archive, Cologne. ZA6670 Data file Version 2.0.0, doi:10.4232/1.12590
The International Social Survey Programme (ISSP) represents a series of cross-national collaborations between organizations conducting similar social science research. ISSP researchers focus on developing survey questions that are relevant and comprehensible in all countries and languages, with survey results that are comparable across nations.Topics covered on this round of the ISSP include: working conditions; labor relations and conflict; and employment and unemployment
International Labour Organization (ILO). International Labour Organization Database (ILOSTAT) - Fatal Injuries by Sex and Economic Activity. International Labour Organization (ILO)
Henstridge J, Data Analysis Australia. Australia Analysis of the 2005 Personal Safety Survey. Nedlands, Australia: Data Analysis Australia, 2007
Australian Bureau of Statistics. Australia Personal Safety Survey 2005
Bergen HA, Martin G, Richardson AS, Allison S, Roeger L. Sexual Abuse and Suicidal Behavior: A Model Constructed From a Large Community Sample of Adolescents. J Am Acad Child Adolesc Psychiatry. 2003; 42(11): 1301-9
Dunne MP, Purdie DM, Cook MD, Boyle FM, Najman JM. Is child sexual abuse declining? Evidence from a population-based survey of men and women in Australia. Child Abuse Negl. 2003; 27(2): 141-52
Goldman JD, Padayachi UK. The prevalence and nature of child sexual abuse in Queensland, Australia. Child Abuse Negl. 1997; 21(5): 489-98
Jaquier V, Fisher BS, Johnson H. Prevalence and Risks of Physical and Sexual Violence against Women by Nonintimates: An Exploratory Study across Nine Countries. Vict Offender. 2011; 6(4): 399-415
Moore EE, Romaniuk H, Olsson CA, Jayasinghe Y, Carlin JB, Patton GC. The prevalence of childhood sexual abuse and adolescent unwanted sexual contact among boys and girls living in Victoria, Australia. Child Abuse Negl. 2010; 34(5): 379-85
Schei B, Guthrie JR, Dennerstein L, Alford S. Intimate partner violence and health outcomes in mid-life women: a population-based cohort study. Arch Womens Ment Health. 2006; 9(6): 317-24
European Institute for Crime Prevention and Control, affiliated with the United Nations (HEUNI), United Nations Office on Drugs and Crime (UNODC),

Statistics Canada, United Nations Interregional Crime and Justice Research Institute (UNICRI). International Violence Against Women Surveys (IVAWS) Data 2002-2005. As provided by the Global Burden of Disease Child Sexual Abuse and Intimate Partner Violence Expert Group. [Unpublished]
de Visser RO, Smith AM, Rissel CE, Richters J, Grulich AE. Sex in Australia: experiences of sexual coercion among a representative sample of adults. Aust N Z J Public Health. 2003; 27(2): 198-203
Moore EE, Jayasinghe Y, Olsson CA, Romaniuk H, Sasongko V, Patton GC, Garland SM. Assessment of first sexual intercourse in young women with a history of childhood sexual abuse. Aust N Z J Obstet Gynaecol. 2011; 51(3): 276-79
Dinwiddie S, Heath AC, Dunne MP, Bucholz KK, Madden PA, Slutske WS, Bierut LJ, Statham DB, Martin NG. Early sexual abuse and lifetime psychopathology: a co-twin-control study. Psychol Med. 2000; 30(1): 41–52
Nelson EC, Heath AC, Madden PA, Cooper ML, Dinwiddie SH, Bucholz KK, Glowinski A, McLaughlin T, Dunne MP, Statham DJ, Martin NG. Association between self-reported sexual abuse and adverse psychosocial outcomes: results from a twin study. Arch Gen Psychiatry. 2002; 59(2): 139-45
Fleming J, Mullen PE, Sibthorpe B, Attewell R, Bammer G. The relationship between childhood sexual abuse and alcohol abuse in women-a case-control study. Addiction. 1998; 93(12): 1787
Australian Bureau of Statistics. Australia Women's Safety Survey 1996
Grande ED, Hickling J, Taylor A, Woollacott T. Domestic violence in South Australia: a population survey of males and females. Aust N Z J Public Health. 2003; 27(5): 543-50
Taft AJ, Watson LF, Lee C. Violence against young Australian women and association with reproductive events: a cross-sectional analysis of a national population sample. Aust N Z J Public Health. 2004; 28(4): 324-9
Australian Institute of Criminology. Australia Homicide between Intimate Partners. Canberra, Australia: Australian Institute of Criminology, 1998
Australian Institute of Criminology. Australia National Homicide Monitoring Program Annual Report 2005-2006 . Canberra, Australia: Australian Institute of Criminology, 2007
Australian Institute of Criminology. Australia National Homicide Monitoring Program Annual Report 2006-2007. Canberra, Australia: Australian Institute of Criminology, 2008
Sanmartin J, Molina A, Garcia Y, Queen Sofía Center for the Study of Violence. Partner Violence against Women: Statistics and Legislation, International Report 2003. Valencia, Spain: Queen Sofía Center for the Study of Violence, 2003
Queen Sofía Center for the Study of Violence. Partner Violence Against Women: Statistics and Legislation, 3rd International Report 2010. Valencia, Spain: Queen Sofía Center for the Study of Violence, 2010
Lynch M, Black M. A tale of two cities: a review of homicide in Melbourne and Glasgow in 2005. Med Sci Law. 2008; 48(1): 24-30
Shackelford TK, Mouzos J. Partner killing by men in cohabiting and marital relationships: a comparative, cross-national analysis of data from Australia and the United States. J Interpers Violence. 2005; 20(10): 1310-24

Mouzos J, Australian Institute of Criminology. Homicidal Encounters: A Study of Homicide in Australia 1989-1999. Canberra, Australia: Australian Institute of Criminology, 2000
Loxton D, Schofield M, Hussain R. History of domestic violence and health service use among mid-aged Australian women. Aust N Z J Public Health. 2004; 28(4): 383-8
Loxton D, Schofield M, Hussain R. Psychological health in midlife among women who have ever lived with a violent partner or spouse. J Interpers Violence. 2006; 21(8): 1092-107
Queensland Health. Australia - Queensland Infant Nutrition Project 2006-2007
Department of Education and Early Childhood Development (Victoria, Australia), Department of Human Services (Victoria, Australia). Australia - The State of Victoria's Children Report 2006. Melbourne, Australia: Department of Human Services (Victoria, Australia), 2006
Australian Bureau of Statistics. Breastfeeding in Australia, 2001. Canberra, Australia: Australian Bureau of Statistics, 2003
Australian Bureau of Statistics. Health of Children in Australia: A Snapshot, 2004-2005. Canberra, Australia: Australian Bureau of Statistics, 2007
Kirby Institute, University of New South Wales. Australia HIV, Viral Hepatitis, and Sexually Transmissible Infections Annual Surveillance Report 2013. Darlinghurst, Australia: Kirby Institute, University of New South Wales, 2013
Department of Health (Australia), Joint United Nations Program on HIV/AIDS (UNAIDS), Kirby Institute, University of New South Wales. Australia Global AIDS Response Country Progress Report 2015. Geneva, Switzerland: Joint United Nations Program on HIV/AIDS (UNAIDS), 2015
Roberts L, Jorm L, Patel M, Smith W, Douglas RM, McGilchrist C. Effect of infection control measures on the frequency of diarrheal episodes in child care: a randomized, controlled trial. Pediatrics. 2000; 105(4 Pt 1): 743-6
Roberts L, Smith W, Jorm L, Patel M, Douglas RM, McGilchrist C. Effect of infection control measures on the frequency of upper respiratory infection in child care: a randomized, controlled trial. Pediatrics. 2000; 105(4 Pt 1): 738–42
European Centre for Medium-Range Weather Forecasts (ECMWF). European Reanalysis-5. United Kingdom: European Centre for Medium-Range Weather Forecasts (ECMWF)
Australian Institute of Health and Welfare. Australia's Mothers and Babies 1991. Canberra, Australia: Australian Institute of Health and Welfare, 1994
Australian Institute of Health and Welfare. Australia's Mothers and Babies 1992. Canberra, Australia: Australian Institute of Health and Welfare, 1995
Australian Institute of Health and Welfare. Australia's Mothers and Babies 1993. Canberra, Australia: Australian Institute of Health and Welfare, 1996
Australian Institute of Health and Welfare. Australia's Mothers and Babies 1994. Canberra, Australia: Australian Institute of Health and Welfare, 1997
Australian Institute of Health and Welfare. Australia's Mothers and Babies 1995. Canberra, Australia: Australian Institute of Health and Welfare, 1997
Australian Institute of Health and Welfare. Australia's Mothers and Babies 1996. Canberra, Australia: Australian Institute of Health and Welfare, 1999
Australian Institute of Health and Welfare, National Perinatal Epidemiology and Statistics Unit (NPESU), University of New South Wales. Australia's Mothers and Babies 1997. Canberra, Australia: Australian Institute of Health and Welfare, 1999

Australian Institute of Health and Welfare, National Perinatal Epidemiology and Statistics Unit (NPESU), University of New South Wales. Australia's Mothers and Babies 1998. Canberra, Australia: Australian Institute of Health and Welfare, 2000
Australian Institute of Health and Welfare, National Perinatal Epidemiology and Statistics Unit (NPESU), University of New South Wales. Australia's Mothers and Babies 1999. Canberra, Australia: Australian Institute of Health and Welfare, 2001
Australian Institute of Health and Welfare, National Perinatal Epidemiology and Statistics Unit (NPESU), University of New South Wales. Australia's Mothers and Babies 2000. Canberra, Australia: Australian Institute of Health and Welfare, 2003
Australian Institute of Health and Welfare, National Perinatal Epidemiology and Statistics Unit (NPESU), University of New South Wales. Australia's Mothers and Babies 2001. Canberra, Australia: Australian Institute of Health and Welfare, 2004
Australian Institute of Health and Welfare, National Perinatal Epidemiology and Statistics Unit (NPESU), University of New South Wales. Australia's Mothers and Babies 2002. Canberra, Australia: Australian Institute of Health and Welfare, 2004
Australian Institute of Health and Welfare, National Perinatal Epidemiology and Statistics Unit (NPESU), University of New South Wales. Australia's Mothers and Babies 2003. Canberra, Australia: Australian Institute of Health and Welfare, 2005
Australian Institute of Health and Welfare, National Perinatal Epidemiology and Statistics Unit (NPESU), University of New South Wales. Australia's Mothers and Babies 2004. Canberra, Australia: Australian Institute of Health and Welfare, 2006
Australian Institute of Health and Welfare, National Perinatal Epidemiology and Statistics Unit (NPESU), University of New South Wales. Australia's Mothers and Babies 2005. Canberra, Australia: Australian Institute of Health and Welfare, 2007
Australian Institute of Health and Welfare, National Perinatal Epidemiology and Statistics Unit (NPESU), University of New South Wales. Australia's Mothers and Babies 2006. Canberra, Australia: Australian Institute of Health and Welfare, 2008
Australian Institute of Health and Welfare, National Perinatal Epidemiology and Statistics Unit (NPESU), University of New South Wales. Australia's Mothers and Babies 2007. Canberra, Australia: Australian Institute of Health and Welfare, 2009
Australian Institute of Health and Welfare, National Perinatal Epidemiology and Statistics Unit (NPESU), University of New South Wales. Australia's Mothers and Babies 2008. Canberra, Australia: Australian Institute of Health and Welfare, 2010
Australian Institute of Health and Welfare, National Perinatal Epidemiology and Statistics Unit (NPESU), University of New South Wales. Australia's Mothers and Babies 2010. Canberra, Australia: Australian Institute of Health and Welfare, 2012
Australian Institute of Health and Welfare, National Perinatal Epidemiology and Statistics Unit (NPESU), University of New South Wales. Australia's Mothers and Babies 2009. Canberra, Australia: Australian Institute of Health and Welfare, 2011
Australian Institute of Health and Welfare, National Perinatal Epidemiology and Statistics Unit (NPESU), University of New South Wales. Australia's Mothers and Babies 2011. Canberra, Australia: Australian Institute of Health and Welfare, 2013
Australian Institute of Health and Welfare, National Perinatal Epidemiology and Statistics Unit (NPESU), University of New South Wales. Australia's Mothers and Babies 2012. Canberra, Australia: Australian Institute of Health and Welfare, 2014

Lack BM, Smith RM, Arundell MJ, Homer CS. Narrowing the Gap? Describing women's outcomes in Midwifery Group Practice in remote Australia. Women Birth. 2016; 29(5): 465-470
Farrant BM, Stanley FJ, Hardelid P, Shepherd CC. Stillbirth and neonatal death rates across time: the influence of pregnancy terminations and birth defects in a Western Australian population-based cohort study. BMC Pregnancy Childbirth. 2016; 16: 112
Hagan R, Benninger H, Chiffings D, Evans S, French N. Very preterm birth--a regional study. Part 2: The very preterm infant. Br J Obstet Gynaecol. 1996; 103(3): 239-45
Drew JH, Beischer NA, Walstab JE, Liang ST, Stronge JM. Contribution of prematurity and low birth-weight to neonatal death in Chinese and Western populations. Aust N Z J Obstet Gynaecol. 1988; 28(3): 190-2
Atkins R, Polkinghorne K, Chadban S, Chronic Kidney Disease Prognosis Consortium (CKD-PC). Australia Diabetes, Obesity and Lifestyle (AusDiab) Chronic Kidney Disease Prevalence Data 1999-2000 [Unpublished]
Mamun AA, O'Callaghan MJ, Williams GM, Najman JM. Adolescents bullying and young adults body mass index and obesity: a longitudinal study. Int J Obes (Lond). 2013; 37(8): 1140–6
Bond L, Wolfe S, Tollit M, Butler H, Patton G. A comparison of the Gatehouse Bullying Scale and the peer relations questionnaire for students in secondary school. J Sch Health. 2007; 77(2): 75–9
Lester L, Mander DJ, Cross D. Bullying Behaviour Following Students? Transition to a Secondary Boarding School Context. Int J Child Adolesc Health. 2015; 8(2): 141-50
Thomas HJ, Connor JP, Lawrence DM, Hafekost JM, Zubrick SR, Scott JG. Prevalence and correlates of bullying victimisation and perpetration in a nationally representative sample of Australian youth. Aust N Z J Psychiatry. 2017; 51(9): 909–20
Thomas HJ, Chan GC, Scott JG, Connor JP, Kelly AB, Williams J. Association of different forms of bullying victimisation with adolescents' psychological distress and reduced emotional wellbeing. Aust N Z J Psychiatry. 2016; 50(4): 371–9
Rigby K. Peer victimisation at school and the health of secondary school students. Br J Educ Psychol. 1999; 69 (Pt 1): 95–104
Moore SE, Norman RE, Sly PD, Whitehouse AJO, Zubrick SR, Scott J. Adolescent peer aggression and its association with mental health and substance use in an Australian cohort. J Adolesc. 2014; 37(1): 11–21
Patton GC, Olsson C, Bond L, Toumbourou JW, Carlin JB, Hemphill SA, Catalano RF. Predicting female depression across puberty: a two-nation longitudinal study. J Am Acad Child Adolesc Psychiatry. 2008; 47(12): 1424–32
Hemphill SA, Kotevski A, Herrenkohl TI, Bond L, Kim MJ, Toumbourou JW, Catalano RF. Longitudinal consequences of adolescent bullying perpetration and victimisation: A study of students in Victoria, Australia. Crim Behav Ment Health. 2011; 21(2): 107–16
Hemphill SA, Kotevski A, Heerde JA. Longitudinal associations between cyber-bullying perpetration and victimization and problem behavior and mental health problems in young Australians. Int J Public Health. 2015; 60(2): 227–37
Vassallo S, Edwards B, Renda J, Olsson CA. Bullying in Early Adolescence and Antisocial Behavior and Depression Six Years Later: What Are the

Protective Factors?. J Sch Violence. 2014; 13(1): 100–24
Simons LA, Friedlander Y, McCallum J, Simons J, Powell I, Heller R, Berry G. The Dubbo study of the health of elderly: correlates of coronary heart disease at study entry. J Am Geriatr Soc. 1991; 39(6): 584-90
Mèantyselkèa P, Miettola J, Niskanen L, Kumpusalo E. Glucose regulation and chronic pain at multiple sites. Rheumatology (Oxford). 2008; 47(8): 1235-8

DisMod-MR model development and validation:

The DisMod-MR tool evaluated and pooled all available data, adjusted data for systematic bias associated with methods that varied from the reference, and produced estimates by world regions with UIs by using Bayesian statistical methods. In cross-validation tests, the log rates specification worked as well or better than the negative binomial specification. The sequence of estimation occurs at five levels: global, super-region, region, country and, where applicable, subnational location. The super-region priors are generated at the global level with mixed-effects, nonlinear regression by using all available data; the super-region fit, in turn, informs the region fit, and so on down the cascade. The wrapper gives analysts the choice to branch the cascade in terms of time and sex at different levels depending on data density. The default used in most models is to branch by sex after the global fit but to retain all years of data until the lowest level in the cascade is reached. The computational engine is limited to three levels of random effects; we differentiate estimates at the super-region, region and country level. The coefficients for country covariates are re-estimated at each level of the cascade. For a given location, country coefficients are calculated by using both data and prior information available for that location. In the absence of data, the coefficient of its parent location is used to utilise the predictive power of our covariates in data-sparse situations. To determine the robustness of the models, we included the option again to have random effects on cause-specific mortality rates (CSMR) and EMR. Based on simulation testing we found that coverage improved and errors reduced when passing down priors with a wider setting of minimum coefficient of variation (which determines the uncertainty around priors and hence how ‘informative’ the priors are) than had generally been used in past GBD iterations. We settled on a default value of 0.8.

We carried out simulation testing using DisMod-MR 2.1 based on an internally consistent set of 15,601 data points for prevalence, incidence, excess mortality, CSMR, and remission. We aimed to test what level of minimum CV would create the best fit based on the following three performance statistics:

(1) Coverage, ie, the proportion of data point mean values that fall between the 2.5th and 97.5th
percentile of the draws of the fit values;

(2) Root mean square error: the square root of the mean of the squares of the difference between data point mean values and the mean fit value; and

(3) Bias: the difference between the mean fit value and the data point mean value.

We created different datasets culling the initial complete set with values at every age, sex, and location to more realistic data sparsity scenarios for analysis.

A first strategy was to randomly reduce the dataset to 10%, 5%, 2.5%, 1%, and 0.5% of the original data points. Initial results indicated little variation between the data samples culled to 10%, 5%, 2.5%, and 1%. The 0.5% culled dataset was an exception with markedly worse performance statistics, particularly with regard to bias and RMSE. We conducted further studies using the datasets culled to 10%, 5%, and 0.5%.

For some causes such as HIV/AIDS or measles, disease-specific natural history models have been used for which the underlying three state model in DisMod-MR 2.1 (susceptible, cases, dead) is insufficient to capture the complexity of a disease process. For some diseases with a range of sequelae differentiated by severity, such as COPD or diabetes mellitus, DisMod-MR 2.1 was used to meta-analyse the data on overall prevalence with separate DisMod-MR 2.1 models of the proportions of cases with different severity levels or sequelae. Likewise, DisMod-MR 2.1 was used to meta-analyse data on the proportions of liver cancer and cirrhosis due to underlying aetiologies such as hepatitis B, hepatitis C, and alcohol use. The GBD modelling software has been previously validated by comparing projected estimates with observed data for 2014-16. Model performance was assessed using mean error and root-mean-squared error. The model performed better than the most commonly used approach, i.e. the Lee-Carter method.
